# Supplementary material for: Synthesis and Antiproliferative Activity of 2,4,6,7-Tetrasubstituted-2H-pyrazolo[4,3-c]pyridines
Source: Molecules. 2021 Nov 8;26(21):6747. doi: 10.3390/molecules26216747 (PMC8588486; doi:10.3390/molecules26216747)
Supplement: Supplementary file 1 [file molecules-26-06747-s001.zip › molecules-1447331-supplementary.pdf]

Supplementary Materials

Synthesis and Antiproliferative Activity of 2,4,6,7-Tetrasubstituted-2*H*-pyrazolo[4,3-*c*]pyridines

Beatričė Razmienė[1,2], Eva Řezníčková[3], Vaida Dambrauskienė[1], Radek Ostruszka[4], Martin Kubala[4], Asta Žukauskaitė[5,*], Vladimír Kryštof[3,6], Algirdas Šačkus[1,2], Eglė Arbačiauskienė[1,*]

[1] Department of Organic Chemistry, Kaunas University of Technology, Radvilėnų pl. 19, LT-50254 Kaunas, Lithuania

[2] Institute of Synthetic Chemistry, Kaunas University of Technology, K. Baršausko g. 59, LT-51423 Kaunas, Lithuania

[3] Department of Experimental Biology, Palacký University, Šlechtitelů 27, CZ-78371 Olomouc, Czech Republic

[4] Department of Experimental Physics, Faculty of Science, Palacký University, 17. listopadu 12, CZ-77146 Olomouc, Czech Republic

[5] Department of Chemical Biology, Palacký University, Šlechtitelů 27, CZ-78371 Olomouc, Czech Republic

[6] Institute of Molecular and Translational Medicine, Faculty of Medicine and Dentistry, Palacký University, Hněvotínská 5, CZ-77900 Olomouc, Czech Republic

* Corresponding authors: asta.zukauskaite@upol.cz (A. Žukauskaitė), egle.arbaciauskiene@ktu.lt (E. Arbačiauskienė)

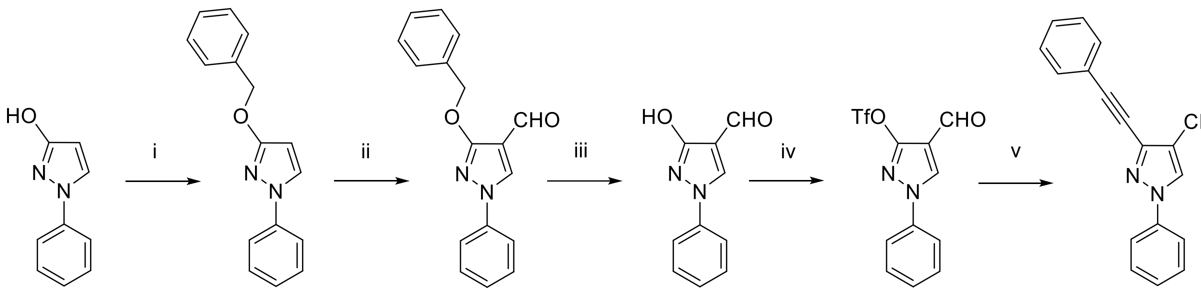

1 **A** **B** **C** **D** 2

Scheme S1. Synthesis of 1-phenyl-3-(phenylethynyl)-1*H*-pyrazole-4-carbaldehyde (**2**) by previously published procedures. *Reagents and conditions*: i: NaH, BnCl, DMF, argon atmosphere, 60 °C, 1 h; ii: $POCl_3$, DMF, 0 ºC for 10 min to 60 ºC for 30 min; iii: TFA, toluene, rt, 24 h; iv: Tf_2O, TEA, DCM, rt, 1 h.

Table S1. Fundamental absorption and fluorescence characteristics of compounds **18-42** in THF (λ_{ex} = 350 nm)

**Compound**	λ_{abs} **(nm)**	$\varepsilon\times10^3$ **($dm^3mol^{-1}cm^{-1}$)**	λ_{em} **(nm)**	**SS (nm)**	Φ_f **(%)**
**18**	260<br>312	30.38<br>13.23	442	130	18.9
**19**	261<br>311	34.67<br>16.86	447	136	26.2
**20**	260<br>311	35.55<br>16.29	437	126	53.2
**21**	263<br>310<br>351 shoulder	31.95<br>15.80<br>8.07	461	151	71.8
**22**	267<br>311<br>357 shoulder	32.04<br>16.09<br>7.74	478	167	56.8
**23**	265	28.94	467	202	72.7
**24**	261<br>311	36.46<br>17.34	449	138	17.6
**25**	261<br>310	29.68<br>15.11	449	139	30.0
**26**	263<br>310	38.05<br>17.48	450	140	48.7
**27**	265<br>317<br>349 shoulder	31.58<br>14.93<br>9.31	466	117	72.2
**28**	266<br>316<br>356 shoulder	30.31<br>13.14<br>7.28	481	125	62.8
**29**	264	24.96	465	201	74.8
**30**	262<br>312	32.79<br>14.13	449	137	21.3
**31**	265	35.93	464	199	73.1
**32**	264	39.45	465	201	64.7
**33**	263<br>308	35.01<br>14.49	449	141	52.2
**34**	260<br>313	21.84<br>10.61	453	140	0.9
**35**	260<br>312	45.49<br>21.11	445	133	7.0
**36**	262<br>314	37.32<br>16.56	455	141	14.2
**37**	266	36.30	470	204	61.7
**38**	265	41.18	467	202	68.9
**39**	269<br>369	29.03<br>9.84	487	118	39.7
**40**	265	48.93	469	204	83.5
**41**	265	36.45	467	202	69.9
**42**	265	42.38	470	205	84.6

Table S2. Fundamental absorption* and fluorescence characteristics of compounds **18-42** in Britton–Robinson buffer at pH 5, 7 and 9 (λ_{ex} = 360 nm)

**Compound**	**pH = 5**			**pH = 7**			**pH = 9**		
	λ_{abs} **(nm)**	λ_{em} **(nm)**	Φ_f **(%)**	λ_{abs} **(nm)**	λ_{em} **(nm)**	Φ_f **(%)**	λ_{abs} **(nm)**	λ_{em} **(nm)**	Φ_f **(%)**
**18**	259	447	40.4	254	439	16.0	253	436	22.1
**19**	258	455	4.4	253	459	33.4	253	458	34.3
**20**	261	444	2.0	255	440	6.5	255	439	5.7
**21**	259	497	12.2	256	458	37.2	256	458	59.9
**22**	257	507	0.4	256	511	4.3	256	495	7.1
**23**	259	445	0.1	255	461	0.1	255	469	0.1
**24**	261	443	81.3	254	442	17.7	254	435	17.7
**25**	259	486	2.0	253	456	18.2	252	455	43.1
**26**	262	446	0.4	257	446	6.1	256	440	6.6
**27**	264	509	17.0	257	459	22.0	258	458	24.0
**28**	257	499	0.2	256	504	9.6	255	495	14.2
**29**	262	446	0.3	255	466	0.2	254	454	0.2
**30**	261	444	77.5	260	438	14.0	262	436	15.3
**31**	262	511	18.2	269	457	35.3	267	457	37.8
**32**	257	497	1.3	255	491	13.5	255	485	14.9
**33**	263	459	35.7	265	449	17.9	266	448	17.4
**34**	264	438	8.6	266	439	8.7	264	440	10.1
**35**	267	438	10.1	269	440	9.9	267	438	11.1
**36**	265	443	15.3	266	441	14.1	265	443	17.6
**37**	264	443	0.4	254	495	0.1	255	475	0.2
**38**	269	457	28.9	270	458	35.6	272	458	36.0
**39**	285	479	26.6	280	481	31.5	277	480	34.3
**40**	266	493	10.1	269	481	15.0	268	482	17.4
**41**	271	474	11.6	272	472	17.0	270	470	19.1
**42**	269	458	18.5	271	459	33.6	270	458	37.5

*In aqueous solution, the absorption bands above 300 nm become extremely broad, and they are manifested as an extended shoulder, for which it is impossible to identify precise maximum position. Nevertheless, for all species, it was possible to use 360 nm as an excitation wavelength in fluorescence experiments in the examined pH range.

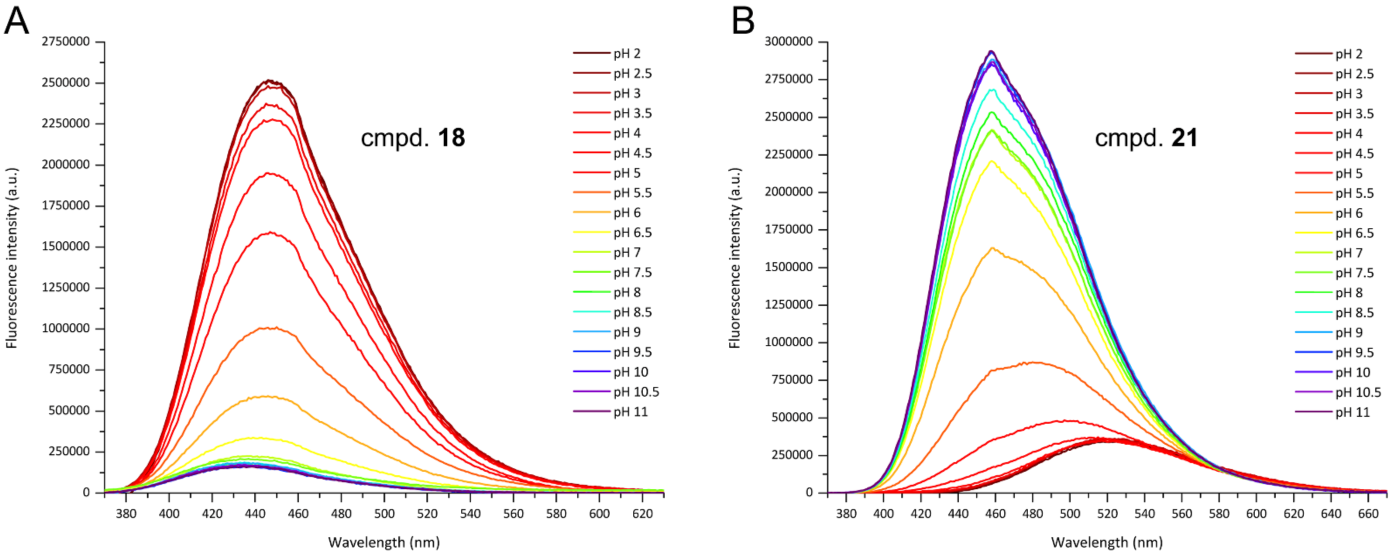

Figure S1. Fluorescence spectra (λ_{ex} = 360 nm) and titration profiles for compounds **18** (part A) and **21** (part B**)** in Britton–Robinson buffer at pH 2-11.

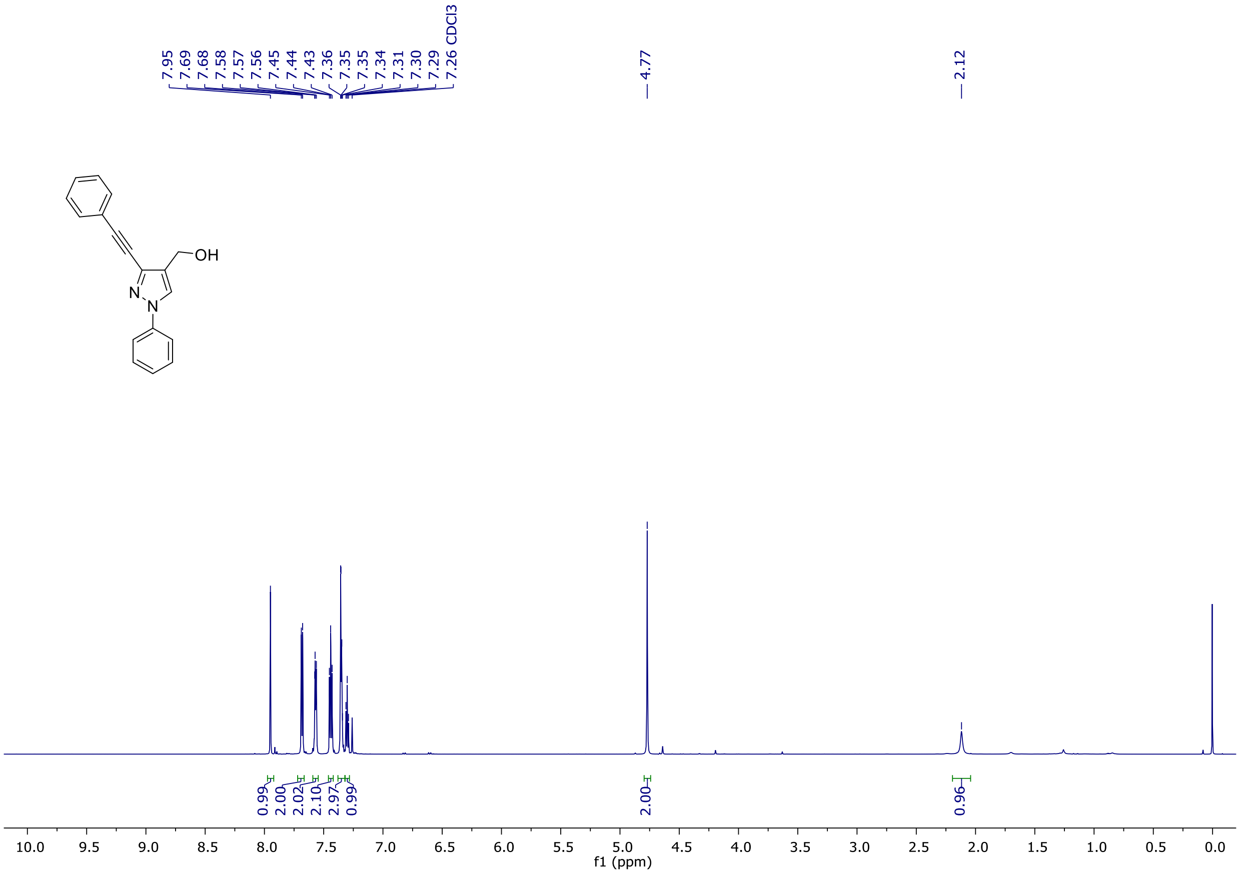

Figure S2. [1-Phenyl-3-(phenylethynyl)-1*H*-pyrazol-4-yl]methanol (**3**). ^{1}H NMR spectrum (700 MHz, $CDCl_3$).

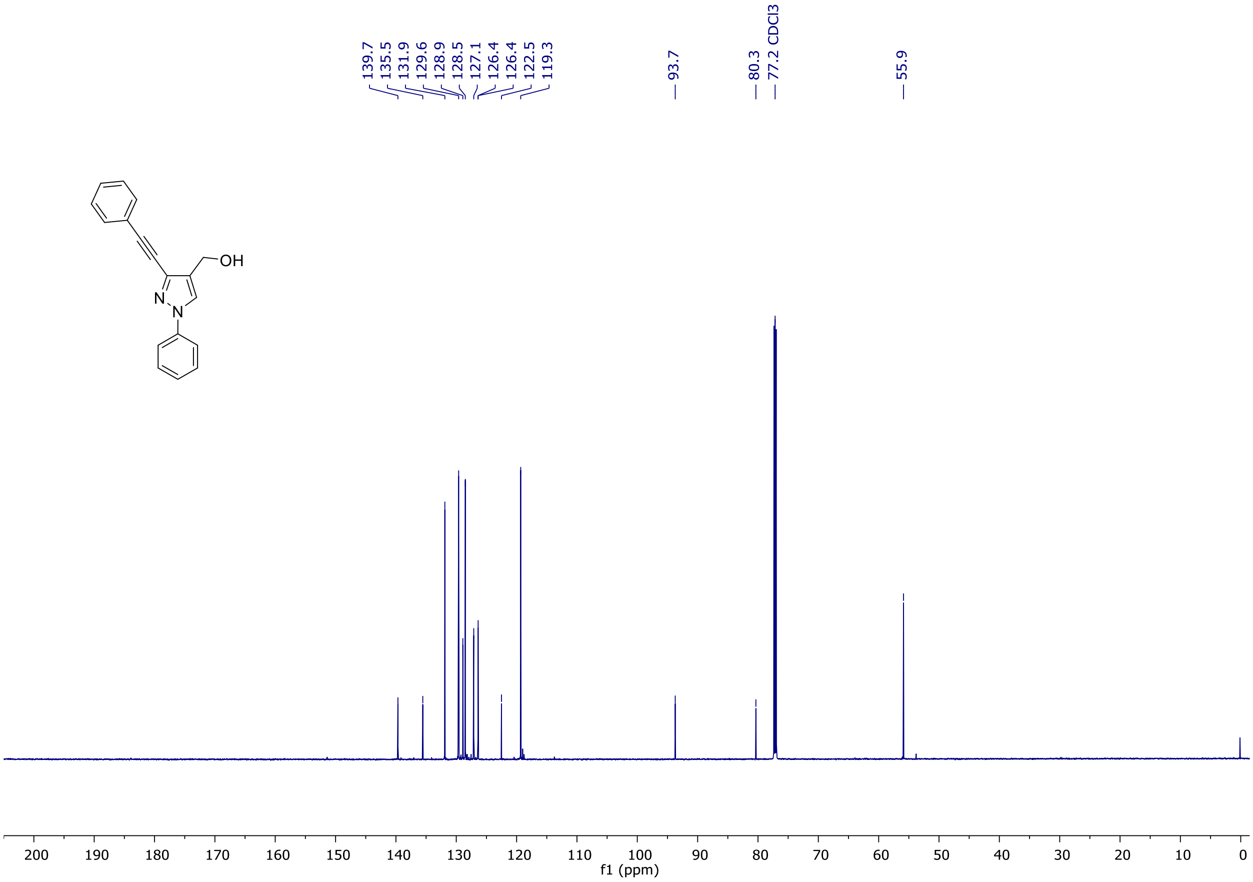

Figure S3. [1-Phenyl-3-(phenylethynyl)-1*H*-pyrazol-4-yl]methanol (**3**). ^{13}C NMR (176 MHz, $CDCl_3$).

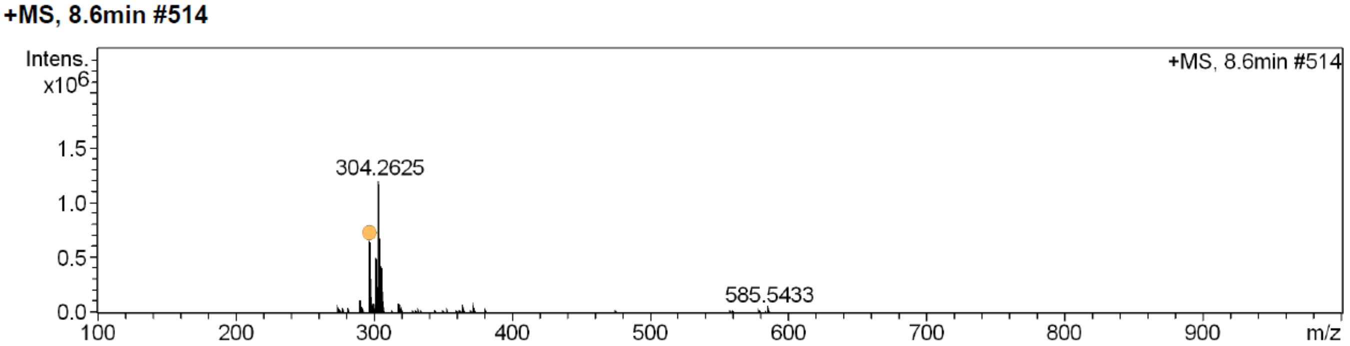

Meas. m/z	#	Ion Formula	m/z	err [ppm]	mSigma	# Sigma	Score	rdb	e⁻ Conf	N-Rule
297.0998	1	C18H14N2NaO	297.0998	0.2	8.9	1	100.00	12.5	even	ok

Figure S4. [1-Phenyl-3-(phenylethynyl)-1*H*-pyrazol-4-yl]methanol (**3**). HRMS (ESI-TOF).

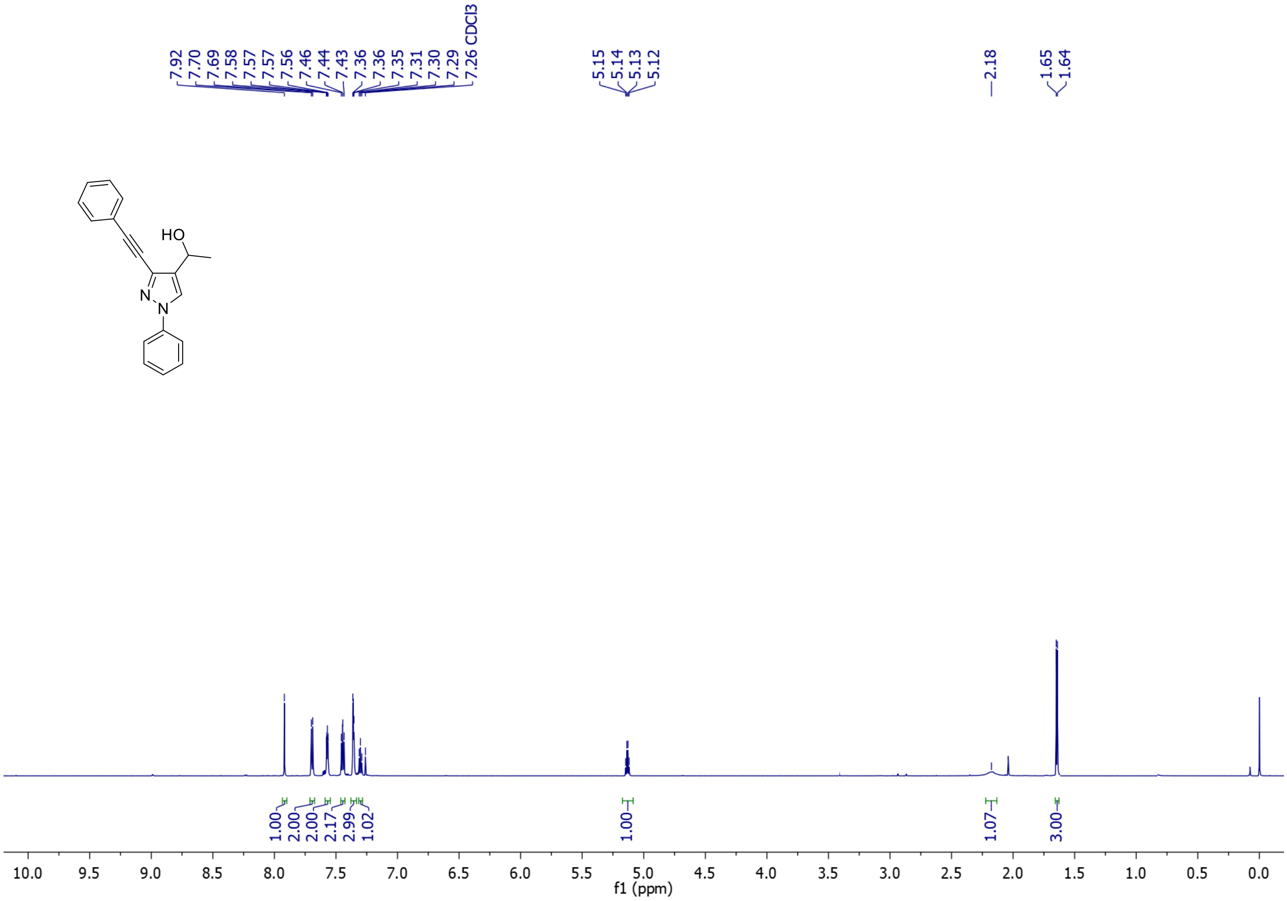

Figure S5. 1-[1-Phenyl-3-(phenylethynyl)-1*H*-pyrazol-4-yl]ethanol-1-ol (**4**). ^{1}H NMR spectrum (700 MHz, $CDCl_3$).

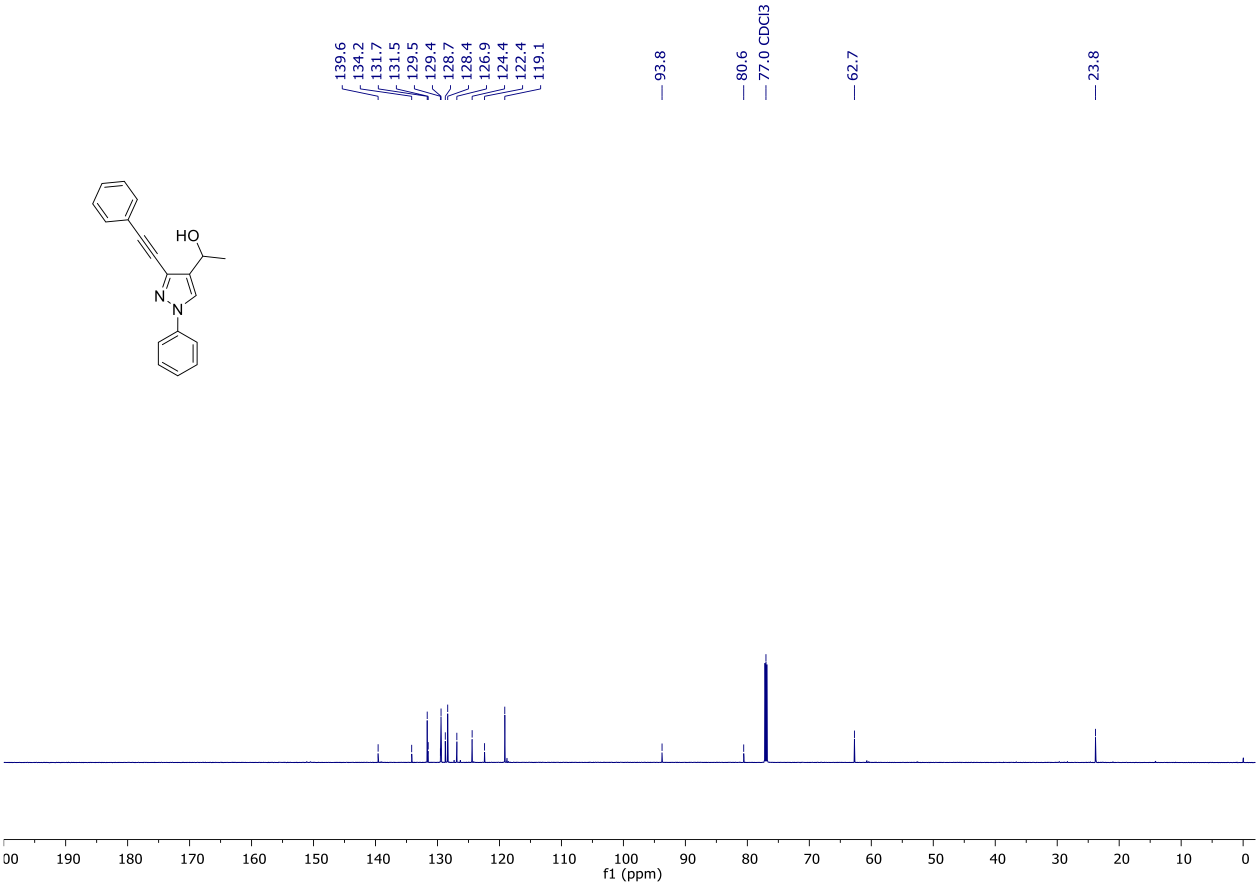

Figure S6. 1-[1-Phenyl-3-(phenylethynyl)-1*H*-pyrazol-4-yl]ethanol-1-ol (**4**). ^{13}C NMR (176 MHz, $CDCl_3$).

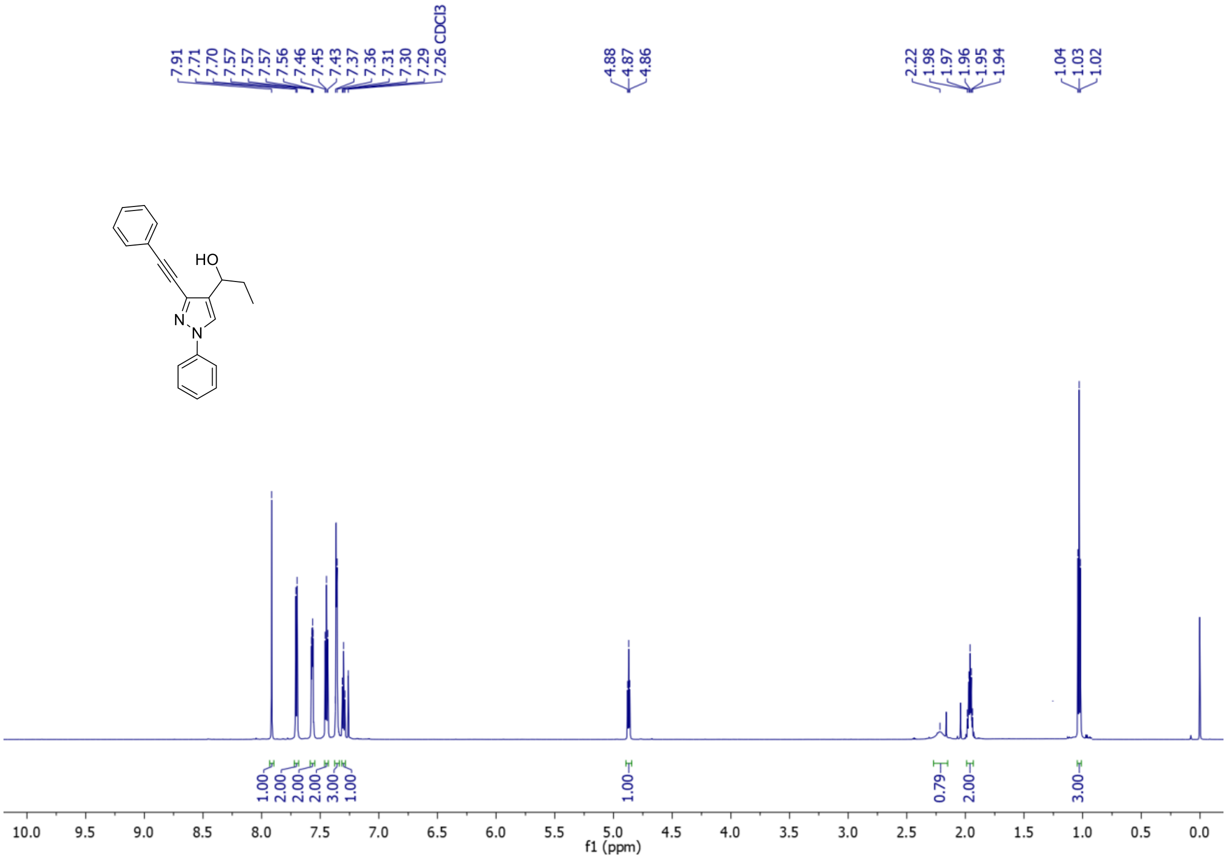

Figure S7. 1-(1-phenyl-3-(phenylethynyl)-1*H*-pyrazol-4-yl)propan-1-ol (**5**). ^{1}H NMR spectrum (700 MHz, $CDCl_3$).

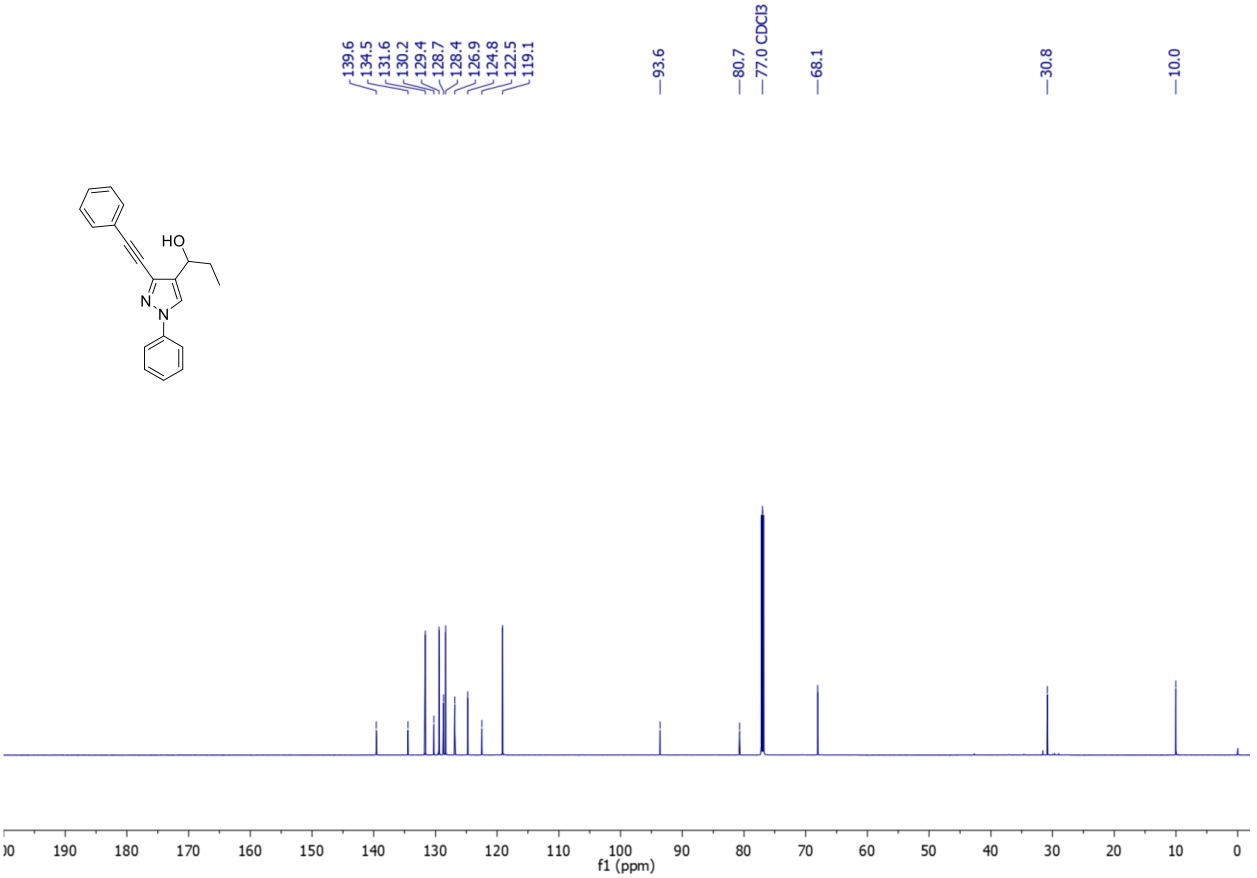

Figure S8. 1-(1-phenyl-3-(phenylethynyl)-1*H*-pyrazol-4-yl)propan-1-ol (**5**). ^{13}C NMR (176 MHz, CDCl3).

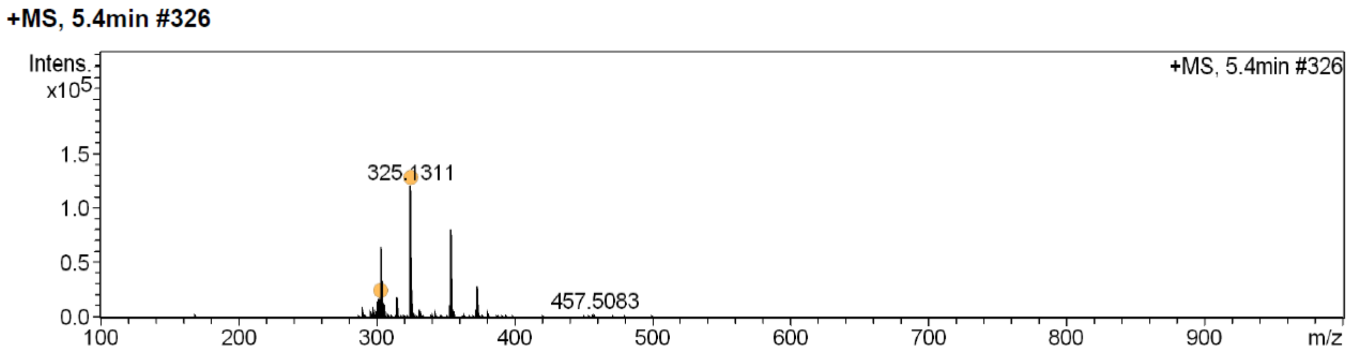

Meas. m/z	#	Ion Formula	m/z	err [ppm]	mSigma	# Sigma	Score	rdb	e⁻ Conf	N-Rule
303.1491	1	C20H19N2O	303.1492	-0.2	12.3	1	100.00	12.5	even	ok
325.1311	1	C20H18N2NaO	325.1311	-0.2	4.5	1	100.00	12.5	even	ok

Figure S9. 1-(1-phenyl-3-(phenylethynyl)-1*H*-pyrazol-4-yl)propan-1-ol (**5**). HRMS (ESI-TOF).

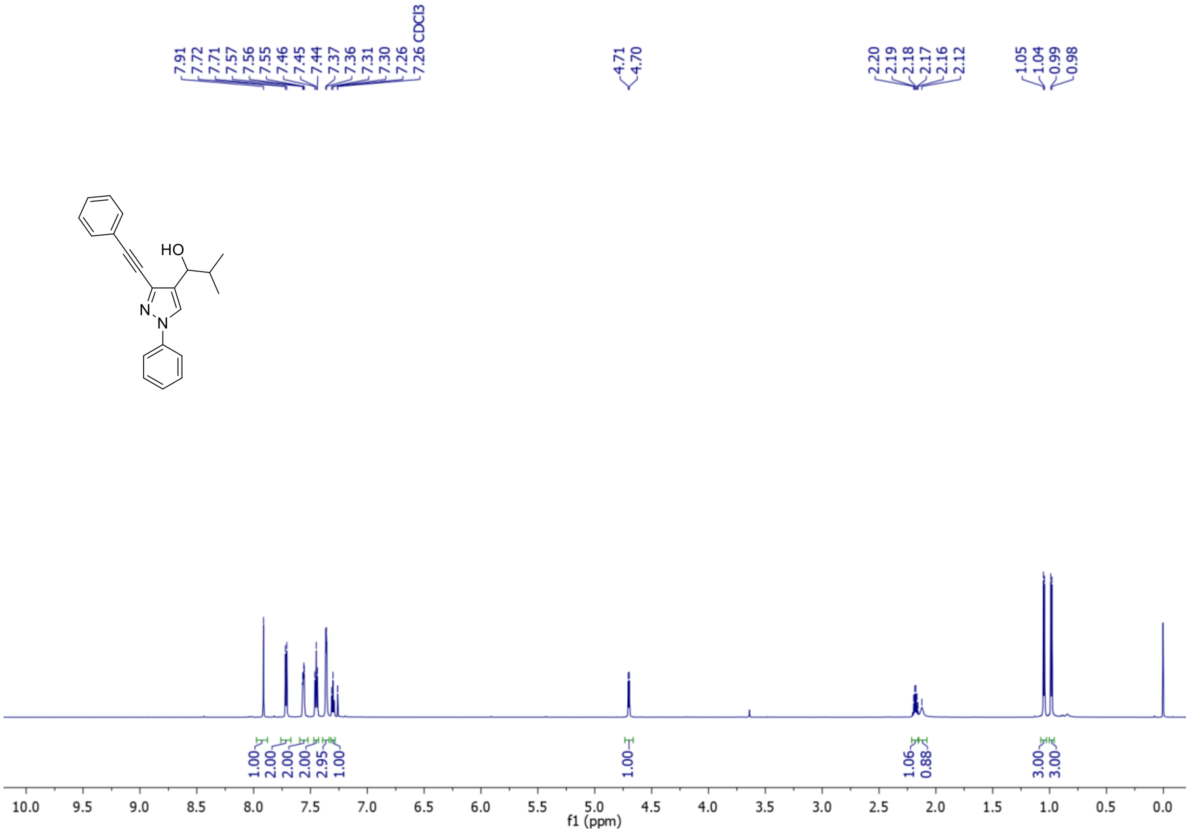

Figure S10. 2-Methyl-1-[1-phenyl-3-(phenylethynyl)-1*H*-pyrazol-4-yl]propan-1-ol (**6**). ^{1}H NMR spectrum (700 MHz, $CDCl_3$).

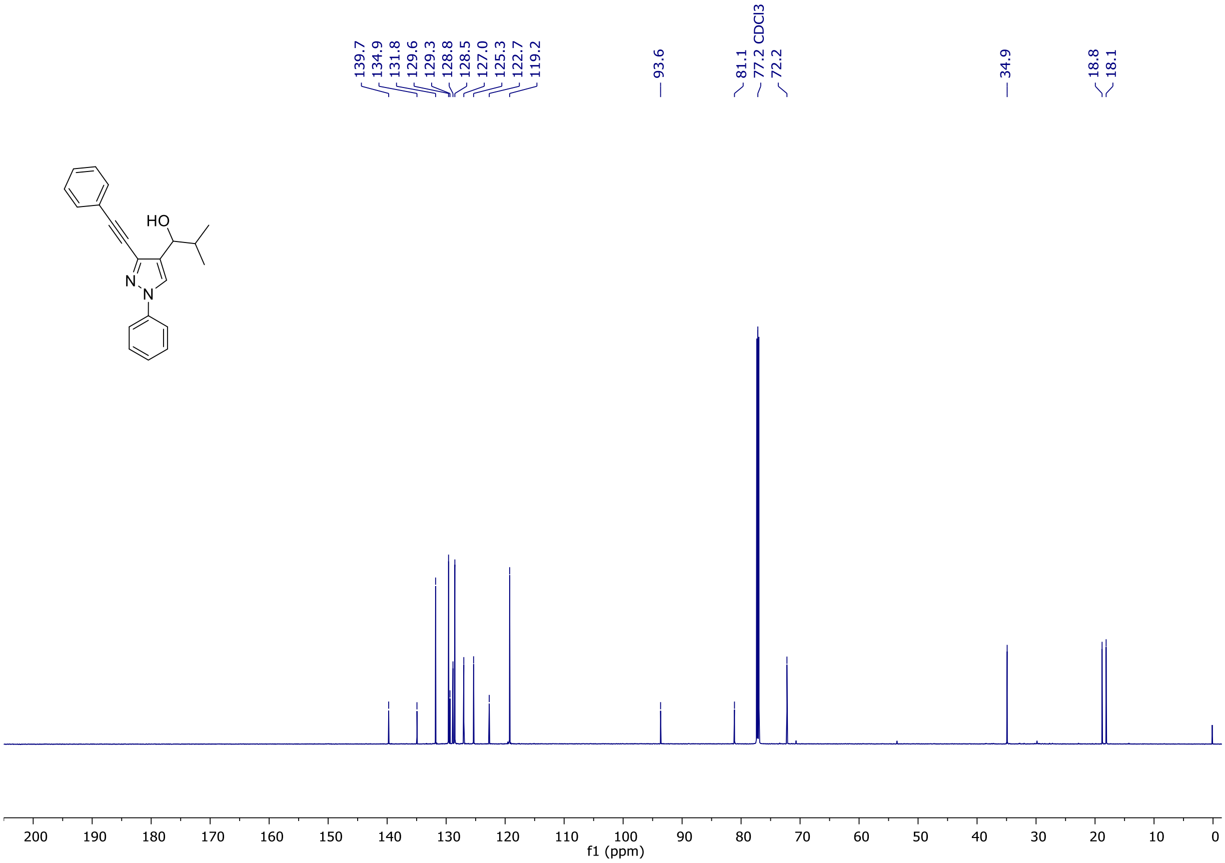

Figure S11. 2-Methyl-1-[1-phenyl-3-(phenylethynyl)-1*H*-pyrazol-4-yl]propan-1-ol (**6**). ^{13}C NMR (176 MHz, $CDCl_3$).

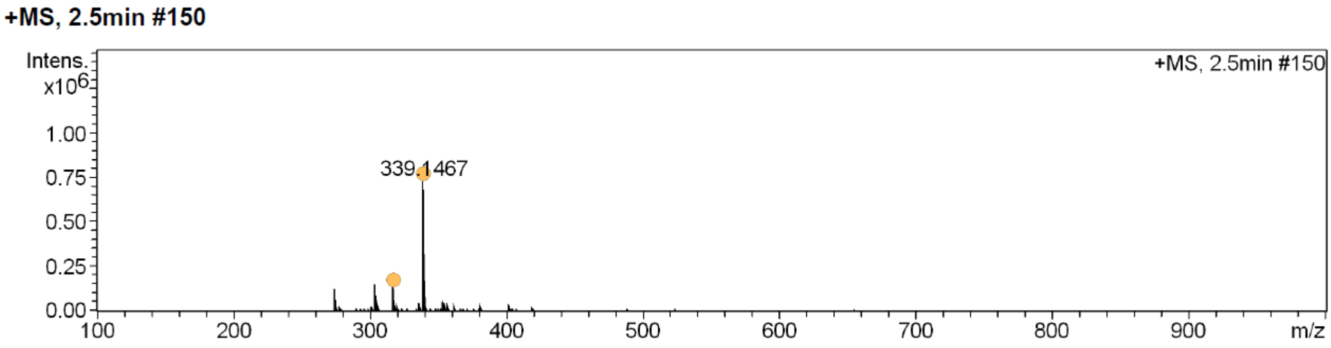

Meas. m/z	#	Ion Formula	m/z	err [ppm]	mSigma	# Sigma	Score	rdb	e⁻ Conf	N-Rule
317.1638	1	C21H21N2O	317.1648	3.3	10.8	1	100.00	12.5	even	ok
339.1467	1	C21H20N2NaO	339.1468	-0.1	9.9	1	100.00	12.5	even	ok

Figure S12. 2-Methyl-1-[1-phenyl-3-(phenylethynyl)-1*H*-pyrazol-4-yl]propan-1-ol (**6**). HRMS (ESI-TOF).

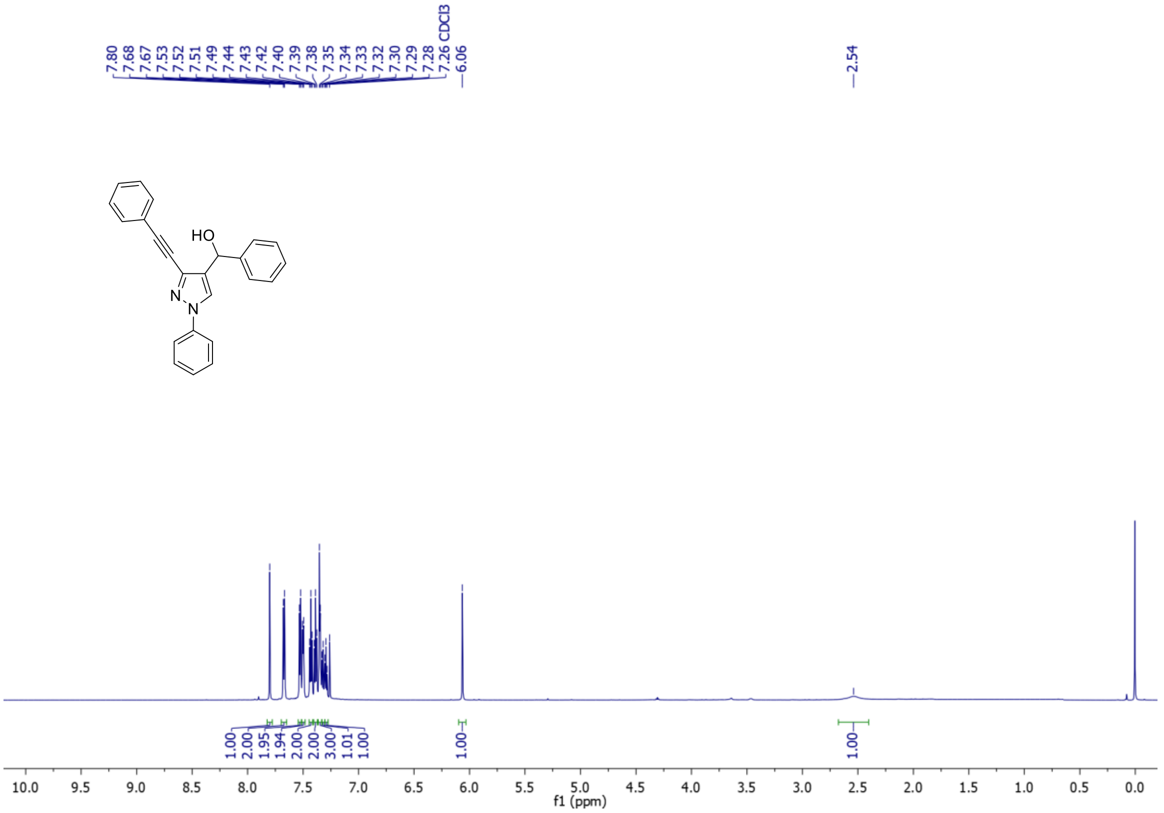

Figure S13. Phenyl(1-phenyl-3-(phenylethynyl)-1*H*-pyrazol-4-yl)methanol (**7**). ^{1}H NMR spectrum (700 MHz, $CDCl_3$).

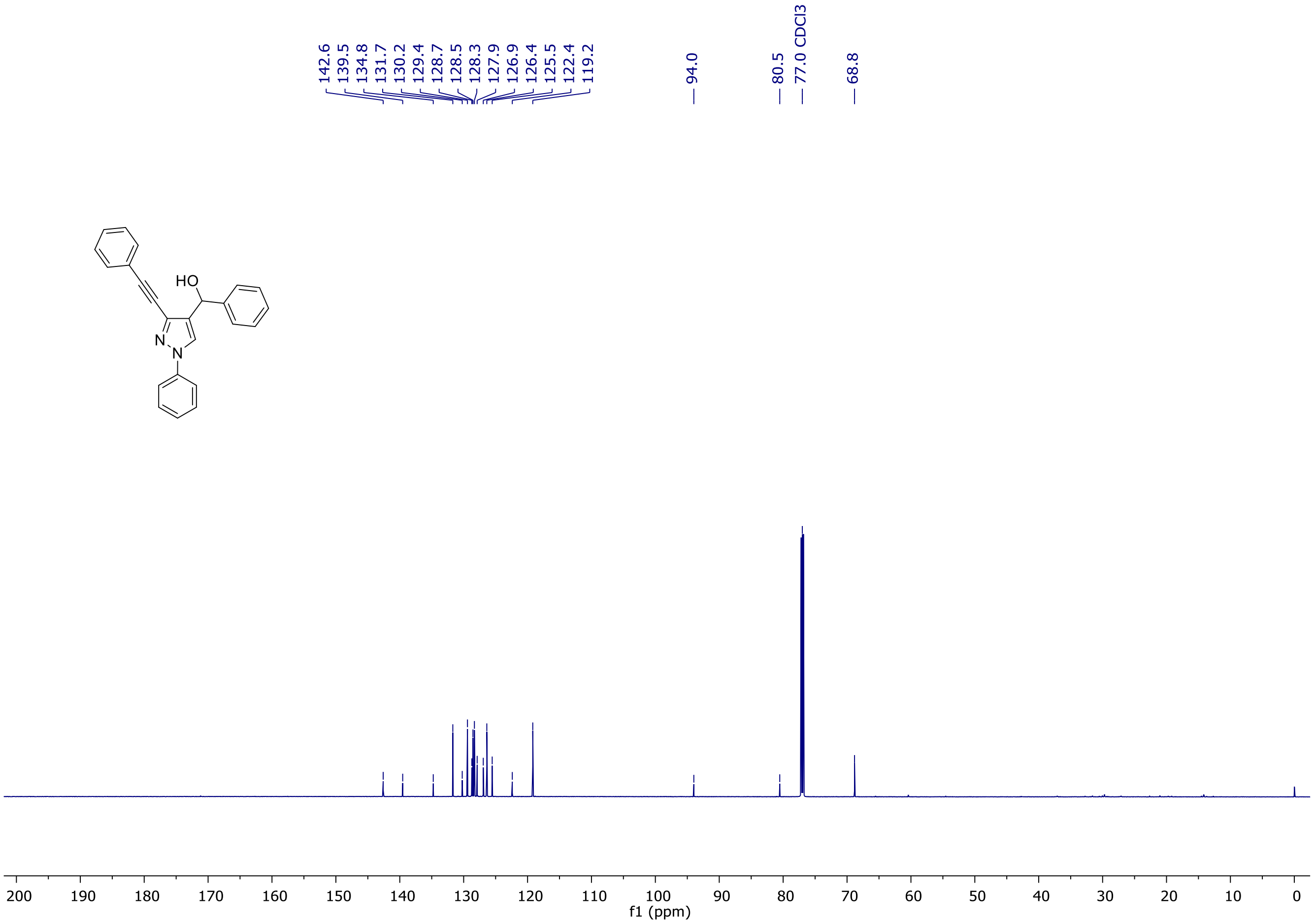

Figure S14. Phenyl(1-phenyl-3-(phenylethynyl)-1*H*-pyrazol-4-yl)methanol (**7**). ^{13}C NMR (176 MHz, $CDCl_3$).

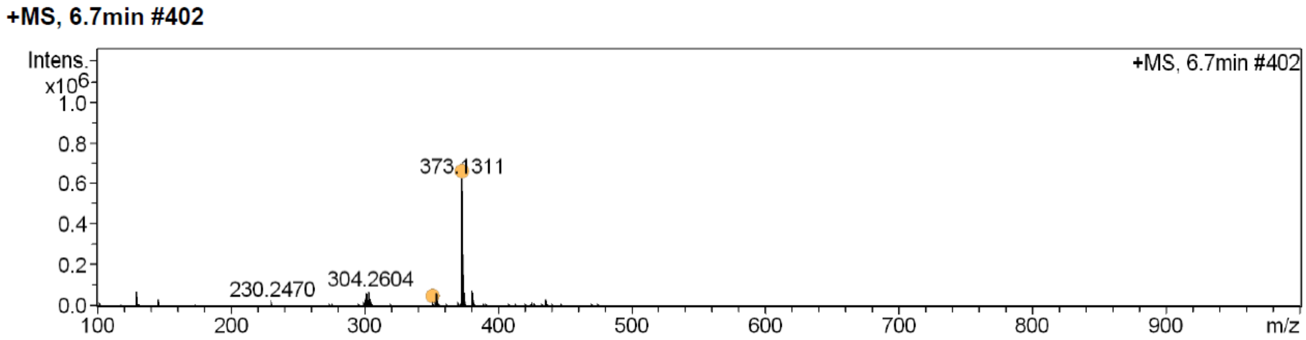

Meas. m/z	#	Ion Formula	m/z	err [ppm]	mSigma	# Sigma	Score	rdb	e⁻ Conf	N-Rule
351.1487	1	C24H19N2O	351.1492	-1.3	24.3	1	100.00	16.5	even	ok
373.1311	1	C24H18N2NaO	373.1311	0.1	3.4	1	100.00	16.5	even	ok

Figure S15. Phenyl(1-phenyl-3-(phenylethynyl)-1*H*-pyrazol-4-yl)methanol (**7**). HRMS (ESI-TOF).

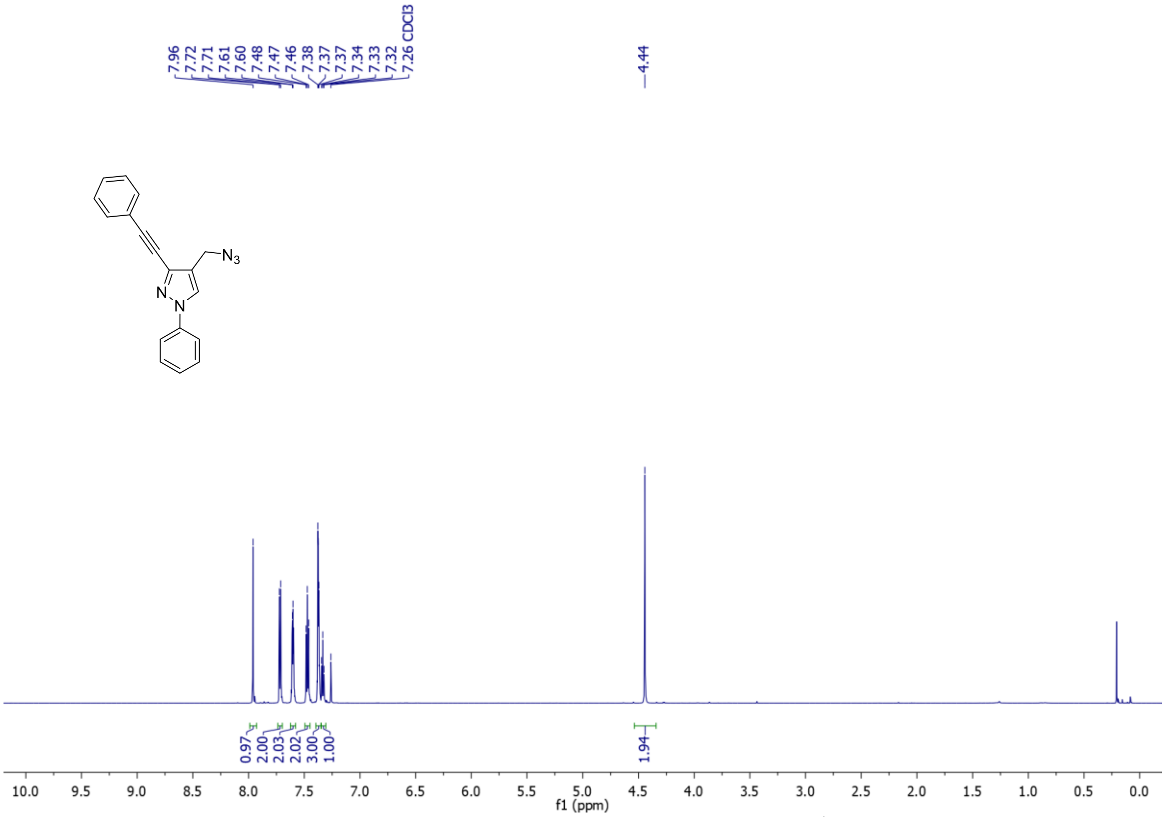

Figure S16. 4-(Azidomethyl)-1-phenyl-3-(phenylethynyl)-1*H*-pyrazole (**8**). ^{1}H NMR spectrum (700 MHz, $CDCl_3$).

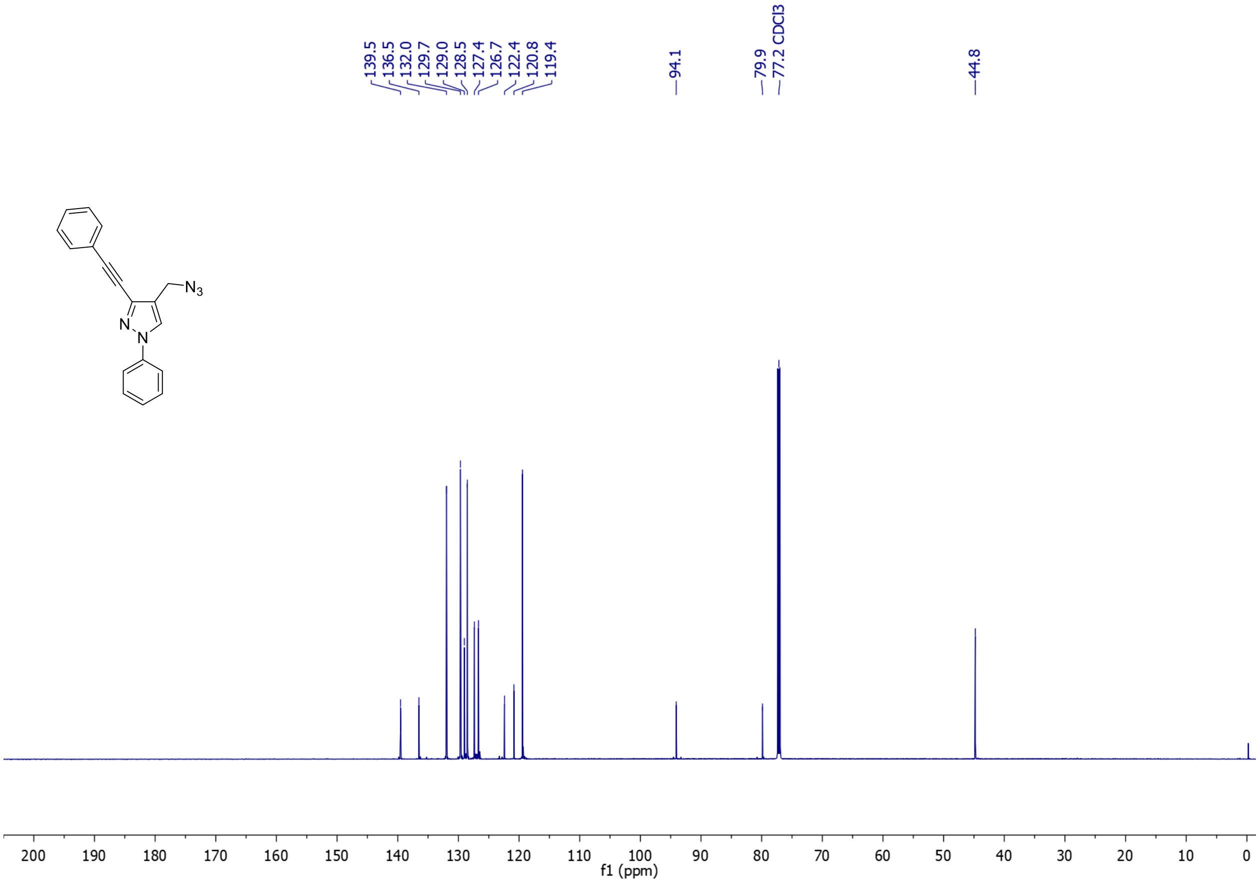

Figure S17. 4-(Azidomethyl)-1-phenyl-3-(phenylethynyl)-1*H*-pyrazole (**8**). ^{13}C NMR (176 MHz, $CDCl_3$).

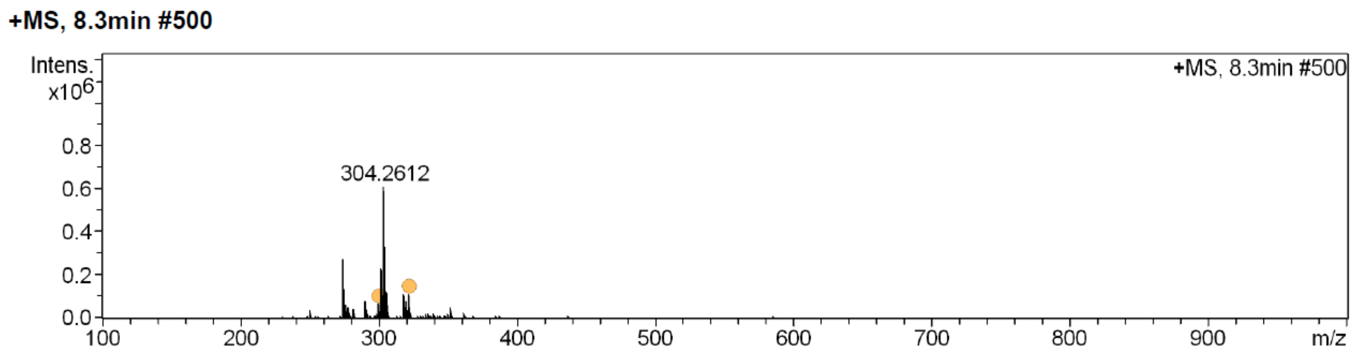

Meas. m/z	#	Ion Formula	m/z	err [ppm]	mSigma	# Sigma	Score	rdb	e⁻ Conf	N-Rule
300.1242	1	C18H14N5	300.1244	-0.6	29.1	1	100.00	14.5	even	ok
322.1065	1	C18H13N5Na	322.1063	-0.5	7.5	1	100.00	14.5	even	ok

Figure S18. 4-(Azidomethyl)-1-phenyl-3-(phenylethynyl)-1*H*-pyrazole (**8**). HRMS (ESI-TOF).

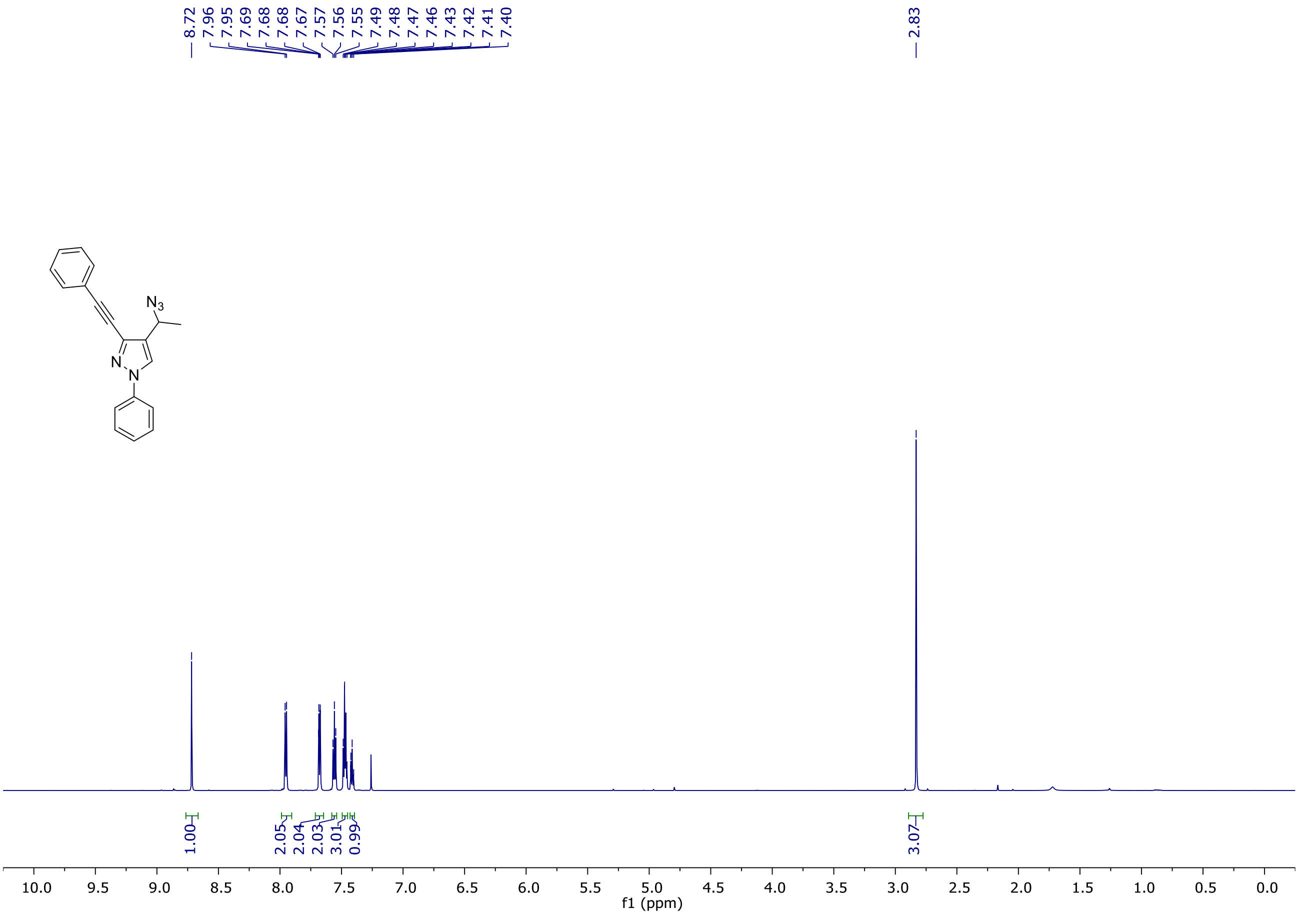

Figure S19. 4-(1-Azidoethyl)-1-phenyl-3-(phenylethynyl)-1*H*-pyrazole (**9**). ^{1}H NMR spectrum (700 MHz, $CDCl_3$).

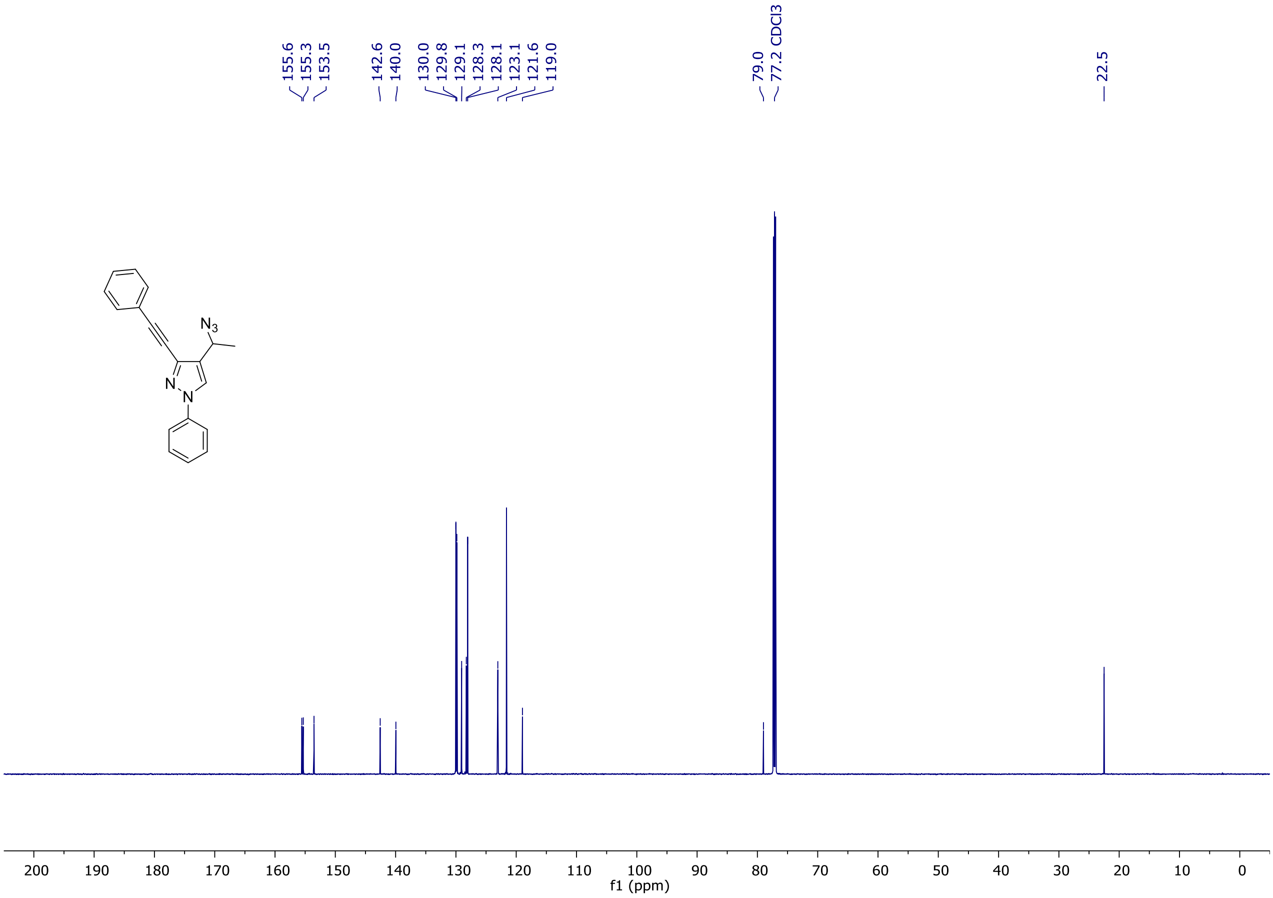

Figure S20. 4-(1-Azidoethyl)-1-phenyl-3-(phenylethynyl)-1*H*-pyrazole (**9**). ^{13}C NMR (176 MHz, $CDCl_3$).

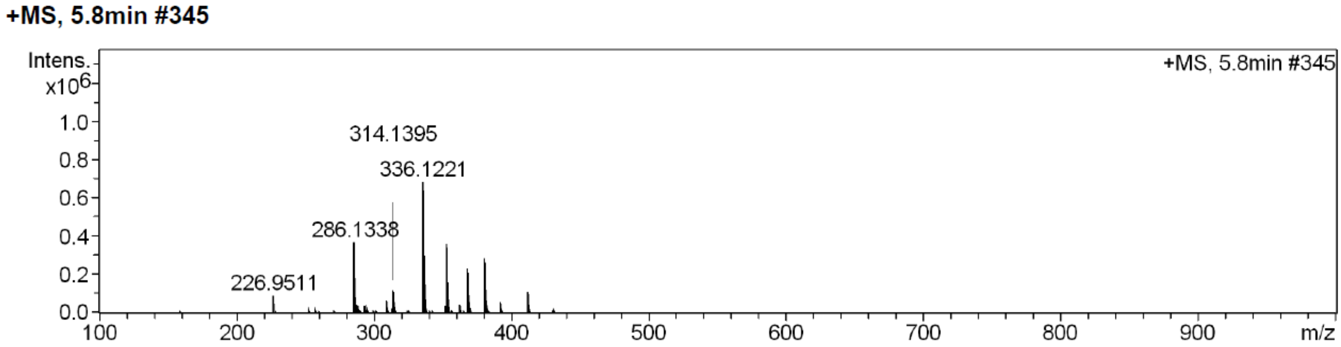

Meas. m/z	#	Ion Formula	m/z	err [ppm]	mSigma	# Sigma	Score	rdb	e⁻ Conf	N-Rule
314.1395	1	C19H16N5	314.1400	-1.6	42.0	1	100.00	14.5	even	ok
336.1221	1	C19H15N5Na	336.1220	-0.4	4.5	1	100.00	14.5	even	ok

Figure S21. 4-(1-Azidoethyl)-1-phenyl-3-(phenylethynyl)-1*H*-pyrazole (**9**). HRMS (ESI-TOF).

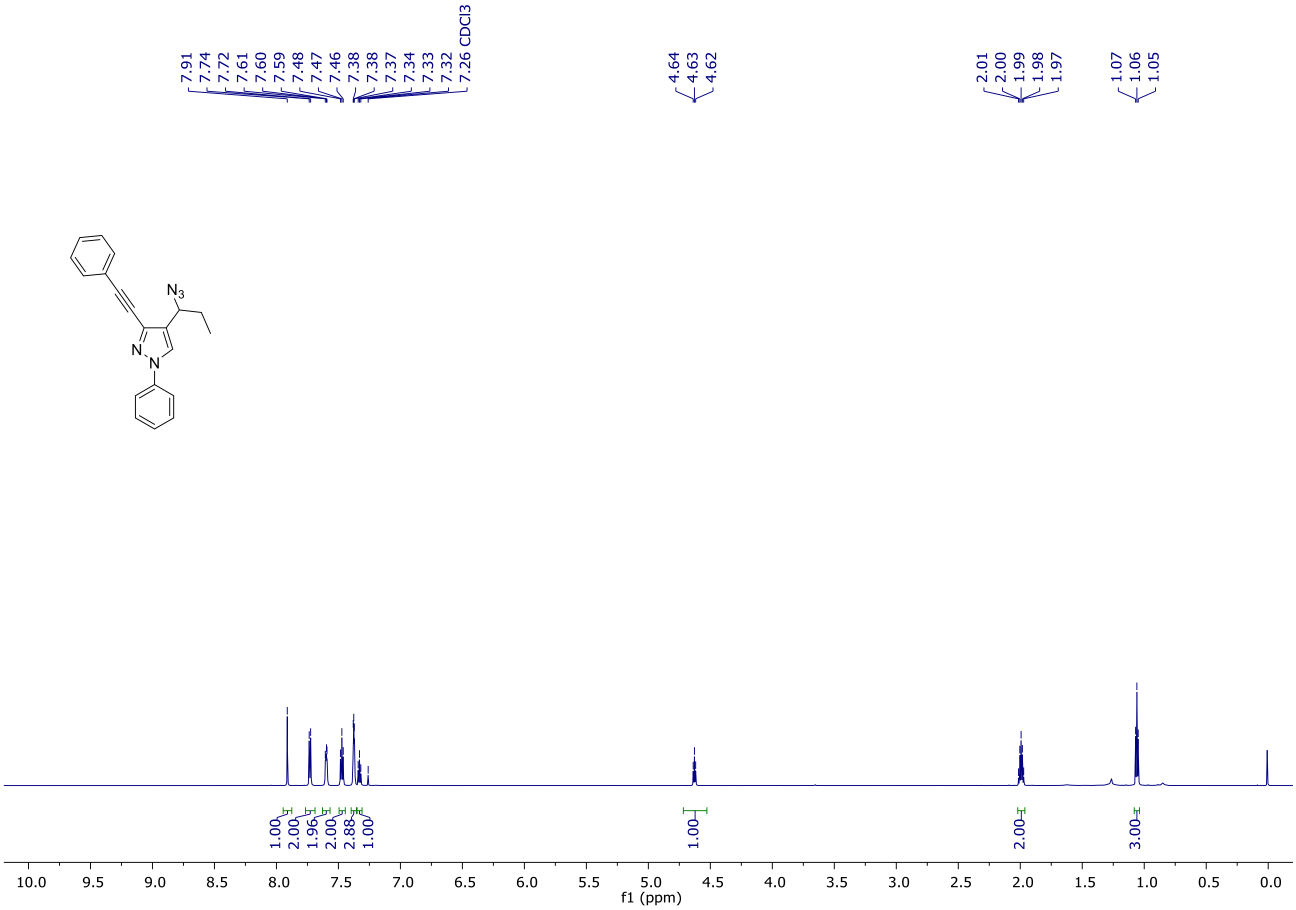

Figure S22. 4-(1-Azidopropyl)-1-phenyl-3-(phenylethynyl)-1*H*-pyrazole (**10**). ^{1}H NMR spectrum (700 MHz, $CDCl_3$).

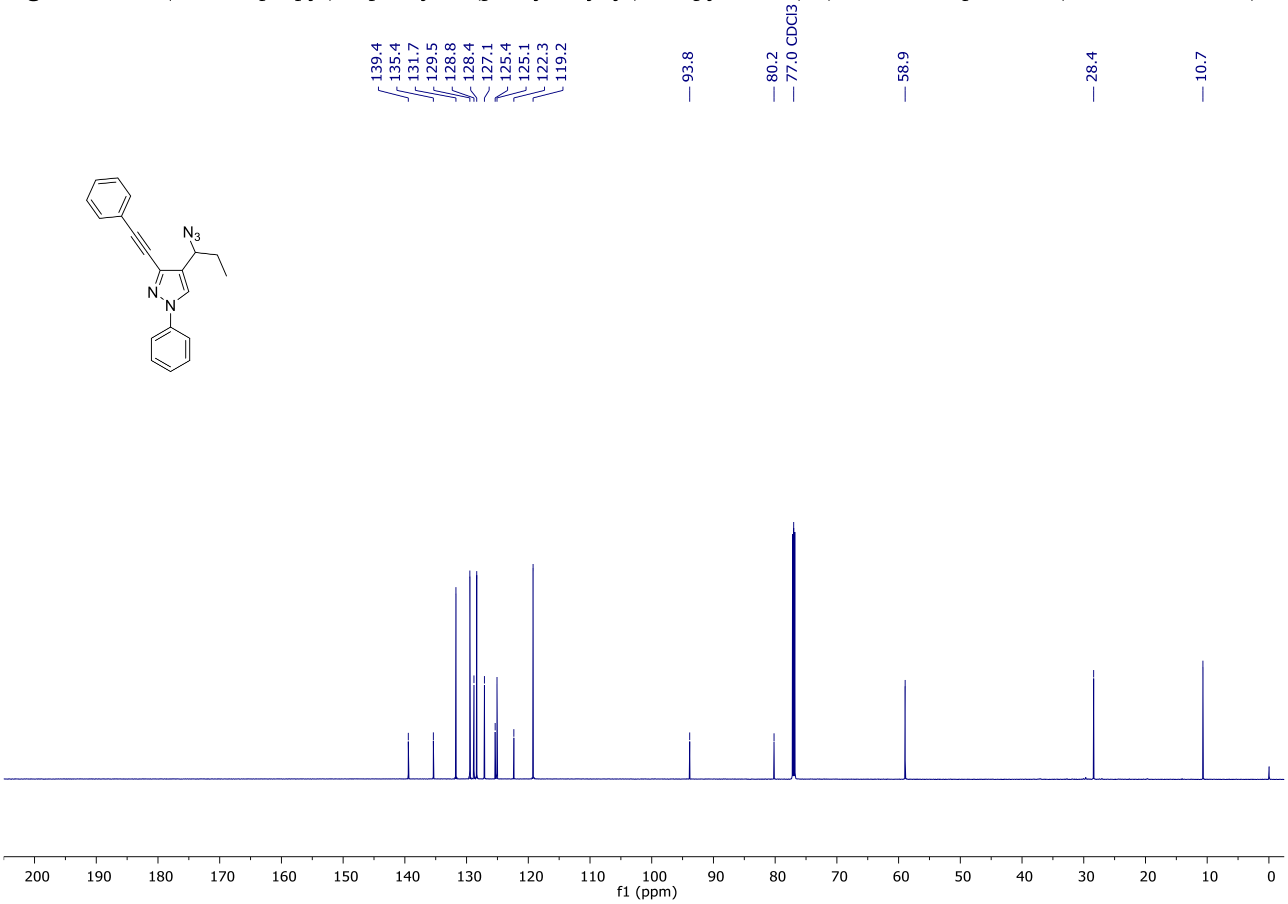

Figure S23. 4-(1-Azidopropyl)-1-phenyl-3-(phenylethynyl)-1*H*-pyrazole (**10**). ^{13}C NMR (176 MHz, $CDCl_3$).

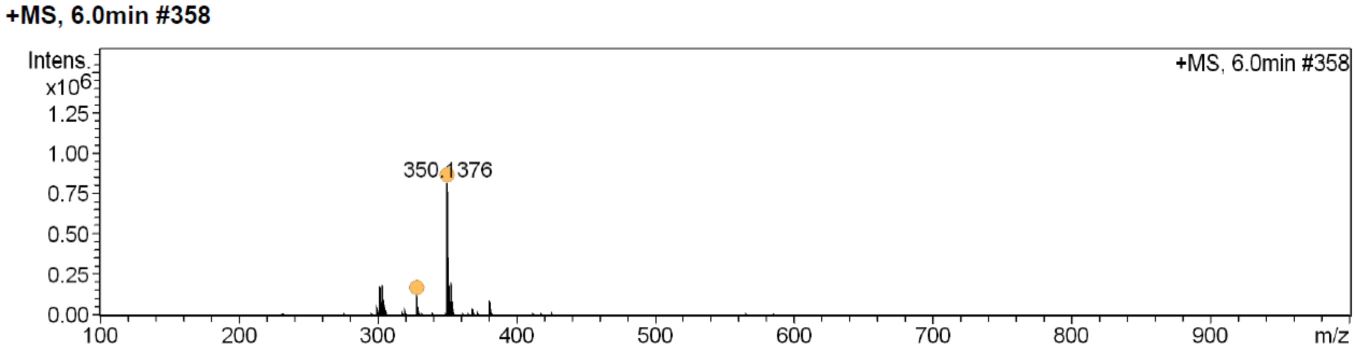

Meas. m/z	#	Ion Formula	m/z	err [ppm]	mSigma	# Sigma	Score	rdb	e¯ Conf	N-Rule
328.1553	1	C20H18N5	328.1557	-1.2	5.3	1	100.00	14.5	even	ok
350.1376	1	C20H17N5Na	350.1376	-0.2	4.6	1	100.00	14.5	even	ok

Figure S24. 2,6,7-Triphenyl-2*H*-pyrazolo[4,3-*c*]pyridine (**18**). HRMS (ESI-TOF).

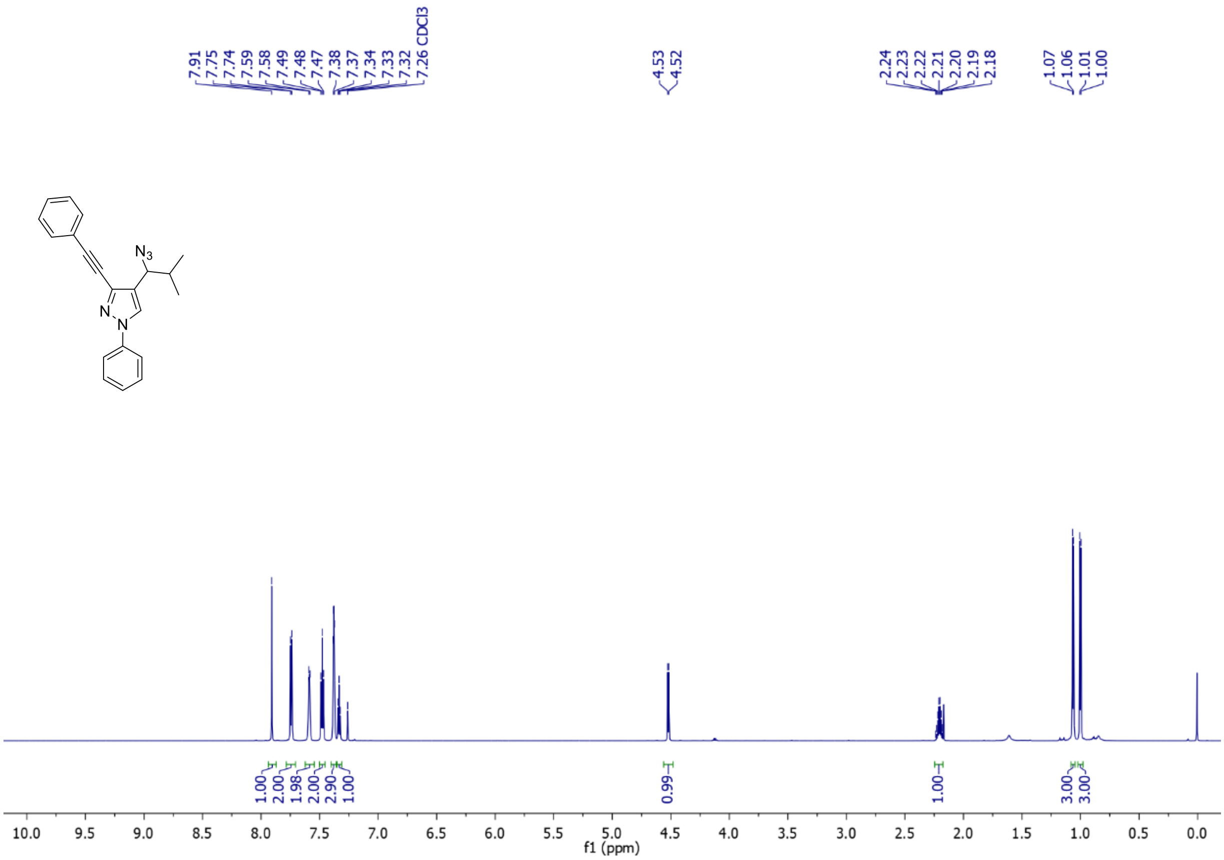

Figure S25. 4-(Azido-2-methylpropyl)-1-phenyl-3-(phenylethynyl)-1*H*-pyrazole (**11**). ^{1}H NMR spectrum (700 MHz, $CDCl_3$).

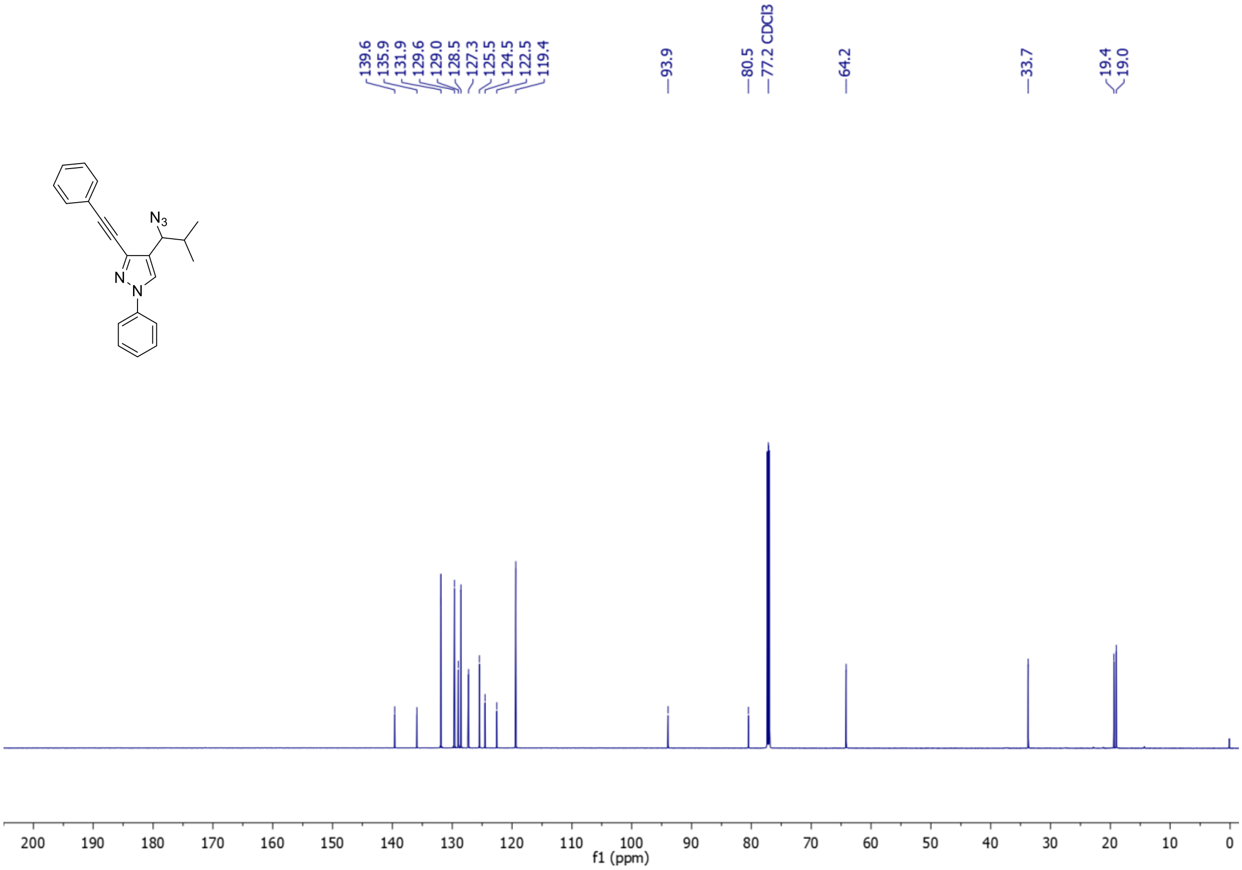

Figure S26. 4-(Azido-2-methylpropyl)-1-phenyl-3-(phenylethynyl)-1*H*-pyrazole (**11**). ^{13}C NMR (176 MHz, $CDCl_3$).

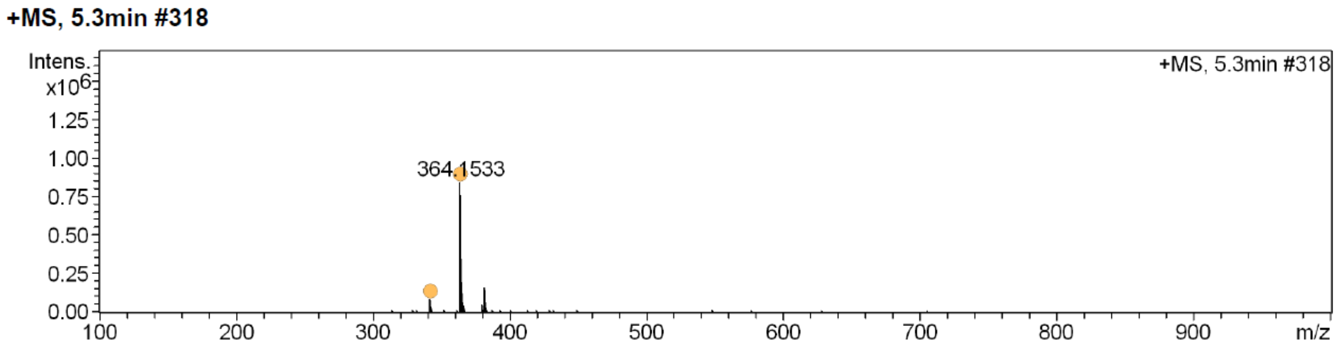

Meas. m/z	#	Ion Formula	m/z	err [ppm]	mSigma	# Sigma	Score	rdb	e⁻ Conf	N-Rule
342.1697	1	C21H20N5	342.1713	4.8	14.5	1	100.00	14.5	even	ok
364.1533	1	C21H19N5Na	364.1533	0.0	4.3	1	100.00	14.5	even	ok

Figure S27. 4-(Azido-2-methylpropyl)-1-phenyl-3-(phenylethynyl)-1*H*-pyrazole (**11**). HRMS (ESI-TOF).

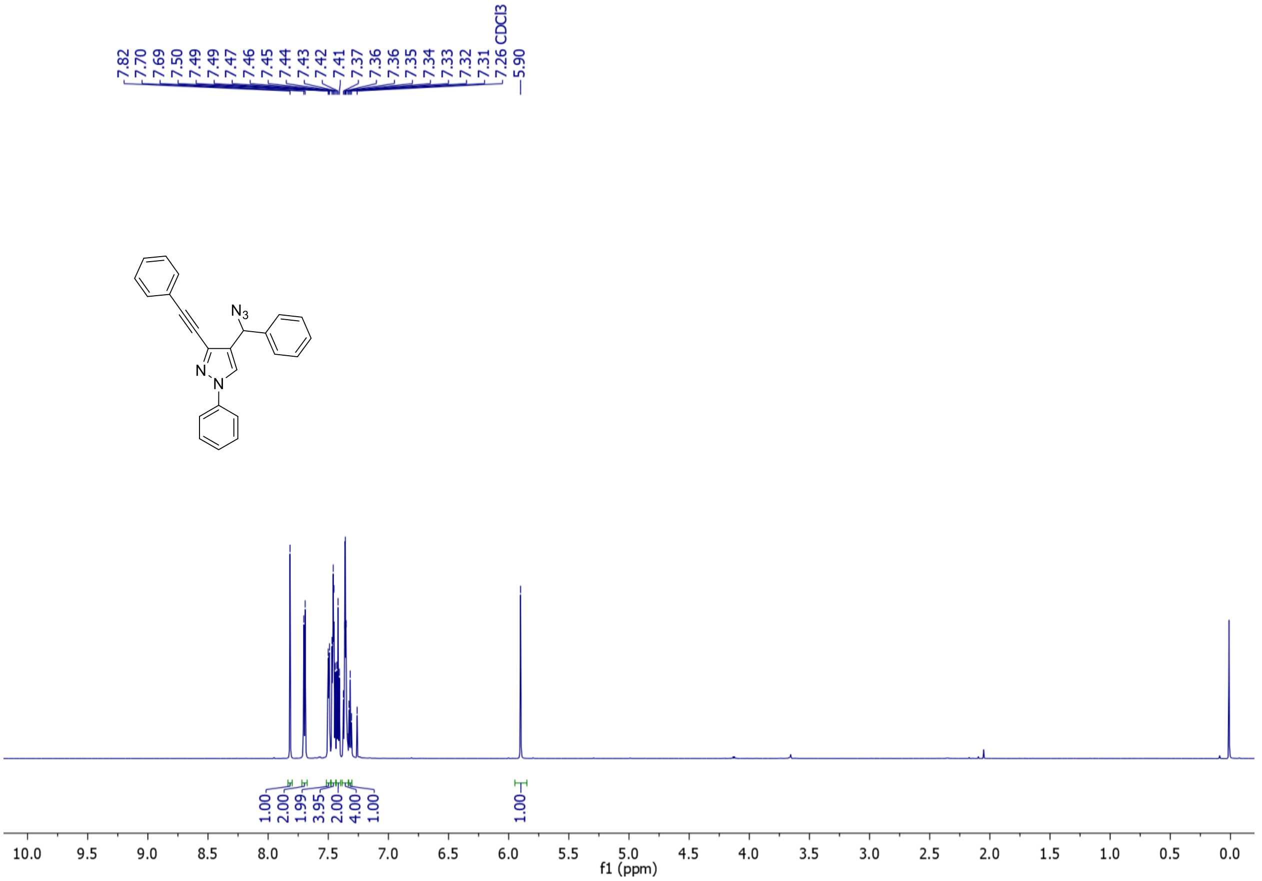

Figure S28. 4-(Azido(phenyl)methyl)-1-phenyl-3-(phenylethynyl)-1*H*-pyrazole (**12**). ^{1}H NMR spectrum (700 MHz, $CDCl_3$).

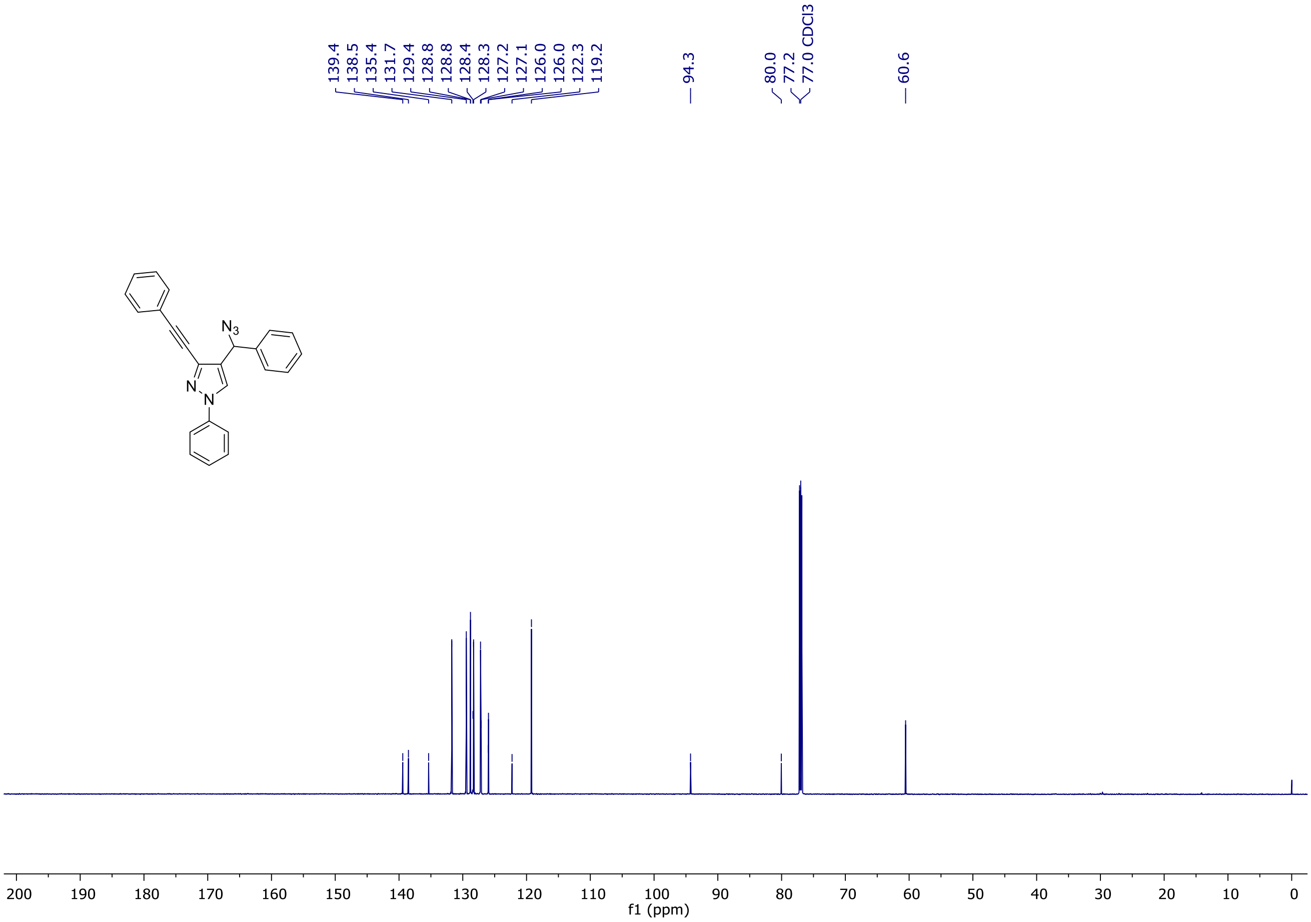

Figure S29. 4-(Azido(phenyl)methyl)-1-phenyl-3-(phenylethynyl)-1*H*-pyrazole (**12**). ^{13}C NMR (176 MHz, $CDCl_3$).

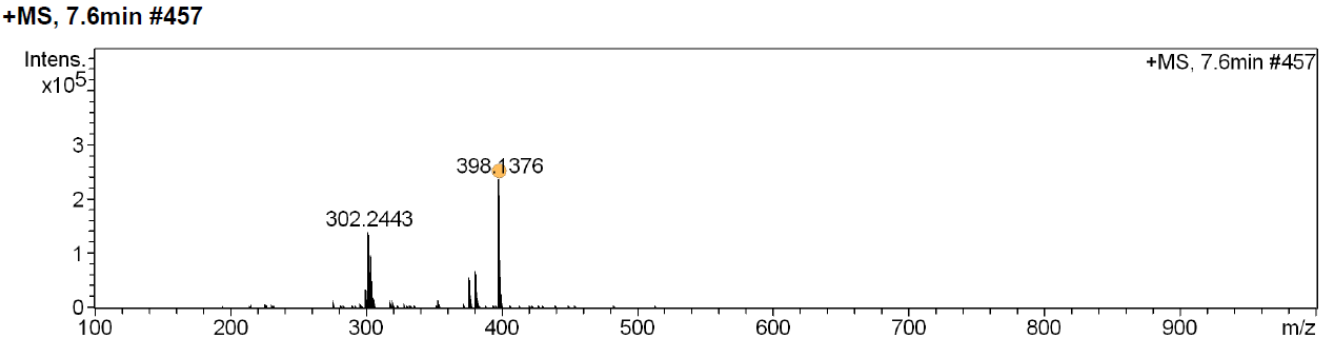

Meas. m/z	#	Ion Formula	m/z	err [ppm]	mSigma	# Sigma	Score	rdb	e¯ Conf	N-Rule
398.1376	1	C24H17N5Na	398.1376	0.1	2.0	1	100.00	18.5	even	ok

Figure S30. 4-(Azido(phenyl)methyl)-1-phenyl-3-(phenylethynyl)-1*H*-pyrazole (**12).** HRMS (ESI-TOF).

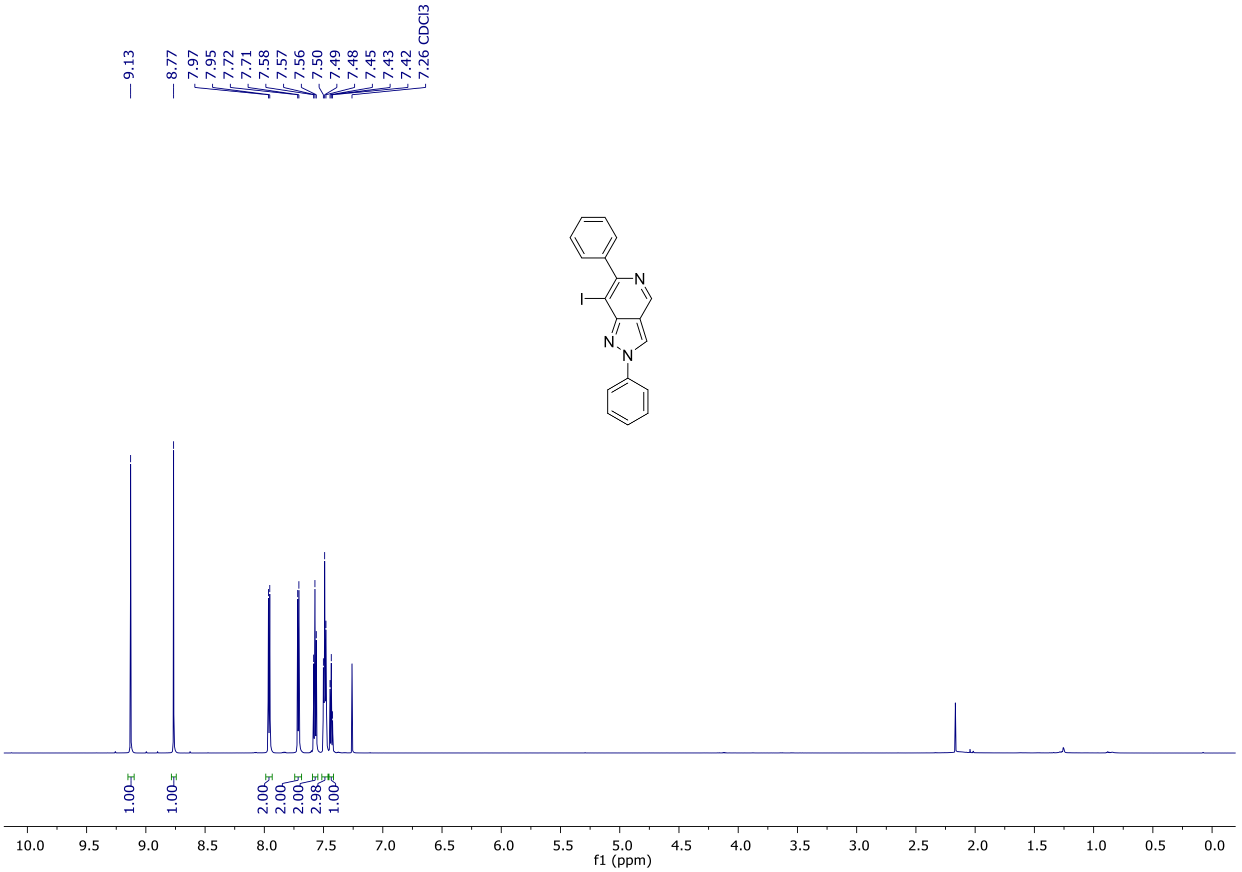

Figure S31. 7-Iodo-2,6-diphenyl-2*H*-pyrazolo[4,3-*c*]pyridine (**13**). ^{1}H NMR spectrum (700 MHz, $CDCl_3$).

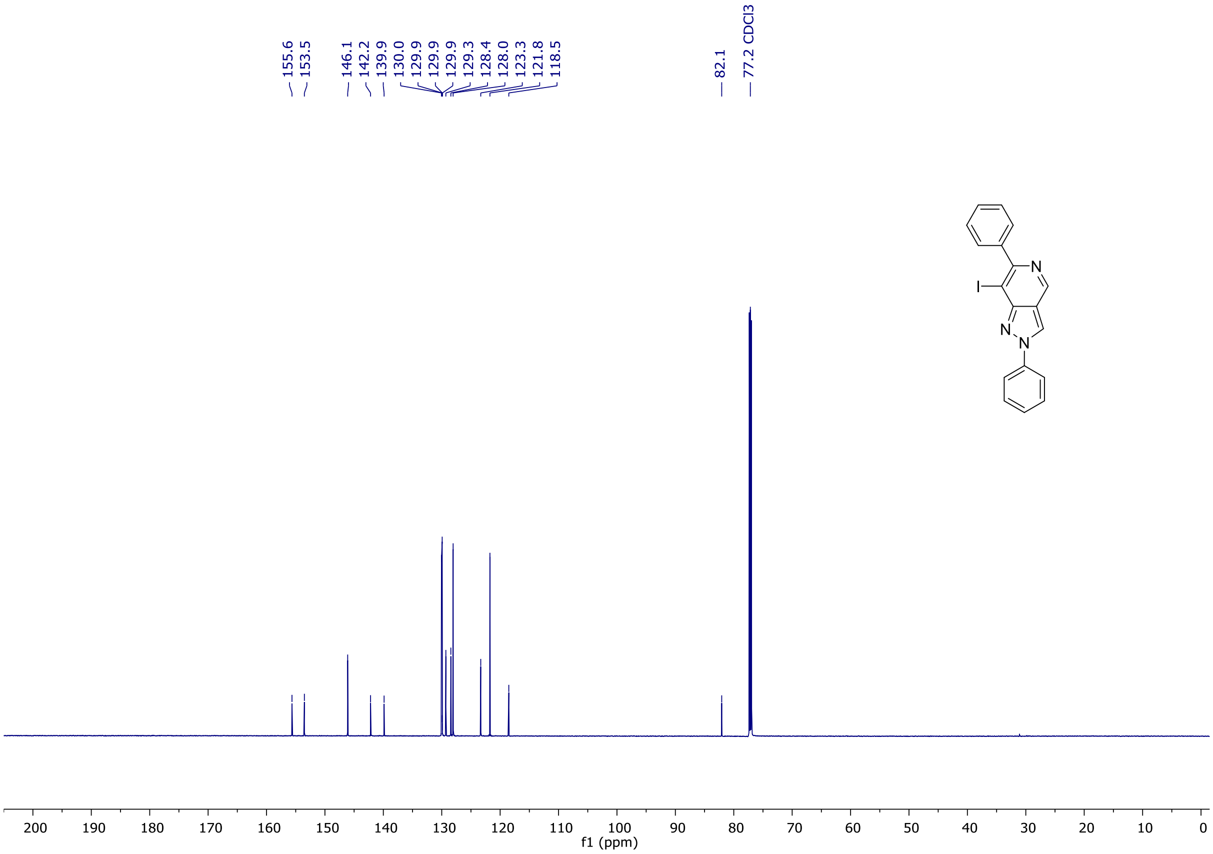

Figure S32. 7-Iodo-2,6-diphenyl-2*H*-pyrazolo[4,3-*c*]pyridine (**13**). ^{13}C NMR (176 MHz, $CDCl_3$).

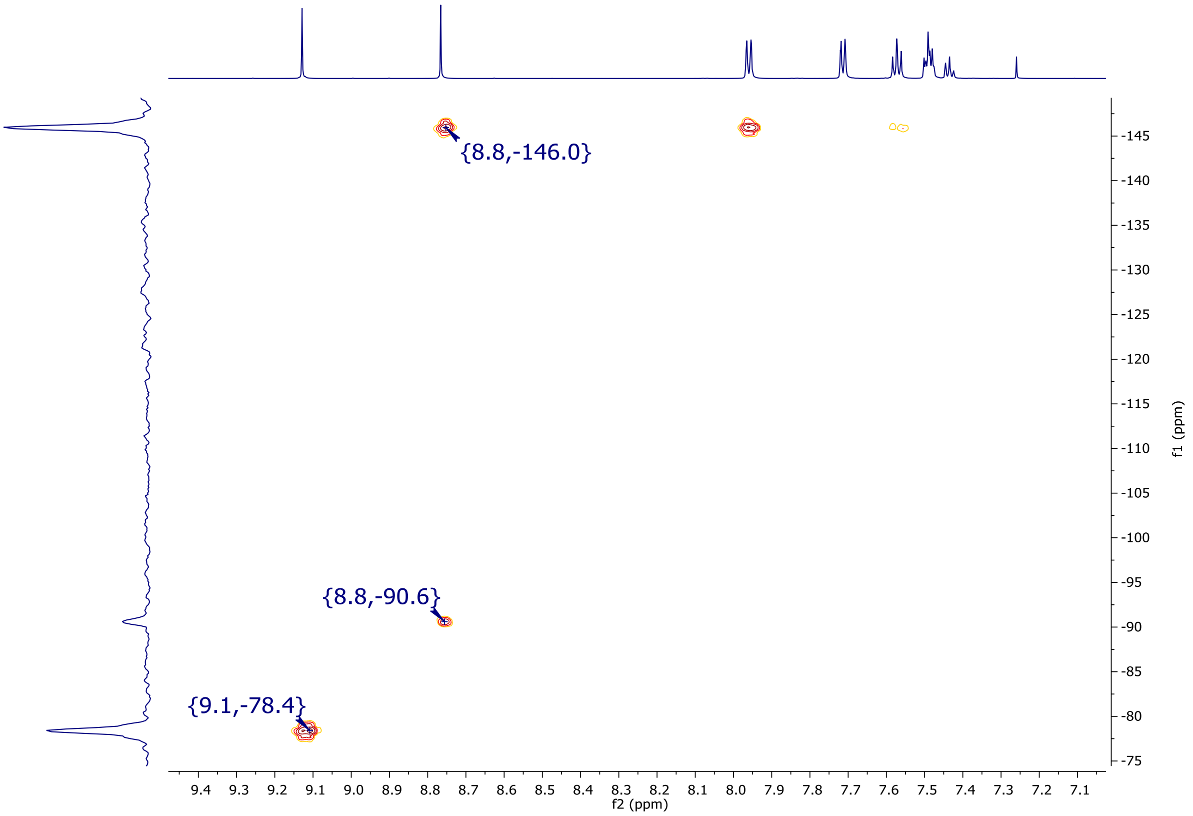

Figure S33. 7-Iodo-2,6-diphenyl-2*H*-pyrazolo[4,3-*c*]pyridine (**13**). ^{1}H-^{15}N HMBC spectrum (700 MHz, $CDCl_3$)

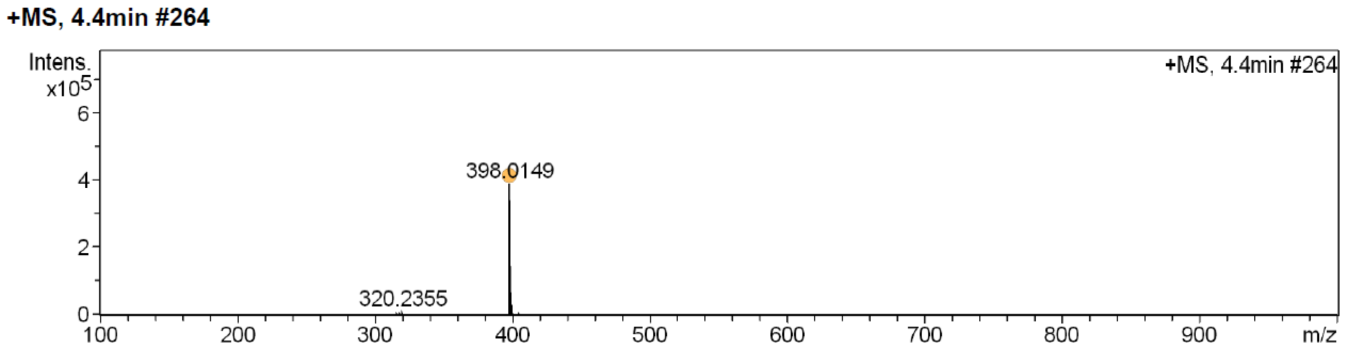

Meas. m/z	#	Ion Formula	m/z	err [ppm]	mSigma	# Sigma	Score	rdb	e⁻ Conf	N-Rule
398.0149	1	C18H13IN3	398.0149	-0.2	1.0	1	100.00	13.5	even	ok

Figure S34. 7-Iodo-2,6-diphenyl-2*H*-pyrazolo[4,3-*c*]pyridine (**13**). HRMS (ESI-TOF).

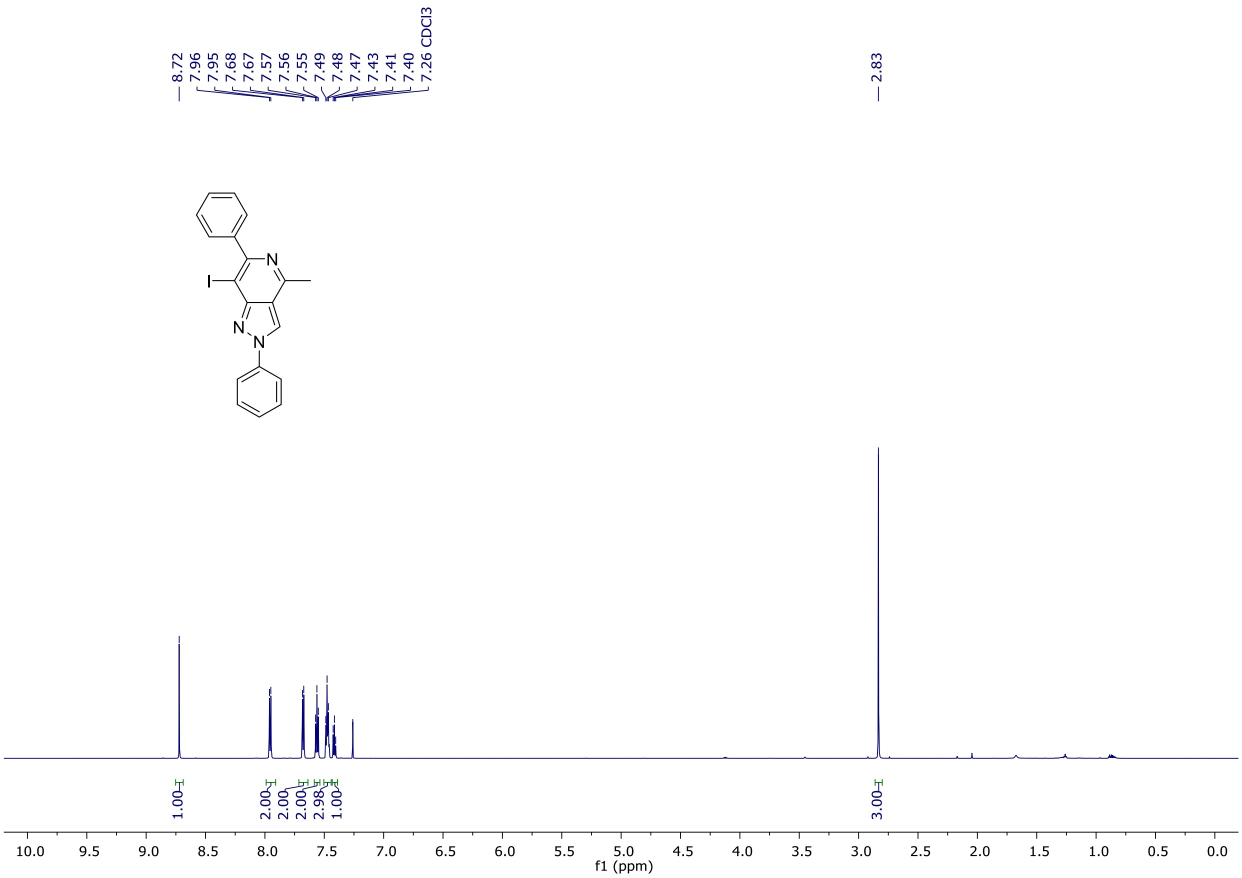

Figure S35. 7-Iodo-4-methyl-2,6-diphenyl-2*H*-pyrazolo[4,3-*c*]pyridine (**14**). ^{1}H NMR spectrum (700 MHz, $CDCl_3$).

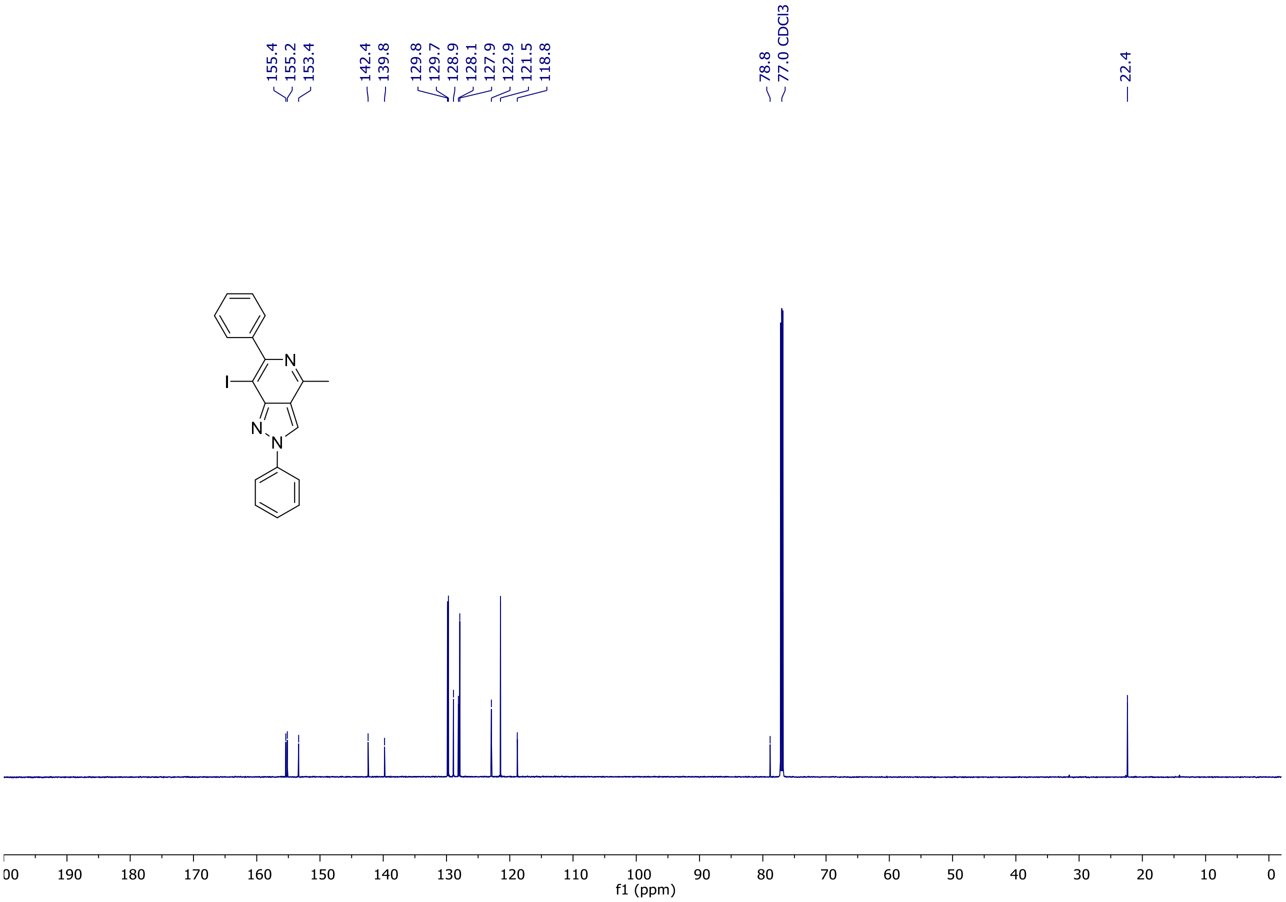

Figure S36. 7-Iodo-4-methyl-2,6-diphenyl-2*H*-pyrazolo[4,3-*c*]pyridine (**14**). ^{13}C NMR (176 MHz, $CDCl_3$).

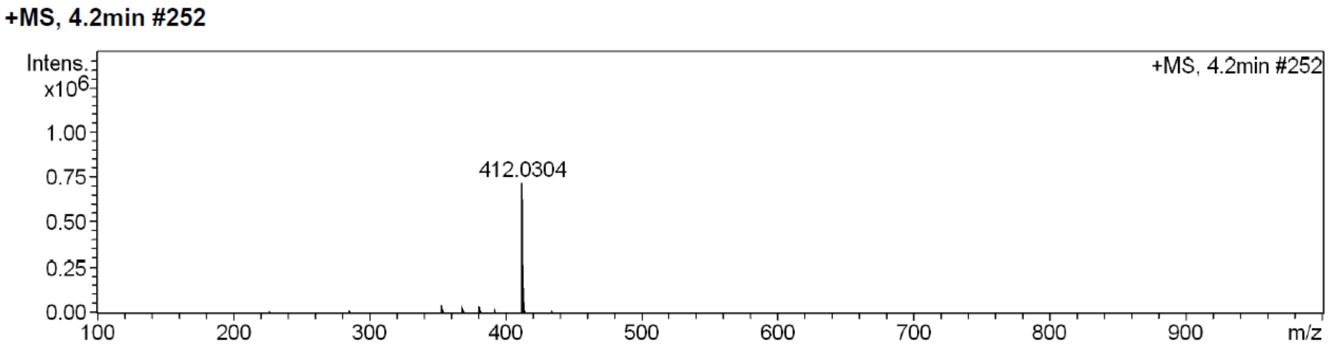

Meas. m/z	#	Ion Formula	m/z	err [ppm]	mSigma	# Sigma	Score	rdb	e⁻ Conf	N-Rule
412.0304	1	C19H15IN3	412.0305	-0.3	3.0	1	100.00	13.5	even	ok

Figure S37. 7-Iodo-4-methyl-2,6-diphenyl-2*H*-pyrazolo[4,3-*c*]pyridine (**14**). HRMS (ESI-TOF).

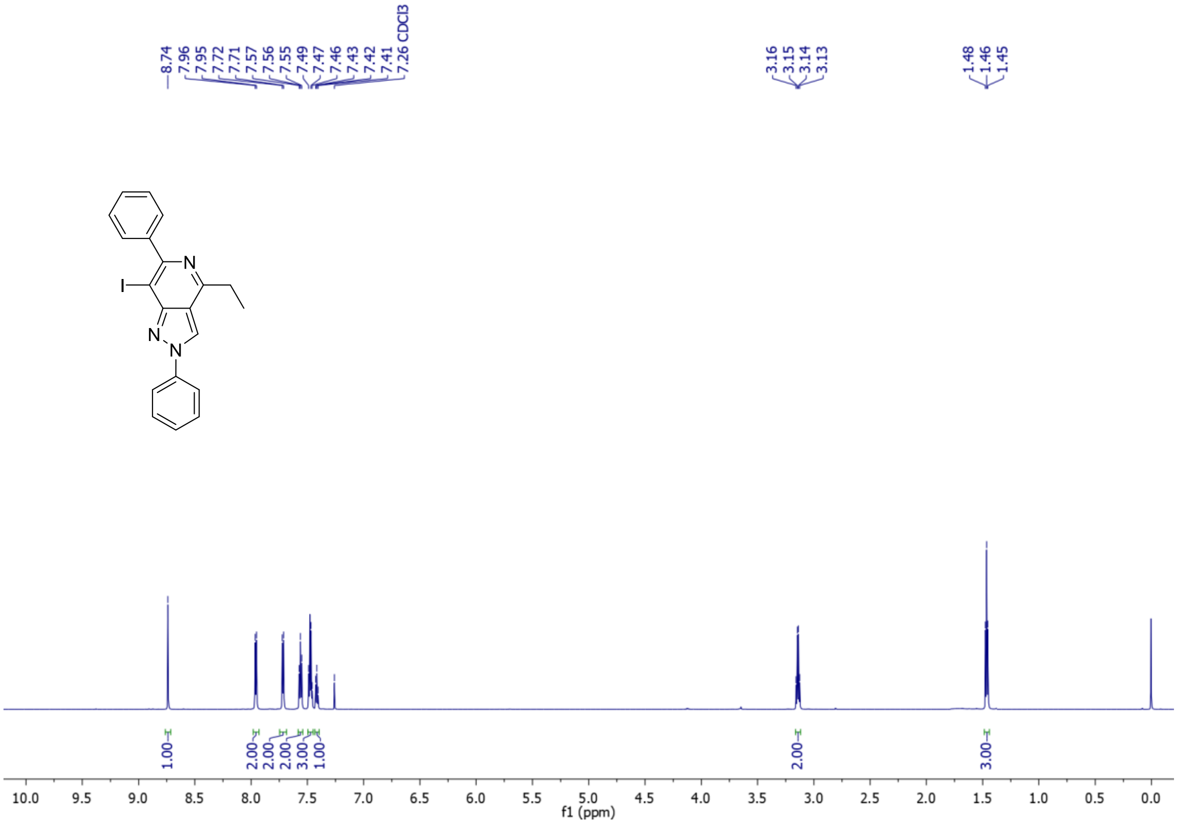

Figure S38. 4-Ethyl-7-iodo-2,6-diphenyl-2*H*-pyrazolo[4,3-*c*]pyridine (**15**). ^{1}H NMR spectrum (700 MHz, $CDCl_3$).

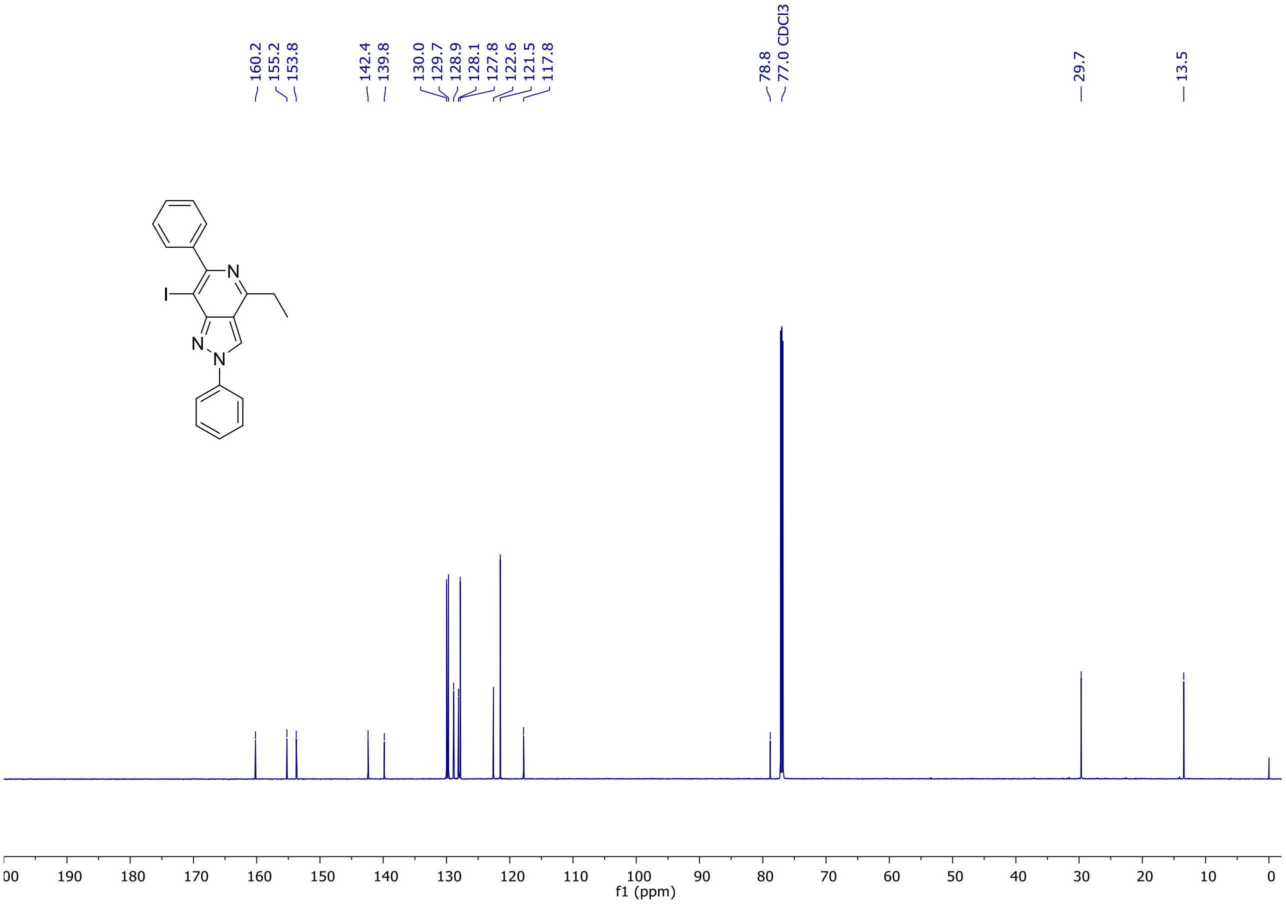

Figure S39. 4-Ethyl-7-iodo-2,6-diphenyl-2*H*-pyrazolo[4,3-*c*]pyridine (**15**). ^{13}C NMR (176 MHz, $CDCl_3$).

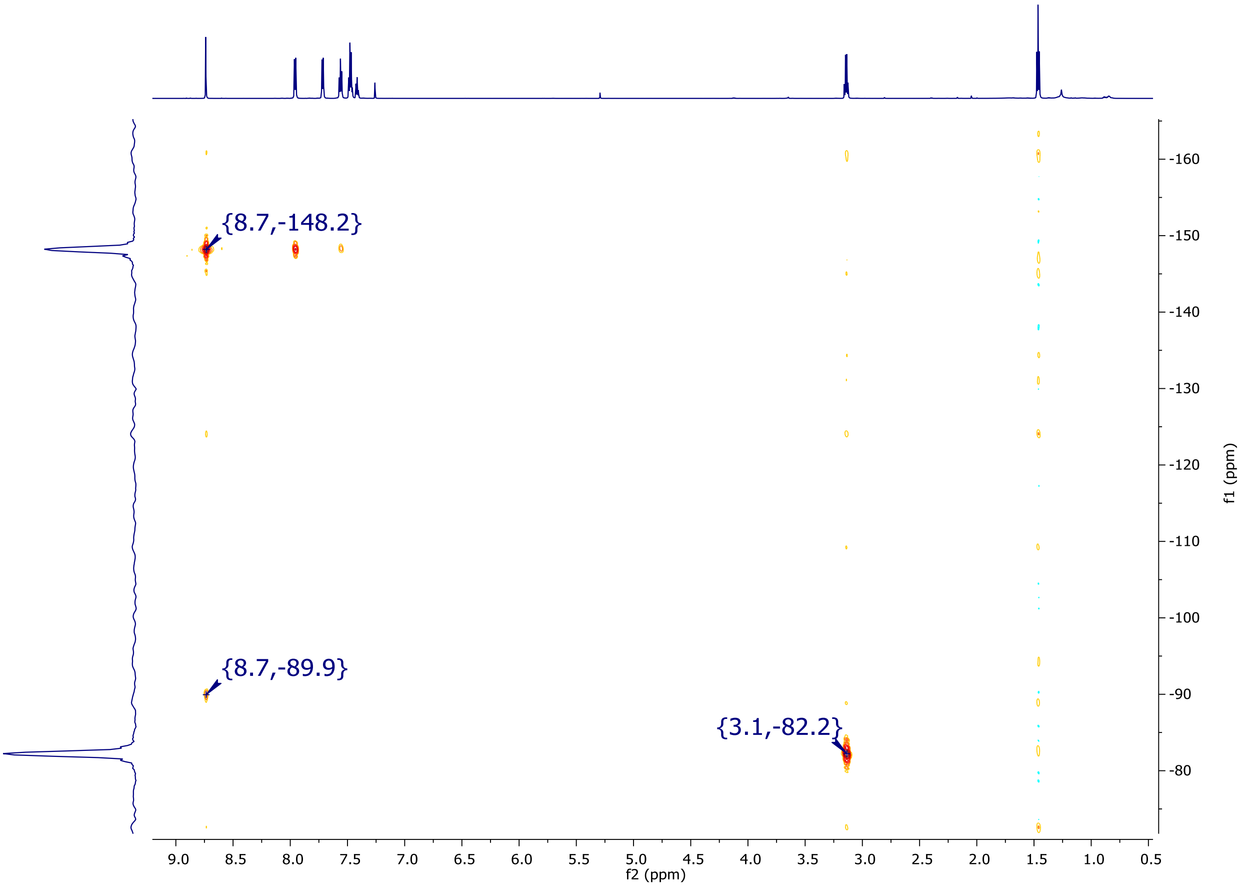

Figure S40. 4-Ethyl-7-iodo-2,6-diphenyl-2*H*-pyrazolo[4,3-*c*]pyridine (**15**). ^{1}H-^{15}N HMBC spectrum (700 MHz, $CDCl_3$)

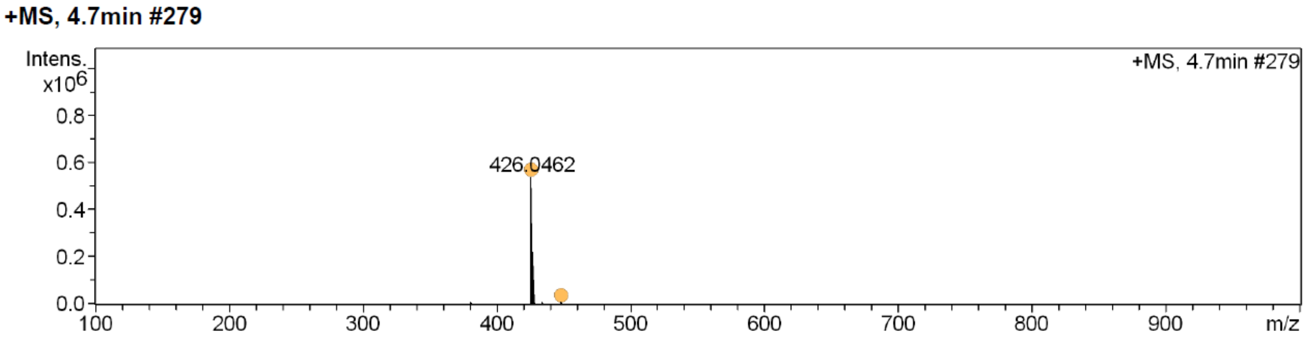

Meas. m/z	#	Ion Formula	m/z	err [ppm]	mSigma	# Sigma	Score	rdb	e⁻ Conf	N-Rule
426.0462	1	C20H17IN3	426.0462	0.0	1.6	1	100.00	13.5	even	ok
448.0274	1	C20H16IN3Na	448.0281	-1.7	14.6	1	100.00	13.5	even	ok

Figure S41. 4-Ethyl-7-iodo-2,6-diphenyl-2*H*-pyrazolo[4,3-*c*]pyridine (**15**). HRMS (ESI-TOF).

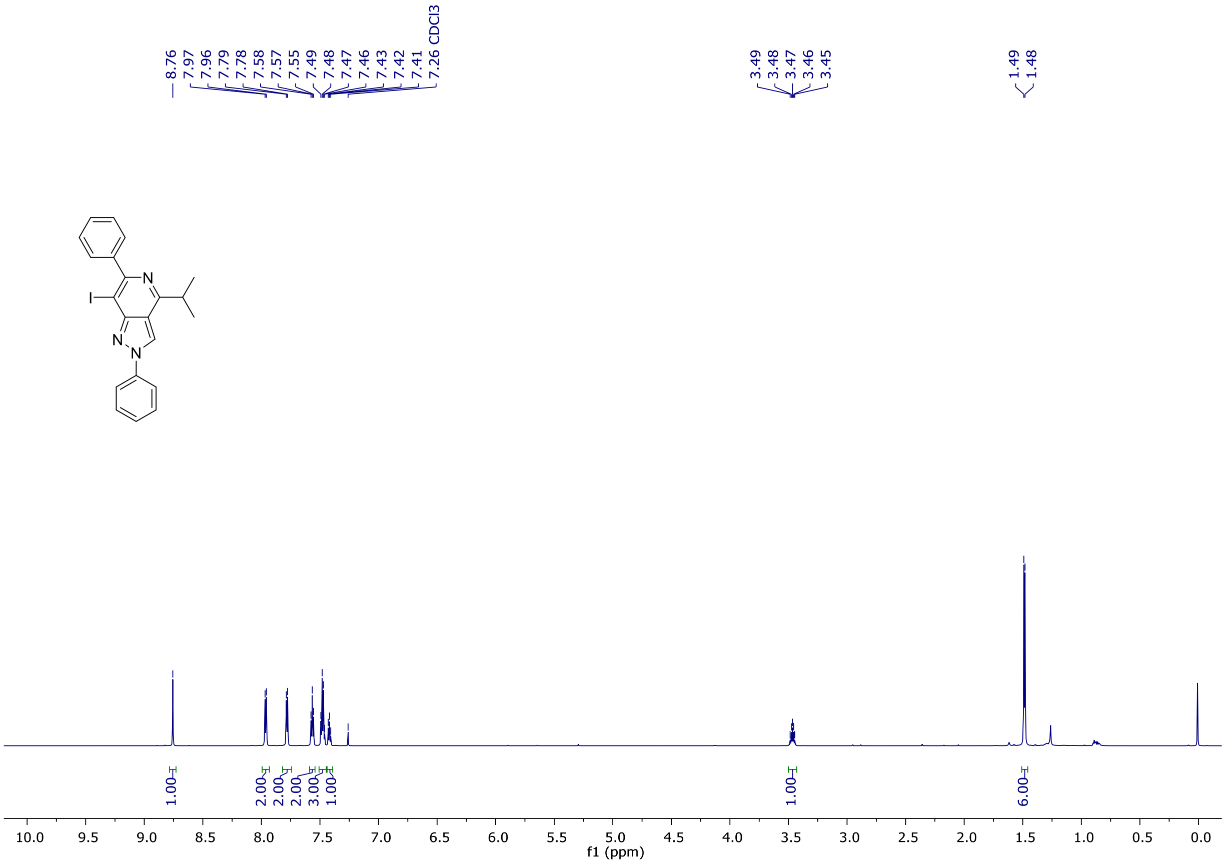

Figure S42. 7-Iodo-4-isopropyl-2,6-diphenyl-2*H*-pyrazolo[4,3-*c*]pyridine (**16**). ^{1}H NMR spectrum (700 MHz, $CDCl_3$).

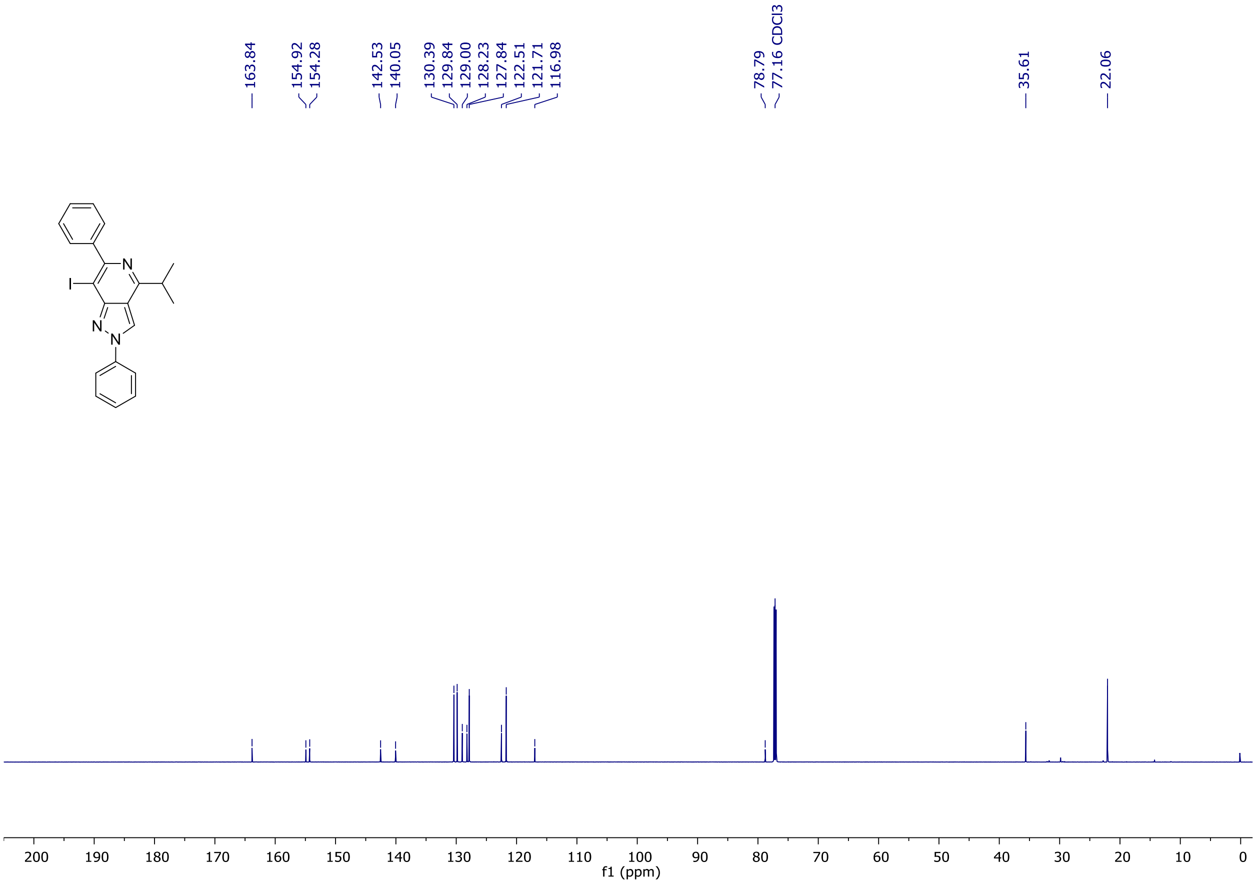

Figure S43. 7-Iodo-4-isopropyl-2,6-diphenyl-2*H*-pyrazolo[4,3-*c*]pyridine (**16**). ^{13}C NMR (176 MHz, $CDCl_3$).

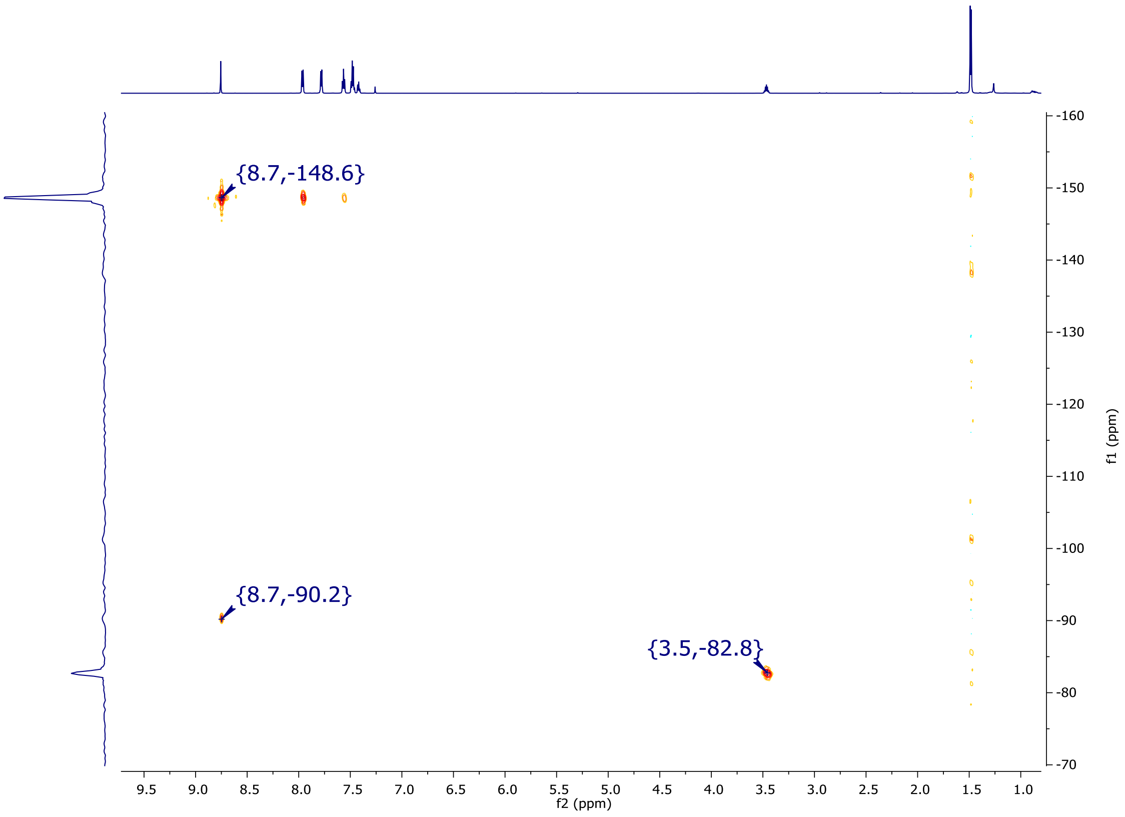

Figure S44. 7-Iodo-4-isopropyl-2,6-diphenyl-2*H*-pyrazolo[4,3-*c*]pyridine (**16**). ^{1}H-^{15}N HMBC spectrum (700 MHz, $CDCl_3$)

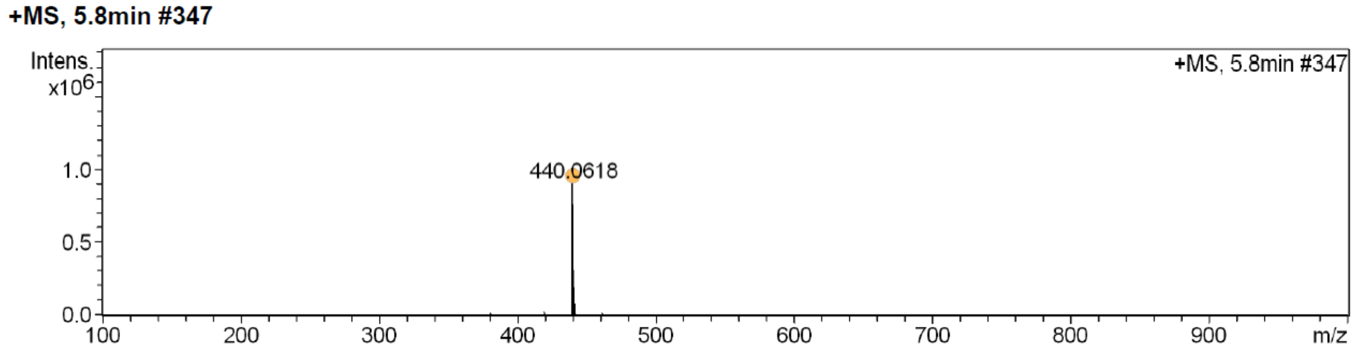

Meas. m/z	#	Ion Formula	m/z	err [ppm]	mSigma	# Sigma	Score	rdb	e⁻ Conf	N-Rule
440.0618	1	C21H19IN3	440.0618	0.0	7.8	1	100.00	13.5	even	ok

Figure S45. 7-Iodo-4-isopropyl-2,6-diphenyl-2*H*-pyrazolo[4,3-*c*]pyridine (**16**). HRMS (ESI-TOF).

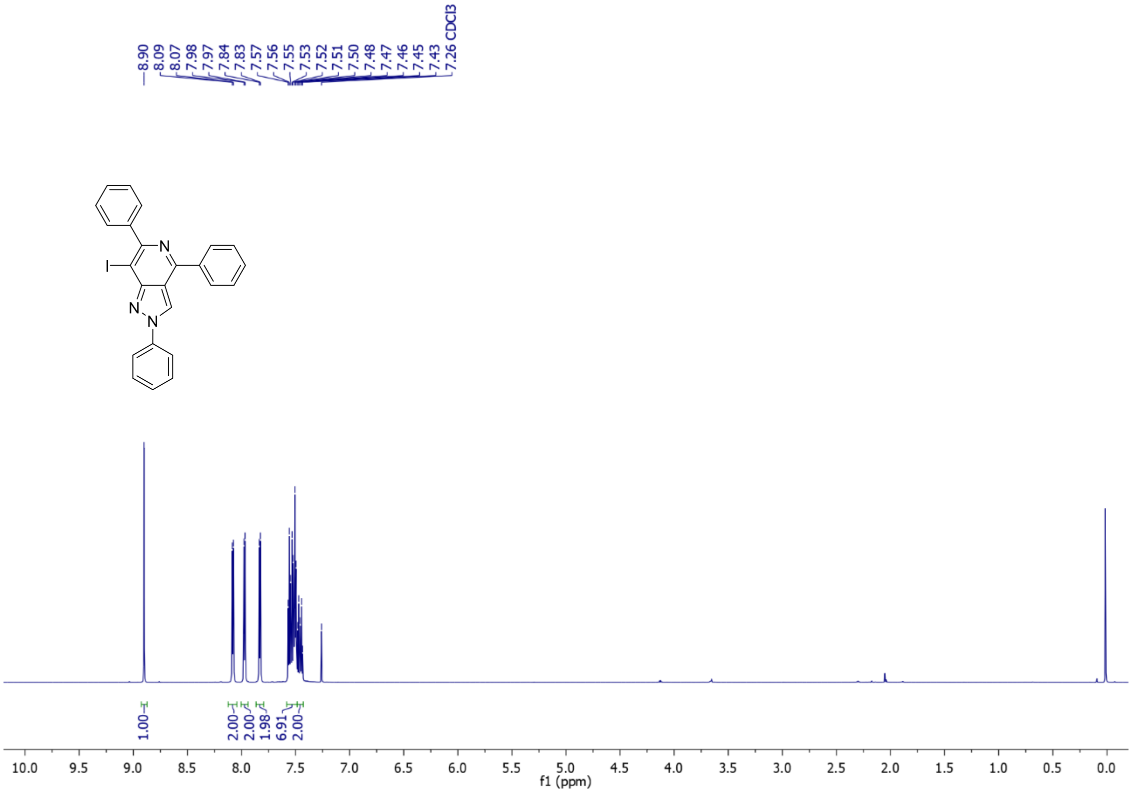

Figure S46. 7-Iodo-2,4,6-triphenyl-2*H*-pyrazolo[4,3-*c*]pyridine (**17**). ^{1}H NMR spectrum (700 MHz, $CDCl_3$).

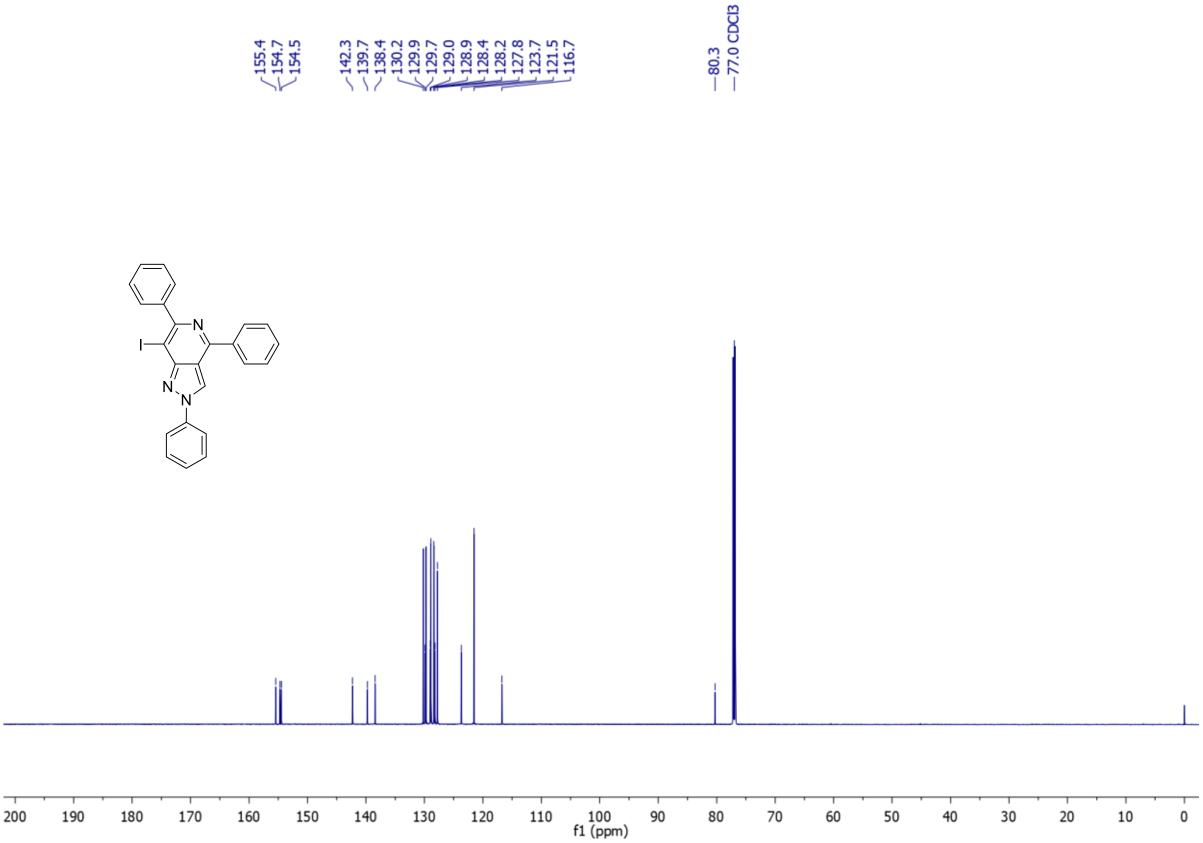

Figure S47. 7-Iodo-2,4,6-triphenyl-2*H*-pyrazolo[4,3-*c*]pyridine (**17**). ^{13}C NMR (176 MHz, $CDCl_3$).

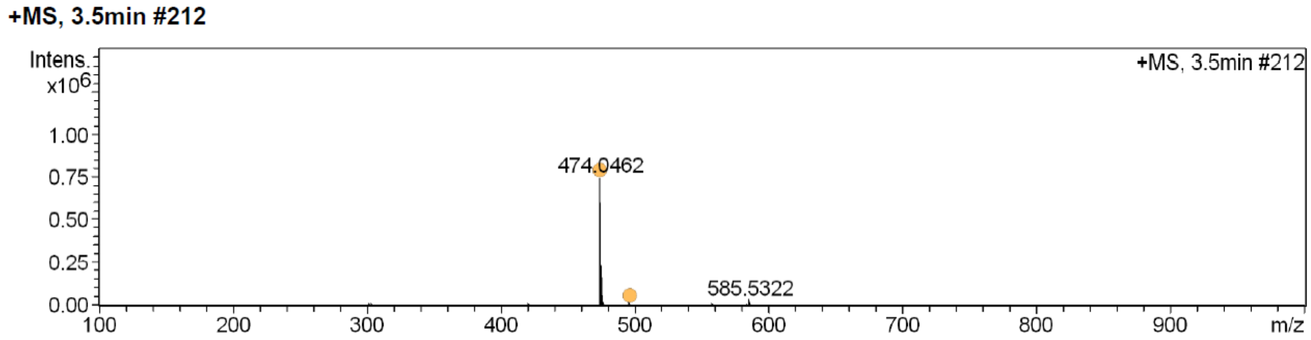

Meas. m/z	#	Ion Formula	m/z	err [ppm]	mSigma	# Sigma	Score	rdb	e⁻ Conf	N-Rule
474.0462	1	C24H17IN3	474.0462	-0.1	0.6	1	100.00	17.5	even	ok
496.0261	1	C24H16IN3Na	496.0281	4.0	21.4	1	100.00	17.5	even	ok

Figure S48. 7-Iodo-2,4,6-triphenyl-2*H*-pyrazolo[4,3-*c*]pyridine (**17**). HRMS (ESI-TOF).

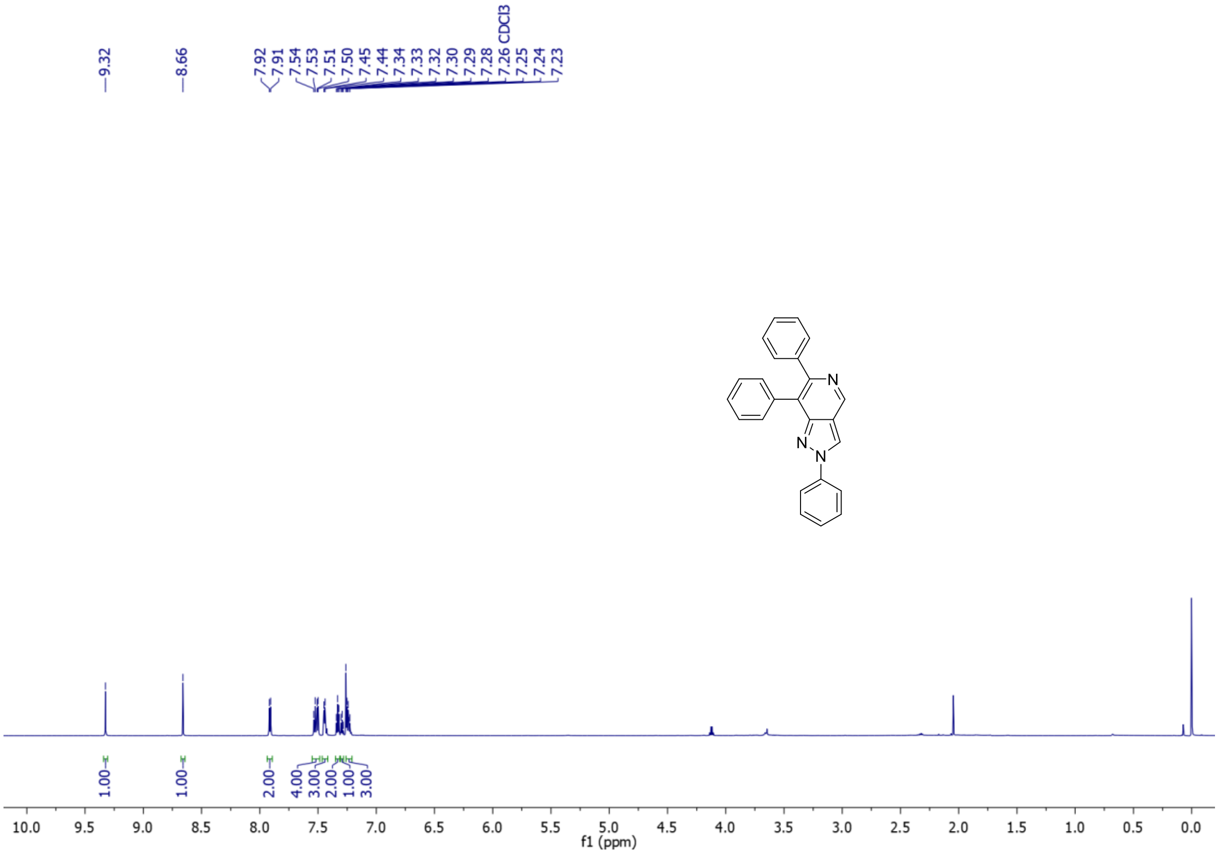

Figure S49. 2,6,7-Triphenyl-2*H*-pyrazolo[4,3-*c*]pyridine (**18**). ^{1}H NMR spectrum (700 MHz, $CDCl_3$).

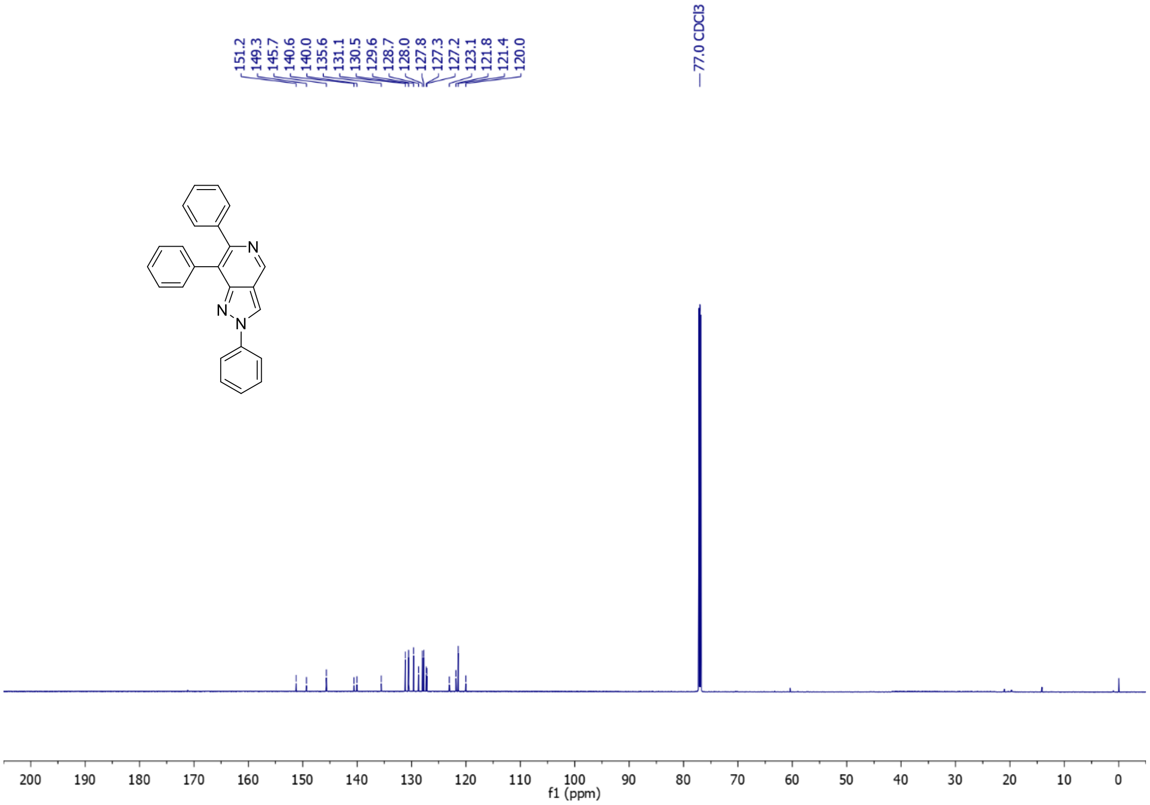

Figure S50. 2,6,7-Triphenyl-2*H*-pyrazolo[4,3-*c*]pyridine (**18**). ^{13}C NMR (176 MHz, $CDCl_3$).

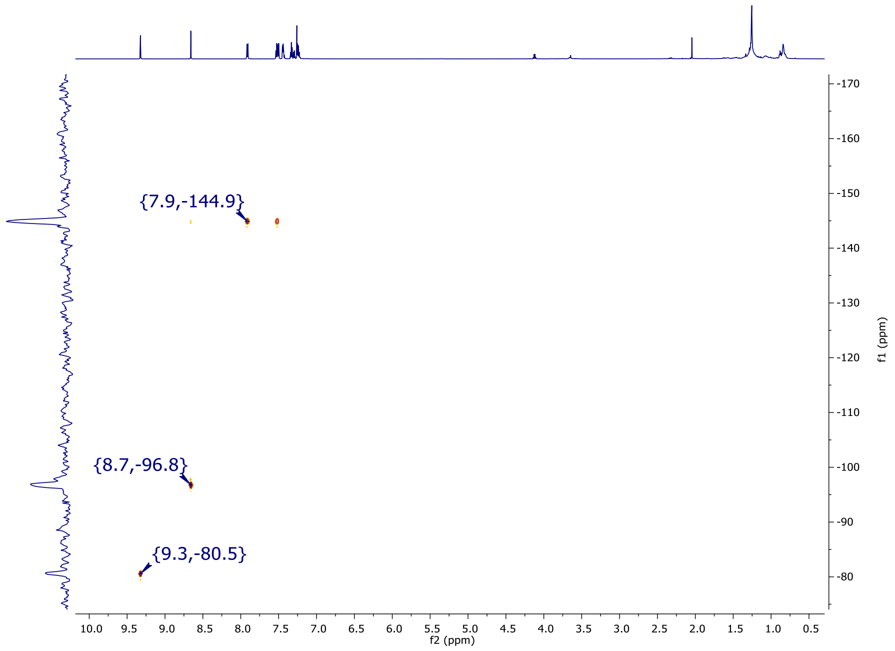

Figure S51. 2,6,7-Triphenyl-2*H*-pyrazolo[4,3-*c*]pyridine (**18**). ^{1}H-^{15}N LR-HSQMBC spectrum (700 MHz, $CDCl_3$)

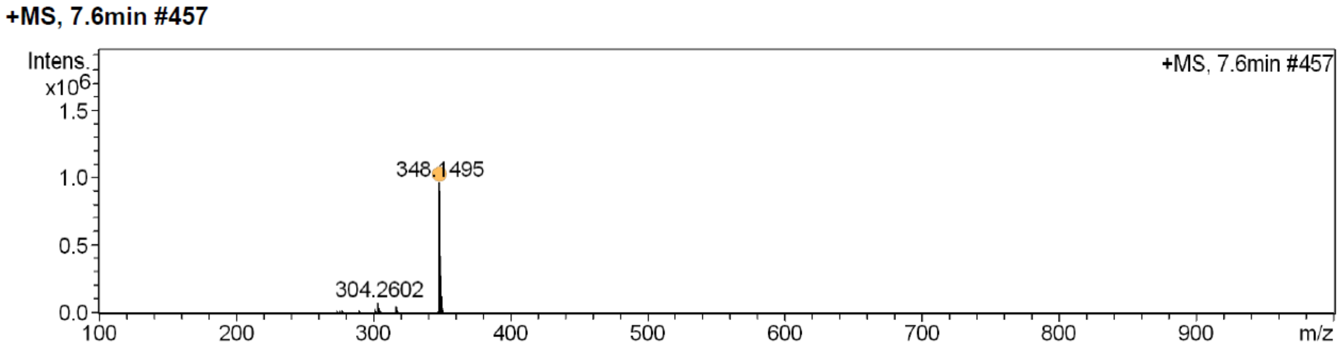

Meas. m/z	#	Ion Formula	m/z	err [ppm]	mSigma	# Sigma	Score	rdb	e⁻ Conf	N-Rule
348.1495	1	C24H18N3	348.1495	0.0	18.0	1	100.00	17.5	even	ok

Figure S52. 2,6,7-Triphenyl-2*H*-pyrazolo[4,3-*c*]pyridine (**18**). HRMS (ESI-TOF).

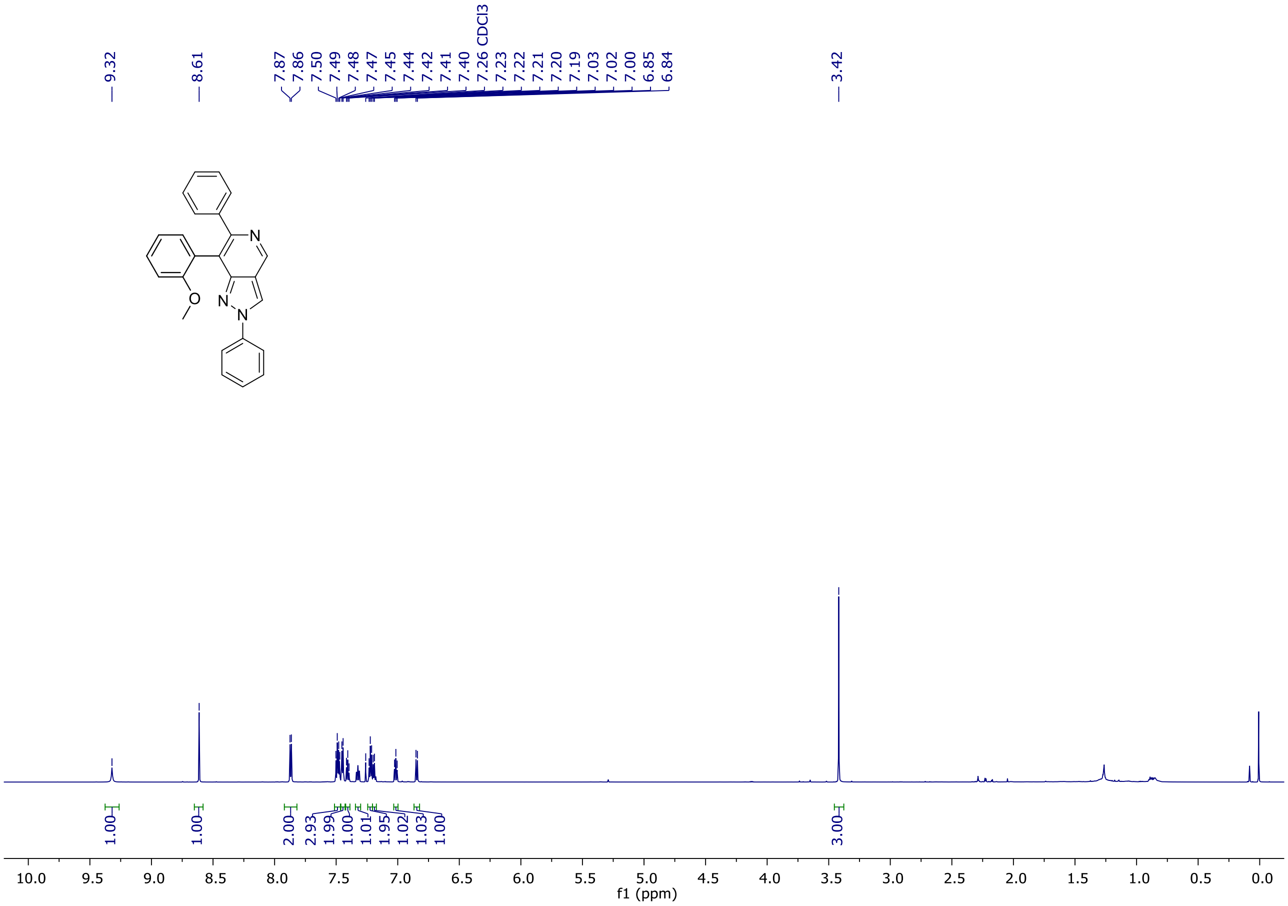

Figure S53. 7-(2-Methoxyphenyl)-2,6-diphenyl-2*H*-pyrazolo[4,3-*c*]pyridine (**19**). ^{1}H NMR spectrum (700 MHz, $CDCl_3$).

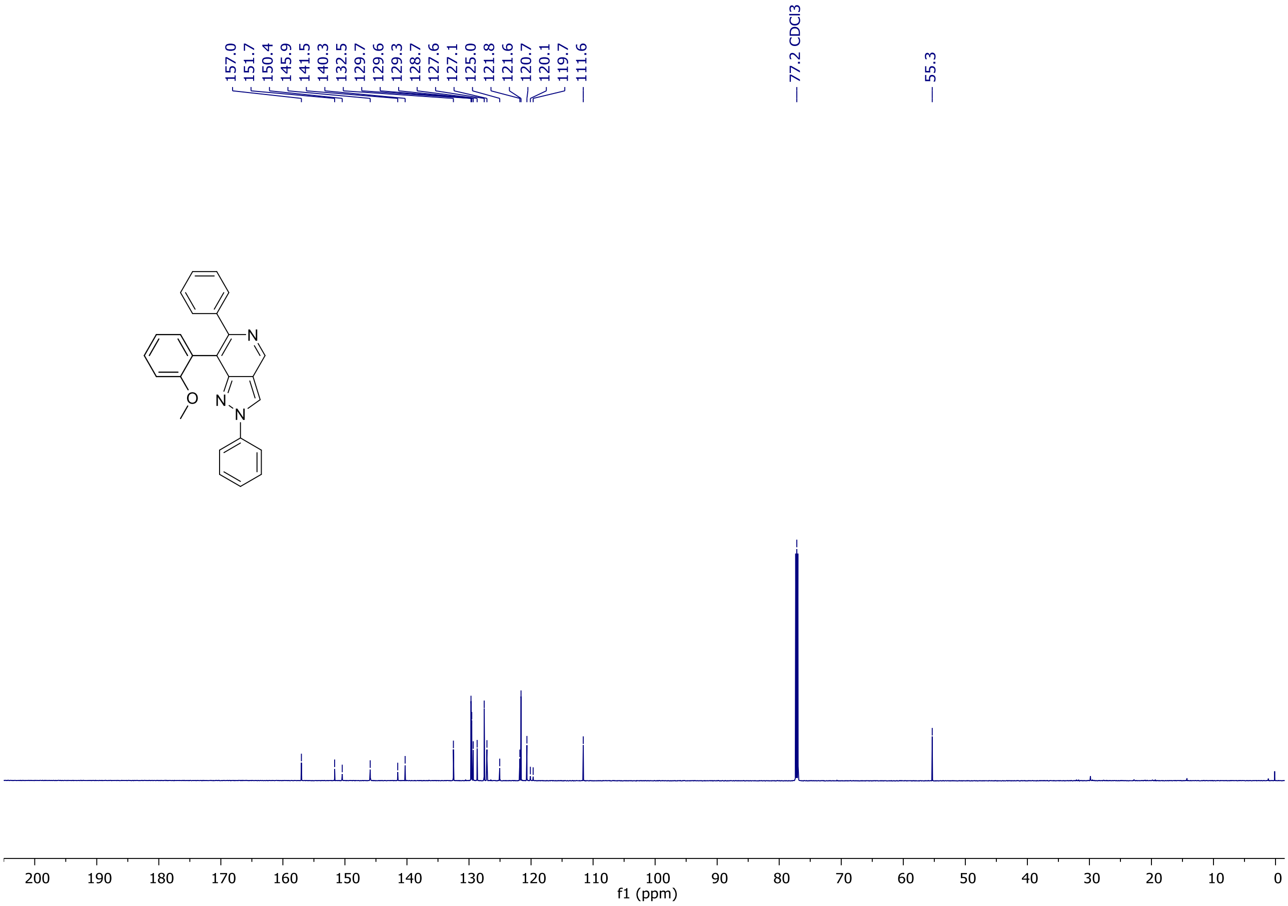

Figure S54. 7-(2-Methoxyphenyl)-2,6-diphenyl-2*H*-pyrazolo[4,3-*c*]pyridine (**19**). ^{13}C NMR (176 MHz, $CDCl_3$).

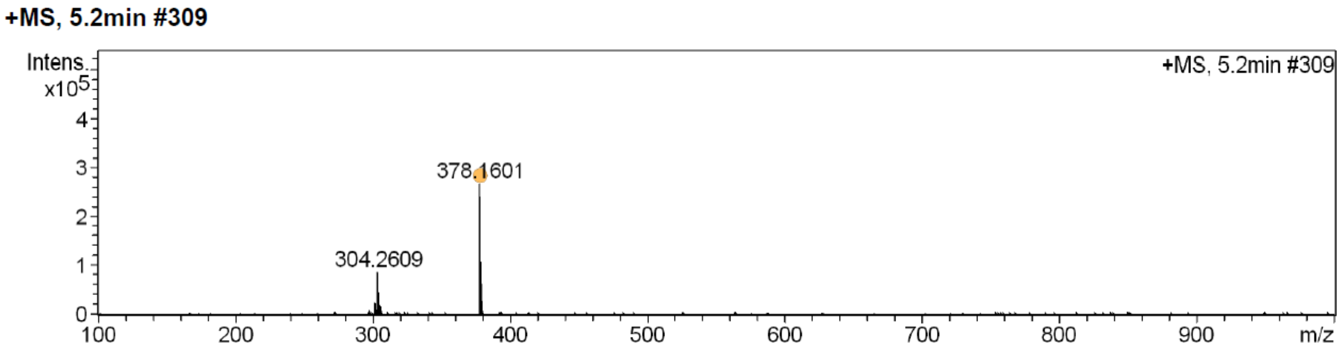

Meas. m/z	#	Ion Formula	m/z	err [ppm]	mSigma	# Sigma	Score	rdb	e⁻ Conf	N-Rule
378.1601	1	C25H20N3O	378.1601	-0.1	10.4	1	100.00	17.5	even	ok

Figure S55. 7-(2-Methoxyphenyl)-2,6-diphenyl-2*H*-pyrazolo[4,3-*c*]pyridine (**19**). HRMS (ESI-TOF).

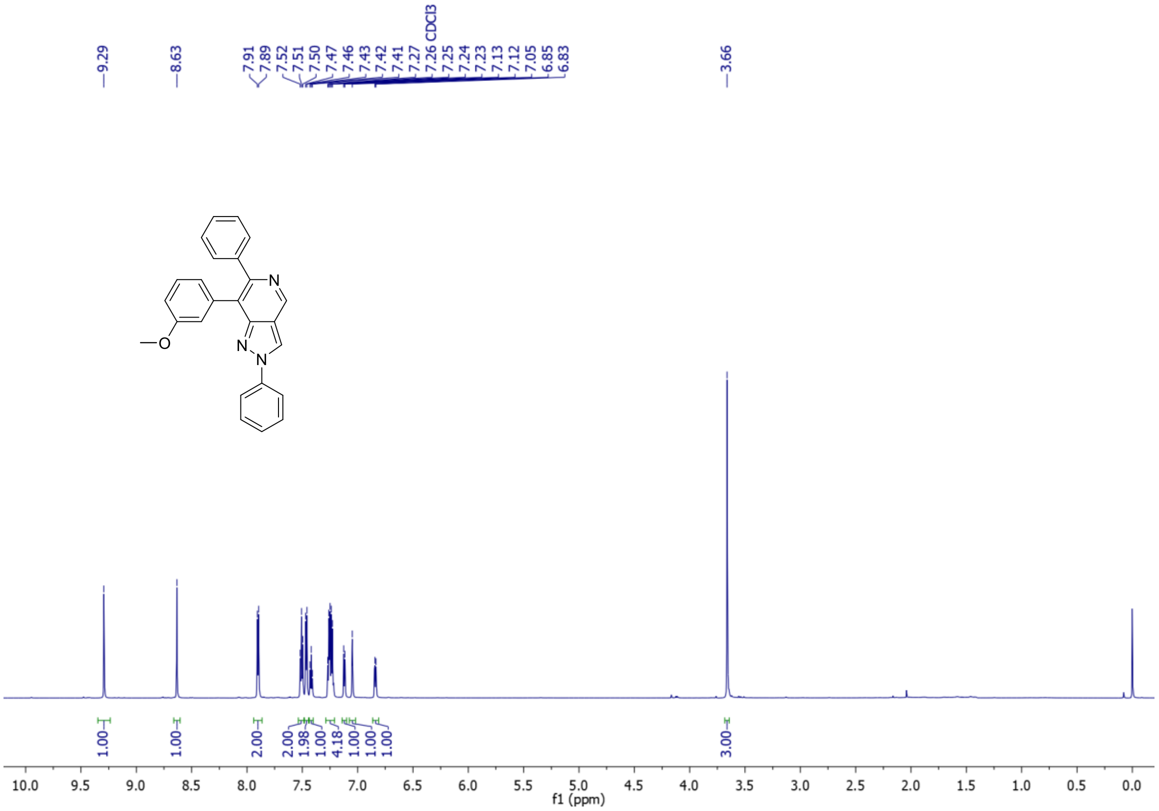

Figure S56. 7-(3-Methoxyphenyl)-2,6-diphenyl-2*H*-pyrazolo[4,3-*c*]pyridine (**20**). ^{1}H NMR spectrum (700 MHz, $CDCl_3$).

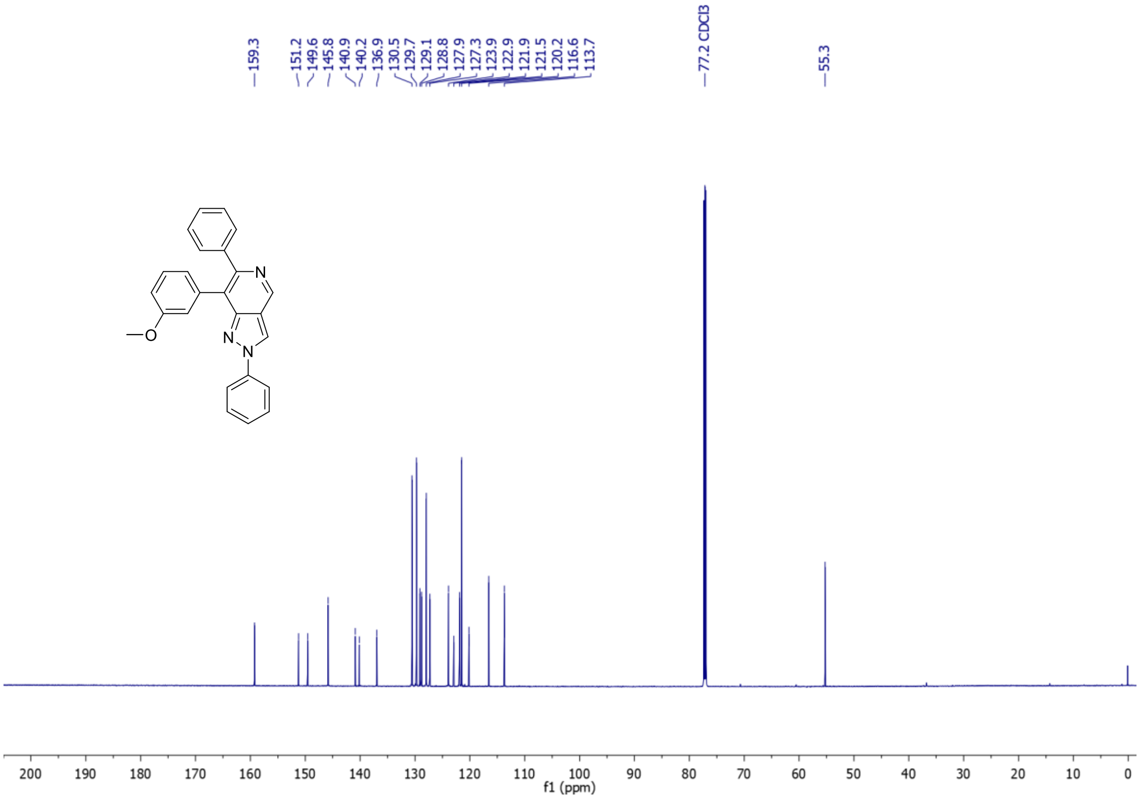

Figure S57. 7-(3-Methoxyphenyl)-2,6-diphenyl-2*H*-pyrazolo[4,3-*c*]pyridine (**20**). ^{13}C NMR (176 MHz, $CDCl_3$).

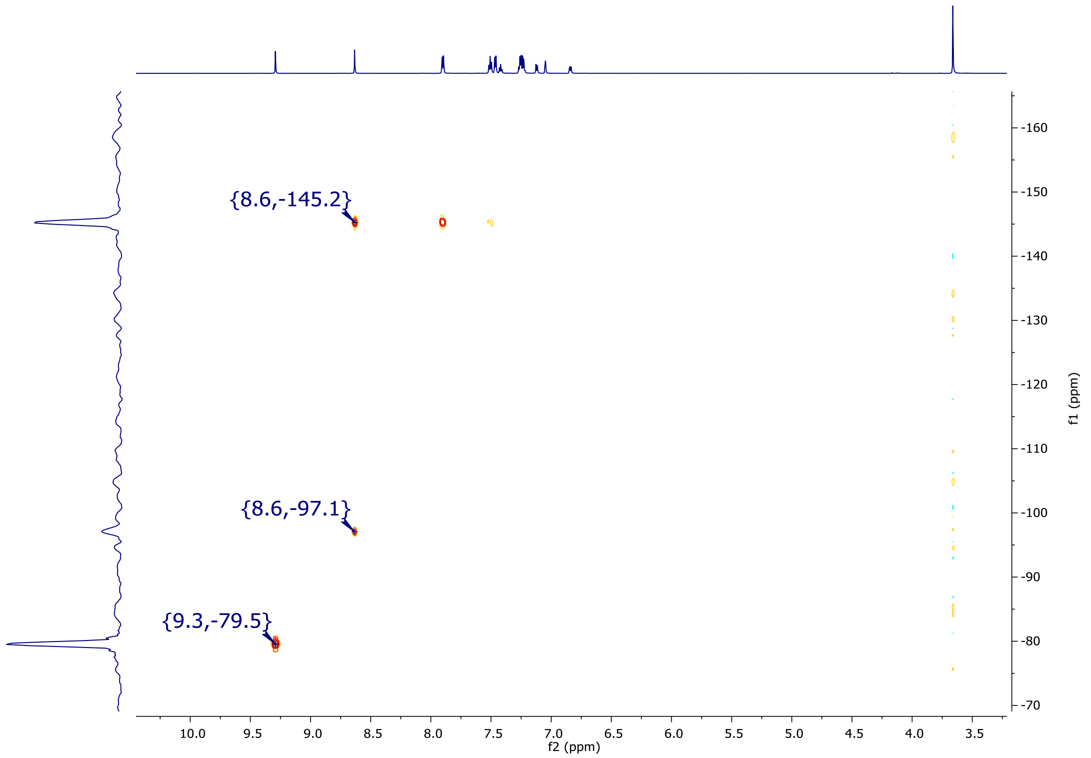

Figure S58. 7-(3-Methoxyphenyl)-2,6-diphenyl-2*H*-pyrazolo[4,3-*c*]pyridine (**20**). ^{1}H-^{15}N HMBC spectrum (700 MHz, $CDCl_3$).

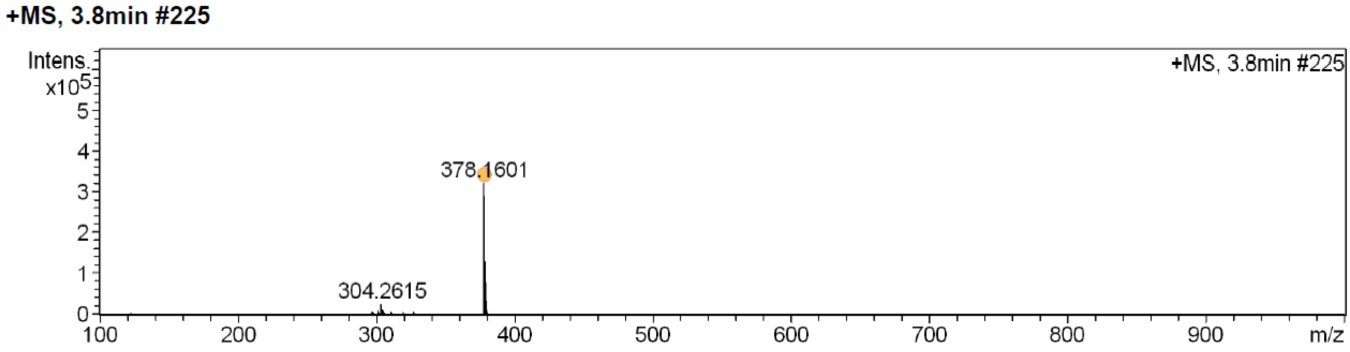

Meas. m/z	#	Ion Formula	m/z	err [ppm]	mSigma	# Sigma	Score	rdb	e⁻ Conf	N-Rule
378.1601	1	C25H20N3O	378.1601	0.1	4.4	1	100.00	17.5	even	ok

Figure S59. 7-(3-Methoxyphenyl)-2,6-diphenyl-2*H*-pyrazolo[4,3-*c*]pyridine (**20**). HRMS (ESI-TOF).

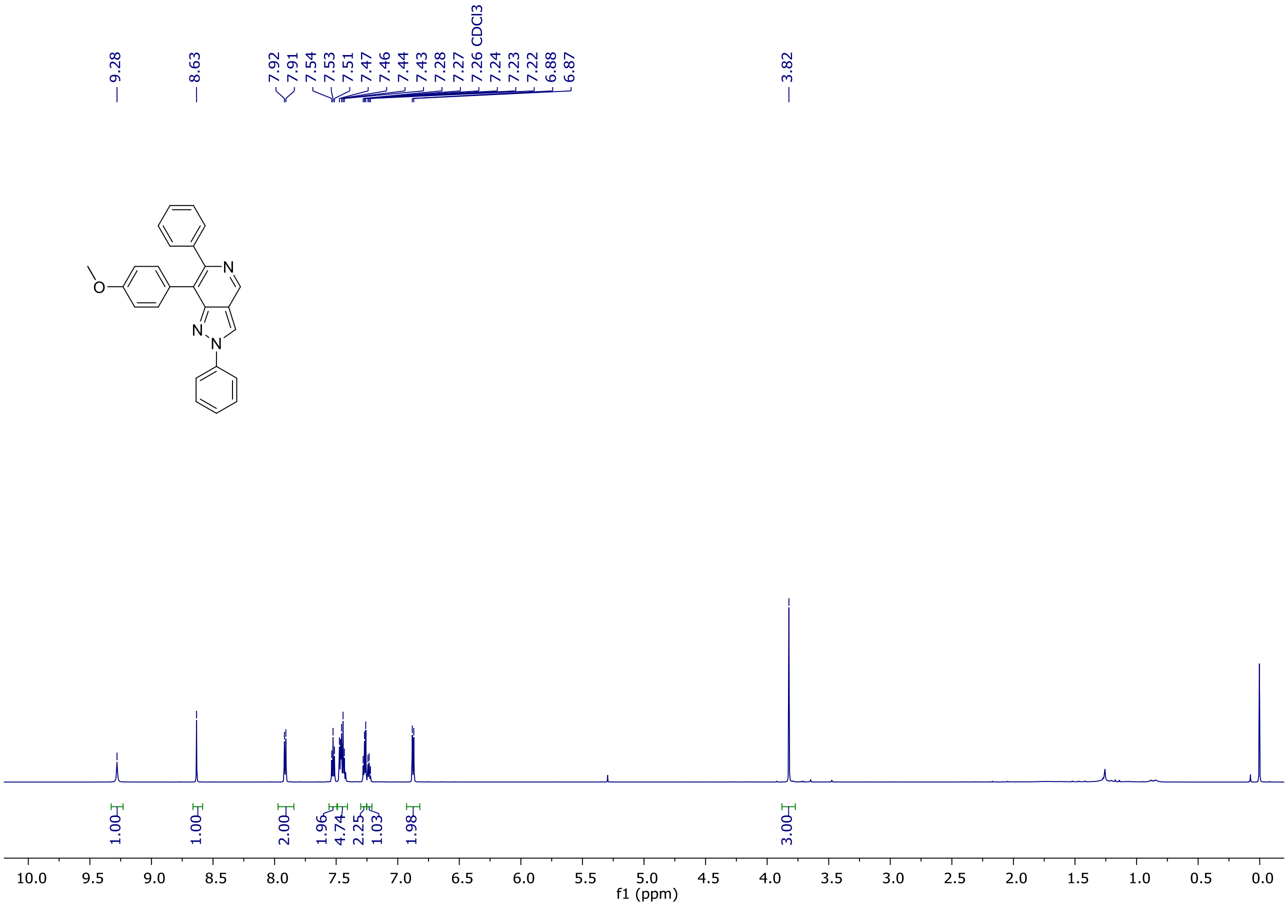

Figure S60. 7-(4-Methoxyphenyl)-2,6-diphenyl-2*H*-pyrazolo[4,3-*c*]pyridine (**21**). ^{1}H NMR spectrum (700 MHz, $CDCl_3$).

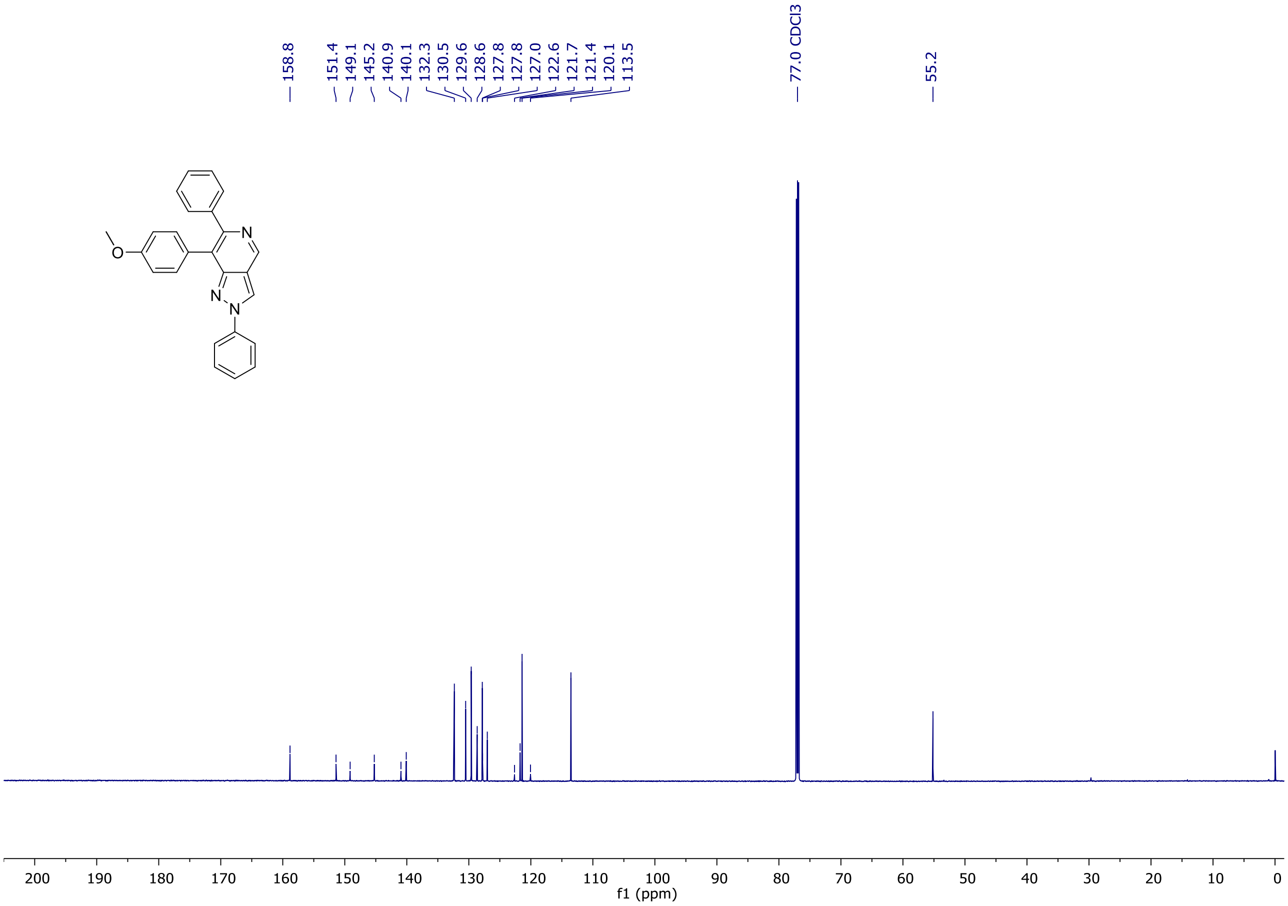

Figure S61. 7-(4-Methoxyphenyl)-2,6-diphenyl-2*H*-pyrazolo[4,3-*c*]pyridine (**21**). ^{13}C NMR (176 MHz, $CDCl_3$).

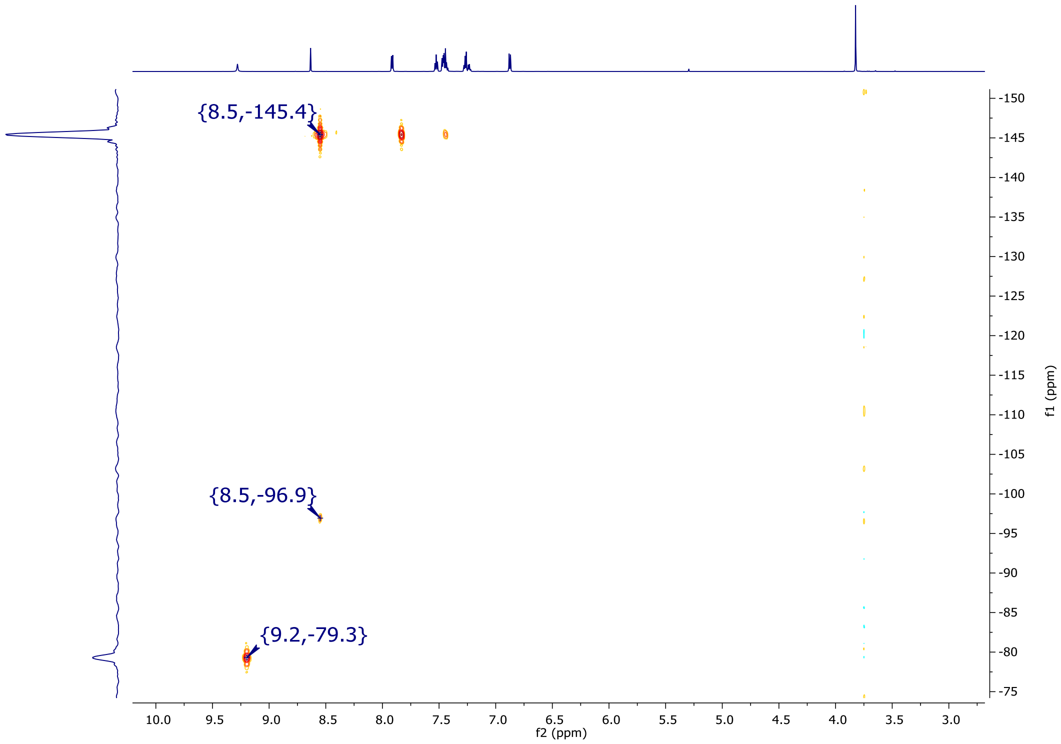

Figure S62. 7-(4-Methoxyphenyl)-2,6-diphenyl-2*H*-pyrazolo[4,3-*c*]pyridine (**21**). ^{1}H-^{15}N HMBC spectrum (700 MHz, $CDCl_3$).

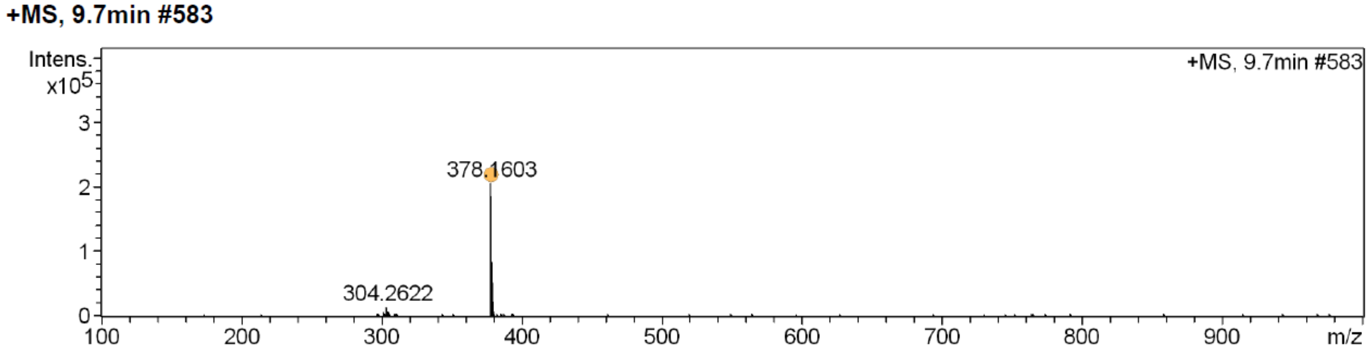

Meas. m/z	#	Ion Formula	m/z	err [ppm]	mSigma	# Sigma	Score	rdb	e⁻ Conf	N-Rule
378.1603	1	C25H20N3O	378.1601	-0.6	1.3	1	100.00	17.5	even	ok

Figure S63. 7-(4-Methoxyphenyl)-2,6-diphenyl-2*H*-pyrazolo[4,3-*c*]pyridine (**21**). HRMS (ESI-TOF).

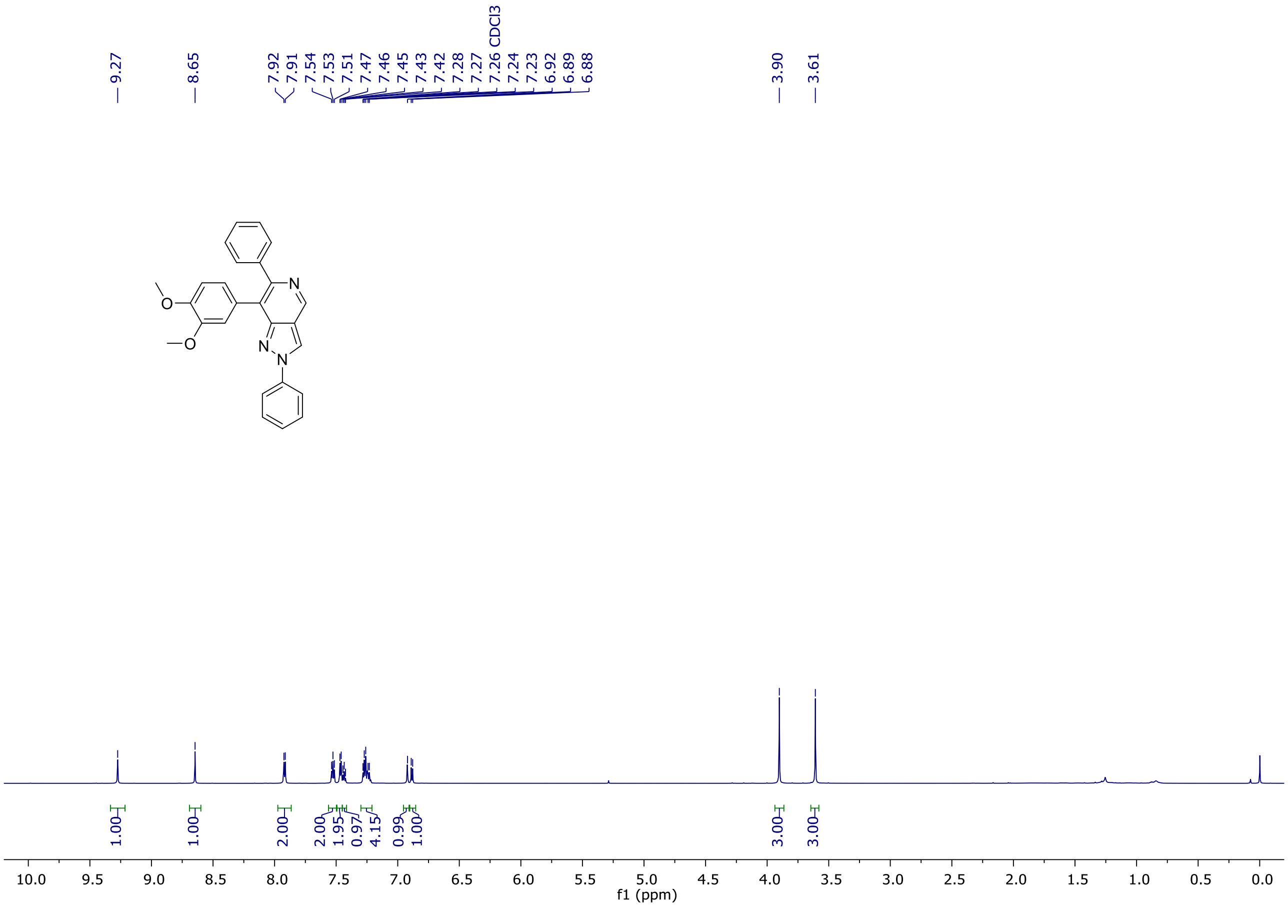

Figure S64. 7-(3,4-Dimethoxyphenyl)-2,6-diphenyl-2*H*-pyrazolo[4,3-*c*]pyridine (**22**). ^{1}H NMR spectrum (700 MHz, $CDCl_3$).

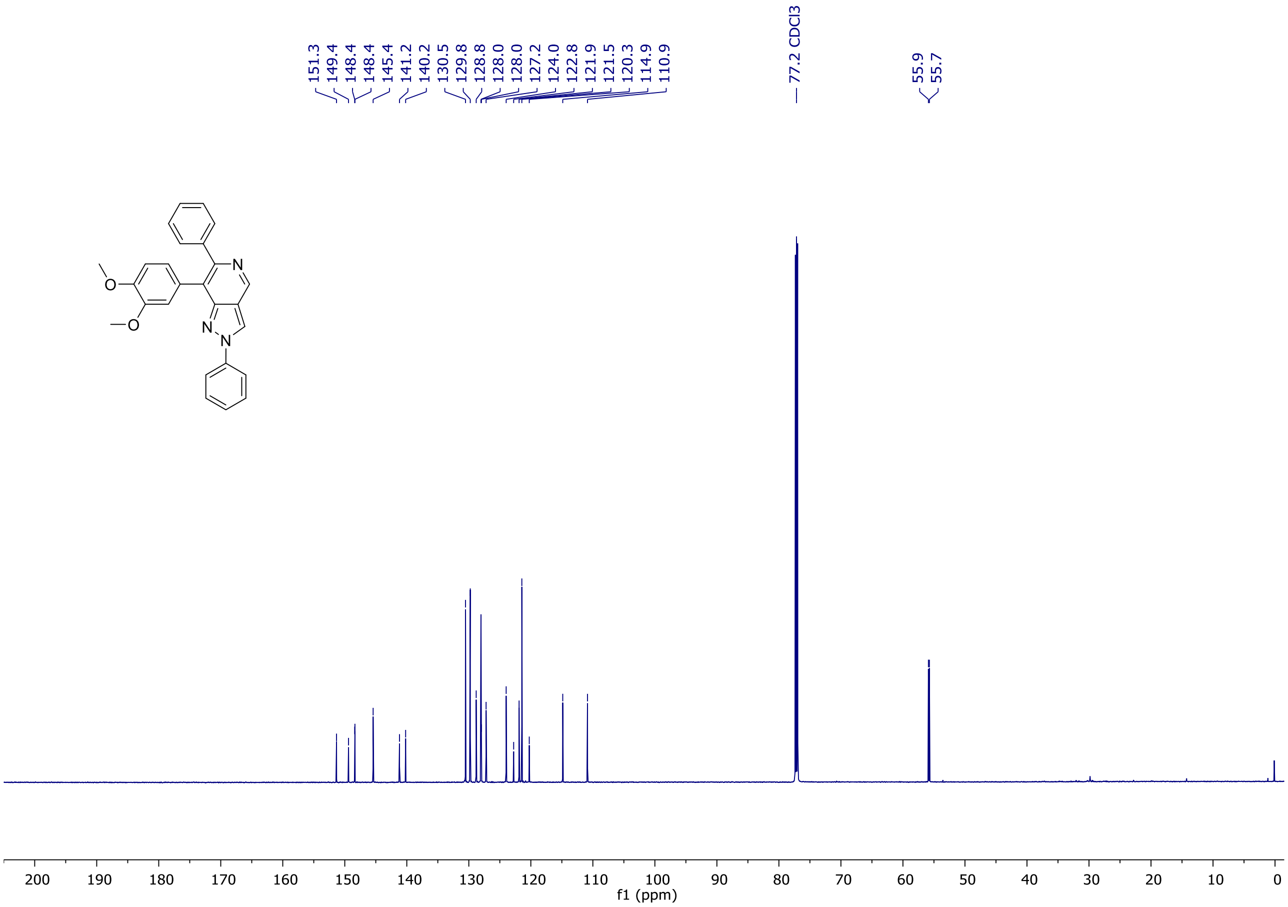

Figure S65. 7-(3,4-Dimethoxyphenyl)-2,6-diphenyl-2*H*-pyrazolo[4,3-*c*]pyridine (**22**). ^{13}C NMR (176 MHz, $CDCl_3$).

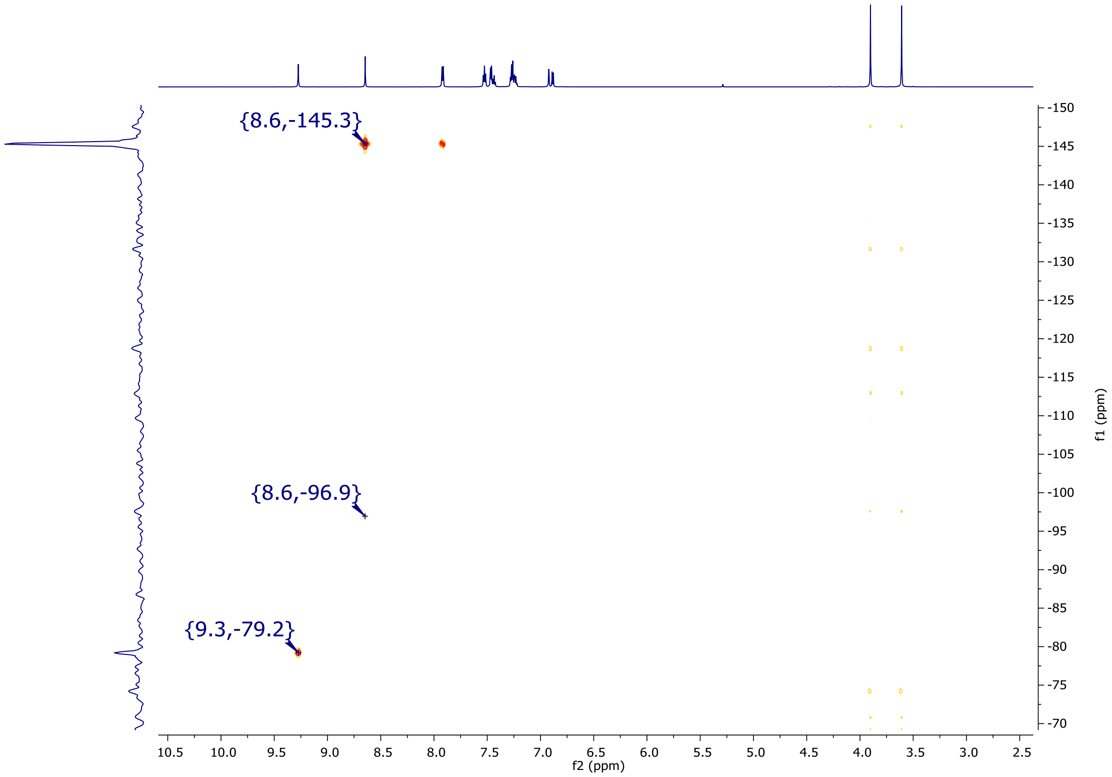

Figure S66. 7-(3,4-Dimethoxyphenyl)-2,6-diphenyl-2*H*-pyrazolo[4,3-*c*]pyridine (**22**). ^{1}H-^{15}N HMBC spectrum (700 MHz, $CDCl_3$).

+MS, 3.1min #185

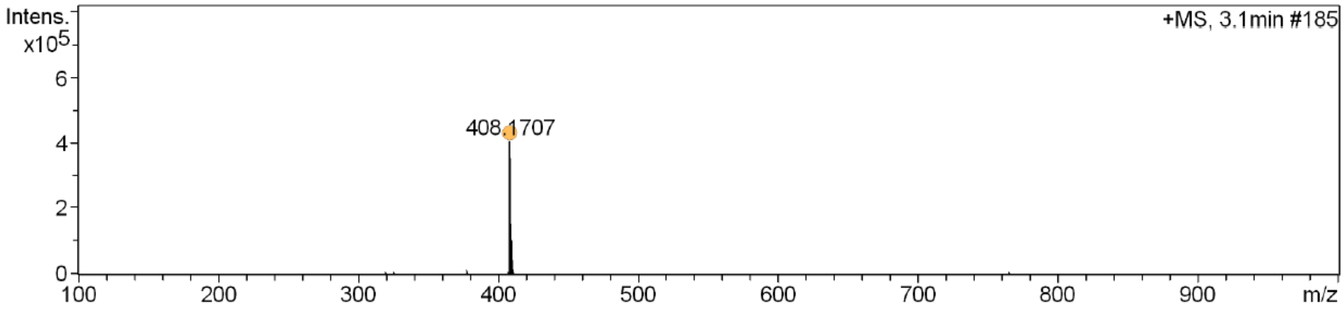

Meas. m/z	#	Ion Formula	m/z	err [ppm]	mSigma	# Sigma	Score	rdb	e⁻ Conf	N-Rule
408.1707	1	C26H22N3O2	408.1707	0.0	2.7	1	100.00	17.5	even	ok

Figure S67. 7-(3,4-Dimethoxyphenyl)-2,6-diphenyl-2*H*-pyrazolo[4,3-*c*]pyridine (**22**). HRMS (ESI-TOF).

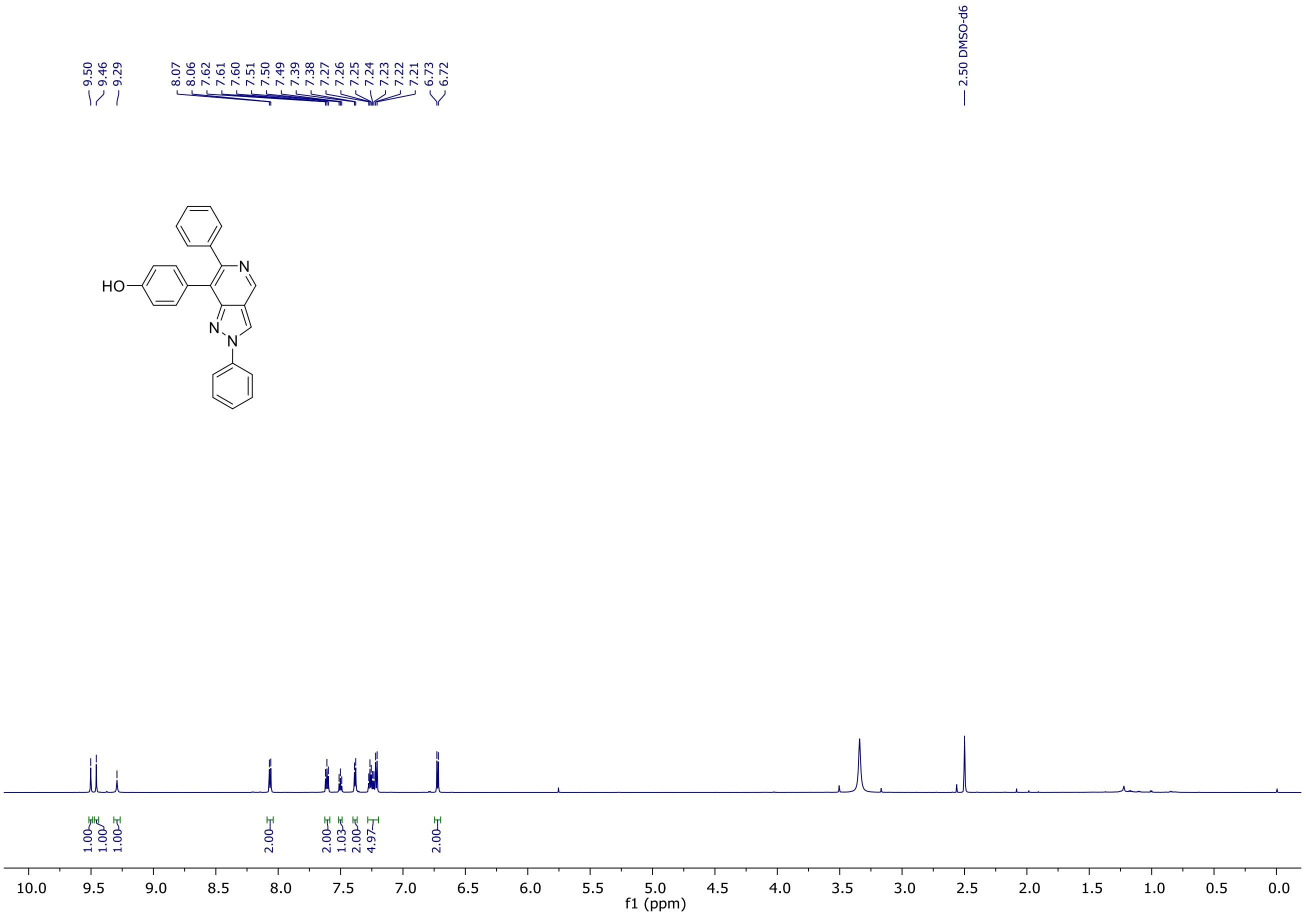

Figure S68. 4-(2,6-Diphenyl-2*H*-pyrazolo[4,3-*c*]pyridin-7-yl)phenol (**23**). ^{1}H NMR spectrum (700 MHz, DMSO-d_6).

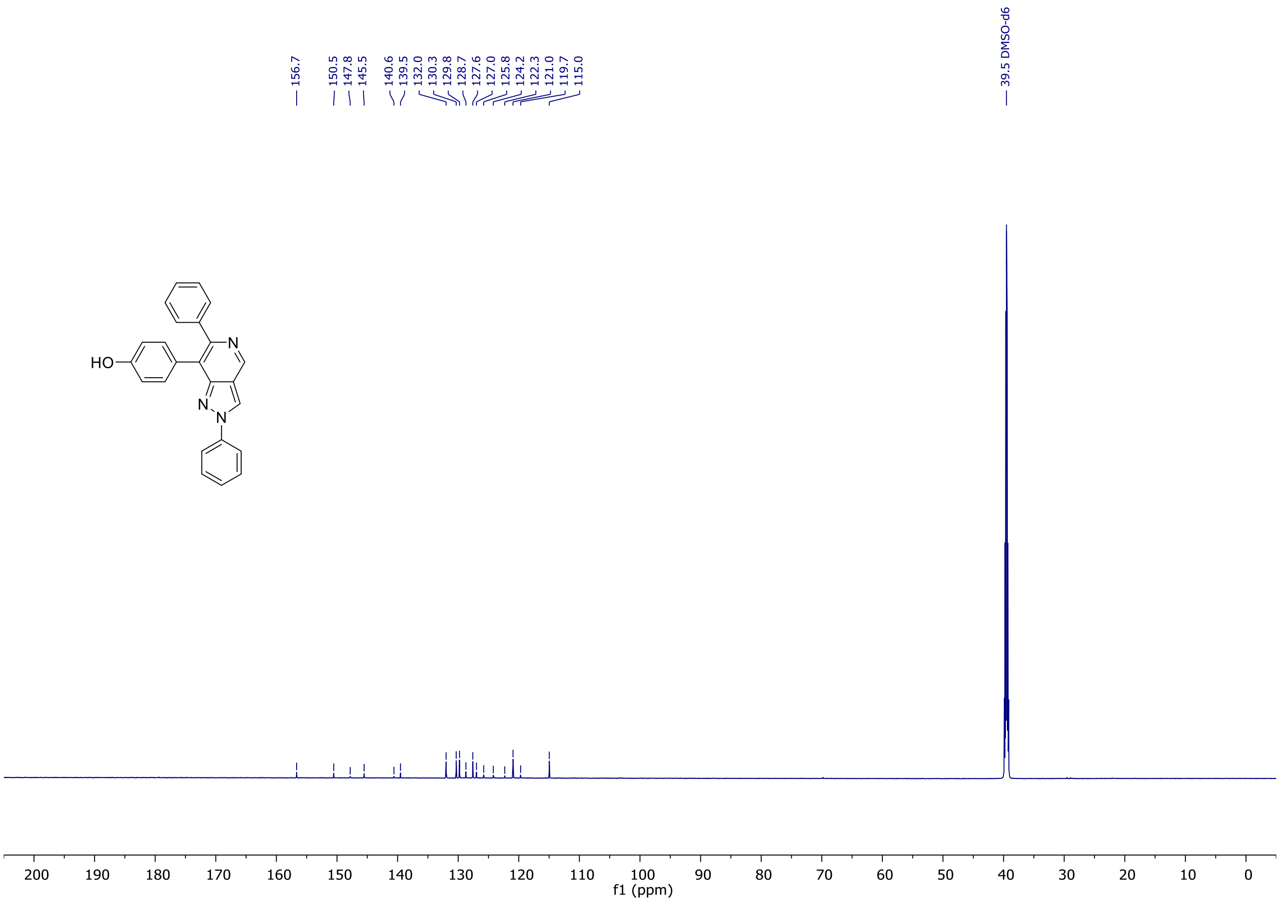

Figure S69. 4-(2,6-Diphenyl-2*H*-pyrazolo[4,3-*c*]pyridin-7-yl)phenol (**23**). ^{13}C NMR (176 MHz, DMSO-d_6).

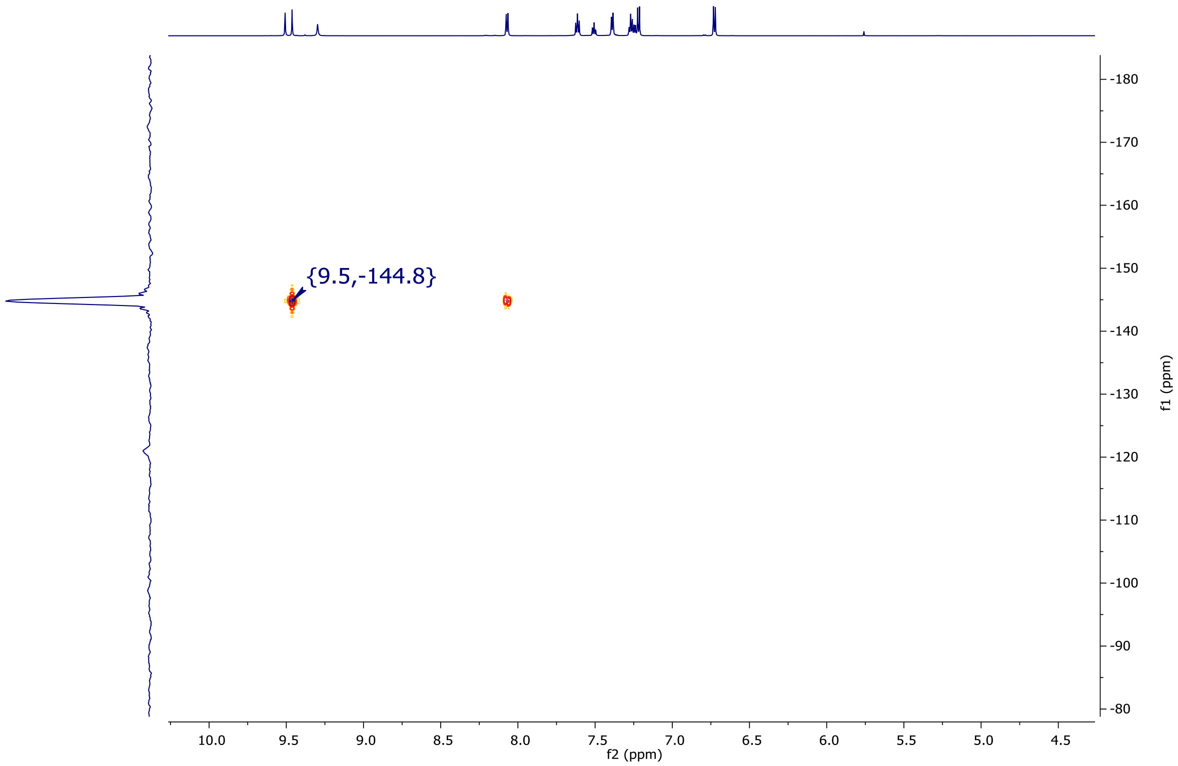

Figure S70. 4-(2,6-Diphenyl-2*H*-pyrazolo[4,3-*c*]pyridin-7-yl)phenol (**23**). ^{1}H-^{15}N HMBC spectrum (700 MHz, DMSO-d_6).

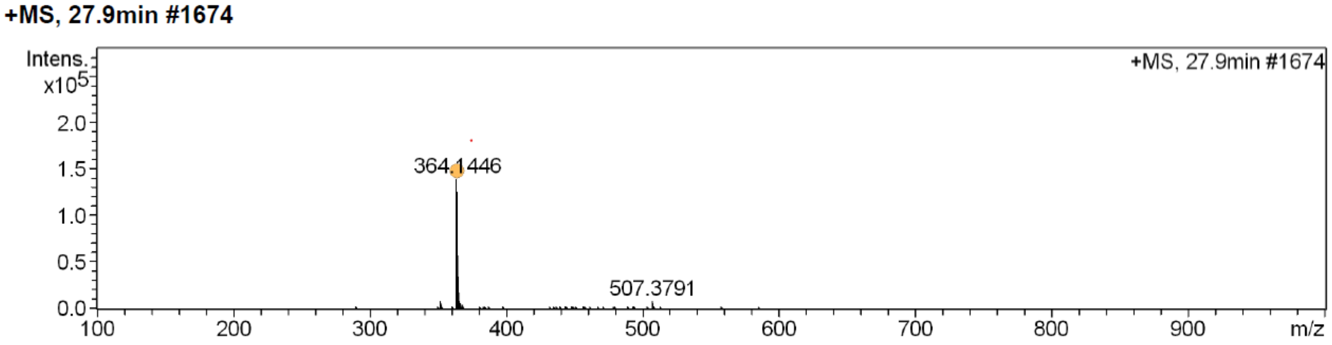

Meas. m/z	#	Ion Formula	m/z	err [ppm]	mSigma	# Sigma	Score	rdb	e⁻ Conf	N-Rule
364.1446	1	C24H18N3O	364.1444	0.4	3.3	1	100.00	17.5	even	ok

Figure S71. 4-(2,6-Diphenyl-2*H*-pyrazolo[4,3-*c*]pyridin-7-yl)phenol (**23**). HRMS (ESI-TOF).

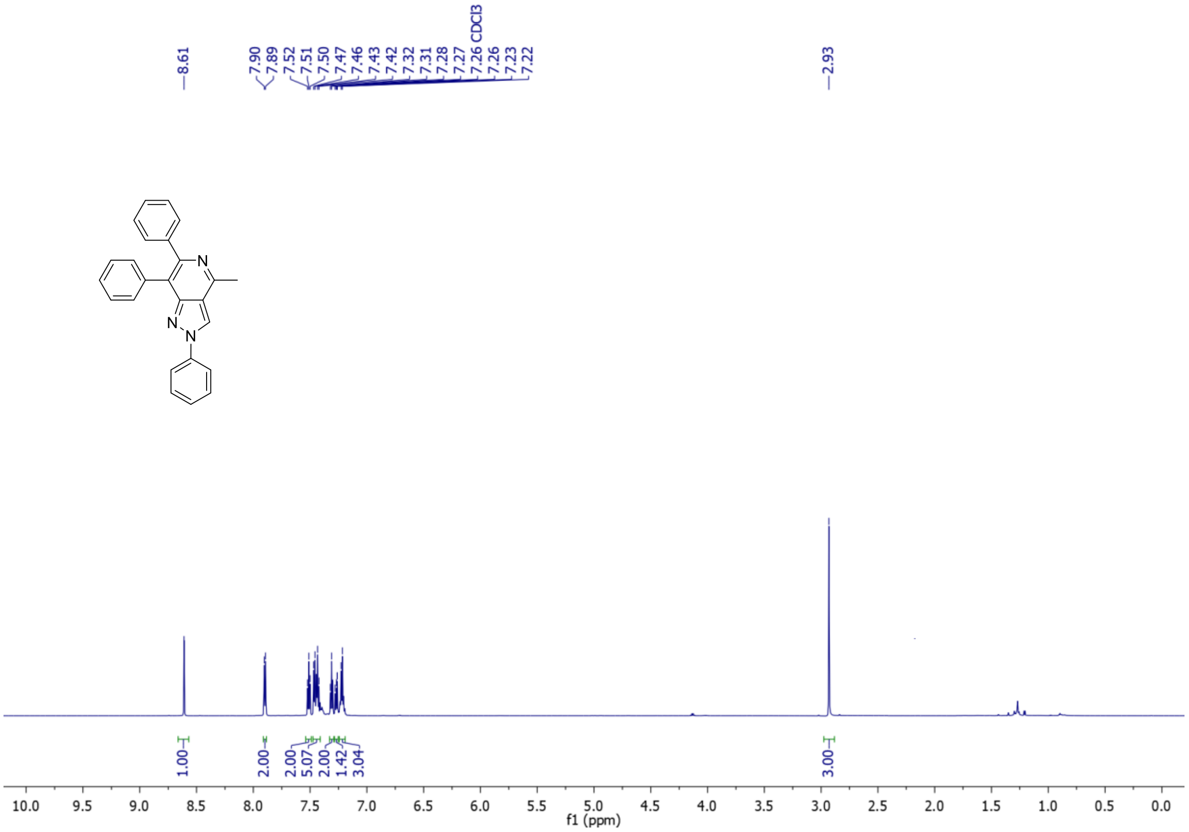

Figure S72. 4-Methyl-2,6,7-triphenyl-2*H*-pyrazolo[4,3-*c*]pyridine (**24**). ^{1}H NMR spectrum (700 MHz, $CDCl_3$).

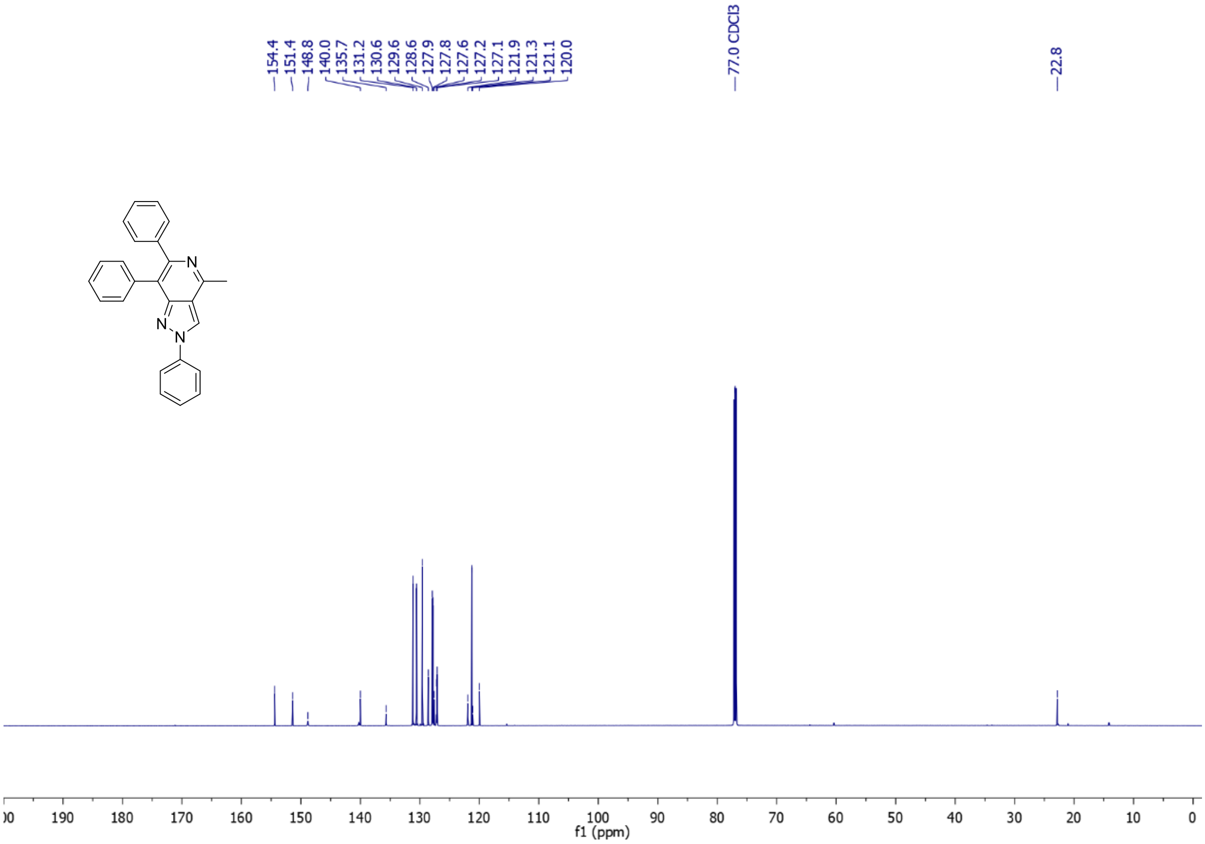

Figure S73. 4-Methyl-2,6,7-triphenyl-2*H*-pyrazolo[4,3-*c*]pyridine (**24**). ^{13}C NMR (176 MHz, $CDCl_3$).

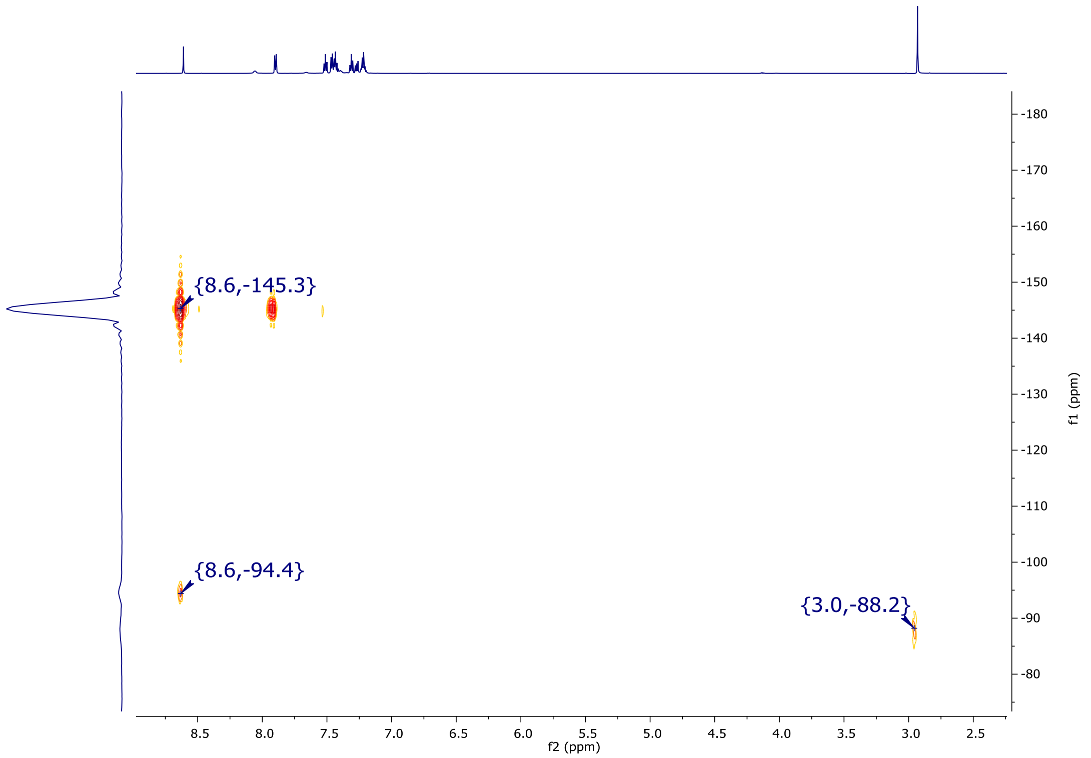

Figure S74. 4-Methyl-2,6,7-triphenyl-2*H*-pyrazolo[4,3-*c*]pyridine (**24**). ^{1}H-^{15}N HMBC spectrum (700 MHz, $CDCl_3$).

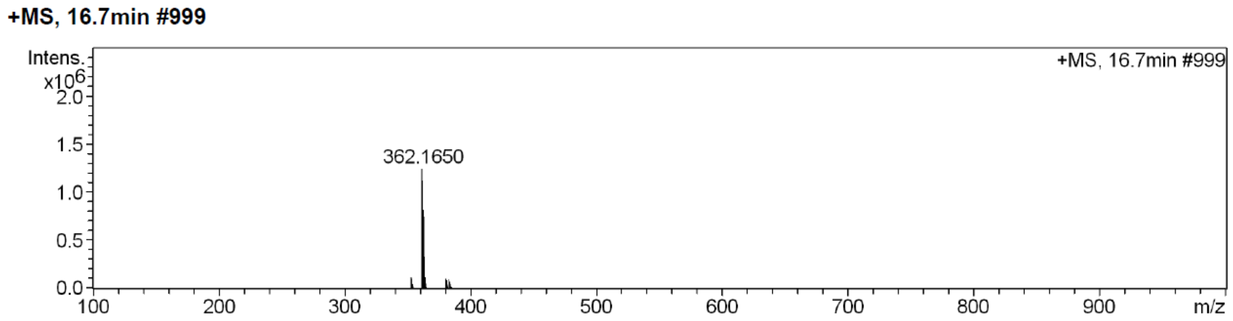

Meas. m/z	#	Ion Formula	m/z	err [ppm]	mSigma	# Sigma	Score	rdb	e⁻ Conf	N-Rule
362.1650	1	C25H20N3	362.1652	0.6	218.5	1	100.00	17.5	even	ok

Figure S75. 4-Methyl-2,6,7-triphenyl-2*H*-pyrazolo[4,3-*c*]pyridine (**24**). HRMS (ESI-TOF).

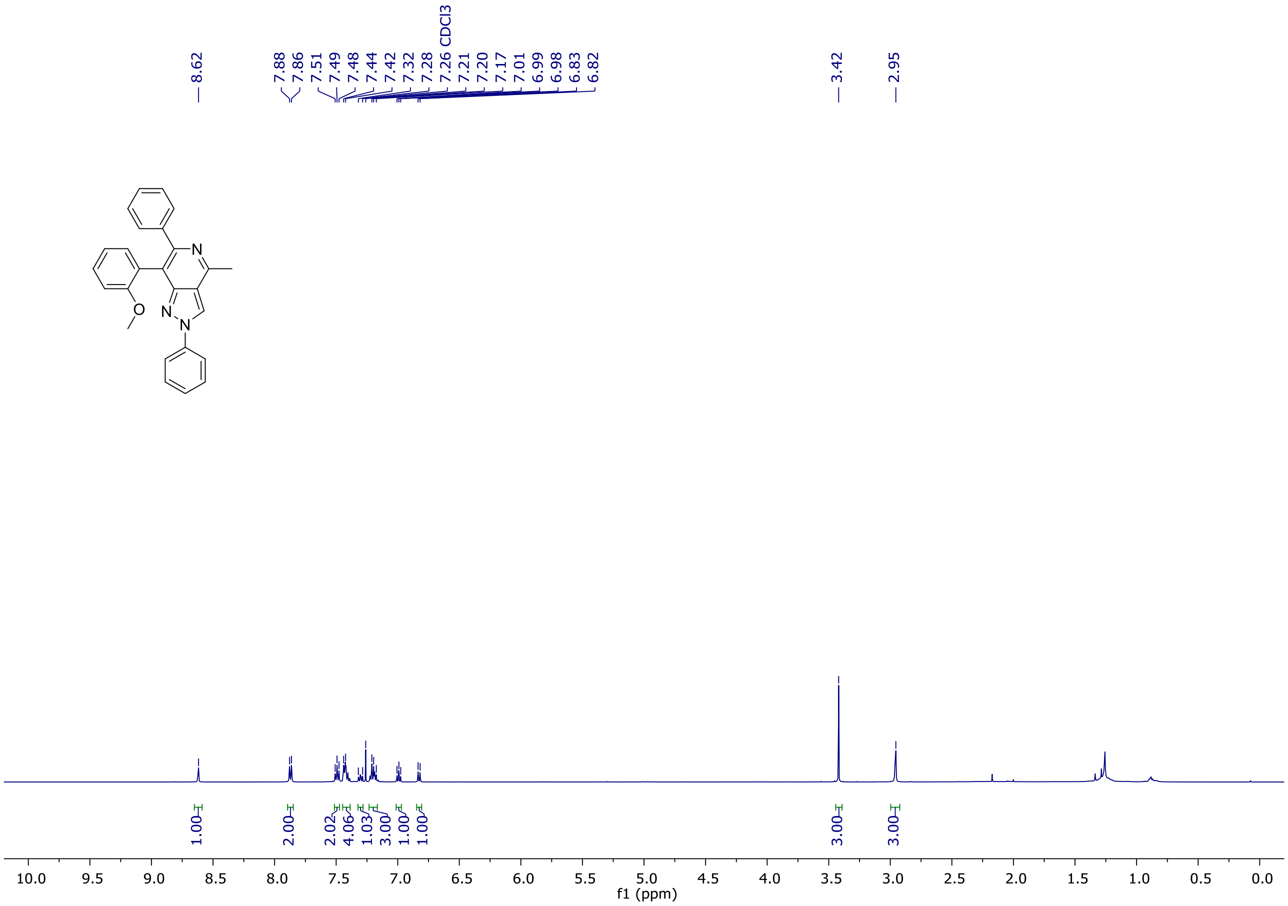

Figure S76. 7-(2-Methoxyphenyl)-4-methyl-2,6-diphenyl-2*H*-pyrazolo[4,3-*c*]pyridine (**25**). ^{1}H NMR spectrum (500 MHz, $CDCl_3$).

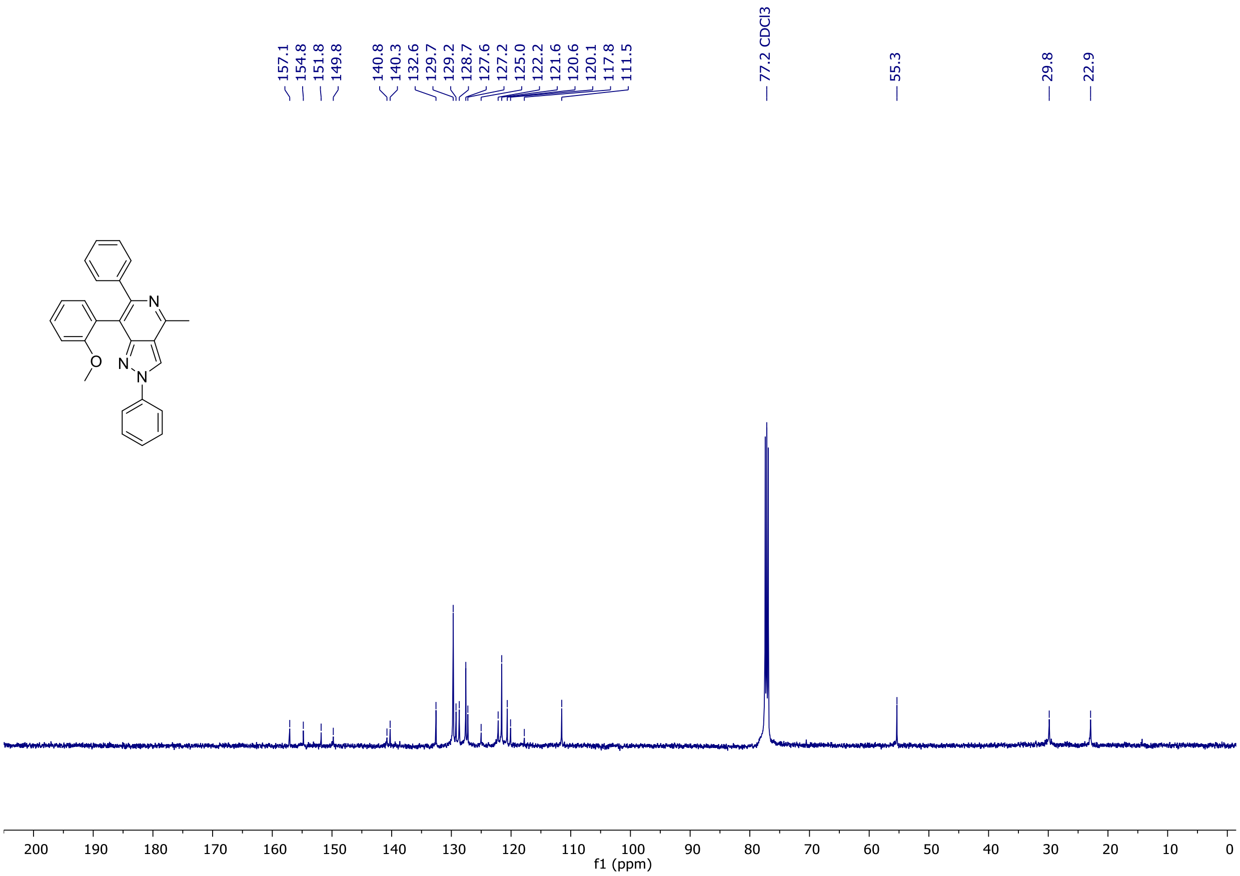

Figure S77. 7-(2-Methoxyphenyl)-4-methyl-2,6-diphenyl-2*H*-pyrazolo[4,3-*c*]pyridine (**25**). ^{13}C NMR (126 MHz, $CDCl_3$).

+MS, 4.5min #267

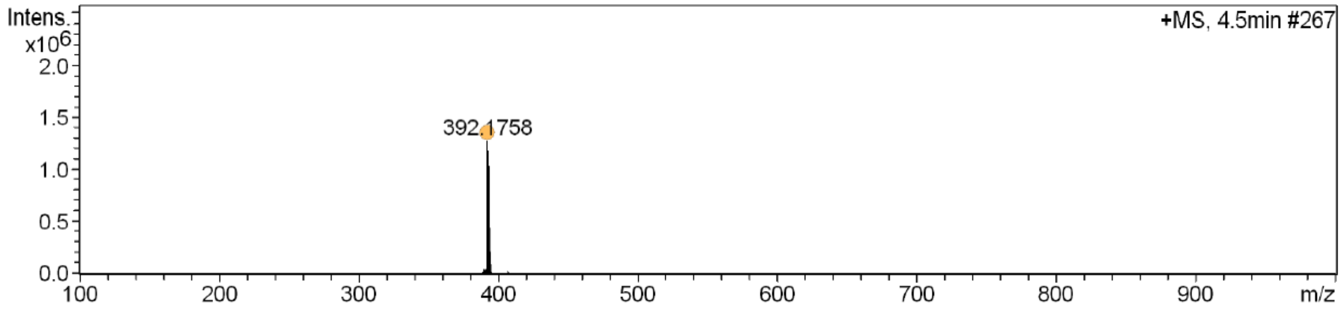

Meas. m/z	#	Ion Formula	m/z	err [ppm]	mSigma	# Sigma	Score	rdb	e¯ Conf	N-Rule
392.1758	1	C26H22N3O	392.1757	0.2	325.3	1	100.00	17.5	even	ok

Figure S78. 7-(2-Methoxyphenyl)-4-methyl-2,6-diphenyl-2*H*-pyrazolo[4,3-*c*]pyridine (**25**). HRMS (ESI-TOF).

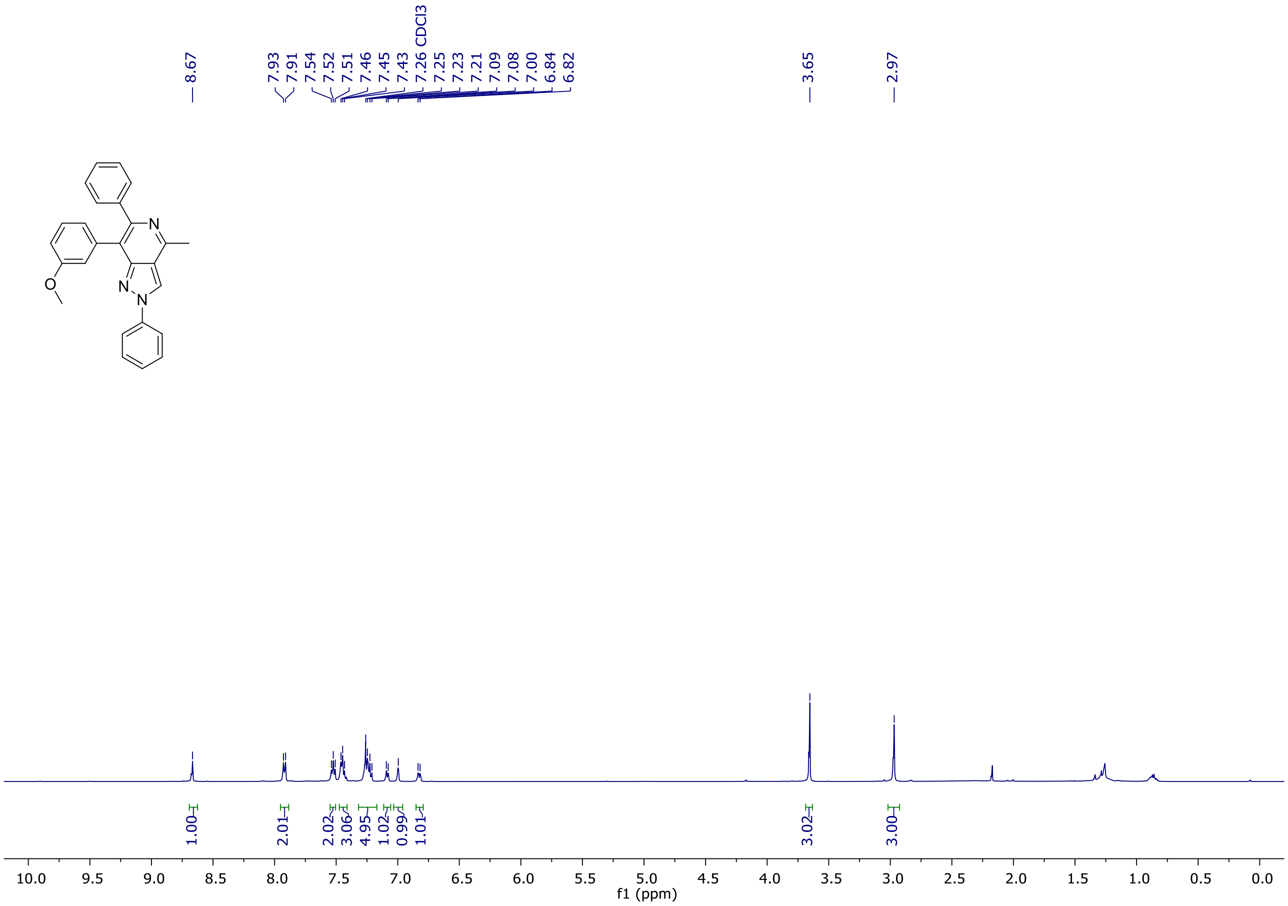

Figure S79. 7-(3-Methoxyphenyl)-4-methyl-2,6-diphenyl-2*H*-pyrazolo[4,3-*c*]pyridine (**26**). ^{1}H NMR spectrum (500 MHz, $CDCl_3$).

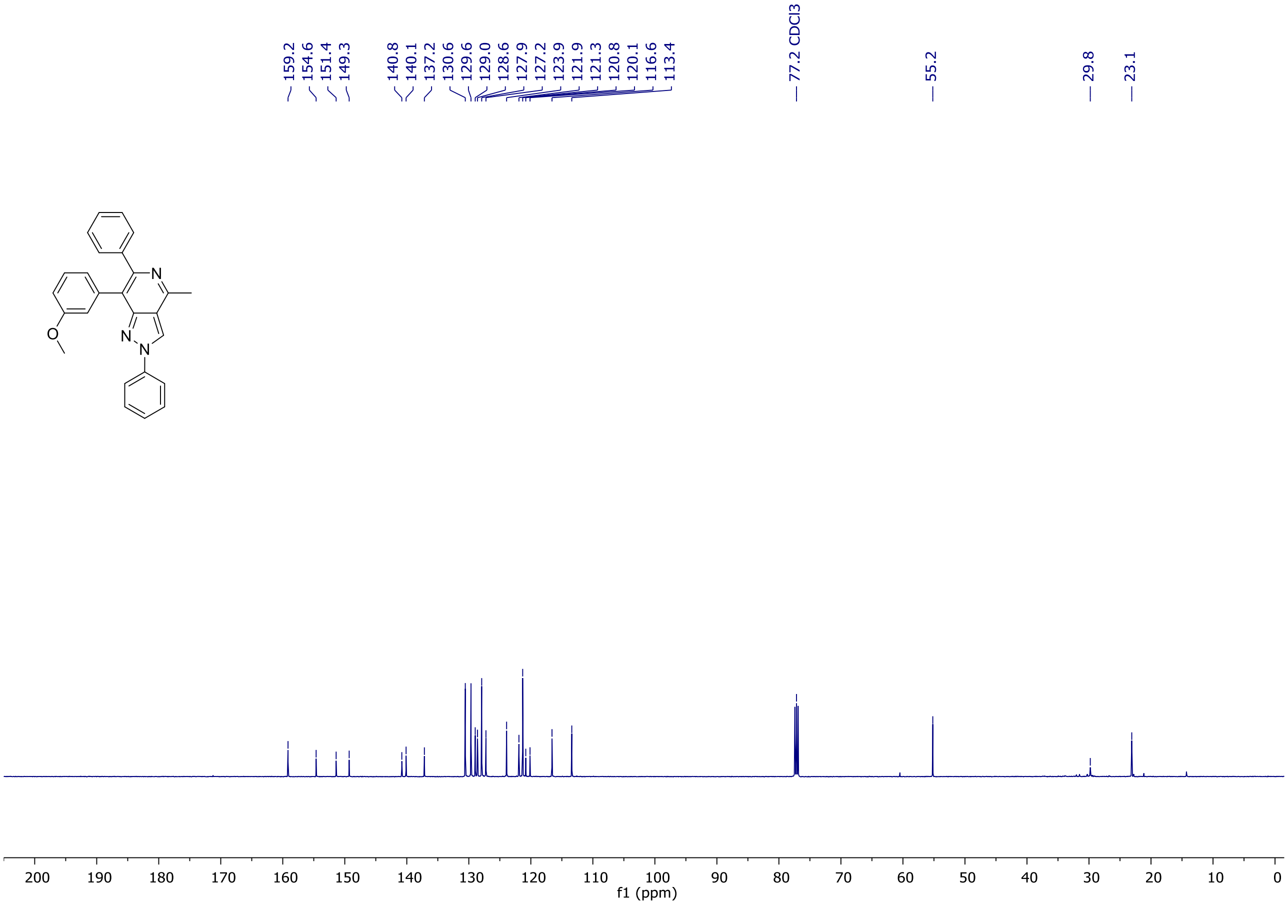

Figure S80. 7-(3-Methoxyphenyl)-4-methyl-2,6-diphenyl-2*H*-pyrazolo[4,3-*c*]pyridine (**26**). ^{13}C NMR (126 MHz, $CDCl_3$).

+MS, 5.8min #348

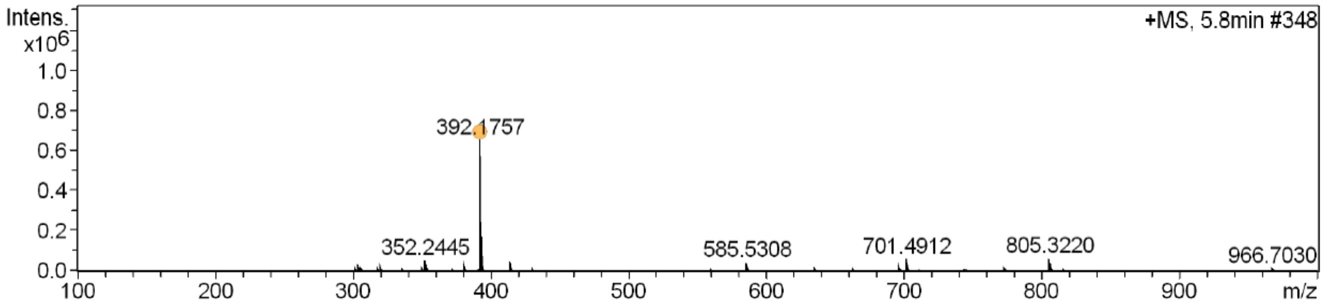

Meas. m/z	#	Ion Formula	m/z	err [ppm]	mSigma	# Sigma	Score	rdb	e⁻ Conf	N-Rule
392.1757	1	C26H22N3O	392.1757	0.2	4.9	1	100.00	17.5	even	ok

Figure S81. 7-(3-Methoxyphenyl)-4-methyl-2,6-diphenyl-2*H*-pyrazolo[4,3-*c*]pyridine (**26**). HRMS (ESI-TOF).

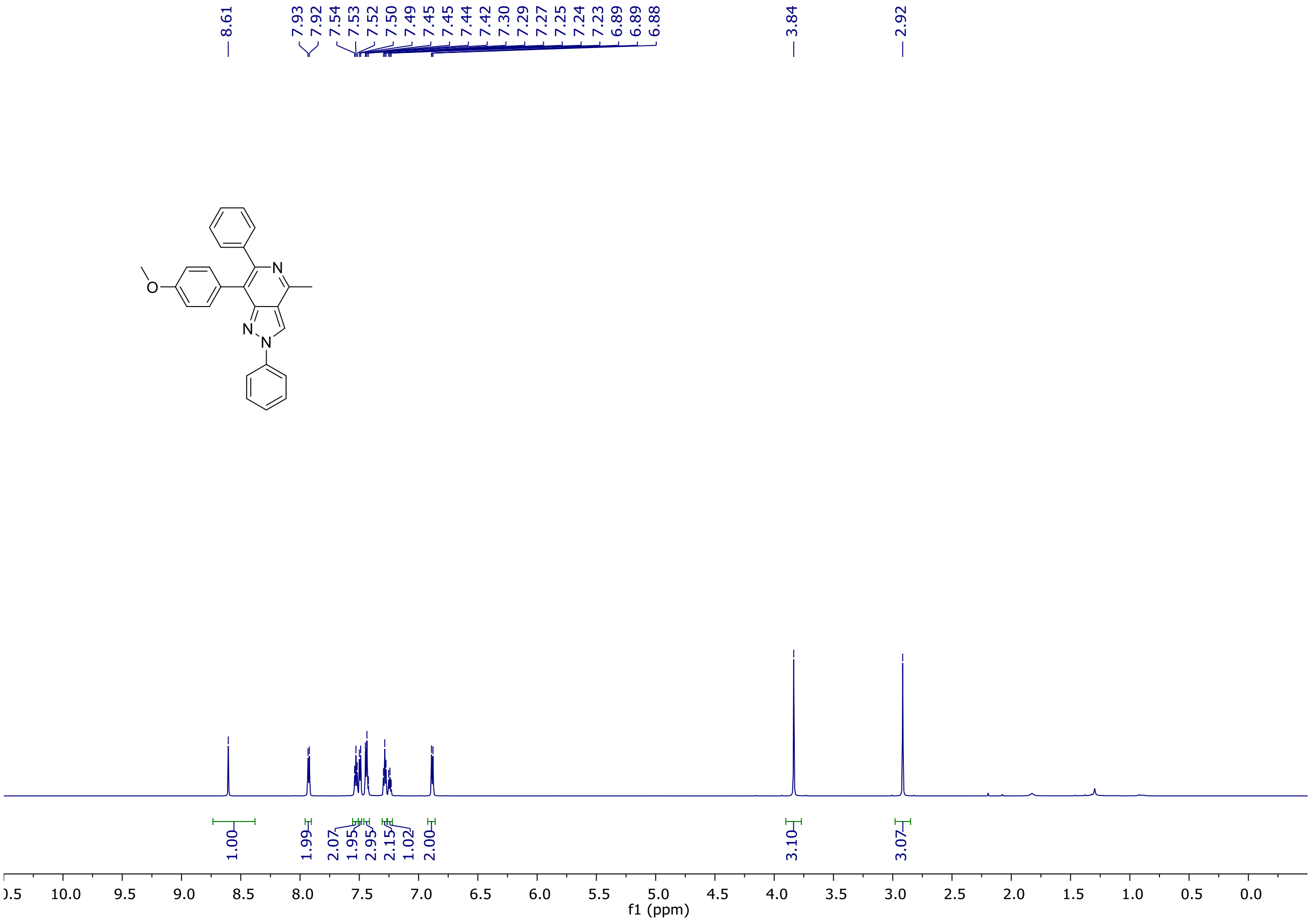

Figure S82. 7-(4-Methoxyphenyl)-4-methyl-2,6-diphenyl-2*H*-pyrazolo[4,3-*c*]pyridine (**27**). ^{1}H NMR spectrum (700 MHz, $CDCl_3$).

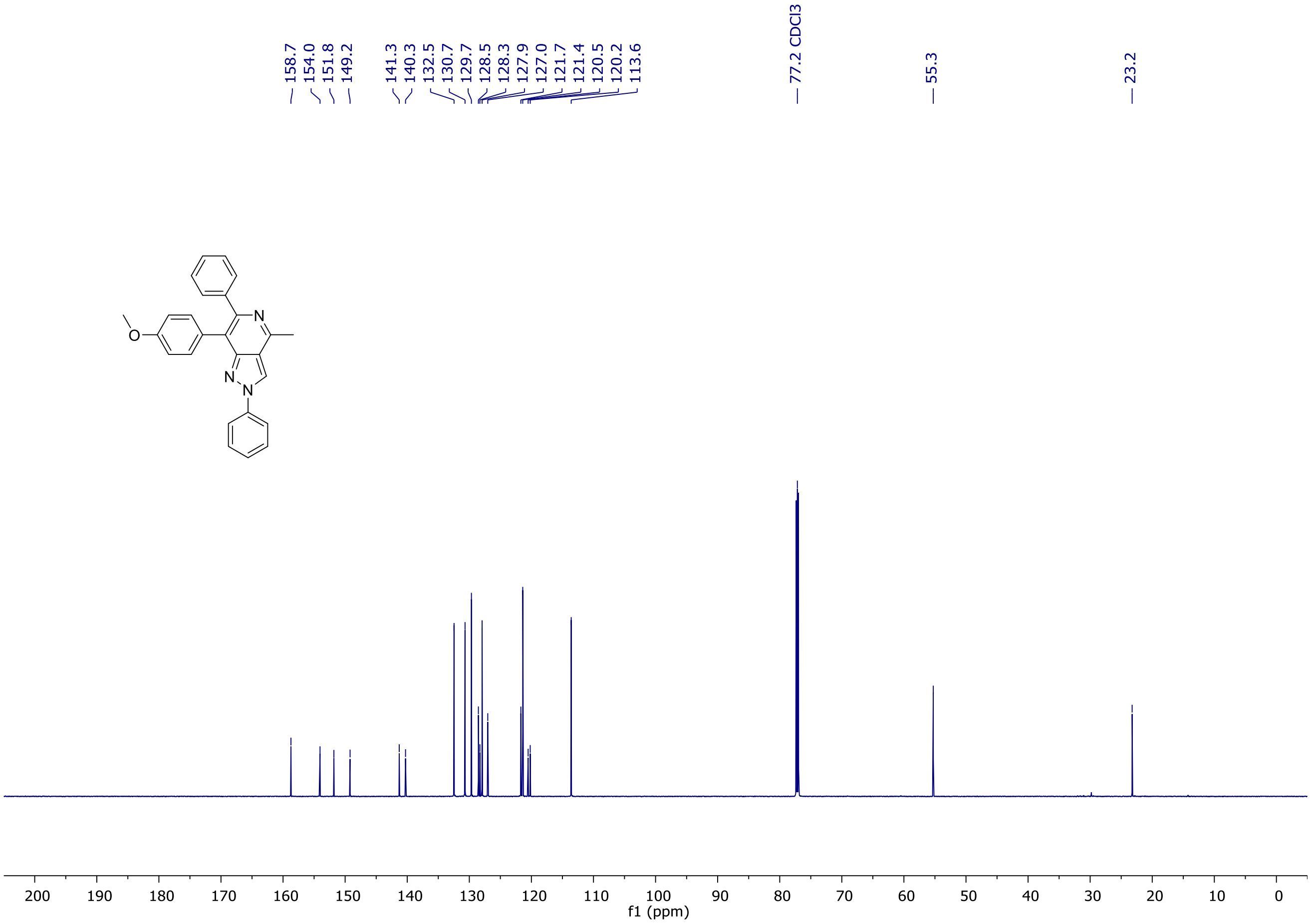

Figure S83. 7-(4-Methoxyphenyl)-4-methyl-2,6-diphenyl-2*H*-pyrazolo[4,3-*c*]pyridine (**27**). ^{13}C NMR (176 MHz, $CDCl_3$).

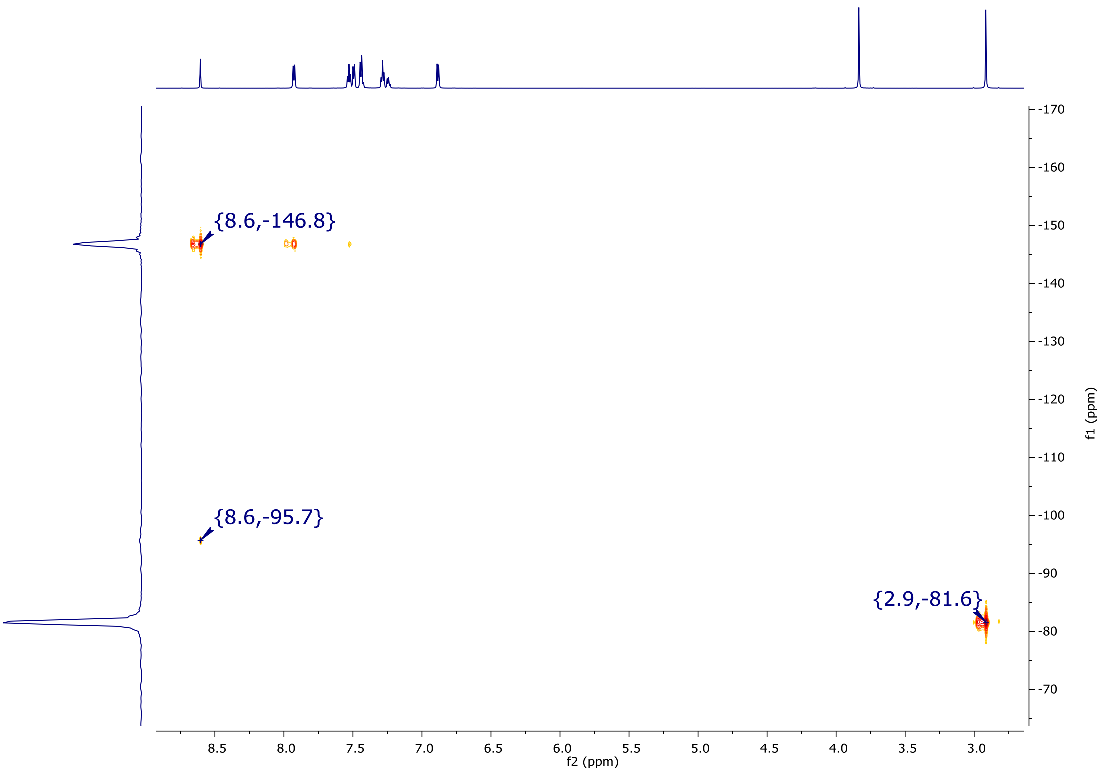

Figure S84. 7-(4-Methoxyphenyl)-4-methyl-2,6-diphenyl-2*H*-pyrazolo[4,3-*c*]pyridine (**27**). ^{1}H-^{15}N HMBC spectrum (700 MHz, $CDCl_3$).

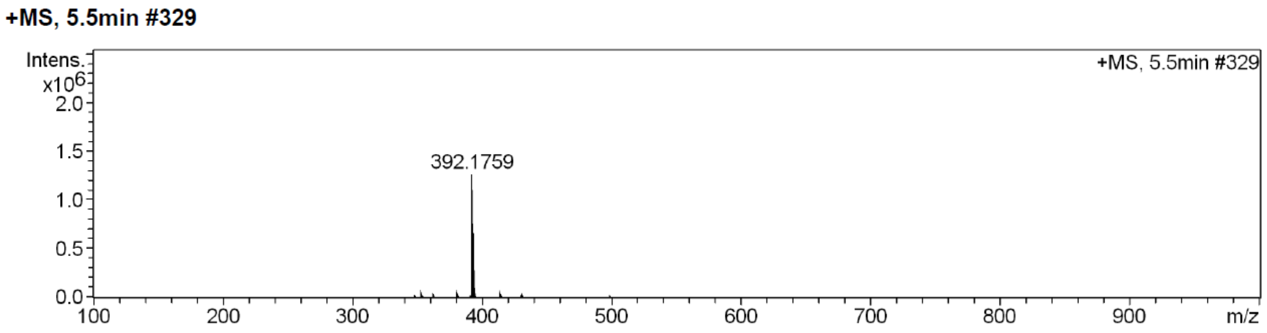

Meas. m/z	#	Ion Formula	m/z	err [ppm]	mSigma	# Sigma	Score	rdb	e⁻ Conf	N-Rule
392.1759	1	C26H22N3O	392.1757	0.4	155.5	1	100.00	17.5	even	ok

Figure S85. 7-(4-Methoxyphenyl)-4-methyl-2,6-diphenyl-2*H*-pyrazolo[4,3-*c*]pyridine (**27**). HRMS (ESI-TOF).

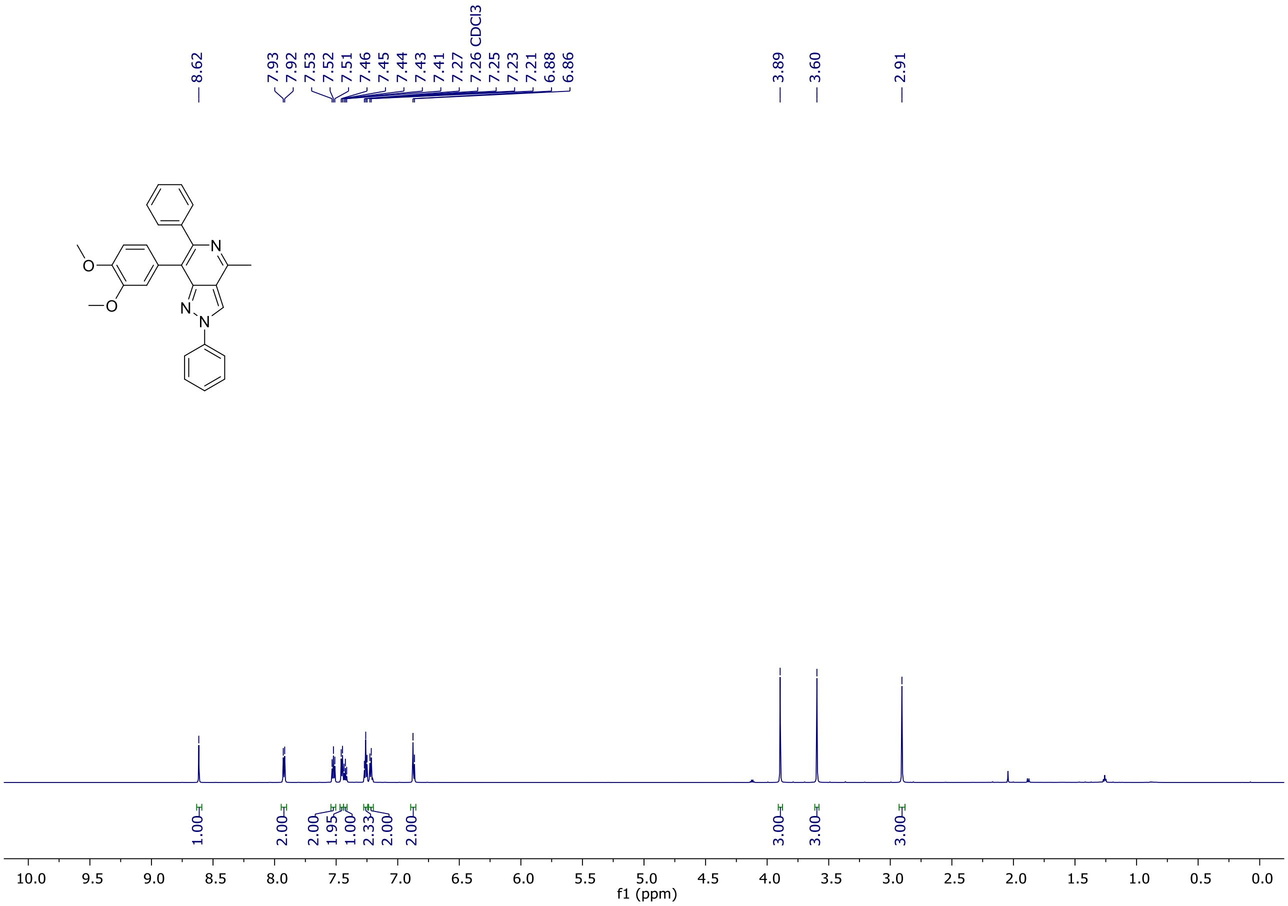

Figure S86. 7-(3,4-Dimethoxyphenyl)-4-methyl-2,6-diphenyl-2*H*-pyrazolo[4,3-*c*]pyridine (**28**). ^{1}H NMR spectrum (700 MHz, $CDCl_3$).

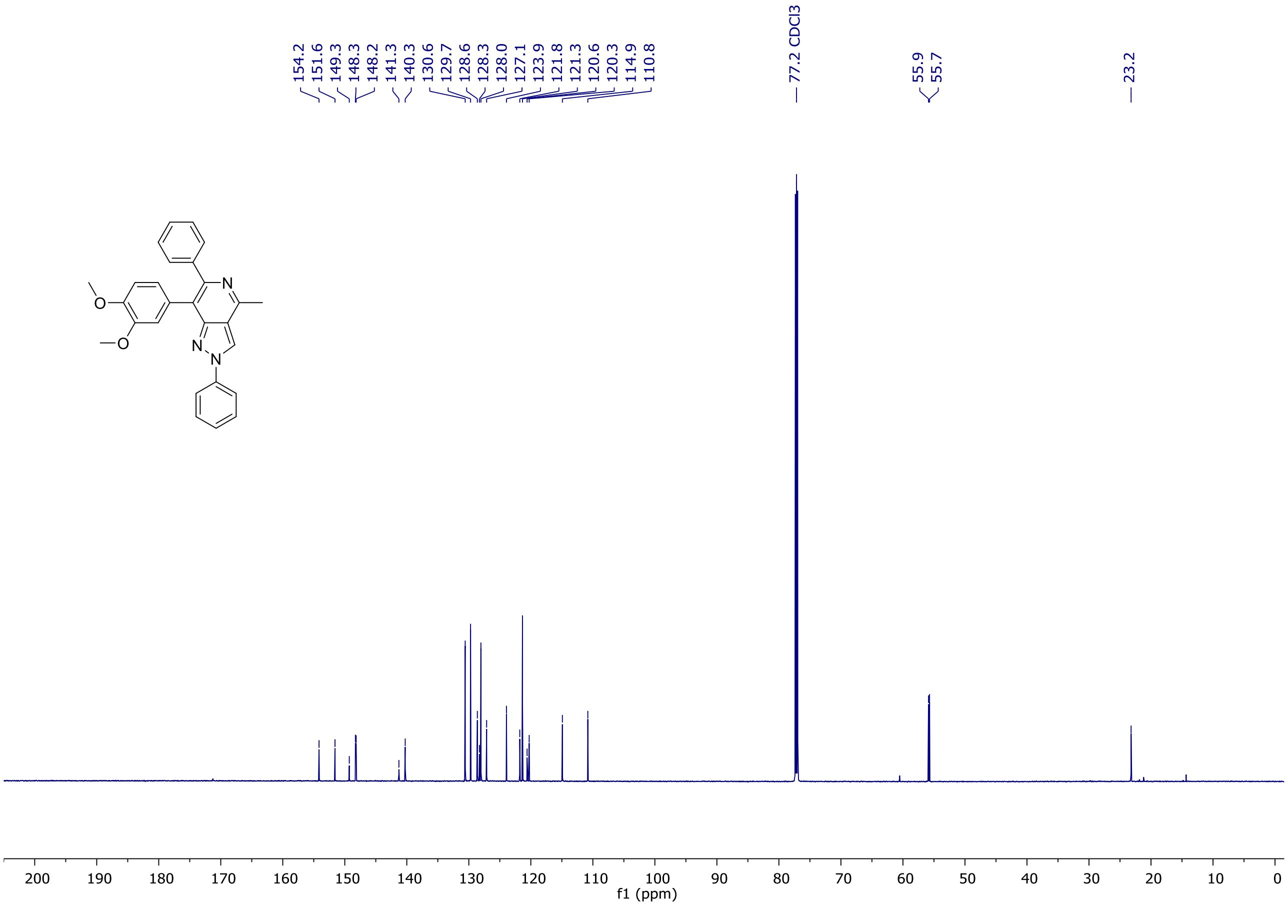

Figure S87. 7-(3,4-Dimethoxyphenyl)-4-methyl-2,6-diphenyl-2*H*-pyrazolo[4,3-*c*]pyridine (**28**). ^{13}C NMR (176 MHz, $CDCl_3$).

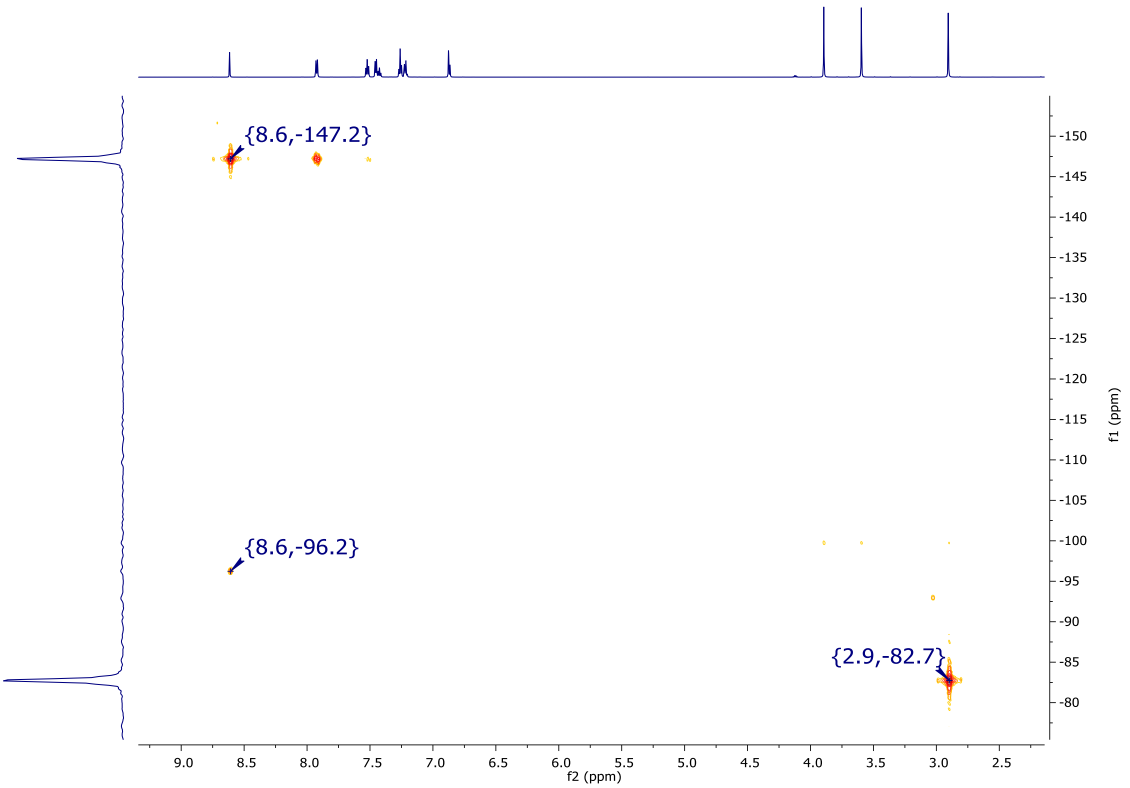

Figure S88. 7-(3,4-Dimethoxyphenyl)-4-methyl-2,6-diphenyl-2*H*-pyrazolo[4,3-*c*]pyridine (**28**). ^{1}H-^{15}N HMBC spectrum (700 MHz, $CDCl_3$).

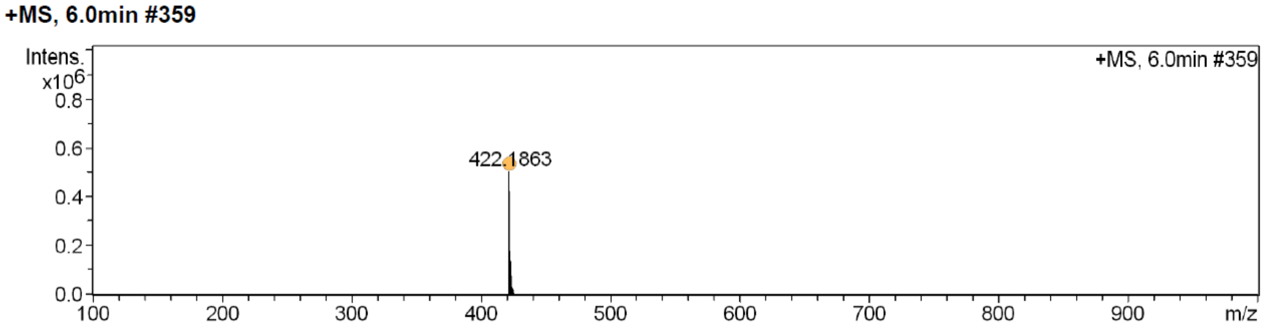

Meas. m/z	#	Ion Formula	m/z	err [ppm]	mSigma	# Sigma	Score	rdb	e⁻ Conf	N-Rule
422.1863	1	C27H24N3O2	422.1863	-0.1	2.3	1	100.00	17.5	even	ok

Figure S89. 7-(3,4-Dimethoxyphenyl)-4-methyl-2,6-diphenyl-2*H*-pyrazolo[4,3-*c*]pyridine (**28**). HRMS (ESI-TOF).

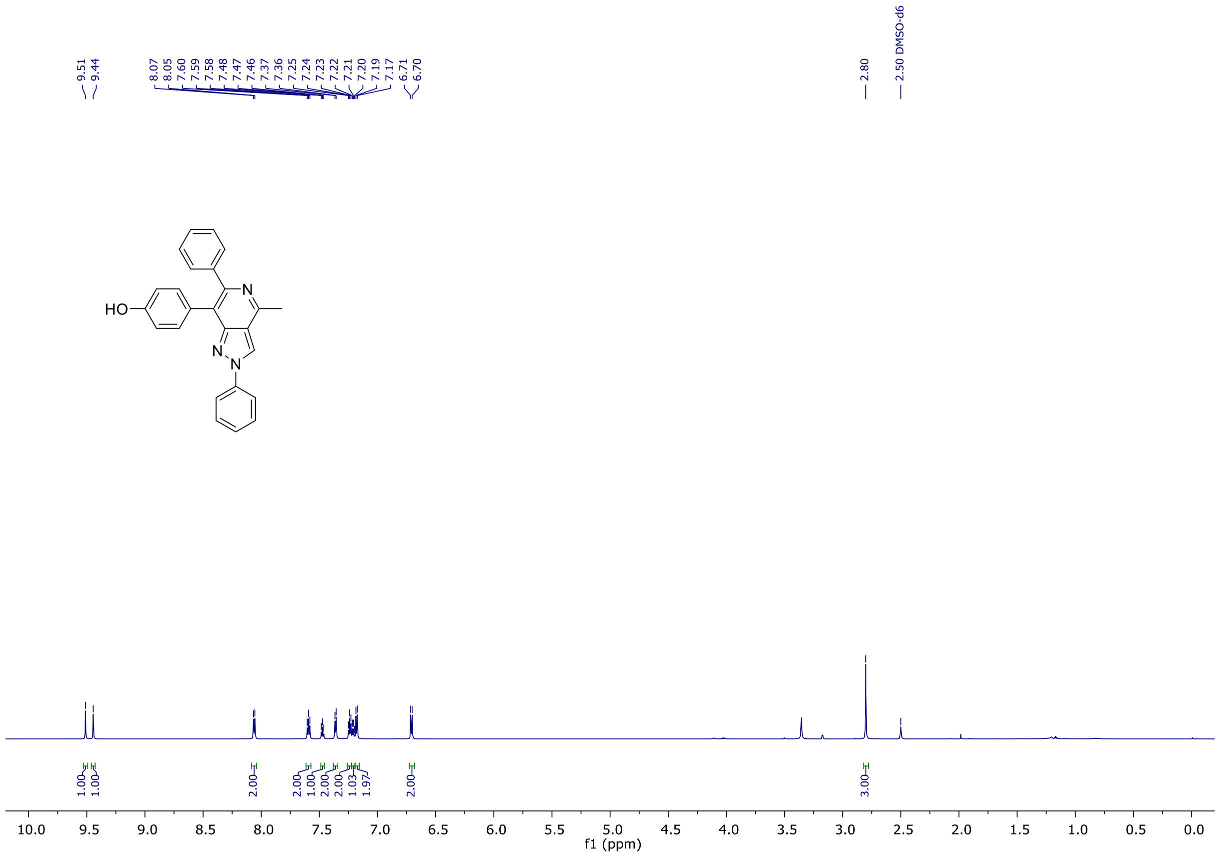

Figure S90. 4-(4-Methyl-2,6-diphenyl-2*H*-pyrazolo[4,3-*c*]pyridin-7-yl)phenol (**29**). ^{1}H NMR spectrum (700 MHz, DMSO-d_6).

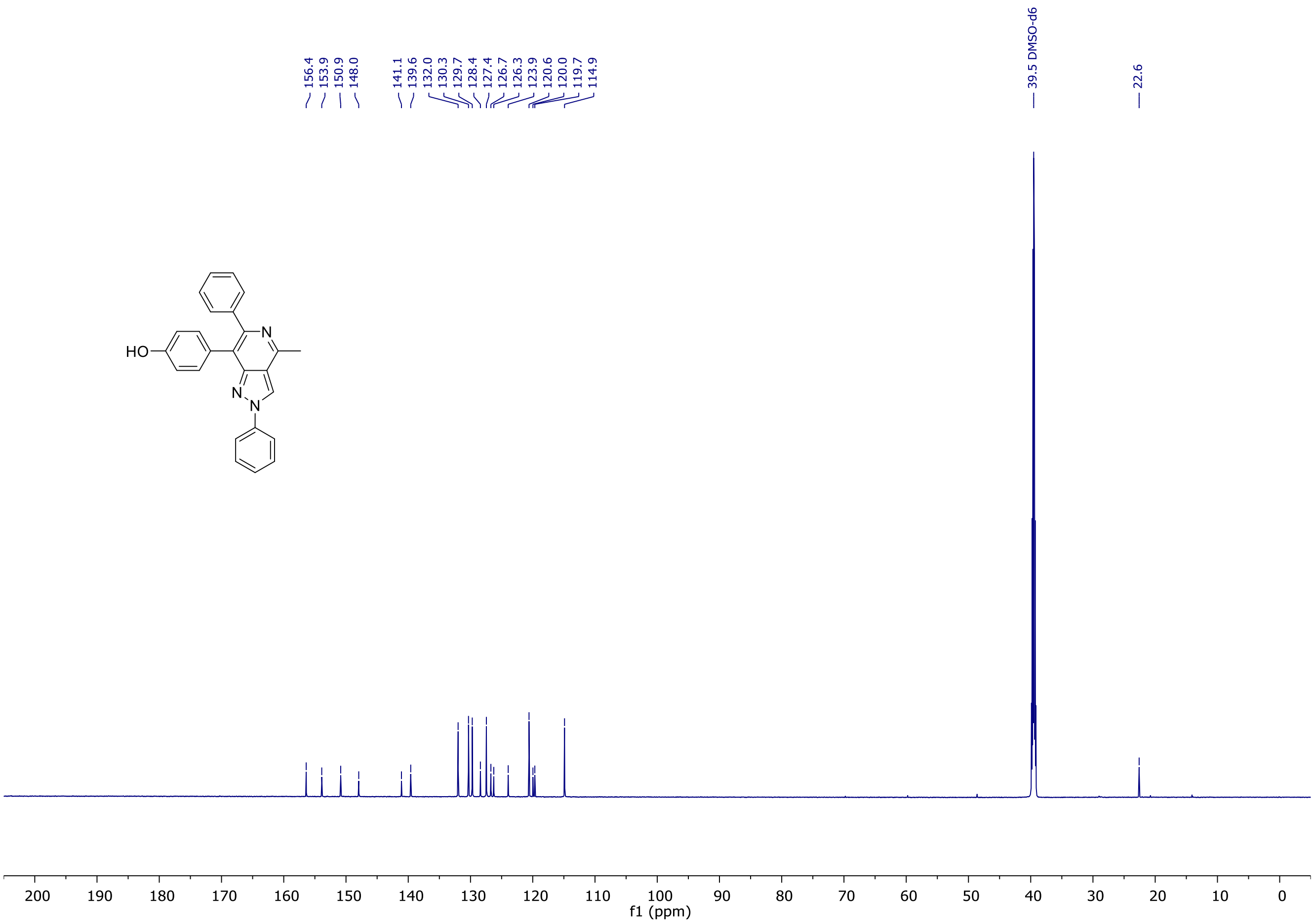

Figure S91. 4-(4-Methyl-2,6-diphenyl-2*H*-pyrazolo[4,3-*c*]pyridin-7-yl)phenol (**29**). ^{13}C NMR (176 MHz, DMSO-d_6).

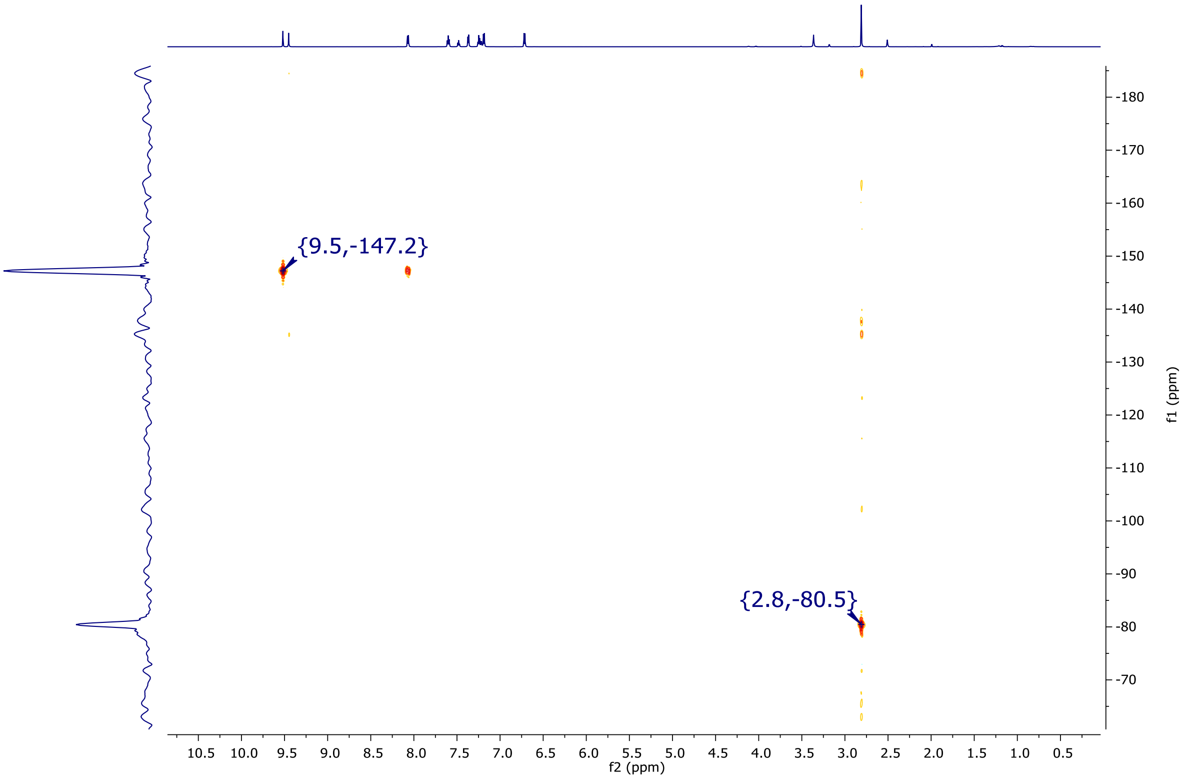

Figure S92. 4-(4-Methyl-2,6-diphenyl-2*H*-pyrazolo[4,3-*c*]pyridin-7-yl)phenol (**29**). ^{1}H-^{15}N HMBC spectrum (700 MHz, DMSO-d_6).

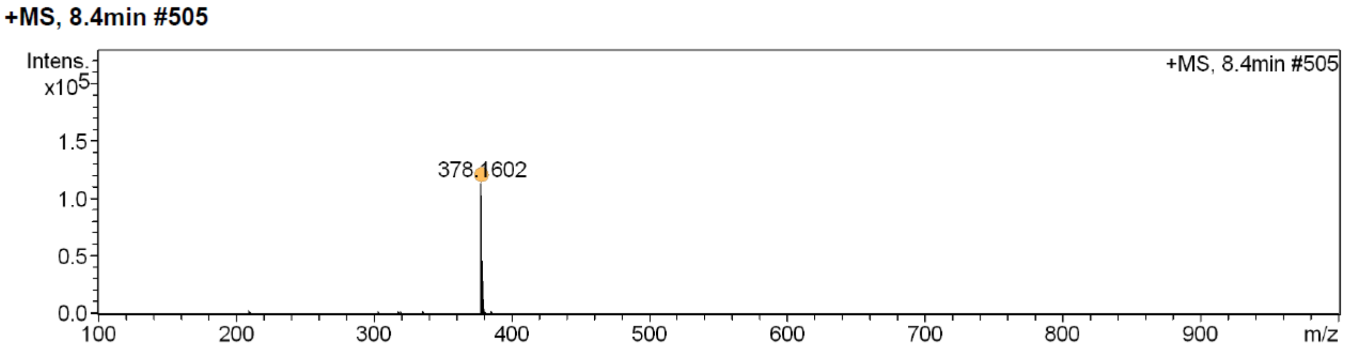

Meas. m/z	#	Ion Formula	m/z	err [ppm]	mSigma	# Sigma	Score	rdb	e⁻ Conf	N-Rule
378.1602	1	C25H20N3O	378.1601	-0.4	1.7	1	100.00	17.5	even	ok

Figure S93. 4-(4-Methyl-2,6-diphenyl-2*H*-pyrazolo[4,3-*c*]pyridin-7-yl)phenol (**29**). HRMS (ESI-TOF).

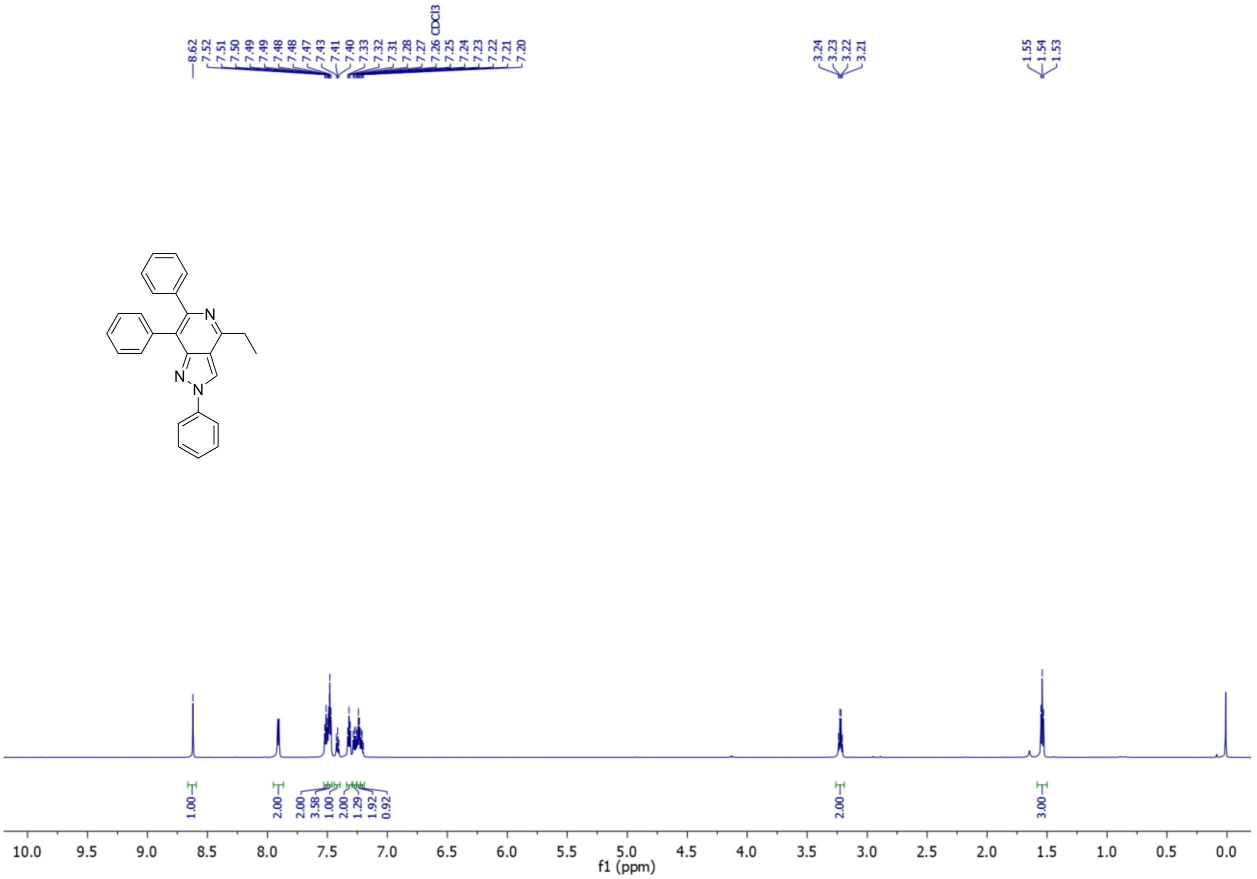

Figure S94. 4-Ethyl-2,6,7-triphenyl-2*H*-pyrazolo[4,3-*c*]pyridine (**30**). ^{1}H NMR spectrum (700 MHz, $CDCl_3$).

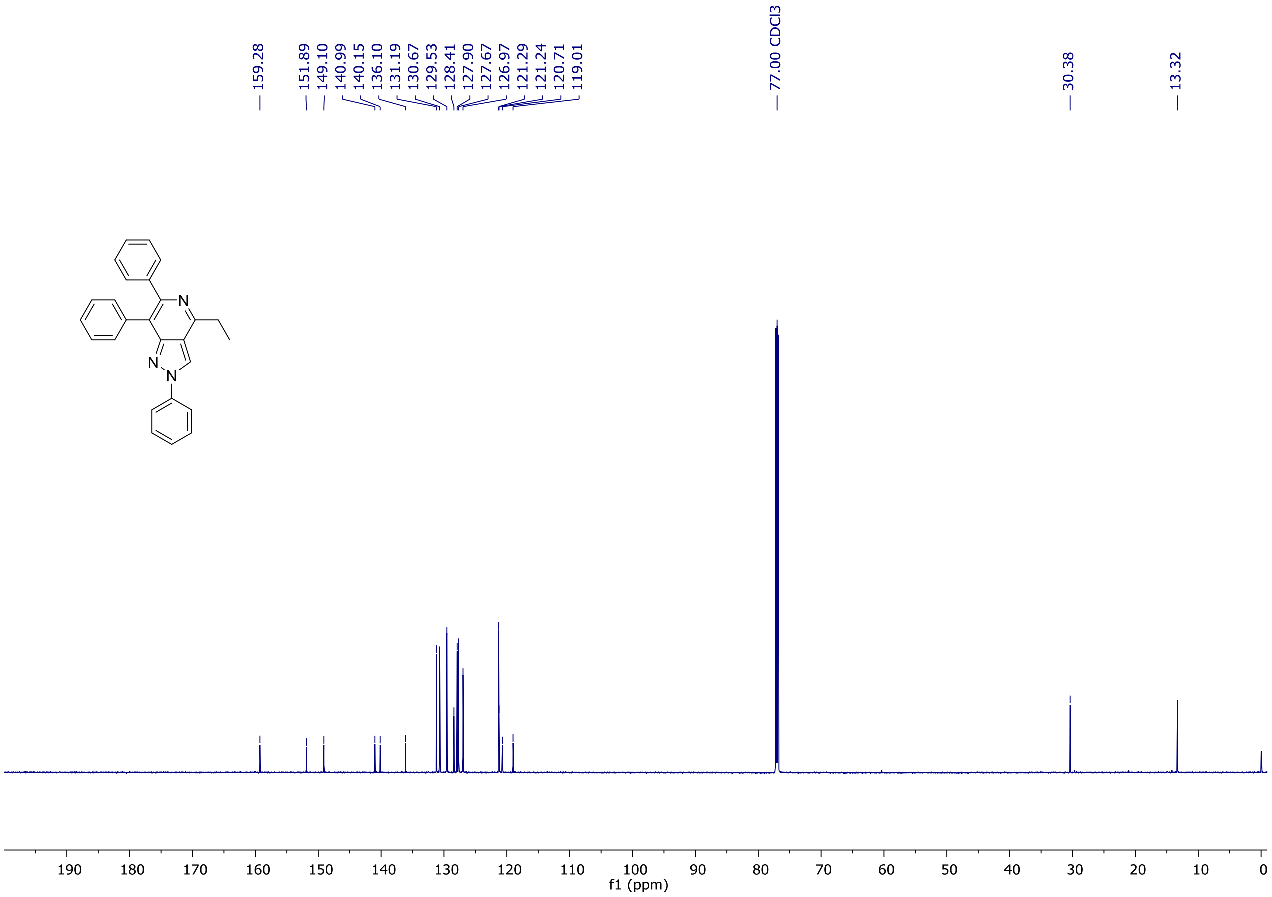

Figure S95. 4-Ethyl-2,6,7-triphenyl-2*H*-pyrazolo[4,3-*c*]pyridine (**30**). ^{13}C NMR (176 MHz, $CDCl_3$).

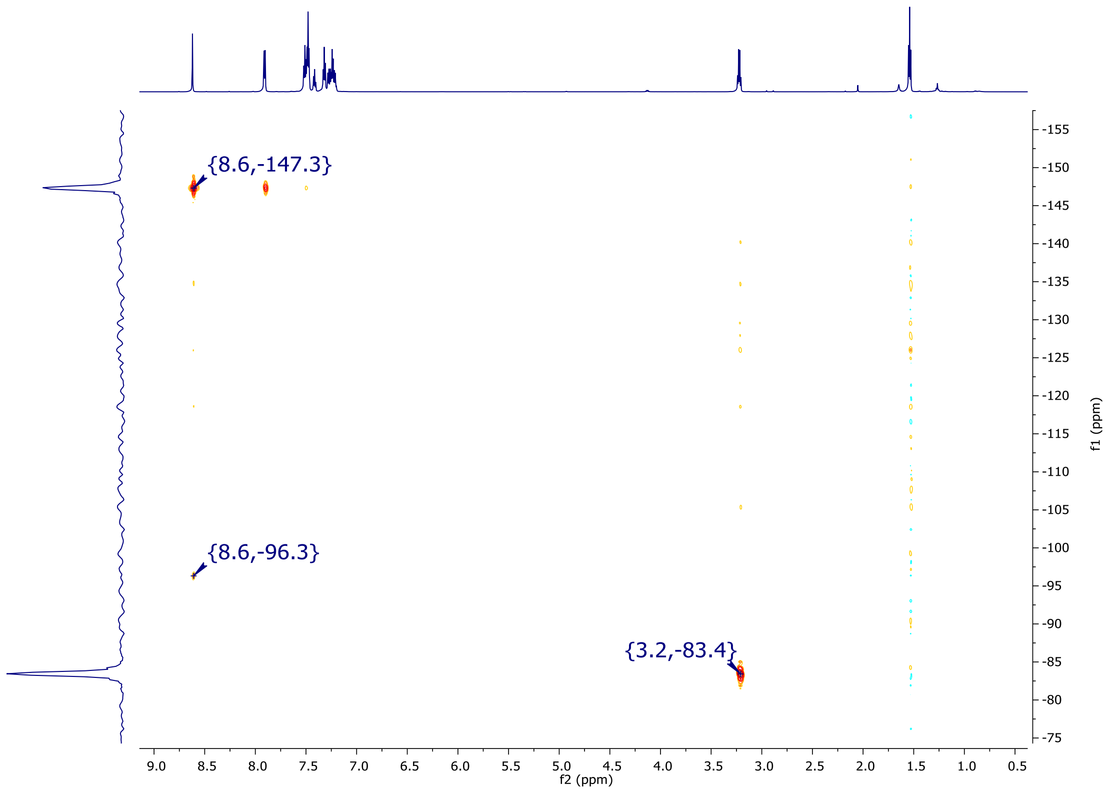

Figure S96. 4-Ethyl-2,6,7-triphenyl-2*H*-pyrazolo[4,3-*c*]pyridine (**30**). ^{1}H-^{15}N HMBC spectrum (700 MHz, $CDCl_3$).

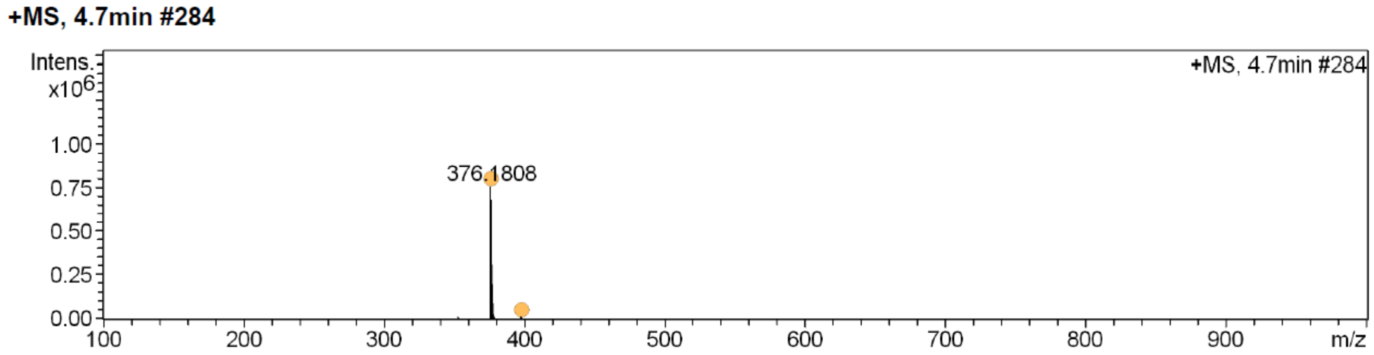

Meas. m/z	#	Ion Formula	m/z	err [ppm]	mSigma	# Sigma	Score	rdb	e¯ Conf	N-Rule
376.1808	1	C26H22N3	376.1808	0.1	2.5	1	100.00	17.5	even	ok
398.1611	1	C26H21N3Na	398.1628	4.2	33.1	1	100.00	17.5	even	ok

Figure S97. 4-Ethyl-2,6,7-triphenyl-2*H*-pyrazolo[4,3-*c*]pyridine (**30**). HRMS (ESI-TOF).

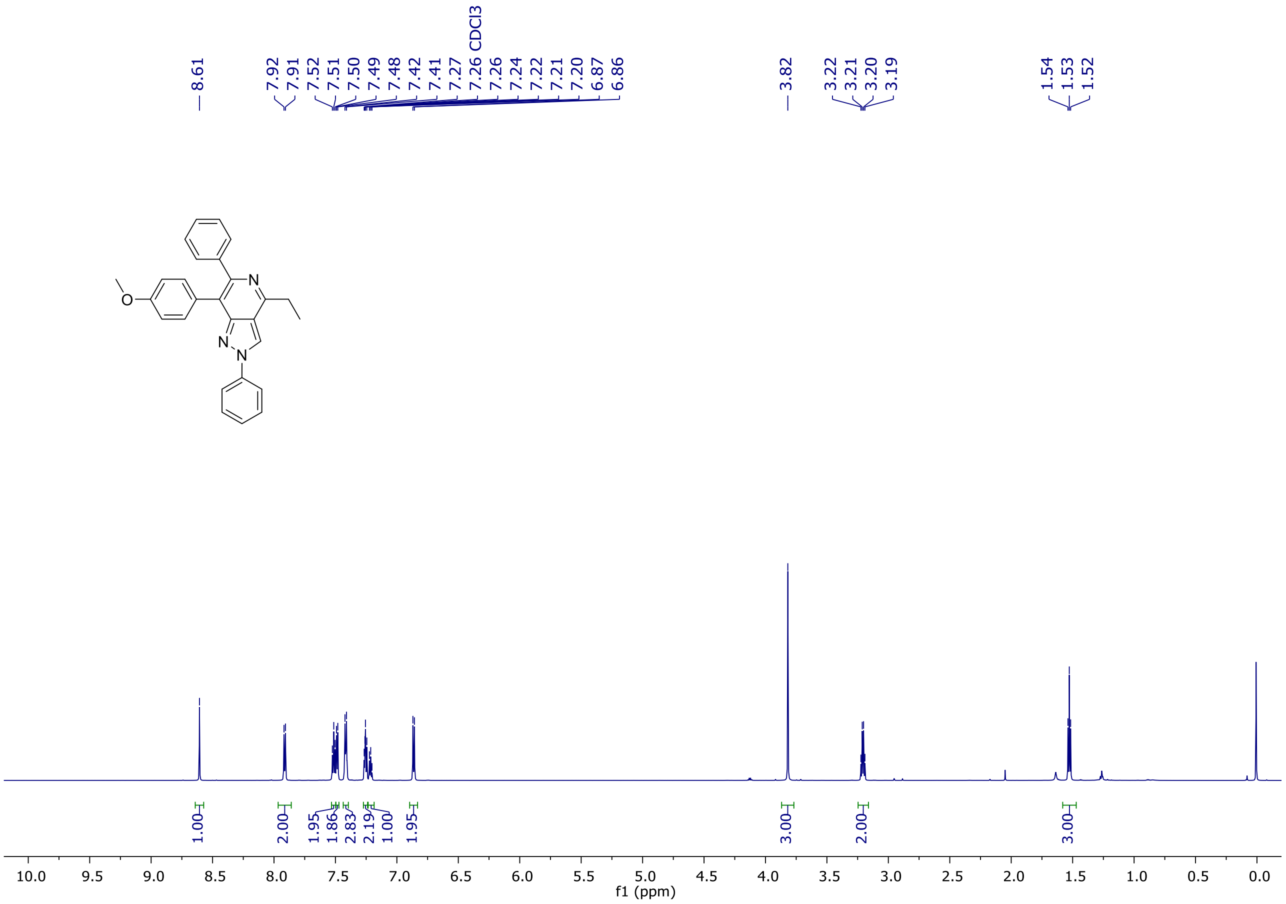

Figure S98. 4-Ethyl-7-(4-methoxyphenyl)-2,6-diphenyl-2*H*-pyrazolo[4,3-*c*]pyridine (**31**). ^{1}H NMR spectrum (700 MHz, $CDCl_3$).

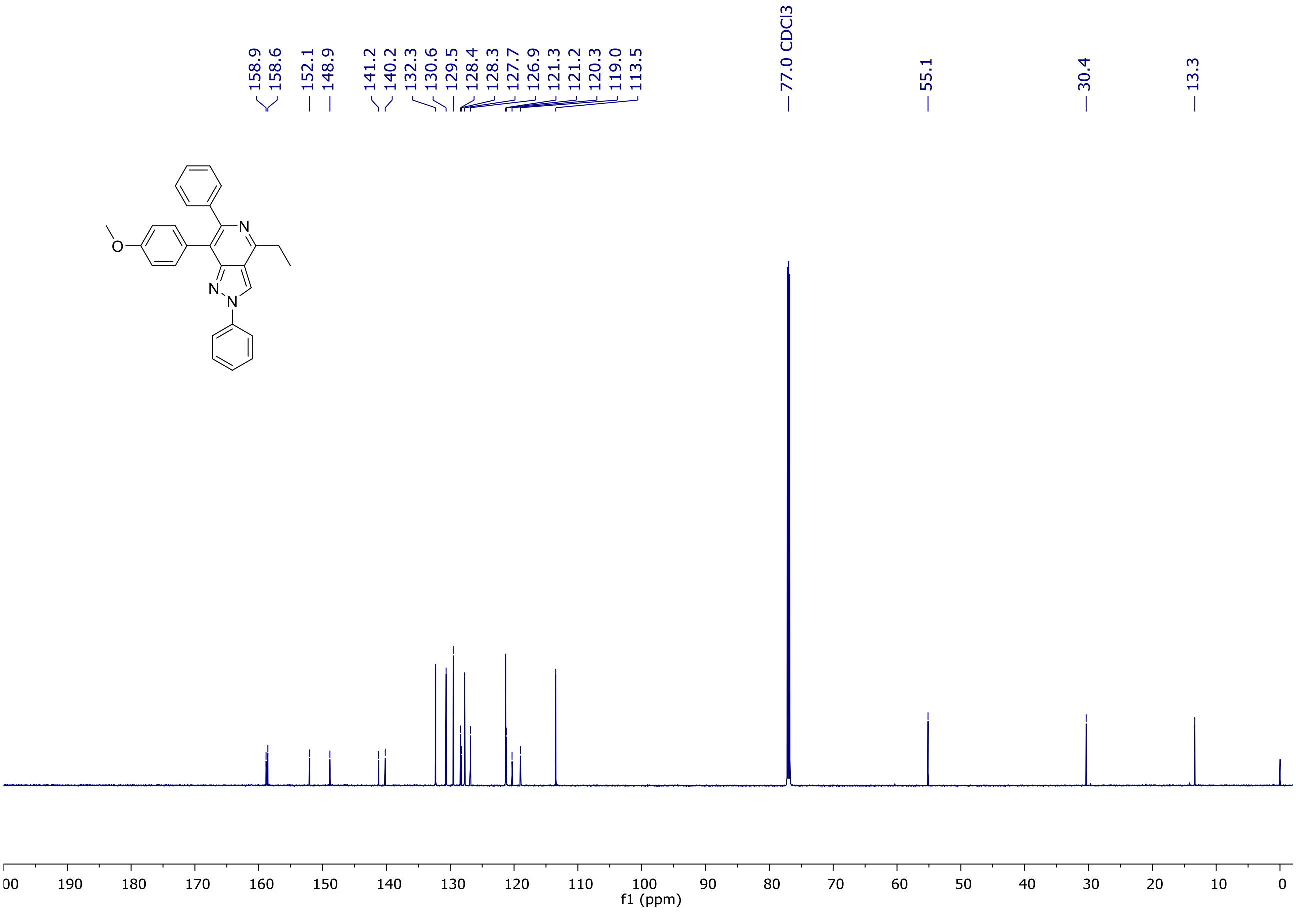

Figure S99. 4-Ethyl-7-(4-methoxyphenyl)-2,6-diphenyl-2*H*-pyrazolo[4,3-*c*]pyridine (**31**). ^{13}C NMR (176 MHz, $CDCl_3$).

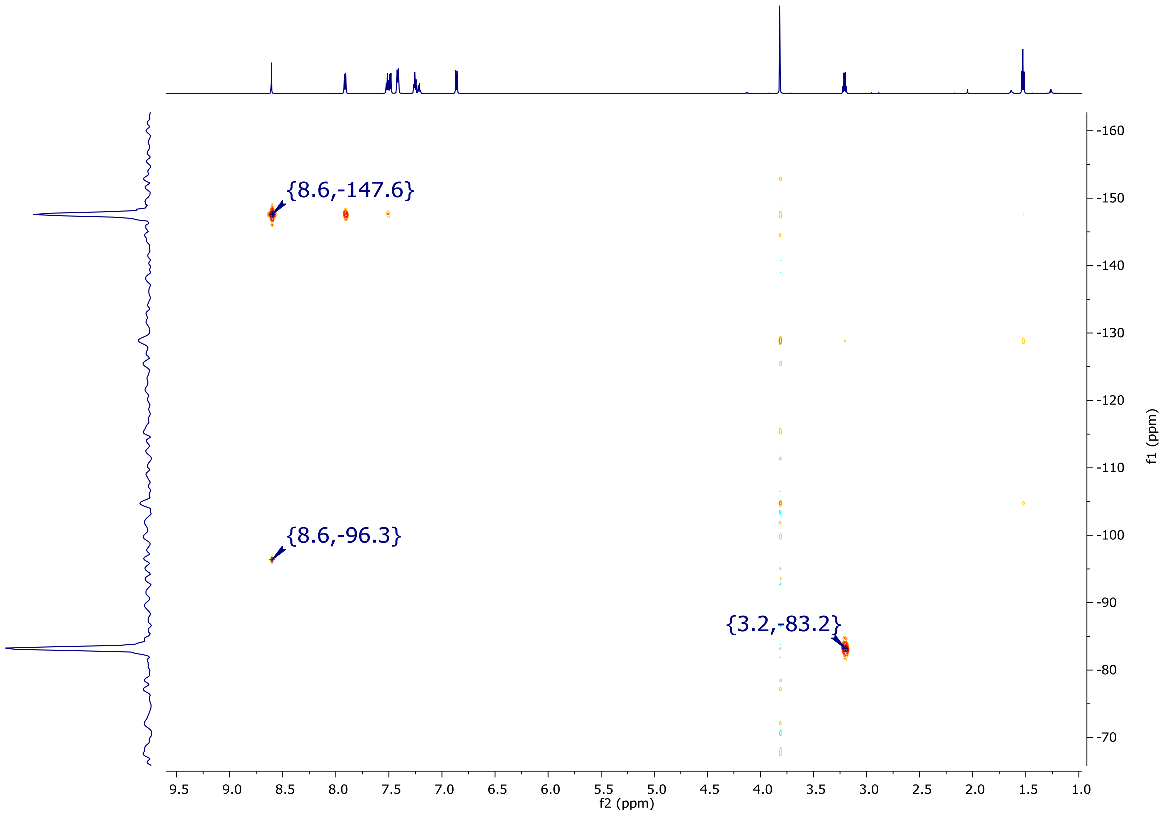

Figure S100. 4-Ethyl-7-(4-methoxyphenyl)-2,6-diphenyl-2*H*-pyrazolo[4,3-*c*]pyridine (**31**). ^{1}H-^{15}N HMBC spectrum (700 MHz, $CDCl_3$).

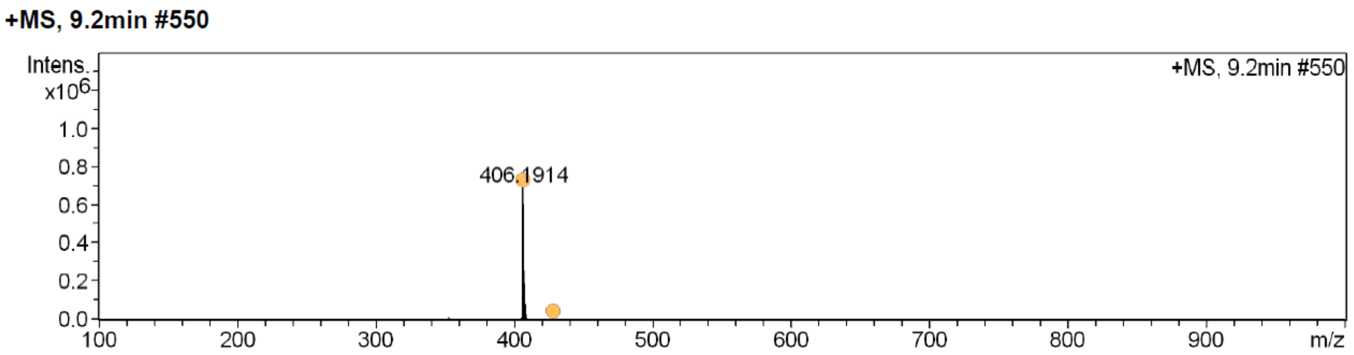

Meas. m/z	#	Ion Formula	m/z	err [ppm]	mSigma	# Sigma	Score	rdb	e⁻ Conf	N-Rule
406.1914	1	C27H24N3O	406.1914	-0.0	2.1	1	100.00	17.5	even	ok
428.1745	1	C27H23N3NaO	428.1733	-2.8	25.2	1	100.00	17.5	even	ok

Figure S101. 4-Ethyl-7-(4-methoxyphenyl)-2,6-diphenyl-2*H*-pyrazolo[4,3-*c*]pyridine (**31**). HRMS (ESI-TOF).

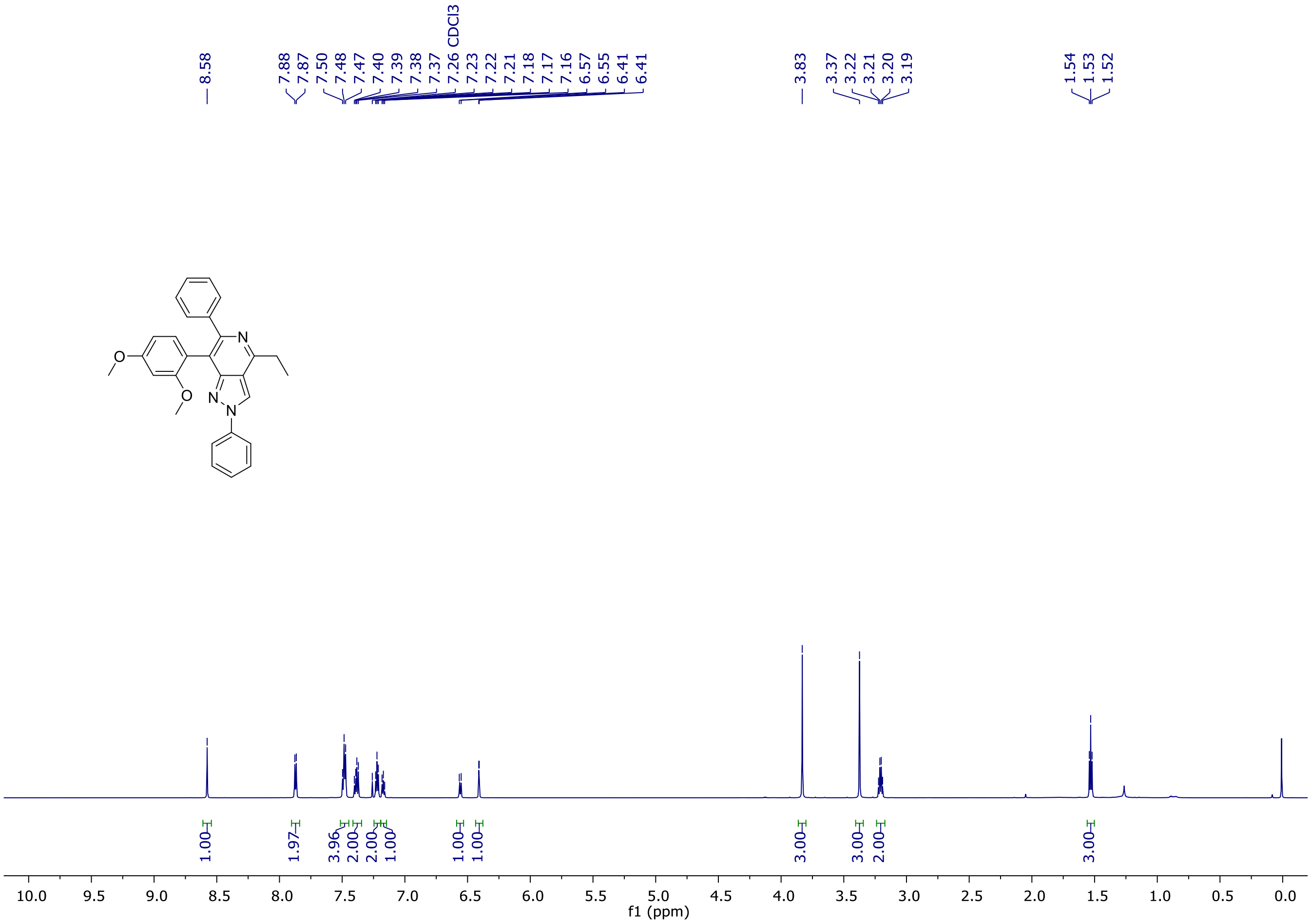

Figure S102. 7-(2,4-Dimethoxyphenyl)-4-ethyl-2,6-diphenyl-2*H*-pyrazolo[4,3-*c*]pyridine (**32**). ^{1}H NMR spectrum (700 MHz, $CDCl_3$).

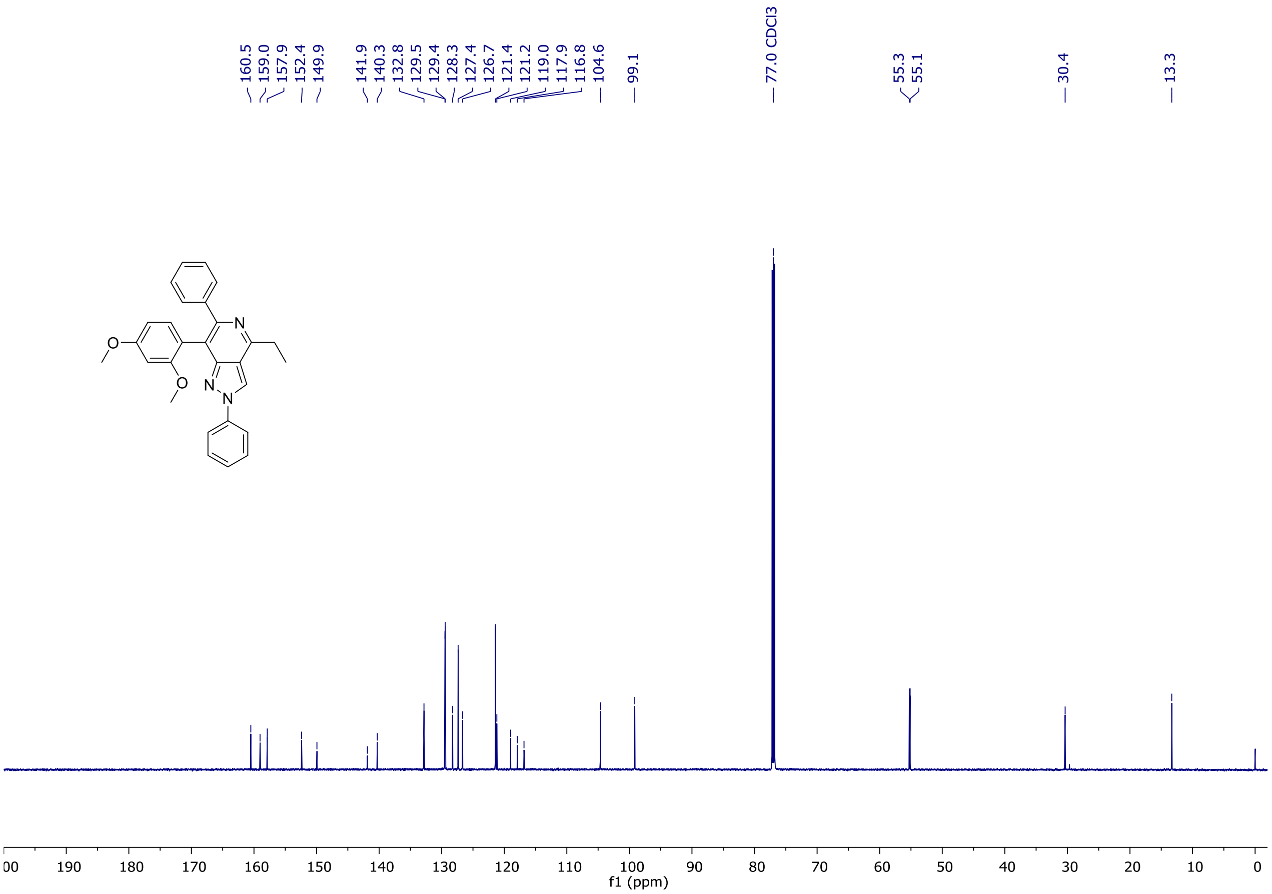

Figure S103. 7-(2,4-Dimethoxyphenyl)-4-ethyl-2,6-diphenyl-2*H*-pyrazolo[4,3-*c*]pyridine (**32**). ^{13}C NMR (176 MHz, $CDCl_3$).

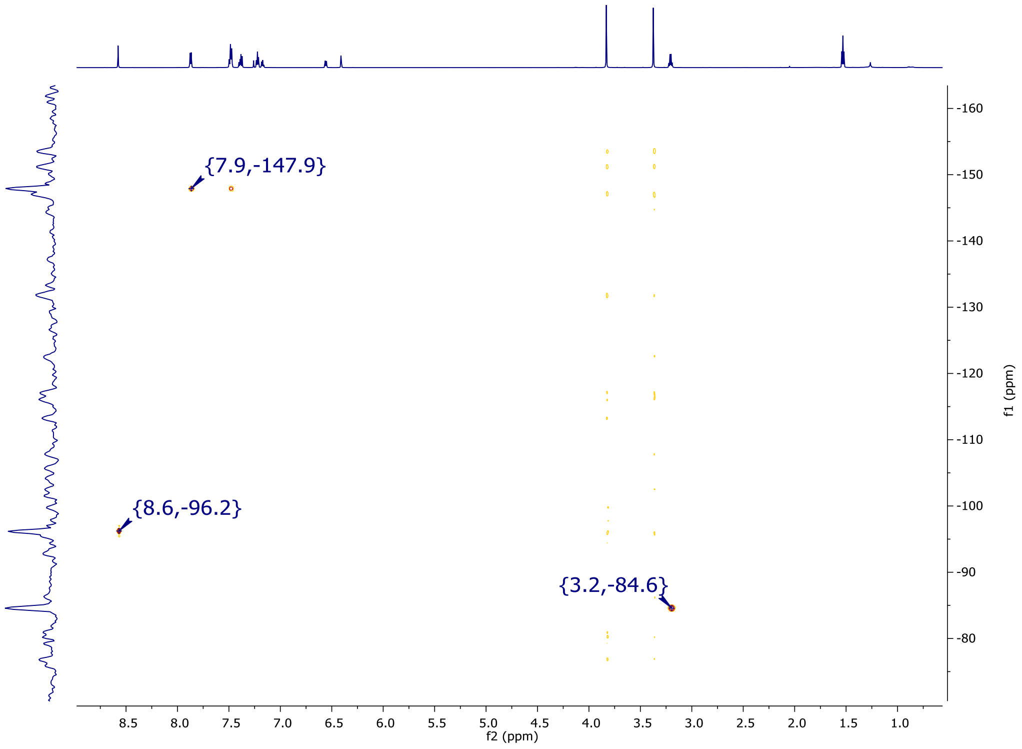

Figure S104. 7-(2,4-Dimethoxyphenyl)-4-ethyl-2,6-diphenyl-2*H*-pyrazolo[4,3-*c*]pyridine (**32**). ^{1}H-^{15}N LR HSQMBC spectrum (700 MHz, $CDCl_3$).

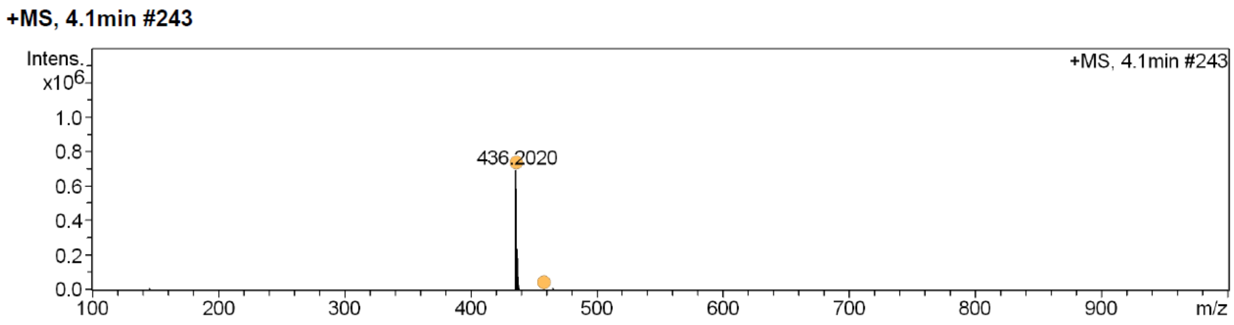

Meas. m/z	#	Ion Formula	m/z	err [ppm]	mSigma	# Sigma	Score	rdb	e⁻ Conf	N-Rule
436.2020	1	C28H26N3O2	436.2020	-0.2	2.7	1	100.00	17.5	even	ok
458.1873	1	C28H25N3NaO2	458.1839	7.5	98.4	1	100.00	17.5	even	ok

Figure S105. 7-(2,4-Dimethoxyphenyl)-4-ethyl-2,6-diphenyl-2*H*-pyrazolo[4,3-*c*]pyridine (**32**). HRMS (ESI-TOF).

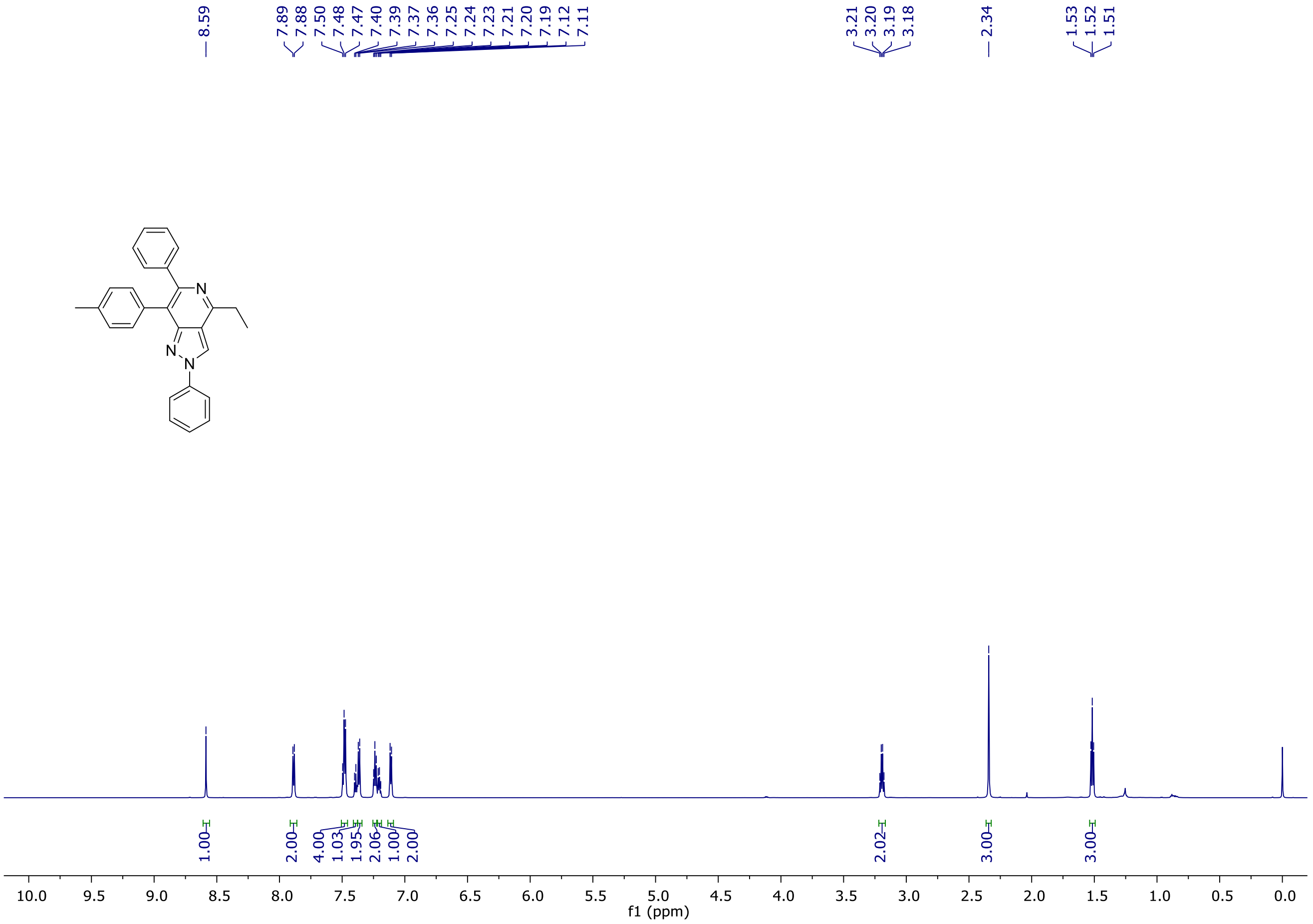

Figure S106. 4-Ethyl-2,6-diphenyl-7-(*p*-tolyl)-2*H*-pyrazolo[4,3-*c*]pyridine (**33**). ^{1}H NMR spectrum (700 MHz, $CDCl_3$).

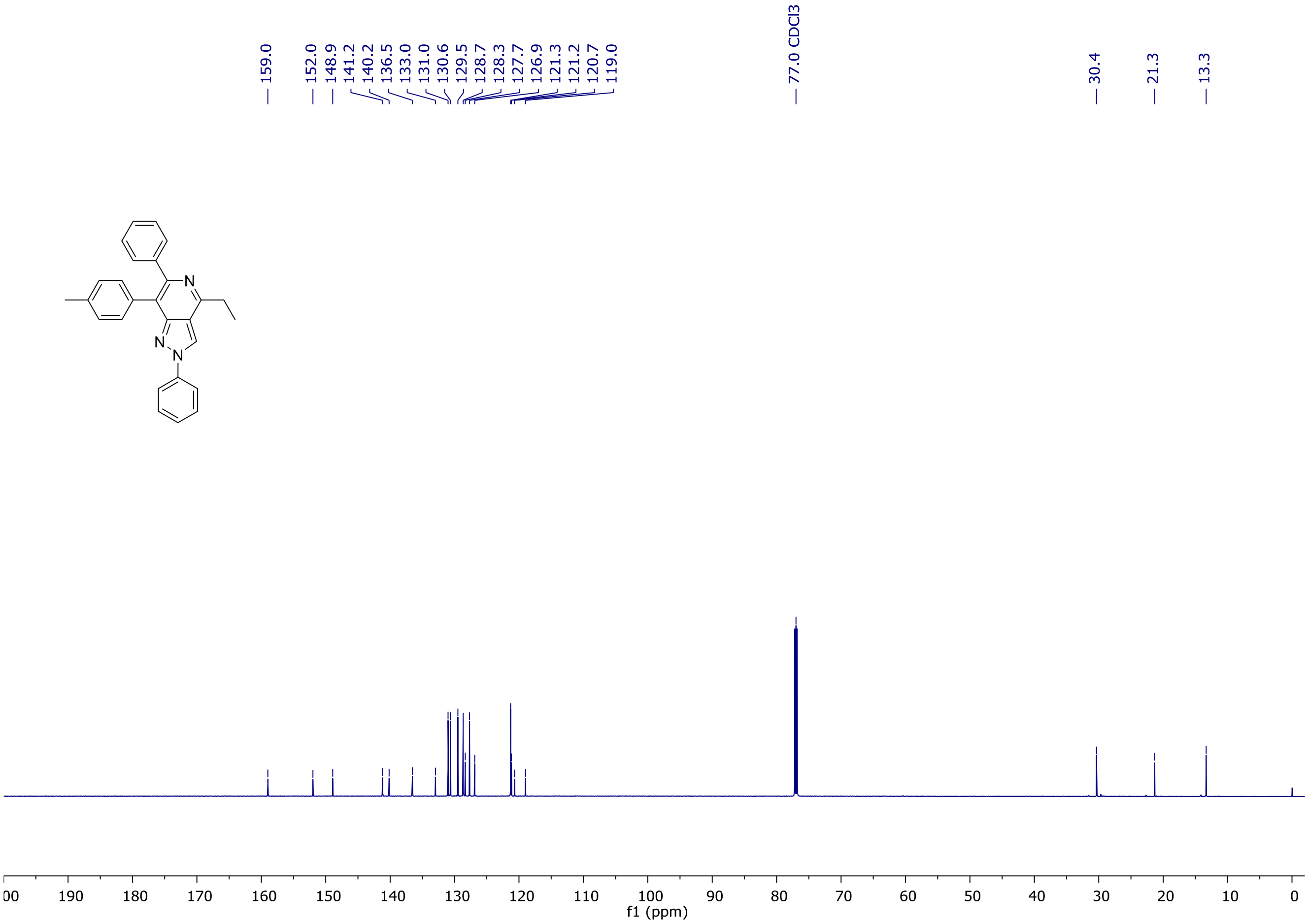

Figure S107. 4-Ethyl-2,6-diphenyl-7-(*p*-tolyl)-2*H*-pyrazolo[4,3-*c*]pyridine (**33**). ^{13}C NMR (176 MHz, $CDCl_3$).

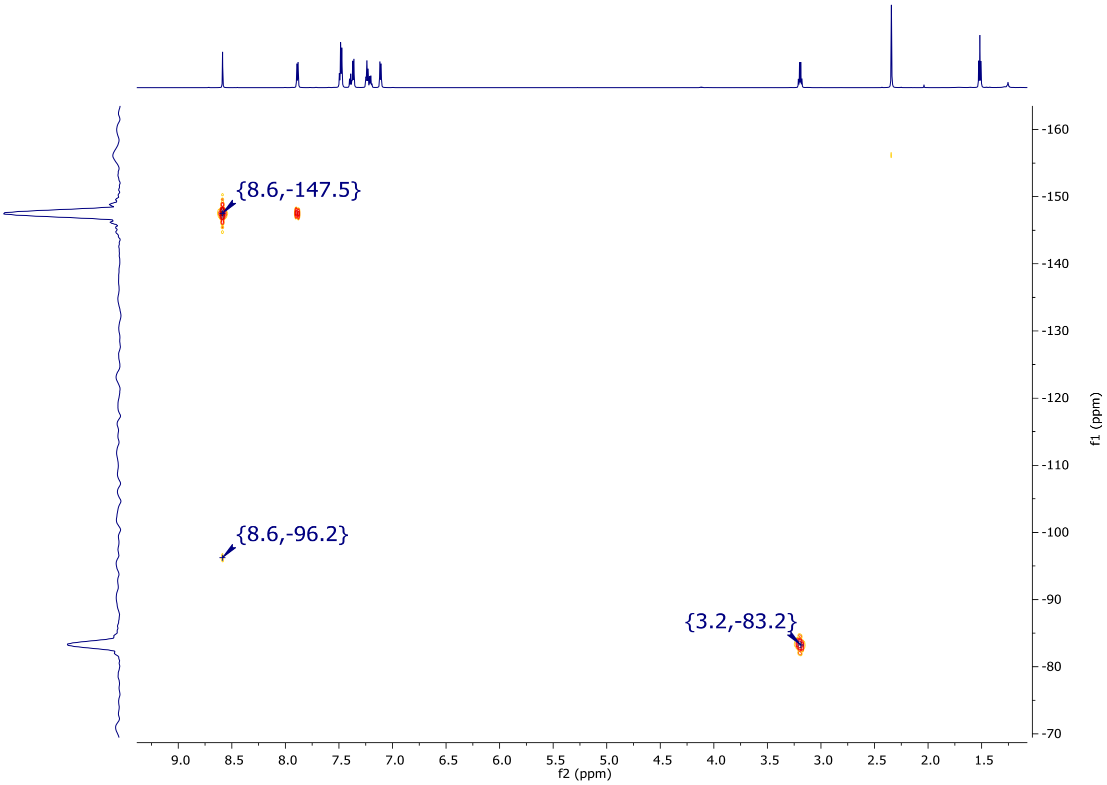

Figure S108. 4-Ethyl-2,6-diphenyl-7-(*p*-tolyl)-2*H*-pyrazolo[4,3-*c*]pyridine (**33**). ^{1}H-^{15}N HMBC spectrum (700 MHz, $CDCl_3$).

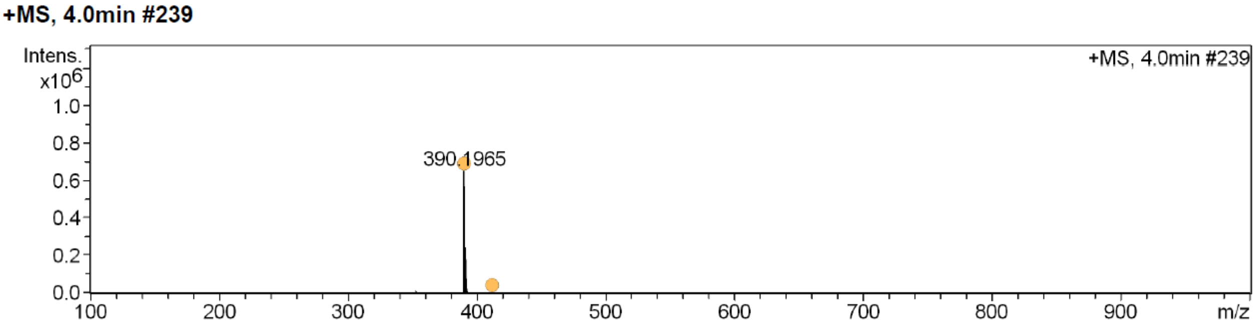

Meas. m/z	#	Ion Formula	m/z	err [ppm]	mSigma	# Sigma	Score	rdb	e⁻ Conf	N-Rule
390.1965	1	C27H24N3	390.1965	-0.2	3.5	1	100.00	17.5	even	ok
412.1796	1	C27H23N3Na	412.1784	-2.8	772.2	1	-1.#J	17.5	even	ok

Figure S109. 4-Ethyl-2,6-diphenyl-7-(*p*-tolyl)-2*H*-pyrazolo[4,3-*c*]pyridine (**33**). HRMS (ESI-TOF).

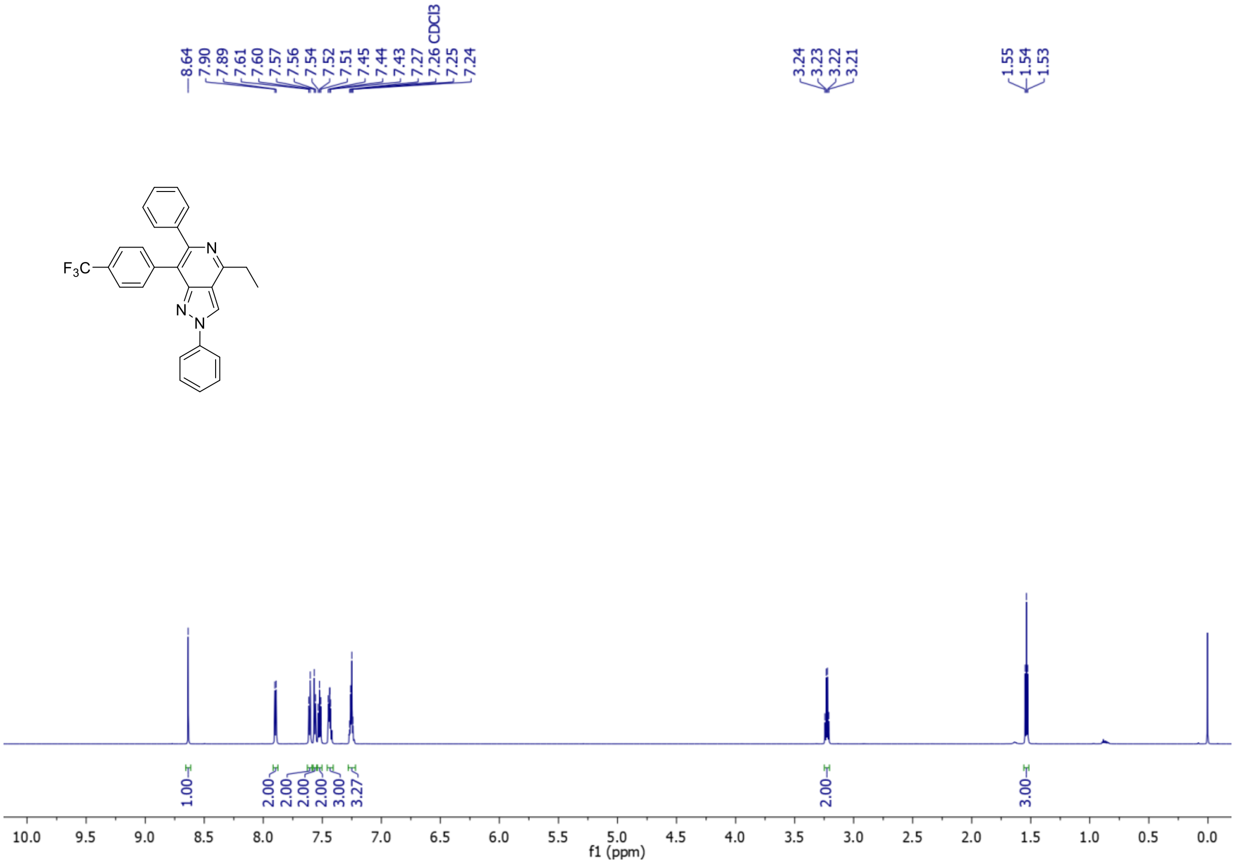

Figure S110. 4-Ethyl-2,6-diphenyl-7-(4-(trifluoromethyl)phenyl)-2*H*-pyrazolo[4,3-*c*]pyridine (**34**). ^{1}H NMR spectrum (700 MHz, $CDCl_3$).

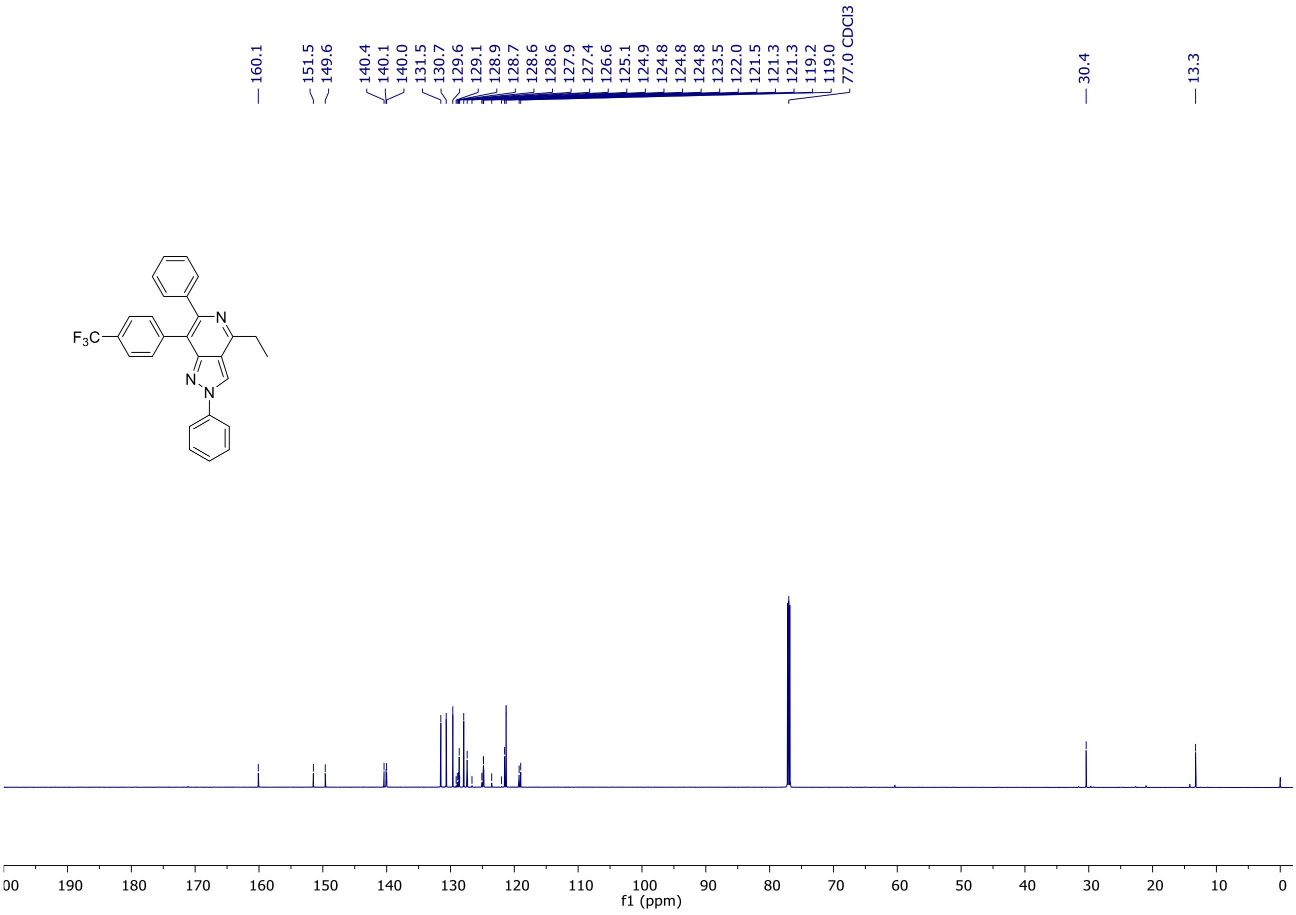

Figure S111. 4-Ethyl-2,6-diphenyl-7-(4-(trifluoromethyl)phenyl)-2*H*-pyrazolo[4,3-*c*]pyridine (**34**). ^{13}C NMR (176 MHz, $CDCl_3$).

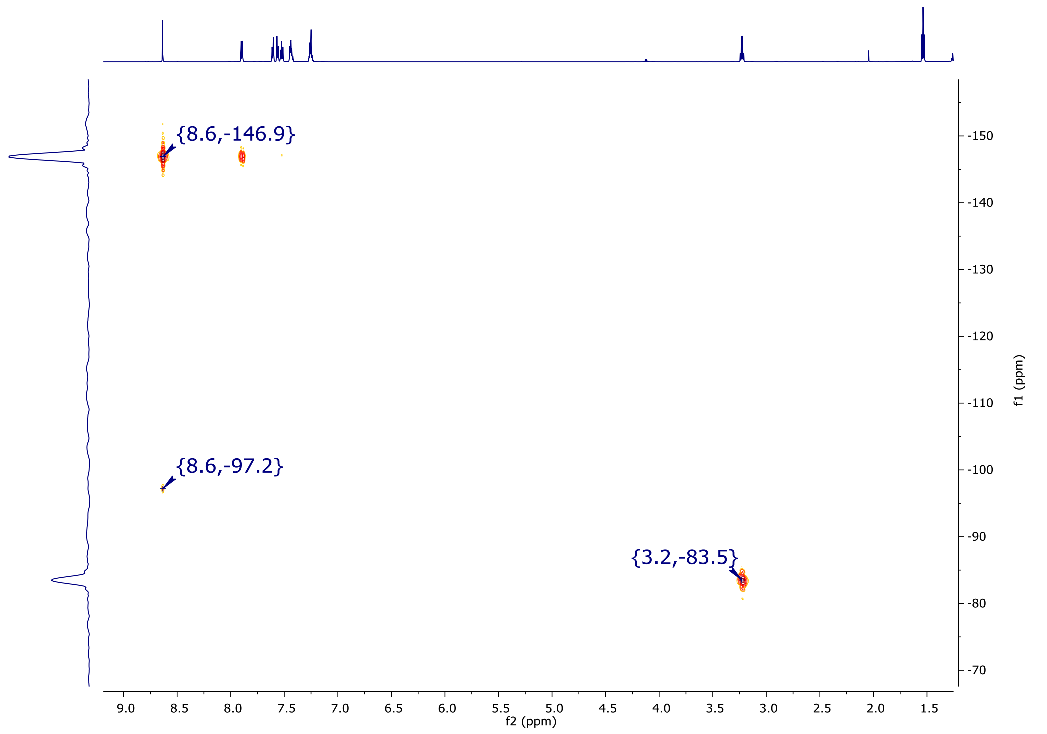

Figure S112. 4-Ethyl-2,6-diphenyl-7-(4-(trifluoromethyl)phenyl)-2*H*-pyrazolo[4,3-*c*]pyridine (**34**). ^{1}H-^{15}N HMBC spectrum (700 MHz, $CDCl_3$).

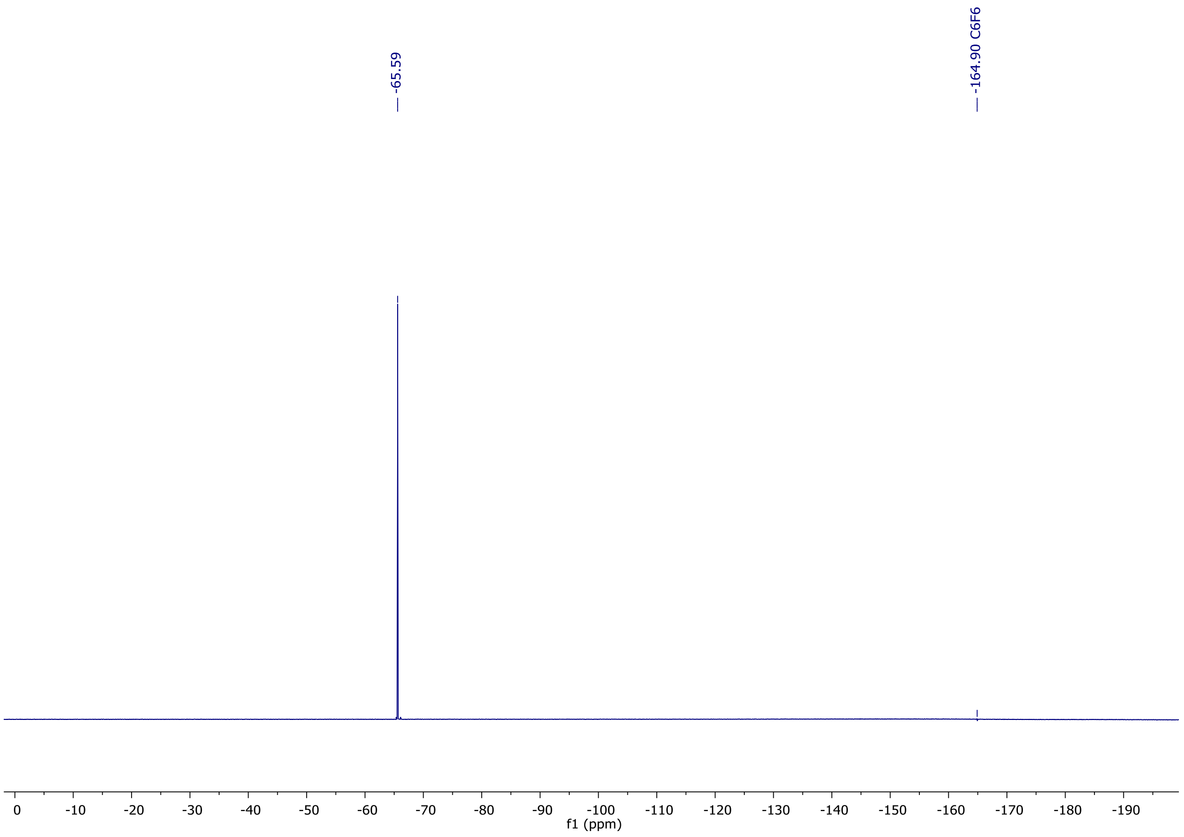

Figure S113. 4-Ethyl-2,6-diphenyl-7-(4-(trifluoromethyl)phenyl)-2*H*-pyrazolo[4,3-*c*]pyridine (**34**). ^{19}F NMR spectrum (500 MHz, $CDCl_3$).

+MS, 3.3min #200

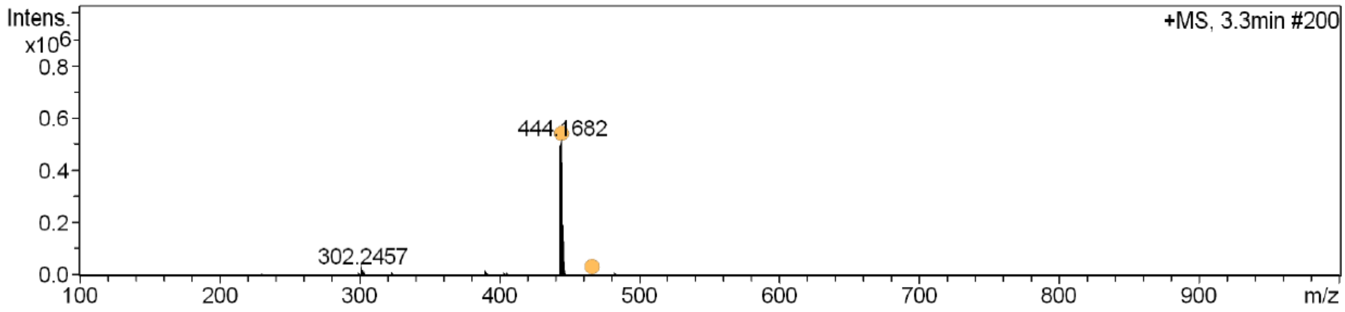

Meas. m/z	#	Ion Formula	m/z	err [ppm]	mSigma	# Sigma	Score	rdb	e⁻ Conf	N-Rule
444.1682	1	C27H21F3N3	444.1682	0.0	2.4	1	100.00	17.5	even	ok
466.1509	1	C27H20F3N3Na	466.1502	-1.6	105.2	1	100.00	17.5	even	ok

Figure S114. 4-Ethyl-2,6-diphenyl-7-(4-(trifluoromethyl)phenyl)-2*H*-pyrazolo[4,3-*c*]pyridine (**34**). HRMS (ESI-TOF).

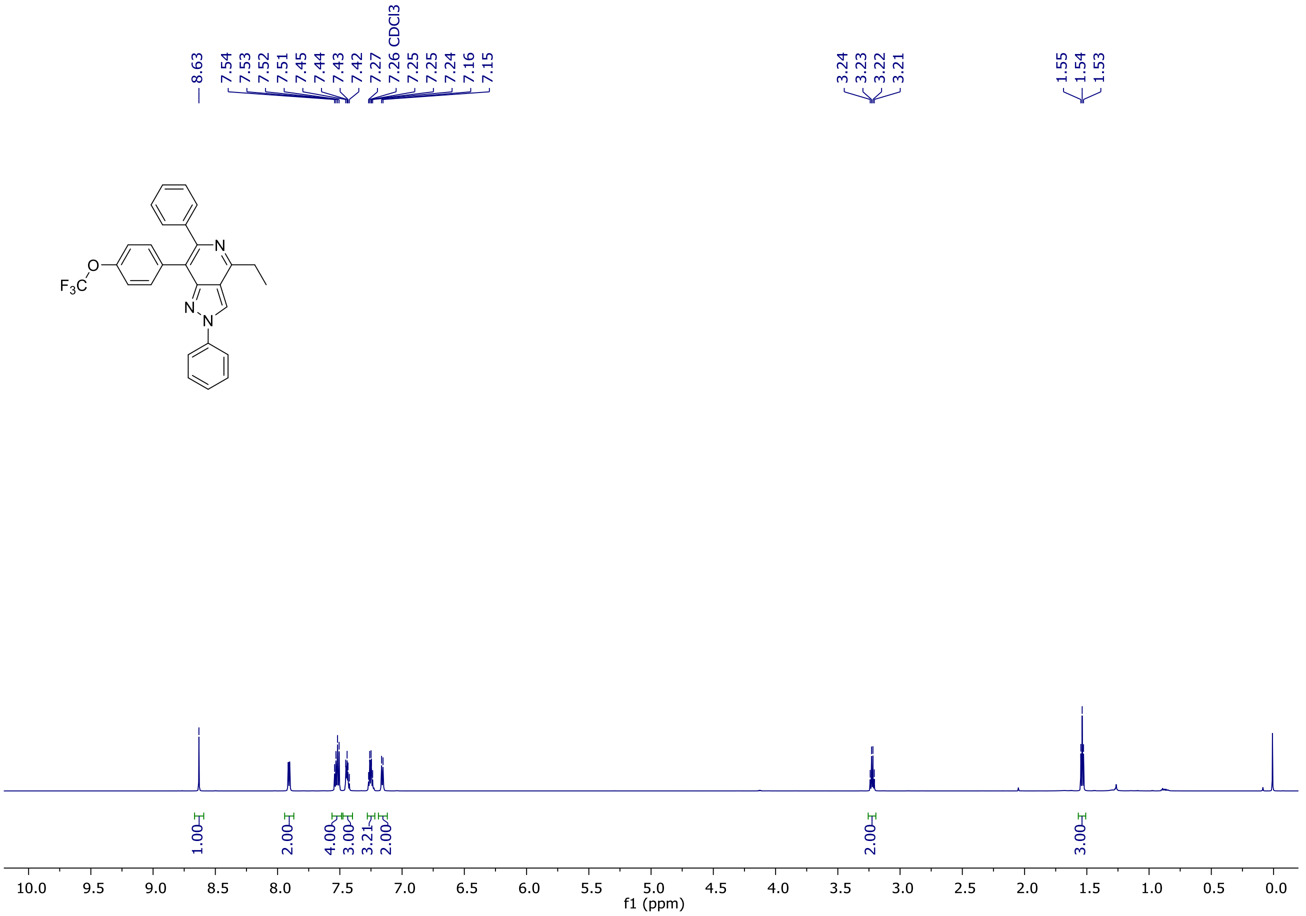

Figure S115. 4-Ethyl-2,6-diphenyl-7-(4-(trifluoromethoxy)phenyl)-2*H*-pyrazolo[4,3-*c*]pyridine (**35**). ^{1}H NMR spectrum (700 MHz, $CDCl_3$).

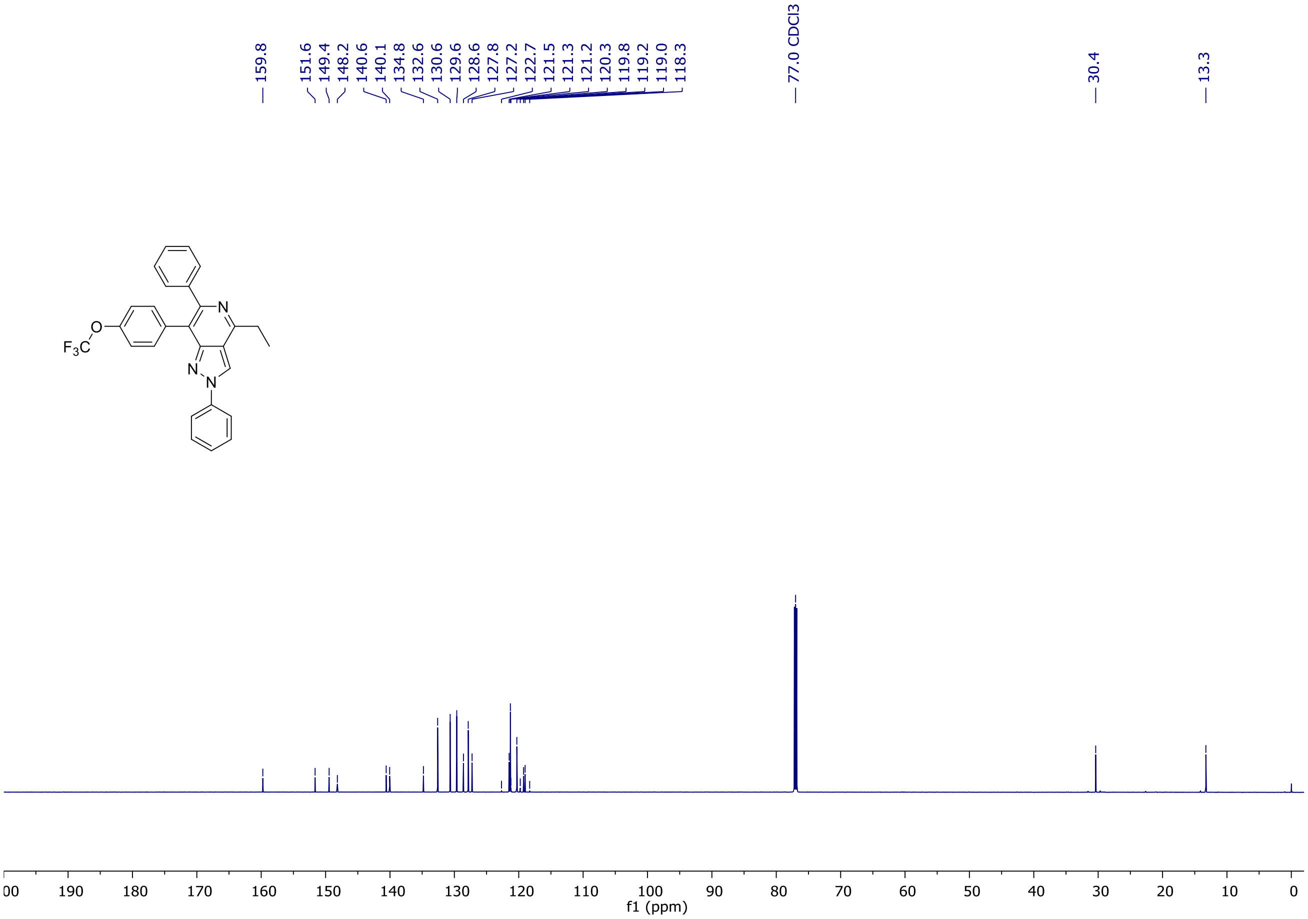

Figure S116. 4-Ethyl-2,6-diphenyl-7-(4-(trifluoromethoxy)phenyl)-2*H*-pyrazolo[4,3-*c*]pyridine (**35**). ^{13}C NMR (176 MHz, $CDCl_3$).

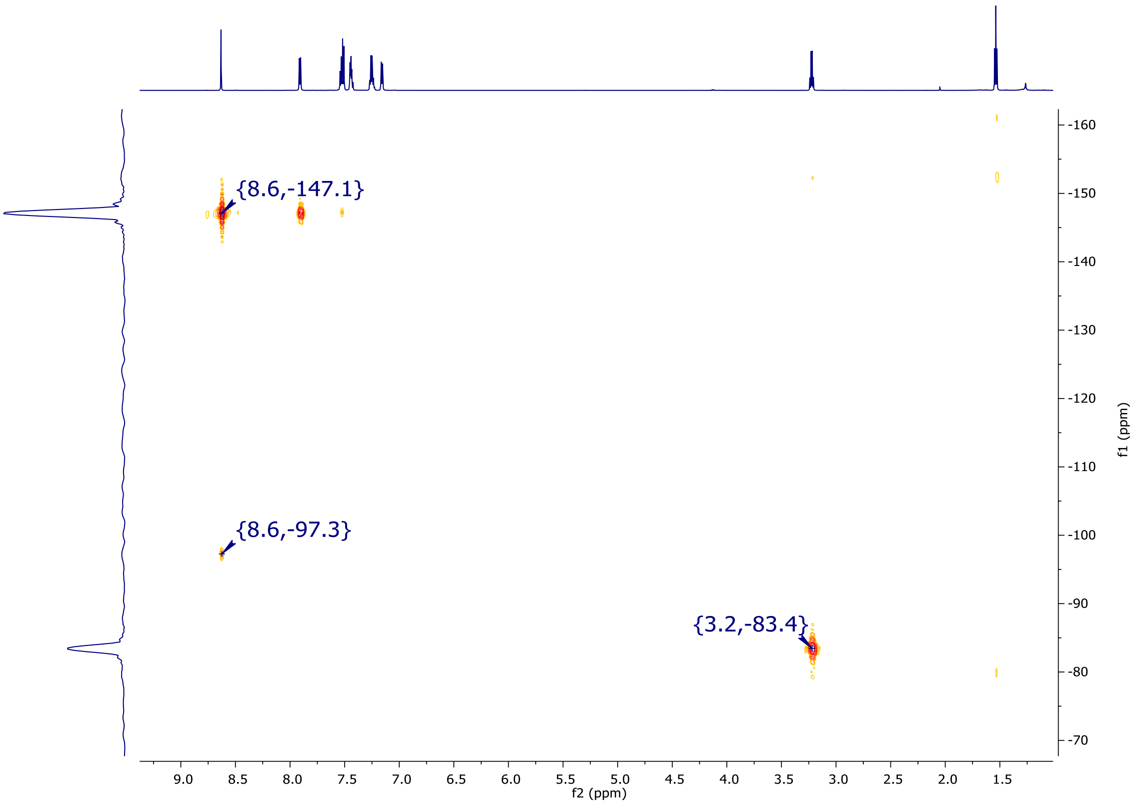

Figure S117. 4-Ethyl-2,6-diphenyl-7-(4-(trifluoromethoxy)phenyl)-2*H*-pyrazolo[4,3-*c*]pyridine (**35**). ^{1}H-^{15}N HMBC spectrum (700 MHz, $CDCl_3$).

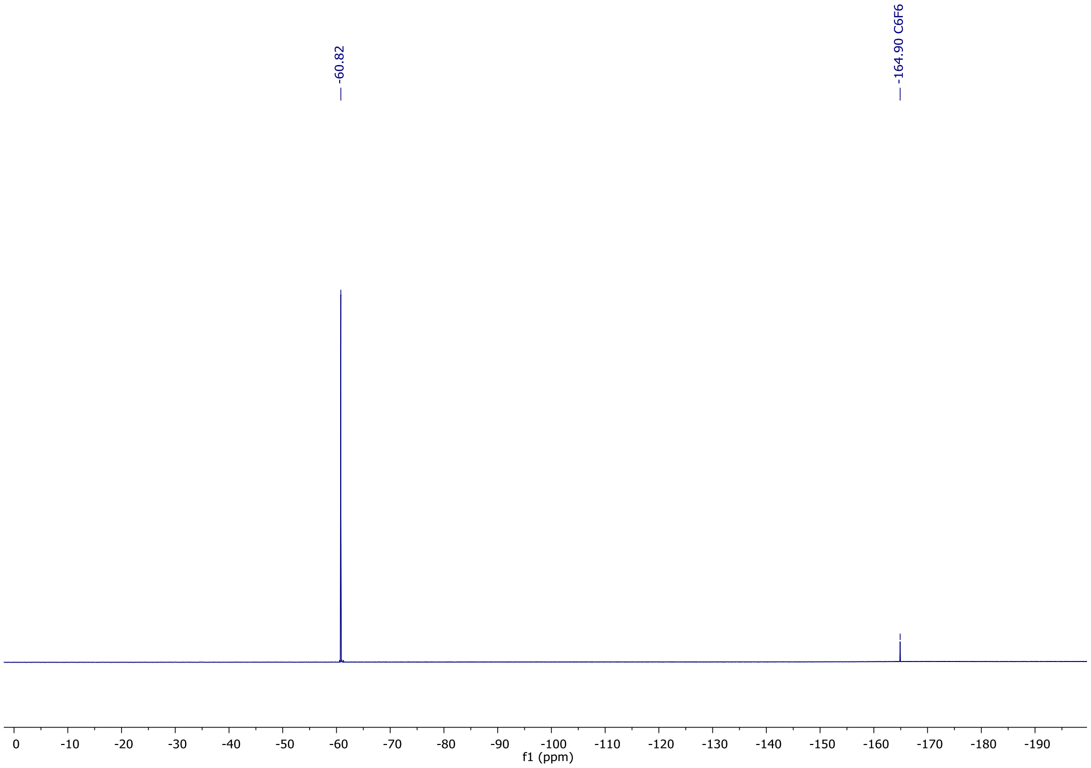

Figure S118. 4-Ethyl-2,6-diphenyl-7-(4-(trifluoromethoxy)phenyl)-2*H*-pyrazolo[4,3-*c*]pyridine (**35**). ^{19}F NMR spectrum (500 MHz, $CDCl_3$).

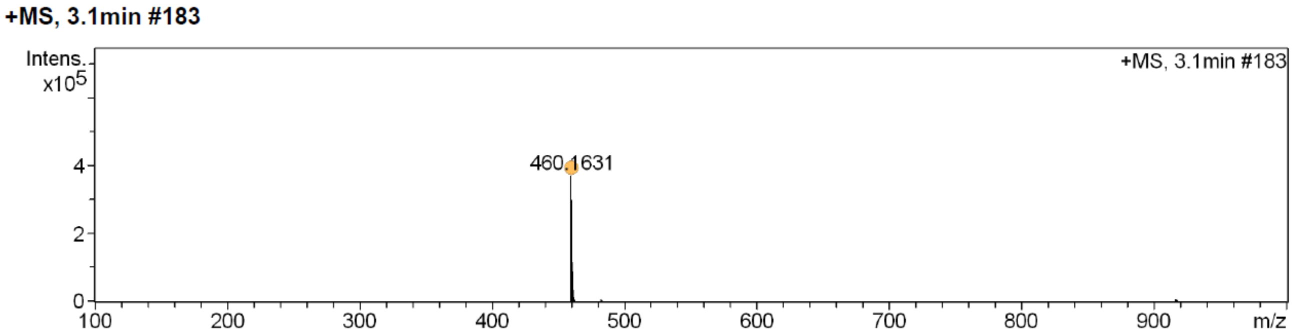

Meas. m/z	#	Ion Formula	m/z	err [ppm]	mSigma	# Sigma	Score	rdb	e⁻ Conf	N-Rule
460.1631	1	C27H21F3N3O	460.1631	0.1	5.5	1	100.00	17.5	even	ok

Figure S119. 4-Ethyl-2,6-diphenyl-7-(4-(trifluoromethoxy)phenyl)-2*H*-pyrazolo[4,3-*c*]pyridine (**35**). HRMS (ESI-TOF).

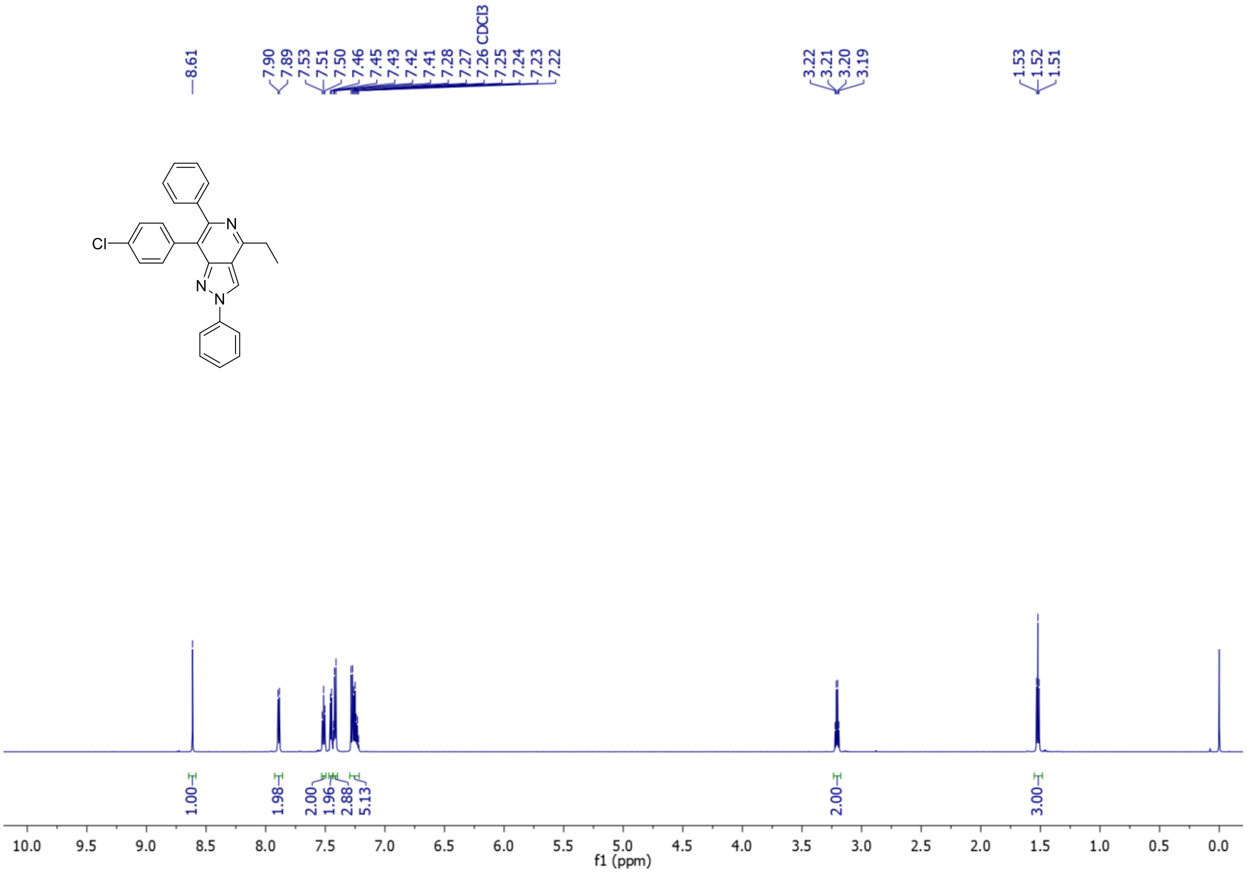

Figure S120. 7-(4-Chlorophenyl)-4-ethyl-2,6-diphenyl-2*H*-pyrazolo[4,3-*c*]pyridine (**36**). ^{1}H NMR spectrum (700 MHz, $CDCl_3$).

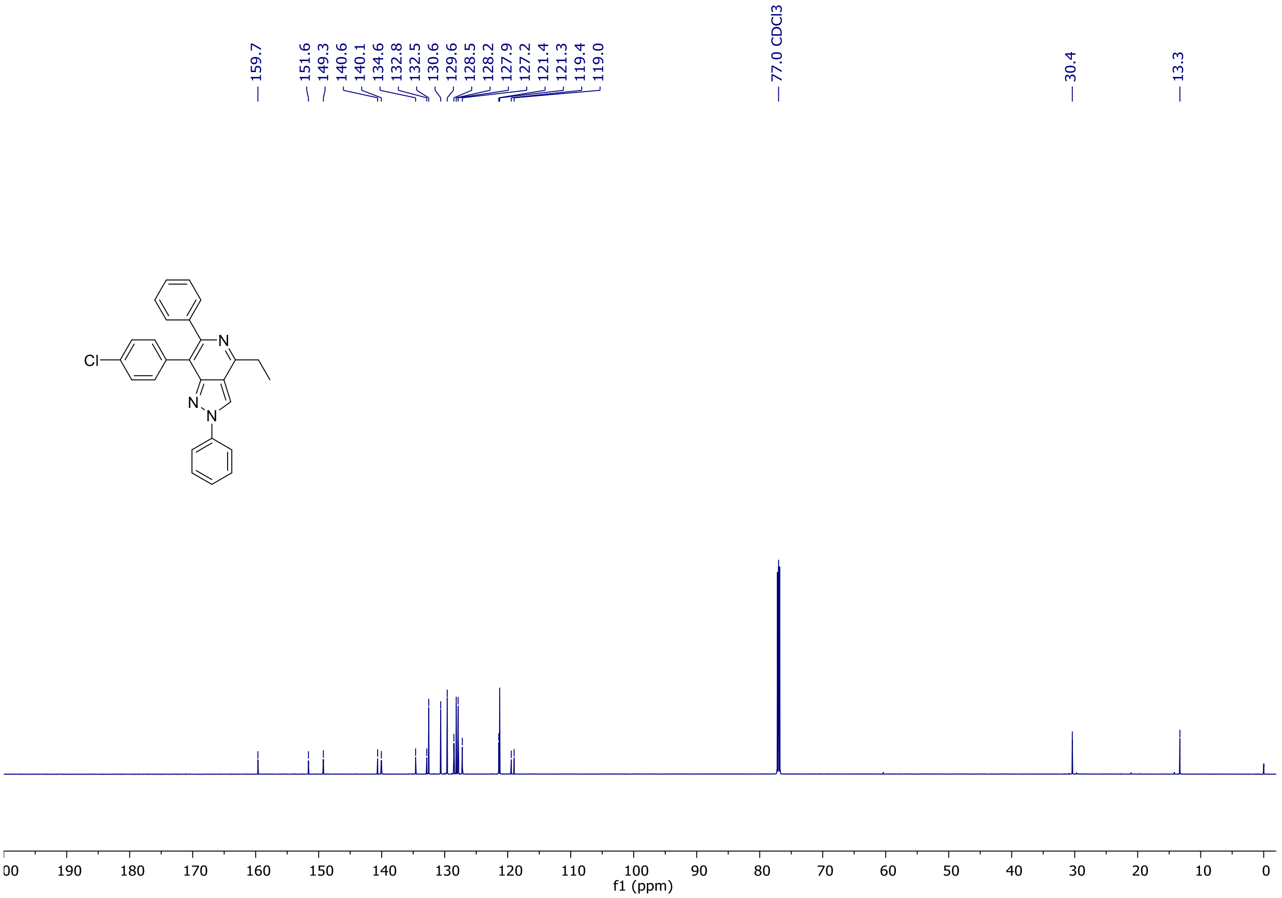

Figure S121. 7-(4-Chlorophenyl)-4-ethyl-2,6-diphenyl-2*H*-pyrazolo[4,3-*c*]pyridine (**36**). ^{13}C NMR (176 MHz, $CDCl_3$).

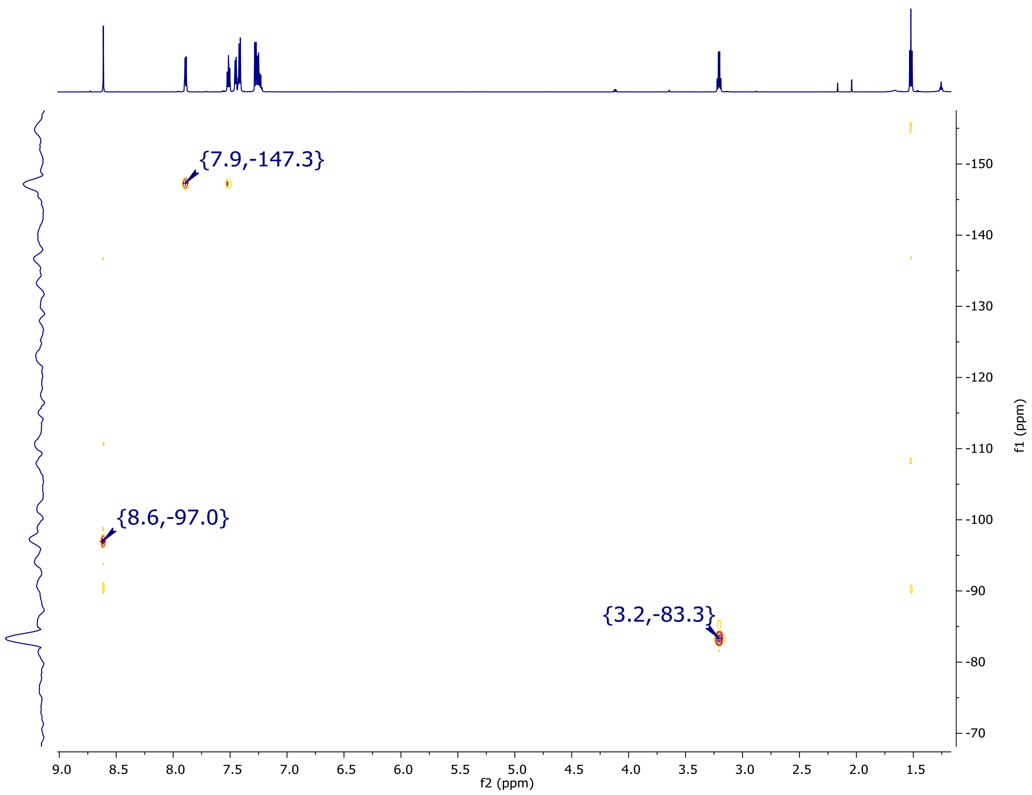

Figure S122. 7-(4-Chlorophenyl)-4-ethyl-2,6-diphenyl-2*H*-pyrazolo[4,3-*c*]pyridine (**36**). ^{1}H-^{15}N LR HSQMBC spectrum (700 MHz, $CDCl_3$).

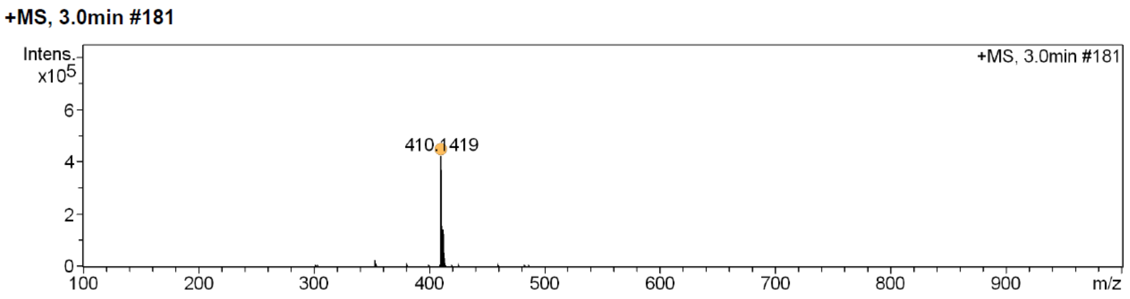

Meas. m/z	#	Ion Formula	m/z	err [ppm]	mSigma	# Sigma	Score	rdb	e⁻ Conf	N-Rule
410.1419	1	C26H21ClN3	410.1419	0.1	9.7	1	100.00	17.5	even	ok

Figure S123. 7-(4-Chlorophenyl)-4-ethyl-2,6-diphenyl-2*H*-pyrazolo[4,3-*c*]pyridine (**36**). HRMS (ESI-TOF).

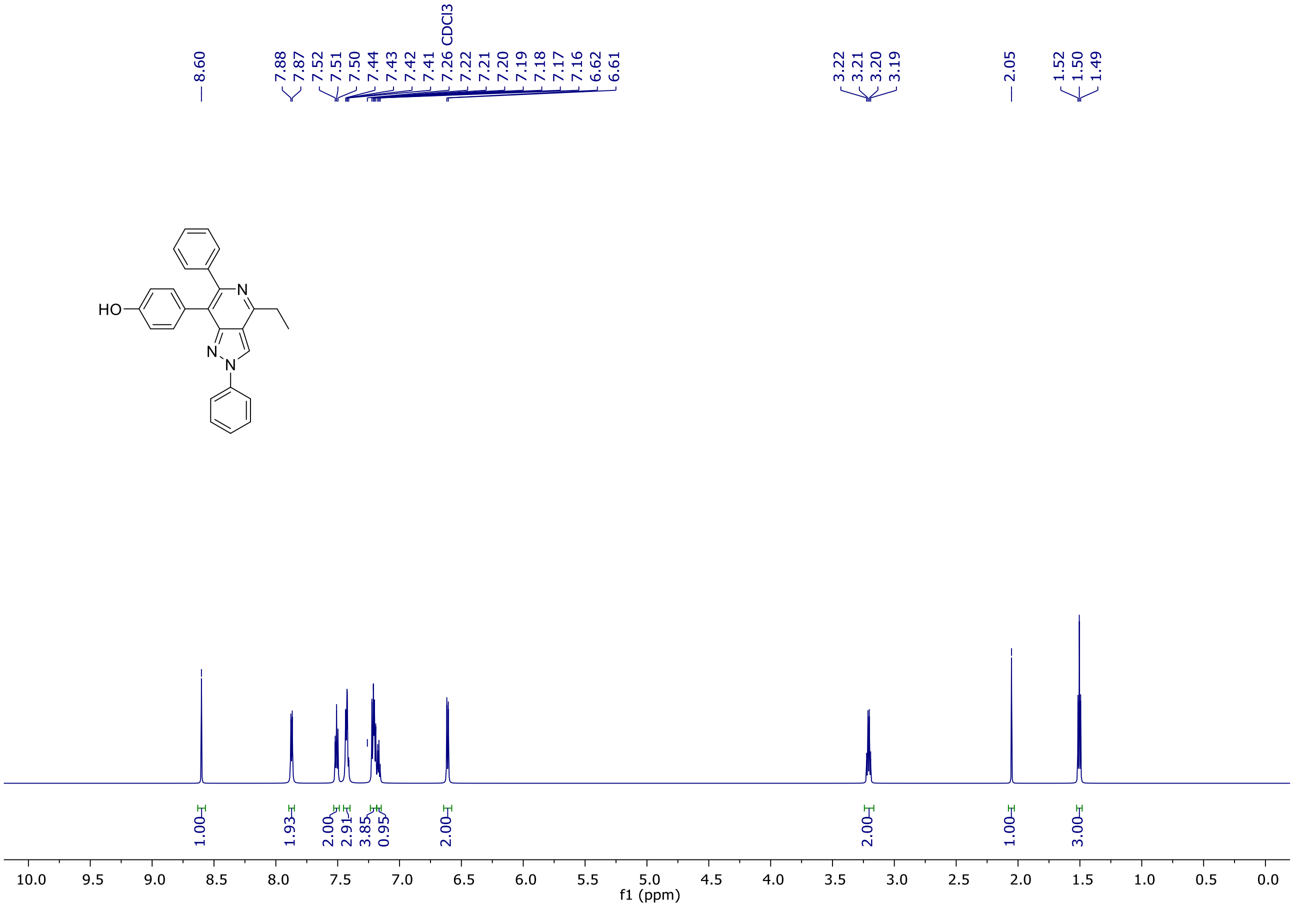

Figure S124. 4-(4-Ethyl-2,6-diphenyl-2*H*-pyrazolo[4,3-*c*]pyridin-7-yl)phenol (**37**). ^{1}H NMR spectrum (700 MHz, $CDCl_3$).

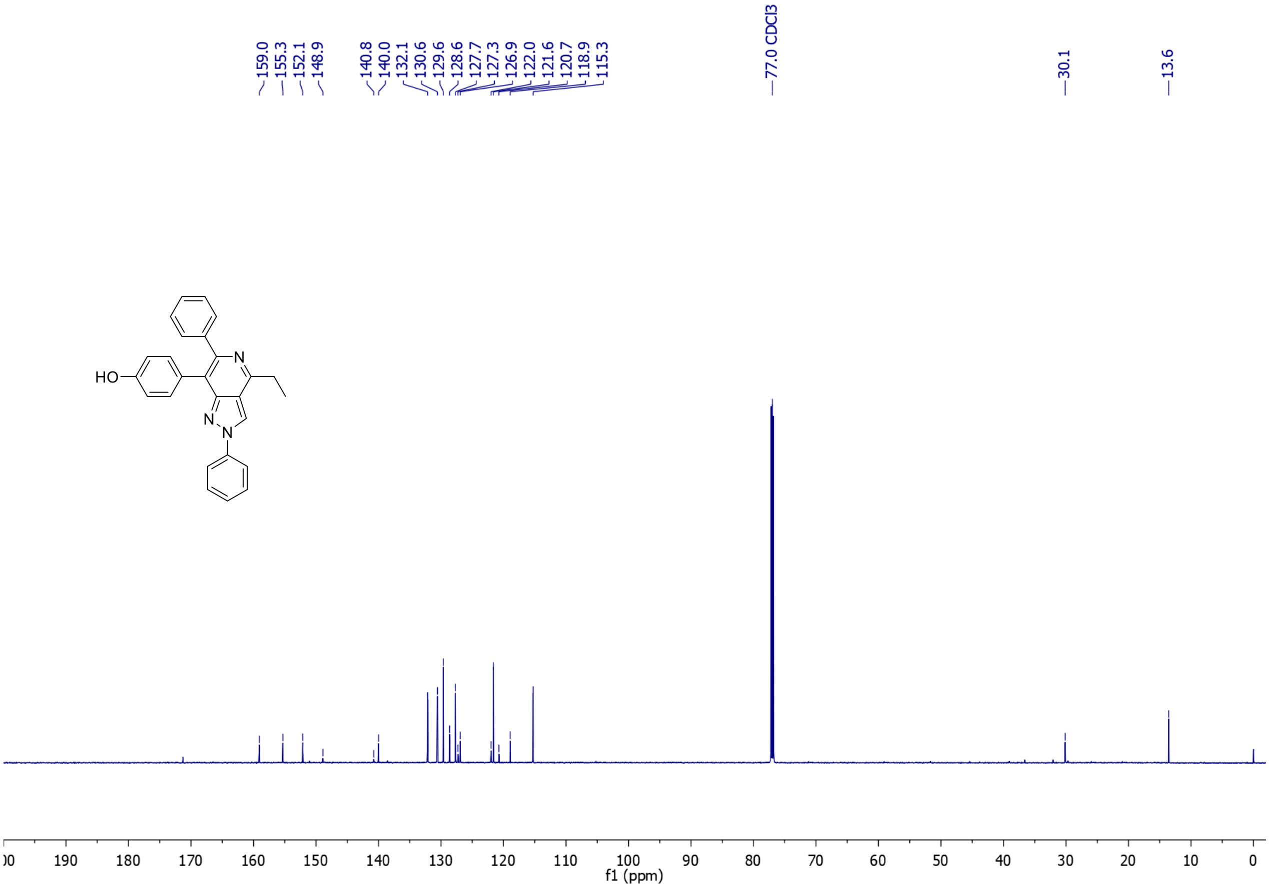

Figure S125. 4-(4-Ethyl-2,6-diphenyl-2*H*-pyrazolo[4,3-*c*]pyridin-7-yl)phenol (**37**). ^{13}C NMR (176 MHz, $CDCl_3$).

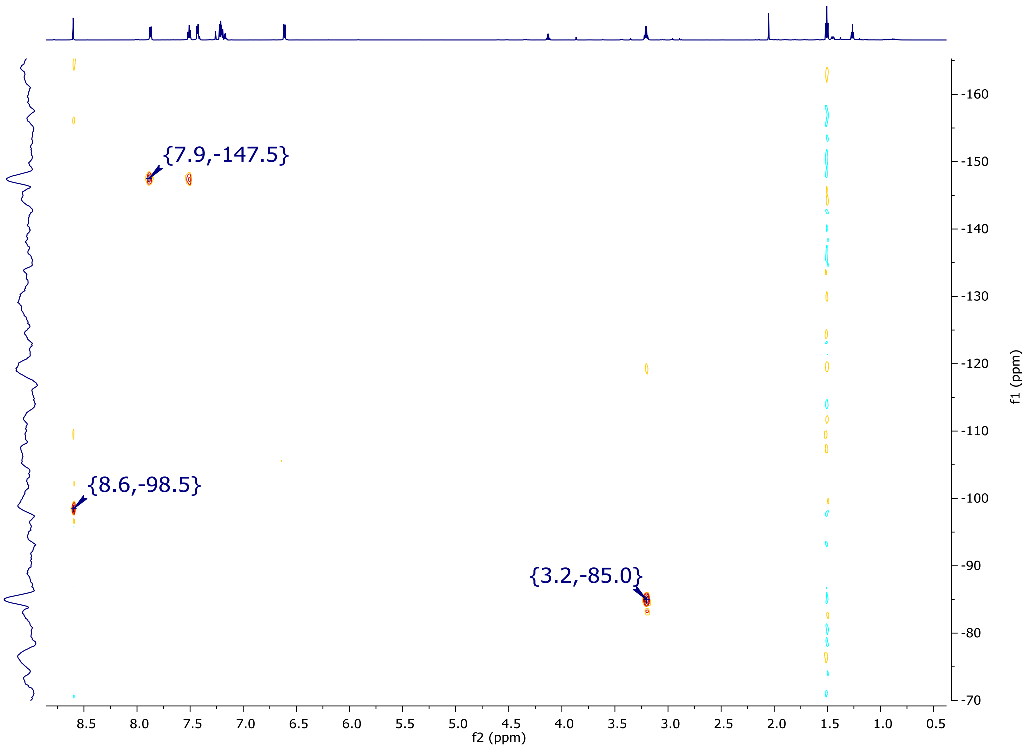

Figure S126. 4-(4-Ethyl-2,6-diphenyl-2*H*-pyrazolo[4,3-*c*]pyridin-7-yl)phenol (**37**). ^{1}H-^{15}N LR HSQMBC spectrum (700 MHz, $CDCl_3$).

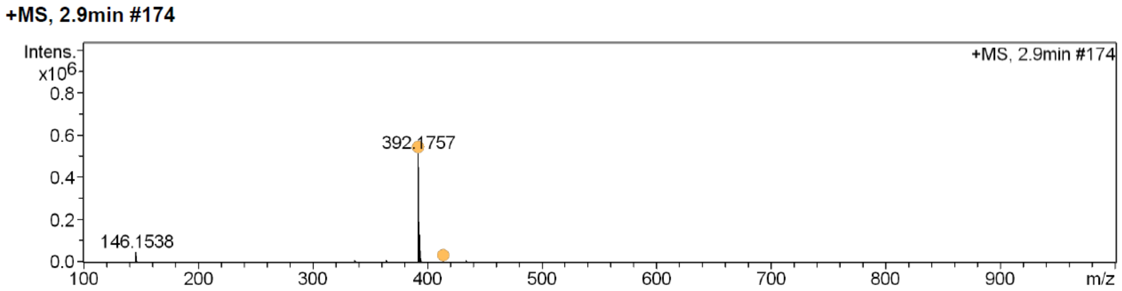

Meas. m/z	#	Ion Formula	m/z	err [ppm]	mSigma	# Sigma	Score	rdb	e¯ Conf	N-Rule
392.1757	1	C26H22N3O	392.1757	-0.0	3.0	1	100.00	17.5	even	ok
414.1589	1	C26H21N3NaO	414.1577	-2.8	41.5	1	100.00	17.5	even	ok

Figure S127. 4-(4-Ethyl-2,6-diphenyl-2*H*-pyrazolo[4,3-*c*]pyridin-7-yl)phenol (**37**). HRMS (ESI-TOF).

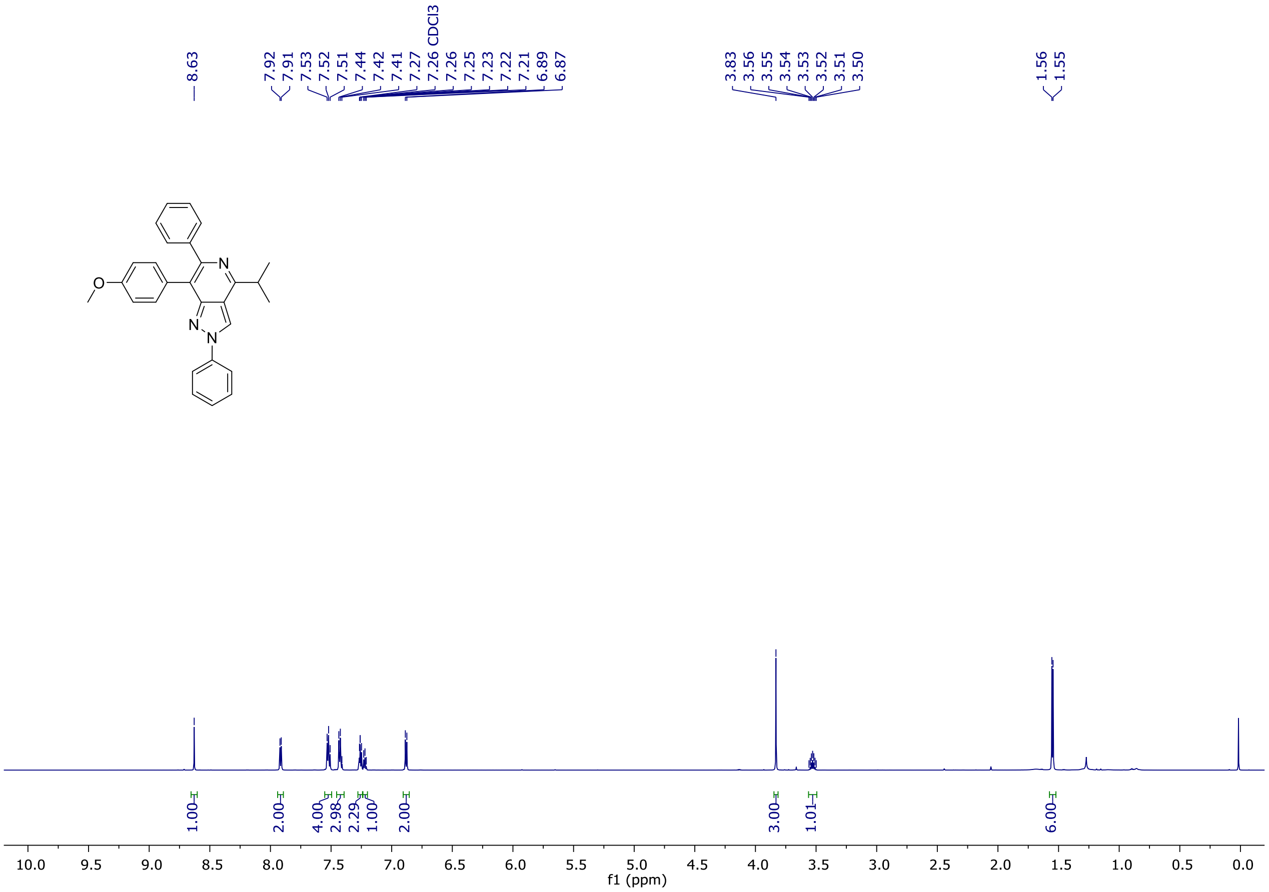

Figure S128. 4-Isopropyl-7-(4-methoxyphenyl)-4-methyl-2,6-diphenyl-2*H*-pyrazolo[4,3-*c*]pyridine (**38**). ^{1}H NMR spectrum (700 MHz, $CDCl_3$).

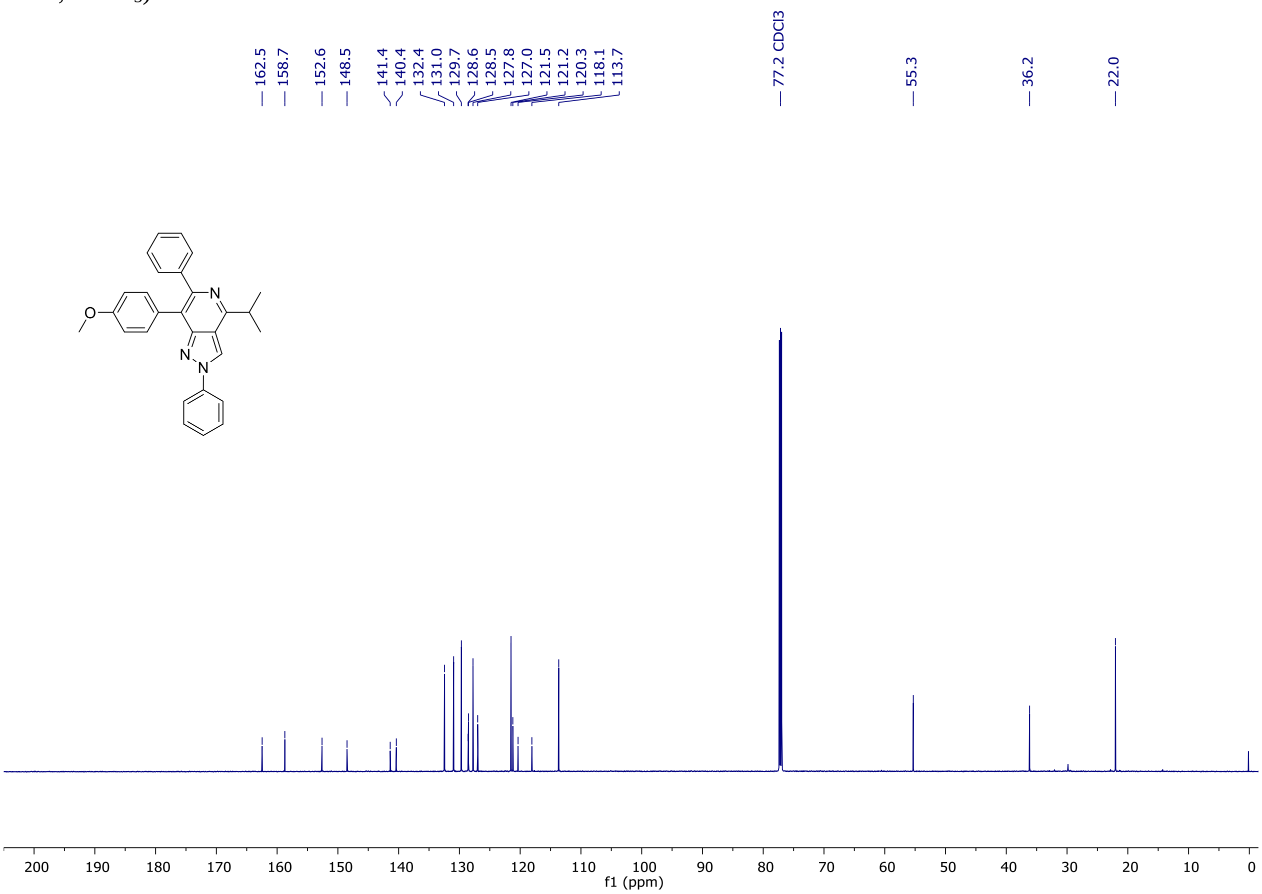

Figure S129. 4-Isopropyl-7-(4-methoxyphenyl)-4-methyl-2,6-diphenyl-2*H*-pyrazolo[4,3-*c*]pyridine (**38**). ^{13}C NMR (176 MHz, $CDCl_3$).

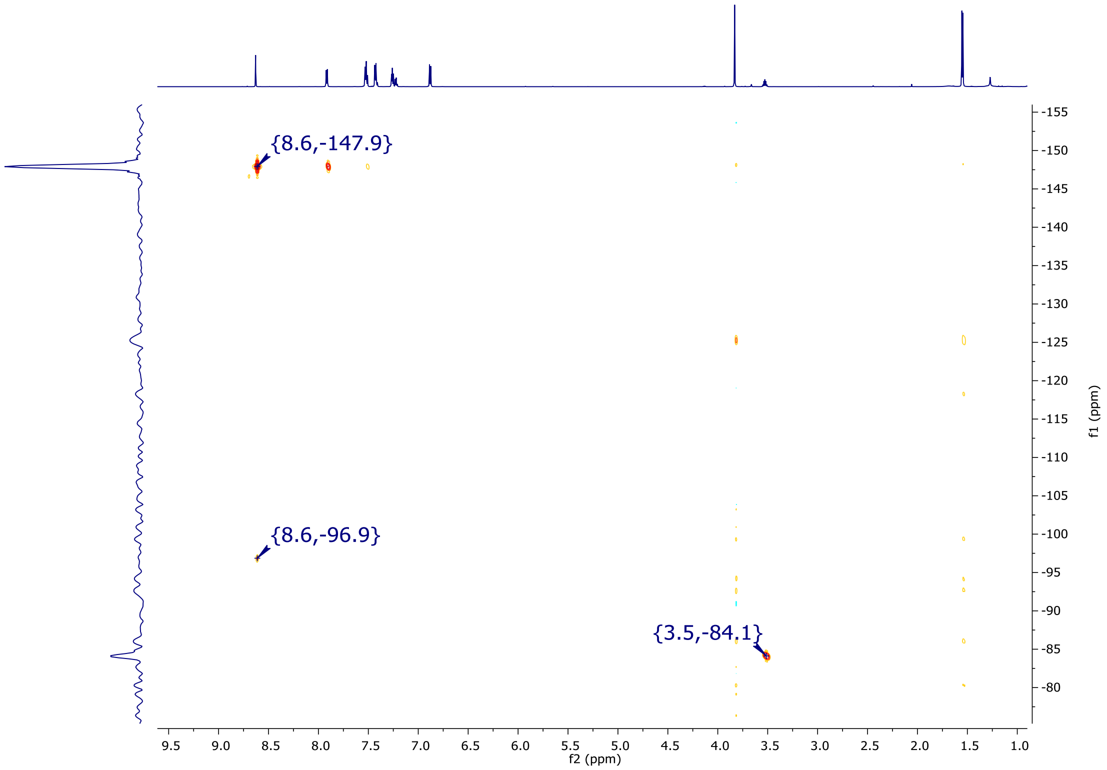

Figure S130. 4-Isopropyl-7-(4-methoxyphenyl)-4-methyl-2,6-diphenyl-2*H*-pyrazolo[4,3-*c*]pyridine (**38**). ^{1}H-^{15}N HMBC spectrum (700 MHz, $CDCl_3$).

+MS, 4.2min #246

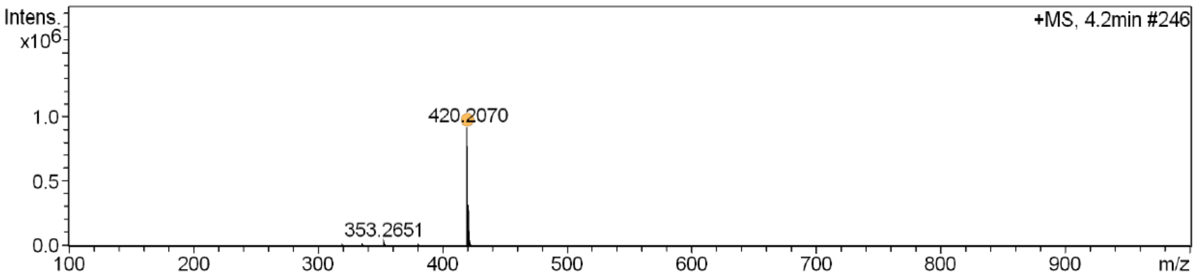

Meas. m/z	#	Ion Formula	m/z	err [ppm]	mSigma	# Sigma	Score	rdb	e⁻ Conf	N-Rule
420.2070	1	C28H26N3O	420.2070	-0.0	17.8	1	100.00	17.5	even	ok

Figure S131. 4-Isopropyl-7-(4-methoxyphenyl)-4-methyl-2,6-diphenyl-2*H*-pyrazolo[4,3-*c*]pyridine (**38**). HRMS (ESI-TOF).

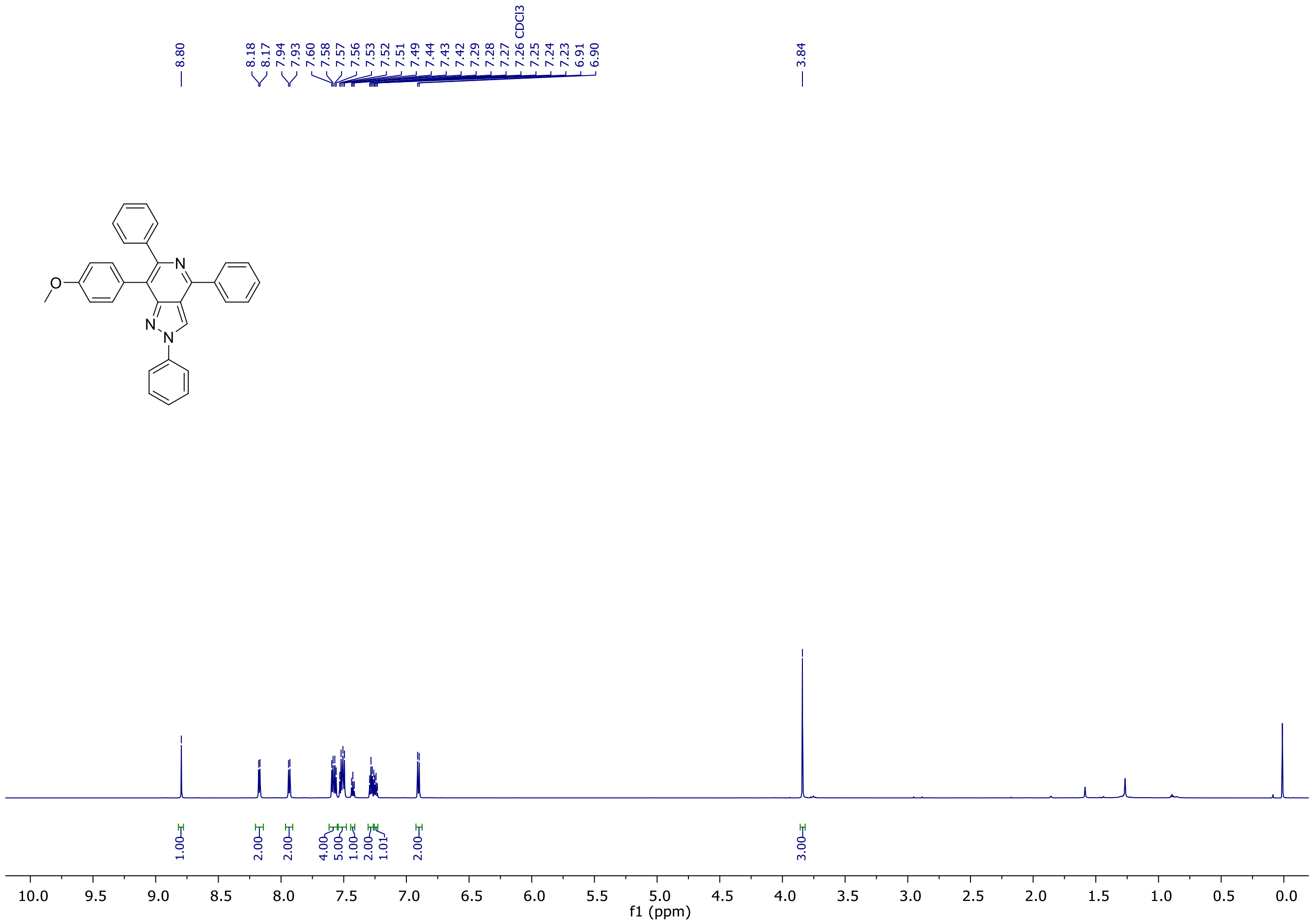

Figure S132. 7-(4-Methoxyphenyl)-2,4,6-triphenyl-2*H*-pyrazolo[4,3-*c*]pyridine (**39**). ^{1}H NMR spectrum (700 MHz, $CDCl_3$).

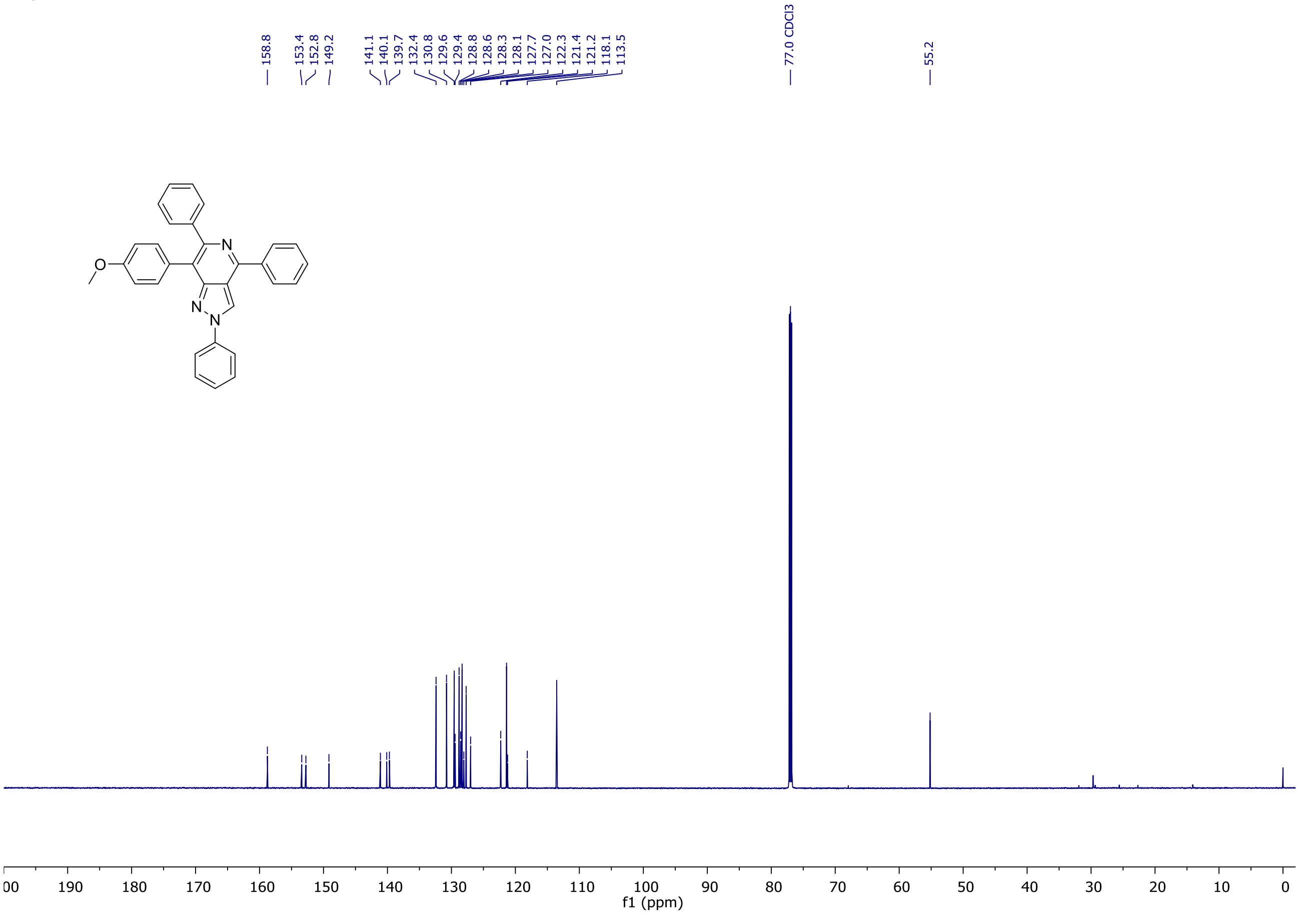

Figure S133. 7-(4-Methoxyphenyl)-2,4,6-triphenyl-2*H*-pyrazolo[4,3-*c*]pyridine (**39**). ^{13}C NMR (176 MHz, $CDCl_3$).

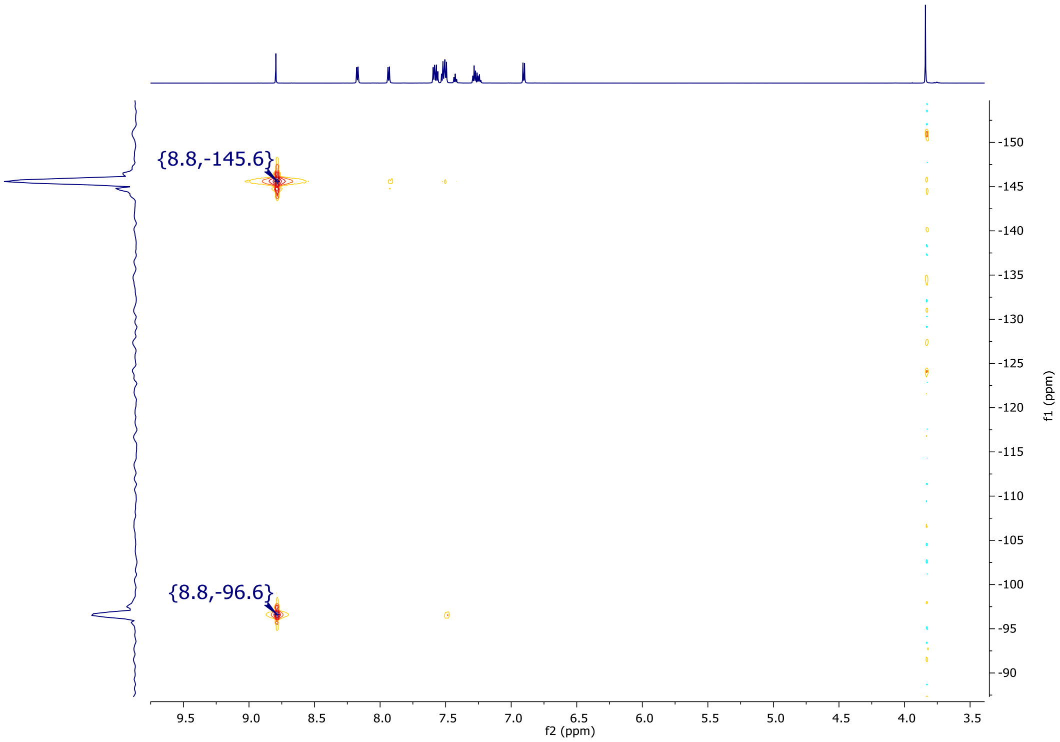

Figure S134. 7-(4-Methoxyphenyl)-2,4,6-triphenyl-2*H*-pyrazolo[4,3-*c*]pyridine (**39**). ^{1}H-^{15}N LR HSQMBC spectrum (700 MHz, $CDCl_3$).

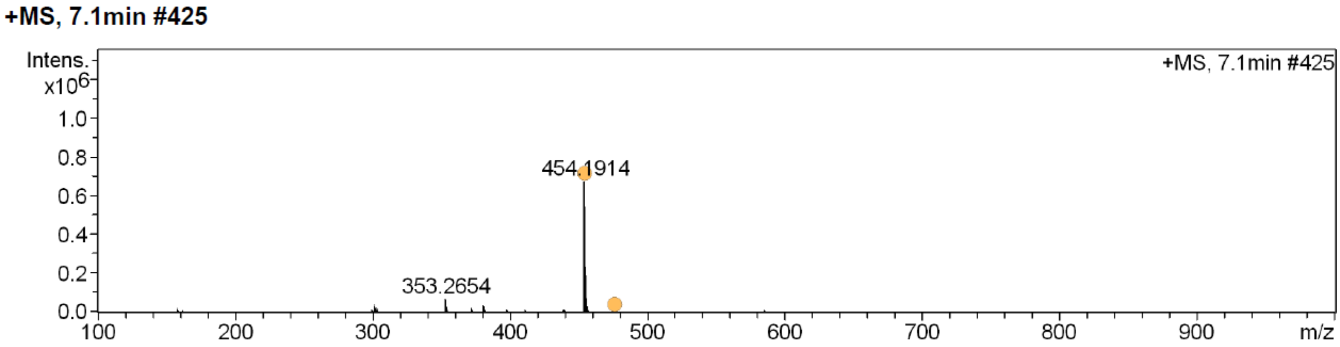

Meas. m/z	#	Ion Formula	m/z	err [ppm]	mSigma	# Sigma	Score	rdb	e⁻ Conf	N-Rule
454.1914	1	C31H24N3O	454.1914	0.0	1.4	1	100.00	21.5	even	ok
476.1721	1	C31H23N3NaO	476.1733	2.6	107.0	1	100.00	21.5	even	ok

Figure S135. 7-(4-Methoxyphenyl)-2,4,6-triphenyl-2*H*-pyrazolo[4,3-*c*]pyridine (**39**). HRMS (ESI-TOF).

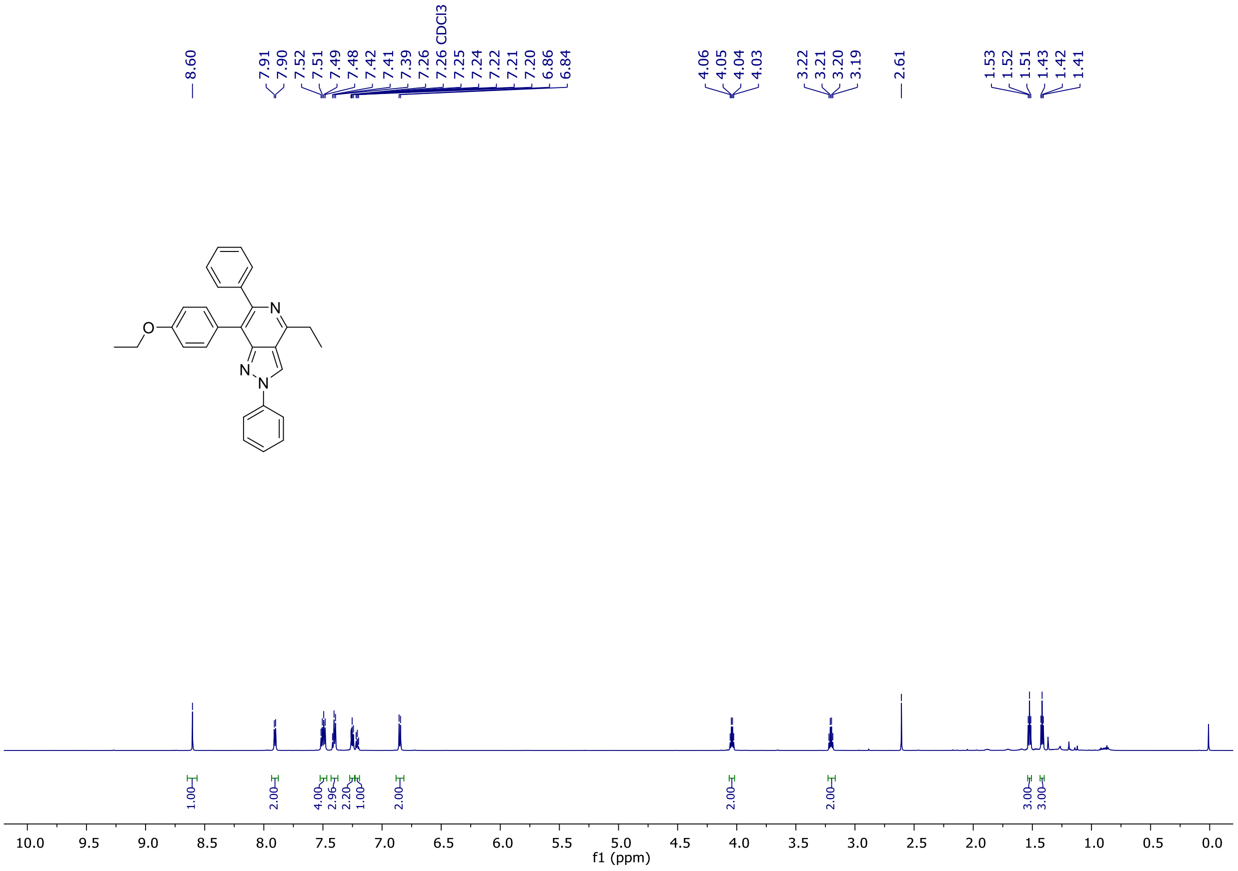

Figure S136. 7-(4-Ethoxyphenyl)-4-ethyl-2,6-diphenyl-2*H*-pyrazolo[4,3-*c*]pyridine (**40**). ^{1}H NMR spectrum (700 MHz, $CDCl_3$).

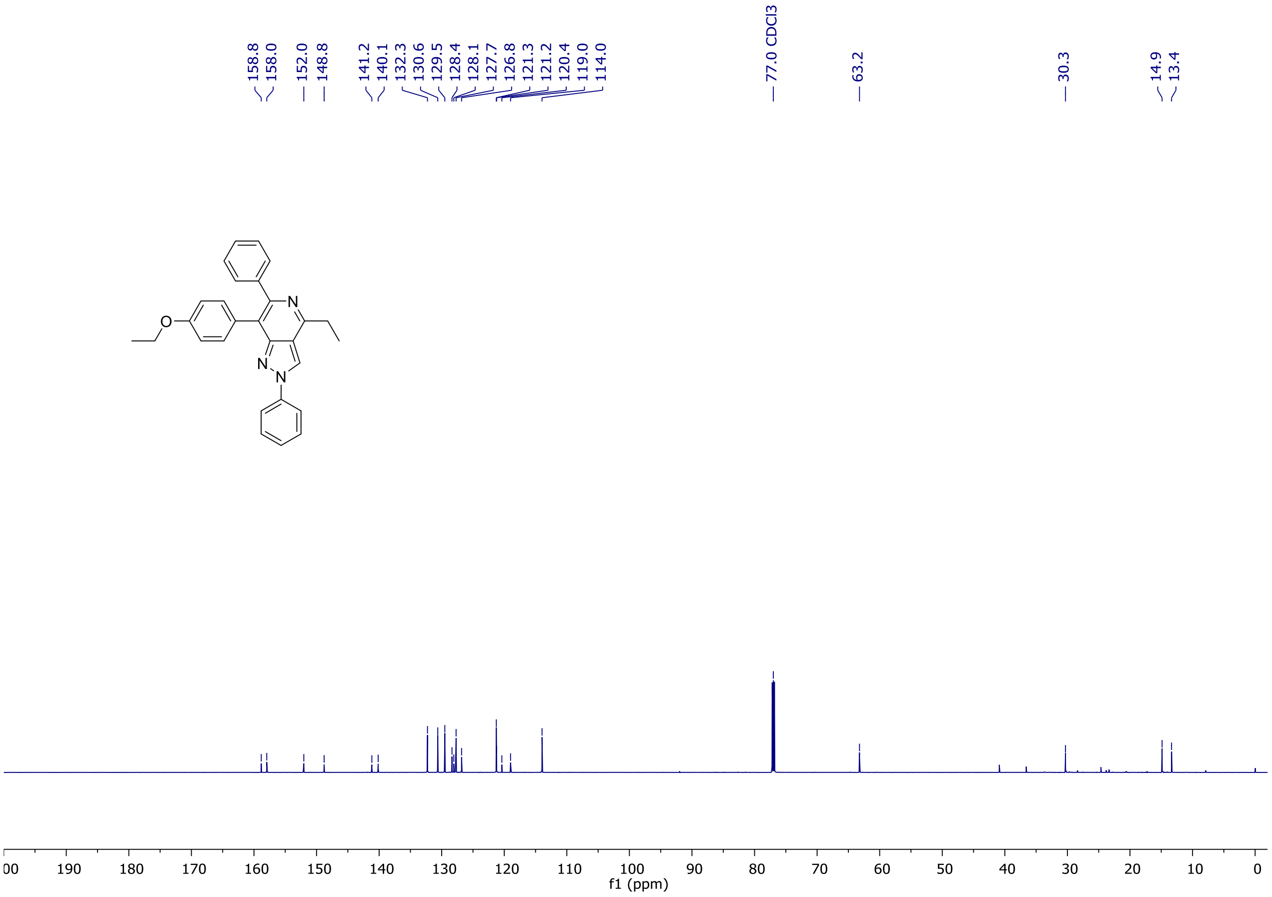

Figure S137. 7-(4-Ethoxyphenyl)-4-ethyl-2,6-diphenyl-2*H*-pyrazolo[4,3-*c*]pyridine (**40**). ^{13}C NMR (176 MHz, $CDCl_3$).

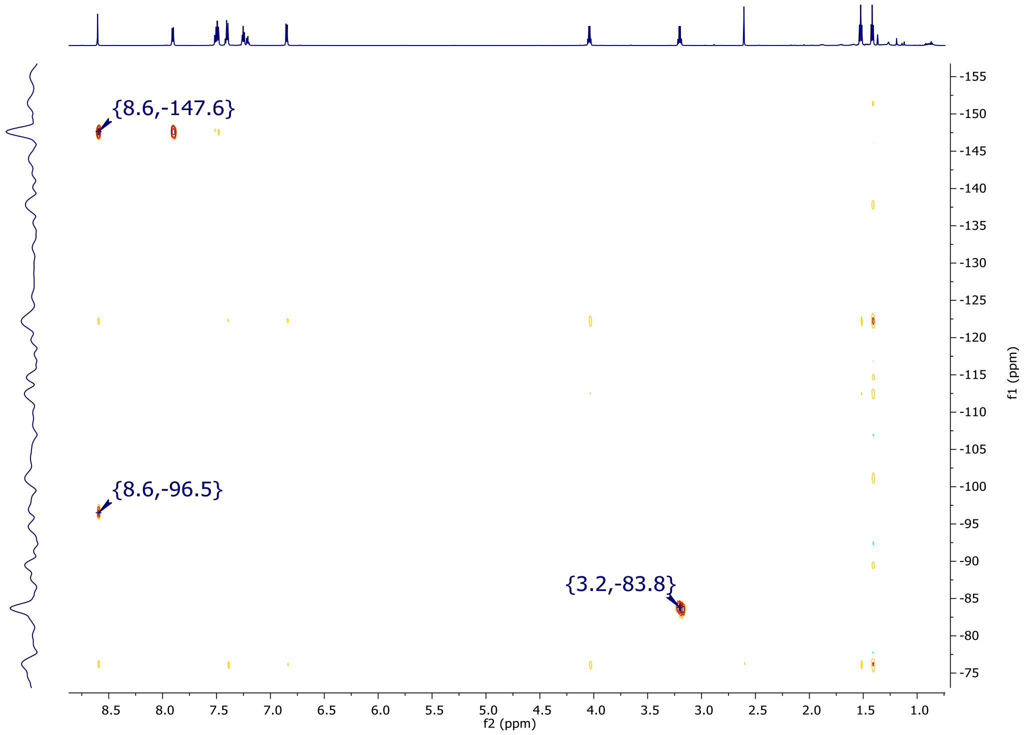

Figure S138. 7-(4-Ethoxyphenyl)-4-ethyl-2,6-diphenyl-2*H*-pyrazolo[4,3-*c*]pyridine (**40**). ^{1}H-^{15}N LR HSQMBC spectrum (700 MHz, $CDCl_3$).

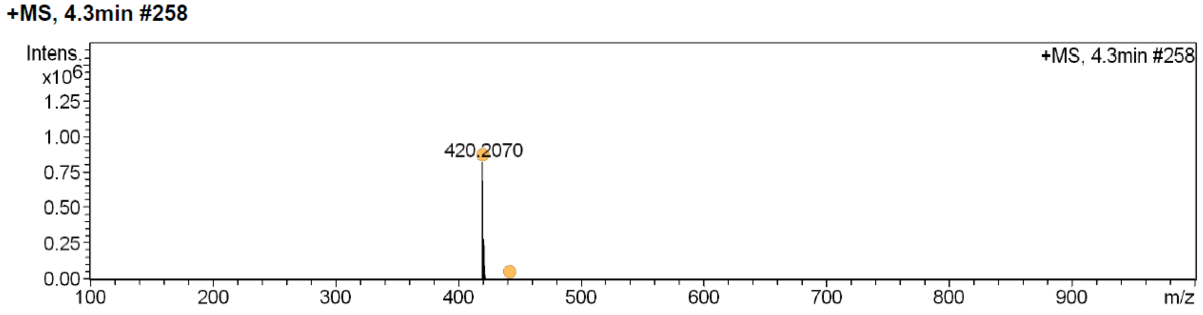

Meas. m/z	#	Ion Formula	m/z	err [ppm]	mSigma	# Sigma	Score	rdb	e⁻ Conf	N-Rule
420.2070	1	C28H26N3O	420.2070	-0.1	14.5	1	100.00	17.5	even	ok
442.1894	1	C28H25N3NaO	442.1890	-1.0	34.0	1	100.00	17.5	even	ok

Figure S139. 7-(4-Ethoxyphenyl)-4-ethyl-2,6-diphenyl-2*H*-pyrazolo[4,3-*c*]pyridine (**40**). HRMS (ESI-TOF).

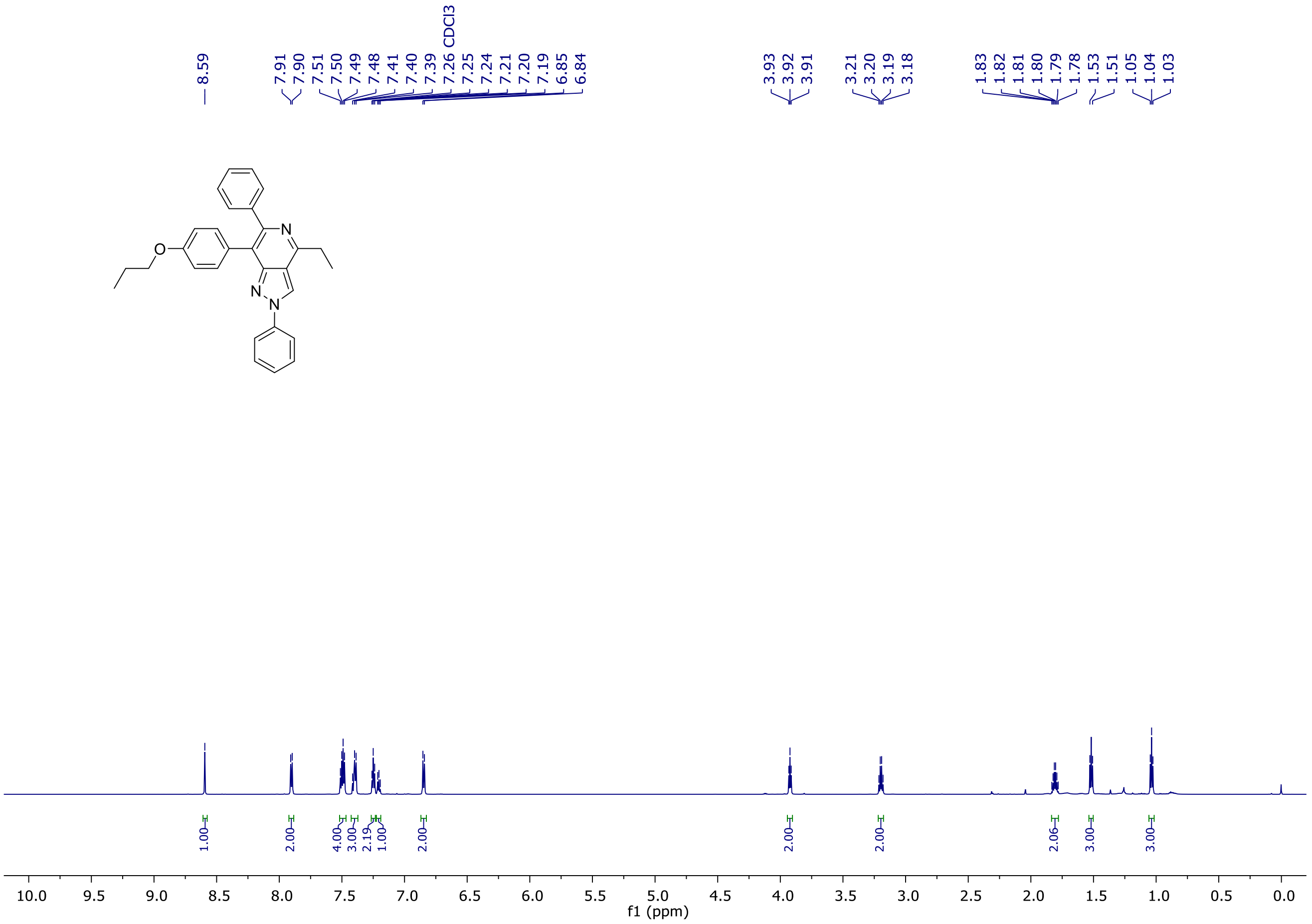

Figure S140. 4-Ethyl-2,6-diphenyl-7-(4-propoxyphenyl)-2*H*-pyrazolo[4,3-*c*]pyridine (**41**). ^{1}H NMR spectrum (700 MHz, $CDCl_3$).

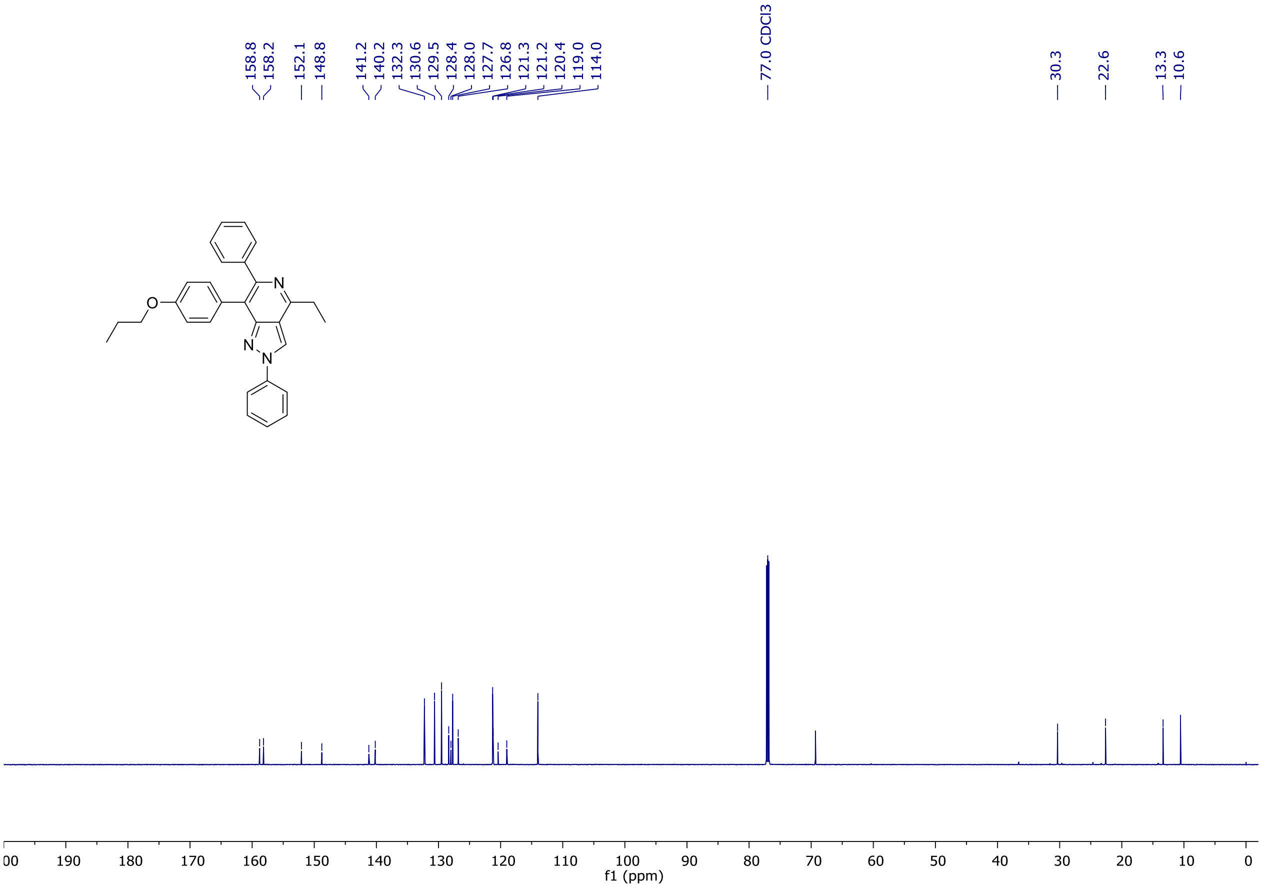

Figure S141. 4-Ethyl-2,6-diphenyl-7-(4-propoxyphenyl)-2*H*-pyrazolo[4,3-*c*]pyridine (**41**). ^{13}C NMR (176 MHz, $CDCl_3$).

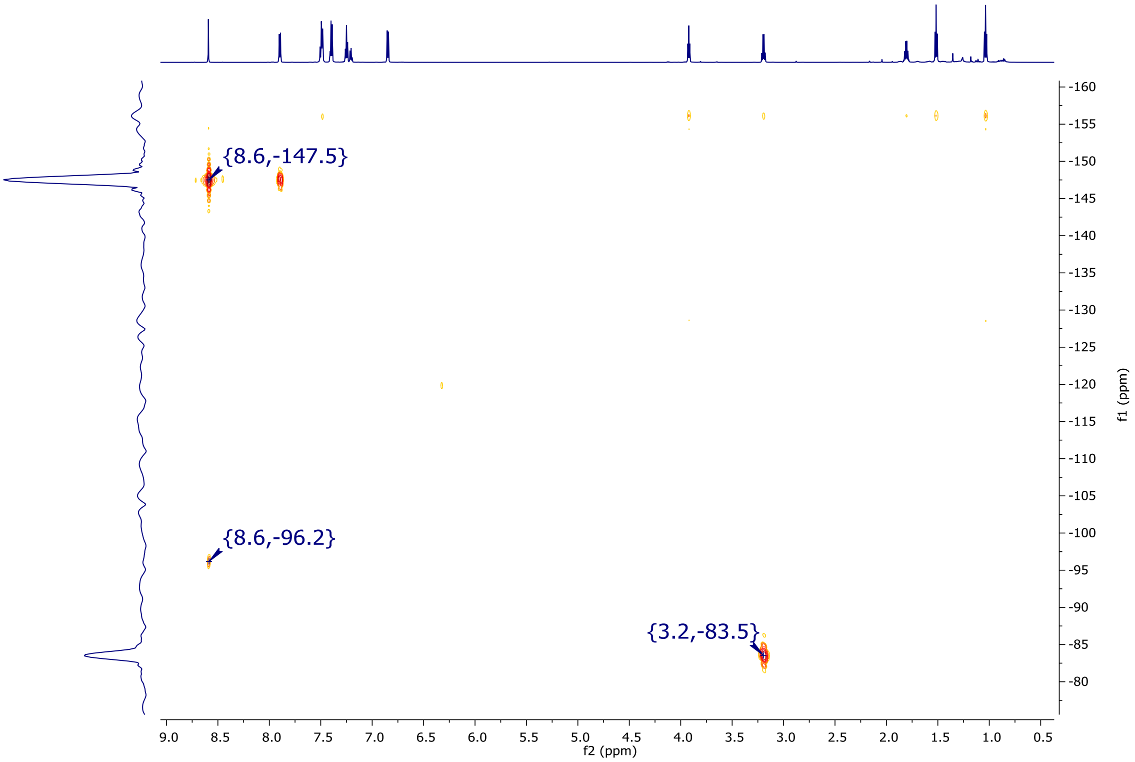

Figure S142. 4-Ethyl-2,6-diphenyl-7-(4-propoxyphenyl)-2*H*-pyrazolo[4,3-*c*]pyridine (**41**). ^{1}H-^{15}N HMBC spectrum (700 MHz, $CDCl_3$).

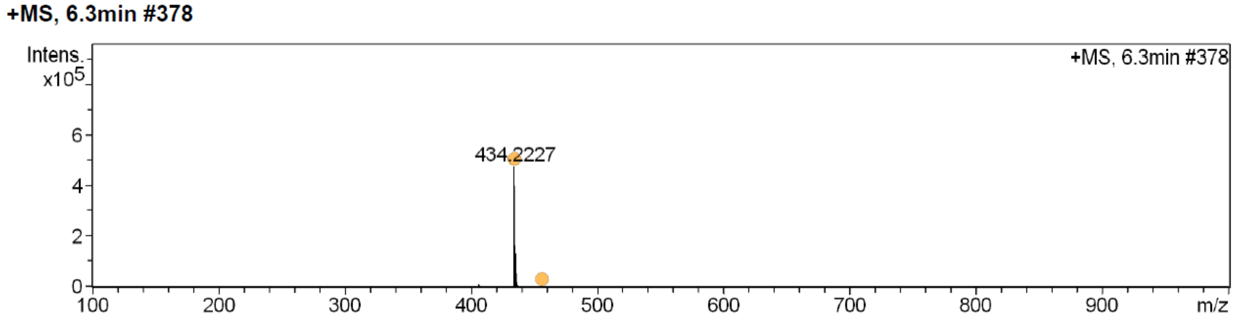

Meas. m/z	#	Ion Formula	m/z	err [ppm]	mSigma	# Sigma	Score	rdb	e⁻ Conf	N-Rule
434.2227	1	C29H28N3O	434.2227	0.1	2.2	1	100.00	17.5	even	ok
456.2041	1	C29H27N3NaO	456.2046	-1.1	30.0	1	100.00	17.5	even	ok

Figure S143. 4-Ethyl-2,6-diphenyl-7-(4-propoxyphenyl)-2*H*-pyrazolo[4,3-*c*]pyridine (**41**). HRMS (ESI-TOF).

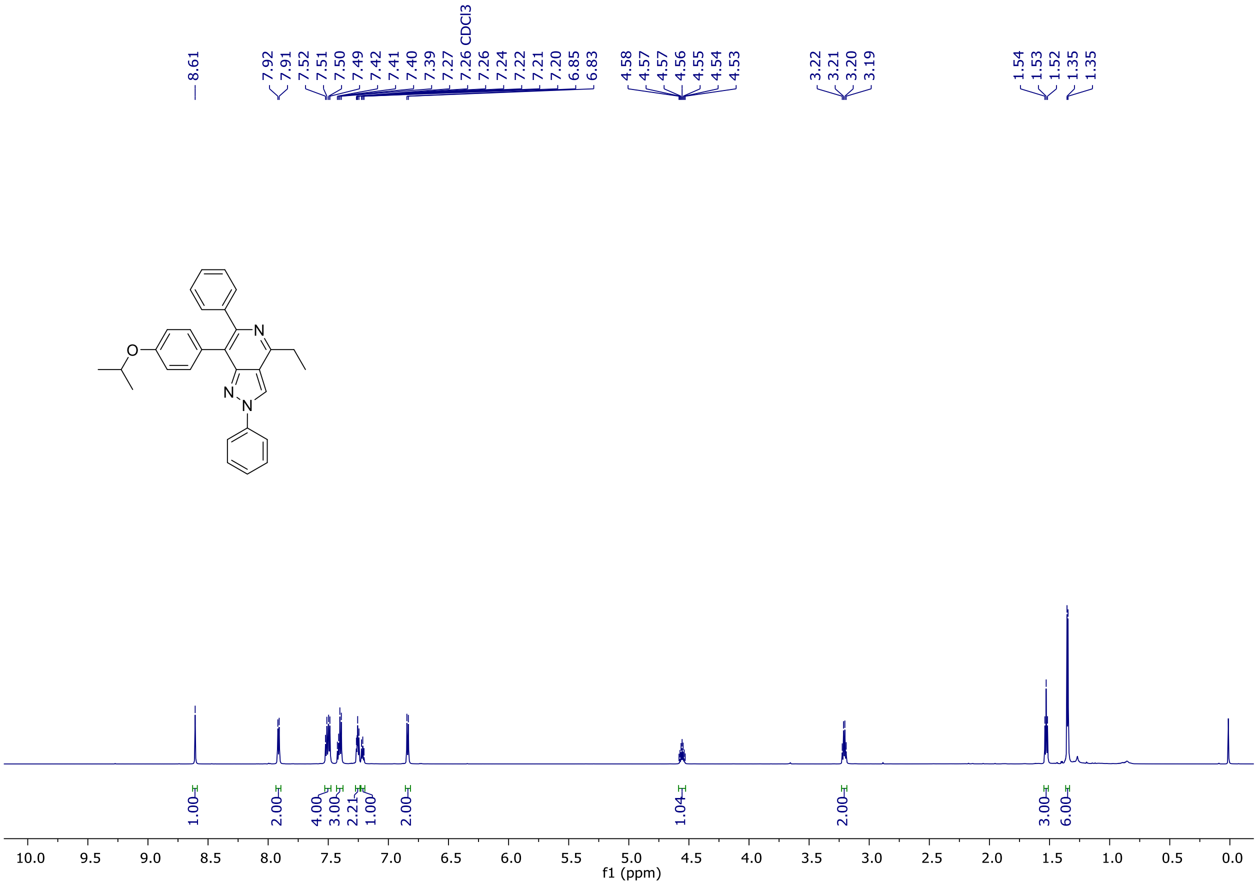

Figure S144. 4-Ethyl-7-(4-isopropoxyphenyl)-2,6-diphenyl-2*H*-pyrazolo[4,3-*c*]pyridine (**42**). ^{1}H NMR spectrum (700 MHz, $CDCl_3$).

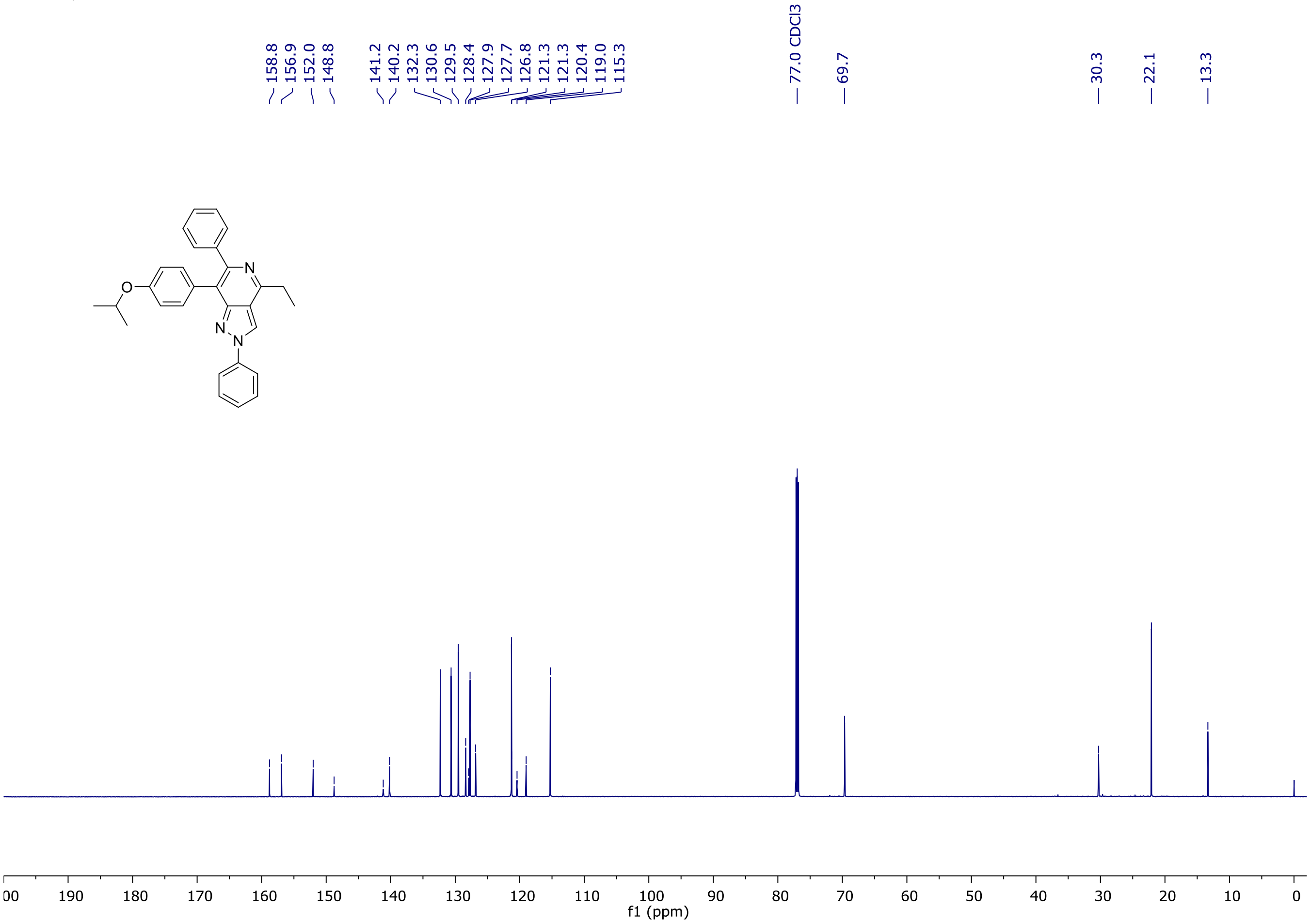

Figure S145. 4-Ethyl-7-(4-isopropoxyphenyl)-2,6-diphenyl-2*H*-pyrazolo[4,3-*c*]pyridine (**42**). ^{13}C NMR (176 MHz, $CDCl_3$).

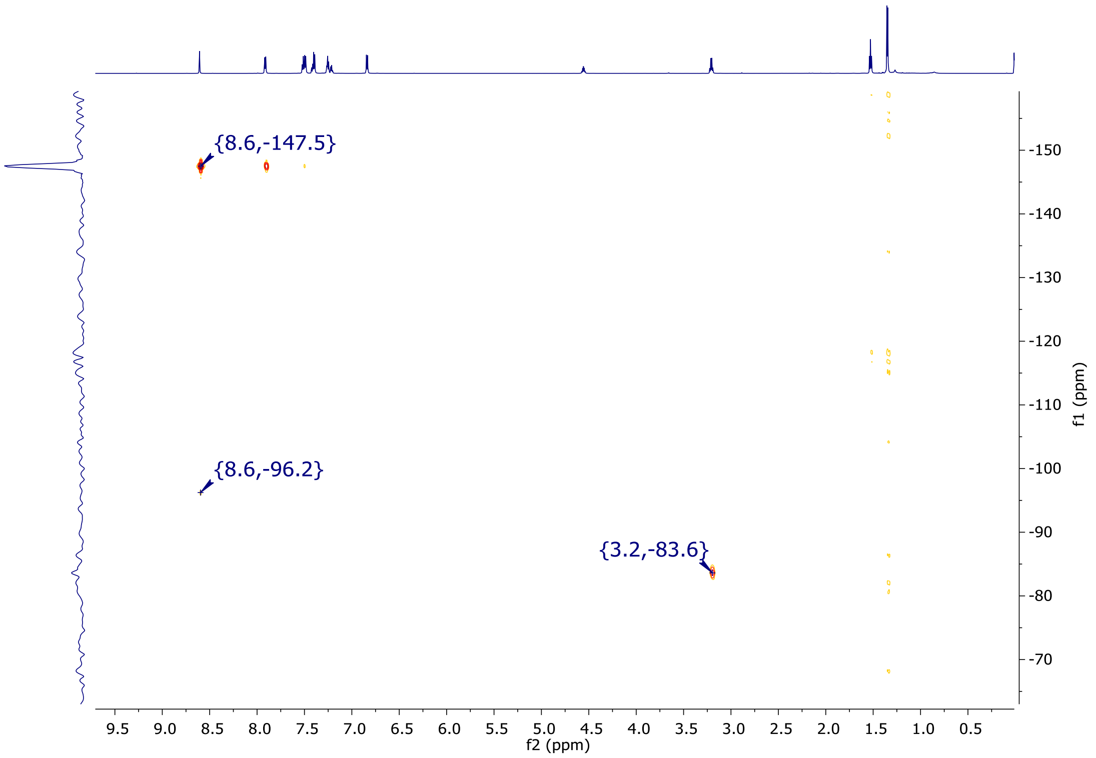

Figure S146. 4-Ethyl-7-(4-isopropoxyphenyl)-2,6-diphenyl-2*H*-pyrazolo[4,3-*c*]pyridine (**42**). ^{1}H-^{15}N HMBC spectrum (700 MHz, $CDCl_3$).

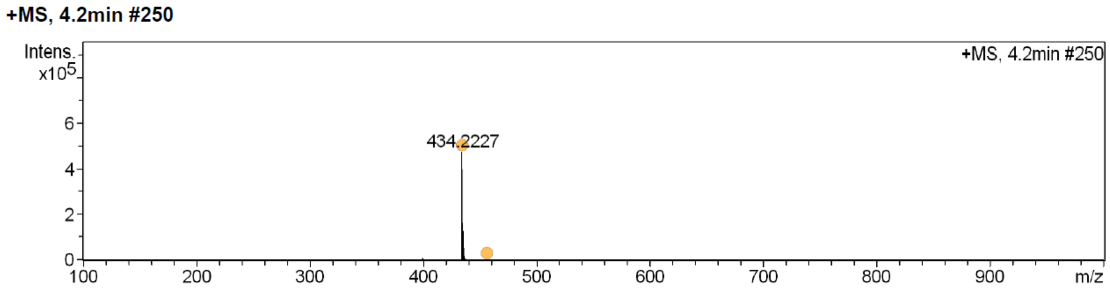

Meas. m/z	#	Ion Formula	m/z	err [ppm]	mSigma	# Sigma	Score	rdb	e⁻ Conf	N-Rule
434.2227	1	C29H28N3O	434.2227	0.1	4.5	1	100.00	17.5	even	ok
456.2052	1	C29H27N3NaO	456.2046	-1.3	31.9	1	100.00	17.5	even	ok

Figure S147. 4-Ethyl-7-(4-isopropoxyphenyl)-2,6-diphenyl-2*H*-pyrazolo[4,3-*c*]pyridine (**42**). HRMS (ESI-TOF).
